# A Soluble Form of the Giant Cadherin Fat1 Is Released from Pancreatic Cancer Cells by ADAM10 Mediated Ectodomain Shedding

**DOI:** 10.1371/journal.pone.0090461

**Published:** 2014-03-13

**Authors:** Nathalie Wojtalewicz, Elham Sadeqzadeh, Jakob V. Weiß, Mahnaz Moradian Tehrani, Susanne Klein-Scory, Stephan Hahn, Wolff Schmiegel, Uwe Warnken, Martina Schnölzer, Charles E. de Bock, Rick F. Thorne, Irmgard Schwarte-Waldhoff

**Affiliations:** 1 Department of Internal Medicine, IMBL, Knappschaftskrankenhaus, Ruhr-University Bochum, Bochum, Germany; 2 School of Biomedical Sciences and Pharmacy, University of Newcastle, Callaghan, Australia; 3 Functional Proteome Analysis, German Cancer Research Center (DKFZ), Heidelberg, Germany; 4 Department of Molecular Gastrointestinal Oncology, Ruhr-University Bochum, Bochum, Germany; 5 Department of Internal Medicine, Knappschaftskrankenhaus, Ruhr-University Bochum, Bochum, Germany; 6 Center for the Biology of Disease, Vlaams Instituut voor Biotechnologie, Leuven, Belgium; West German Cancer Center, Germany

## Abstract

In pancreatic cancer, there is a clear unmet need to identify new serum markers for either early diagnosis, therapeutic stratification or patient monitoring. Proteomic analysis of tumor cell secretomes is a promising approach to indicate proteins released from tumor cells *in vitro*. Ectodomain shedding of transmembrane proteins has previously been shown to contribute significant fractions the tumor cell secretomes and to generate valuable serum biomarkers. Here we introduce a soluble form of the giant cadherin Fat1 as a novel biomarker candidate. Fat1 expression and proteolytic processing was analyzed by mass spectrometry and Western blotting using pancreatic cancer cell lines as compared to human pancreatic ductal epithelial cells. RNA expression in cancer tissues was assessed by *in silico* analysis of publically available microarray data. Involvement of ADAM10 (A Disintegrin and metalloproteinase domain-containing protein 10) in Fat1 ectodomain shedding was analyzed by chemical inhibition and knockdown experiments. A sandwich ELISA was developed to determine levels of soluble Fat1 in serum samples. In the present report we describe the release of high levels of the ectodomain of Fat1 cadherin into the secretomes of human pancreatic cancer cells *in vitro*, a process that is mediated by ADAM10. We confirm the full-length and processed heterodimeric form of Fat1 expressed on the plasma membrane and also show the p60 C-terminal transmembrane remnant fragment corresponding to the shed ectodomain. Fat1 and its sheddase ADAM10 are overexpressed in pancreatic adenocarcinomas and ectodomain shedding is also recapitulated *in vivo* leading to increased Fat1 serum levels in some pancreatic cancer patients. We suggest that soluble Fat1 may find an application as a marker for patient monitoring complementing carbohydrate antigen 19-9 (CA19-9). In addition, detailed analysis of the diverse processed protein isoforms of the candidate tumor suppressor Fat1 can also contribute to our understanding of cell biology and tumor behavior.

## Introduction

Pancreatic ductal adenocarcinoma is the most common malignant tumor of the pancreas and is the fourth ranked cause of cancer-related death worldwide. Considered the most aggressive solid tumor, the mortality rate from pancreatic cancer is high with 5-year survival rates less than 5% [1], [2]. Presently only surgery offers any potential for cure but resection is possible in only 15–20% of patients. Therefore, earlier detection of pancreatic cancer is essential to improve patient outcomes.

Serum biomarkers are highly desirable for early diagnosis, therapeutic stratification and patient monitoring. In the context of pancreatic cancer the carbohydrate antigen 19-9 (CA19-9) also known as sialyl Lewis blood group antigen, is the main serum biomarker used clinically [3]. Serum assays for CA19-9 have limited diagnostic value and can not be used as a screening assay alone ([4] and references therein) but provide important information with regards to prognosis, response to chemotherapy and as an early indicator of post-operative recurrence. The serial determination of CA19-9 levels can detect disease recurrence months before clinical or radiological evidence. Moreover, a decline of CA19-9 in response to chemotherapy may serve as surrogate marker for clinical response [4] (for review see [5]–[7]). However, several confounding variables limit the clinical utility of CA19-9.

The highest CA19-9 levels are detected in patients with biliary obstruction, regardless of whether obstruction is due to cancer or to benign causes [8], [9]. Increased CA19-9 levels are also associated with pancreatitis, liver cirrhosis, cholangitis and multiple adenocarcinomas of other type, e.g. colorectal cancer. Importantly, the expression of CA19-9 depends on a Lewis positive phenotype, with false negative results common mostly due to approximately 7–10% of Caucasians and up to 20% of Africans being *Lewis* antigen negative where CA19-9 is undetectable regardless of tumor burden [10], [11]. Hence there is a clear unmet need to identify new serum markers for either early diagnosis, therapeutic stratification or patient monitoring that have increased utility or can complement with CA19-9 or other serum markers [8].

One approach for biomarker discovery that we and others have utilized, is the interrogation of the complete repertoire of proteins released from cancer cells *in vitro* – the cancer cell secretome [12]–[15]. Proteomic analyzes of secretomes have found thousands of proteins and somewhat surprisingly, among them significant fractions of transmembrane (TM) proteins. This is due first, to the release of microvesicles that carry intact TM proteins. Secondly, TM proteins can be processed to a soluble form by proteolytic processing [16]–[18]. We have previously found that both microvesicular release [19] and proteolytic cleavage of TM proteins occurs not only *in vitro*, but also *in vivo*. Specifically we determined an increase in serum levels of soluble cadherins, namely of soluble E-cadherin [20] and of soluble LI-cadherin (unpublished Data) in patients with colorectal carcinoma.

In this report we have analyzed the secretome of pancreatic cancer cells *in vitro* and describe the identification of a soluble form of Fat1 cadherin as a highly abundant constituent of this fraction. Fat1 belongs to a small subfamily of four vertebrate genes (Fat1, Fat2, Fat3 and Fat4). Fat cadherin genes encode extremely large proteins of ∼500–600 kDa with conservation of structure from invertebrates to mammals. Each member is comprised of up to 34 cadherin repeats, one or two lamininG-like motifs and several epidermal growth factor (EGF)-like motifs in their extracellular region, a single-pass TM domain and a large cytoplasmic domain [21]–[23]. Proteolytic processing of Fat proteins occurring in the early secretory pathway and producing a non-covalently bound heterodimer in the cell membrane has previously been described. It is referred to as “classical” processing and appears to be conserved between Drosophila [24] and man [22], [25].

Fat1 has not previously been investigated in pancreatic cancer. Here, we present the first description of a soluble isoform of Fat1 released from pancreatic cancer cells *in vitro*. We found that A Disintegrin and metalloproteinase domain-containing protein 10 (ADAM10) is largely responsible for the release of this ectodomain fragment. *In silico* analysis of publically available expression array data indicated overexpression of Fat1 as well as ADAM10 in pancreatic adenocarcinomas. Lastly, we developed an ELISA-based assay able to measure the ectodomain of Fat1 and demonstrate that increased levels of soluble Fat1 can be detected in the serum of a proportion of patients with pancreatic adenocarcinoma as compared to unaffected controls.

## Materials and Methods

### Ethics statement

Tissue samples were obtained from patients of the Department of Internal Medicine, Knappschaftskrankenhaus, Ruhr-University Bochum, Germany, after informed consent was obtained. The study was approved by the local ethics review board of the Ruhr-University Bochum and was conducted according to the declaration of Helsinki. See table S5 for patient details.

Written informed consent from all patients and tissue donors was documented according to the local ethics guidelines. The three cancer samples were drawn from patients with PancCa stages UICC IIB. Normal tissue controls were from the same patients' adjacent healthy tissue.

Serum samples were obtained from patients of the Department of Internal Medicine, Knappschaftskrankenhaus, Ruhr-University Bochum, Germany, after informed consent was obtained. The study was approved by the local ethics review board of the Ruhr-University Bochum and was conducted according to the declaration of Helsinki. See table S4 for patient details.

Written informed consent of all patients and blood donors was documented according to the local ethics guidelines. The 30 cancer samples were drawn from patients with PancCa stages UICC I (1), UICC II (9), UICC III (2) and UICC IV (18). A group of 26 patients with negative diagnostic results for cancer were used as (clinical) controls.

### Cell culture

The human pancreatic adenocarcinoma cell line Paca44 [26] was kindly provided by M. Löhr (Heidelberg, Germany), BxPc3, MiaPaca2 and Panc1 were obtained from the American Type Culture Collection (ATCC, Rockville, MD) and A818-4 cells were from our lab (W.S.). Cells were maintained in supplemented Dulbecco's Modified Eagle Medium (DMEM) as described previously [19]. Human pancreatic ductal epithelial (HPDE) cells were kindly provided by M.S. Tsao (Toronto) and cultured in defined Keratinocyte-SFM (KFSM, Life technology) as described previously [19].

### Preparation of protein samples

Secretome preparation was performed as previously described [20]. In brief cells were cultured in standard medium until they reached a confluence of 60–70%. Afterwards they were washed three times with DMEM and incubated in serum-free medium with supplements for either 16 h for MS analysis or another two days for western blot analysis. Supernatants were harvested and cleared from floating cells and debris by sterile filtration. To prevent proteolytic digestion, proteinase inhibitors were added. Secretome proteins were concentrated by ultrafiltration.

For cell culture lysate preparation cells were lysed with NP-40 buffer (25 mMTrisHCl, pH 7.4, 0.5% NP-40, 100 mM NaCl, 1 mM EDTA; 45 min 4°C), with CHAPS-lysis buffer (100 mM NaCl, 10 mM Tris, 1 mM MgCl_2_, 10% Glycerol, 1% CHAPS 90 min, 4°C) or with NDE-lysis buffer (10 mMTris/HCl, pH 7,2, 66 mM EDTA, 0,4% SDS, 1% NP-40, 1 h, 4°C) containing a protease inhibitor cocktail (Roche, Basel, Switzerland) and 1 mM PMSF. The supernatant was collected after centrifugation (14.000 rpm, 10 min 4°C). Protein concentration was determined in a standard Bradford protein assay (BioRad, Hercules, CA, USA).

### Frozen tissue extraction

A small piece of frozen tumor or normal tissue was broken off and pulverized while cooled with liquid nitrogen. The powder was covered with NDE buffer and protein extraction was performed as described above.

### ADAM and MMP inhibition

The ADAM10 specific inhibitor GI254023X [27], [28] and the broad spectrum metalloproteinase inhibitor Batimastat (Tocris Bioscience, Bristol, UK) were both dissolved in DMSO as stock solutions prior to dilution and use. Cells were cultivated until they reached a confluence of approximately 60–70%. They were washed and afterwards incubated with 10 µM Batimastat or 5 µM GI254023X in serum-free medium for two days.

### siRNA-knockdown studies

For siRNA experiments cells were grown to a confluency of approximately 30% and incubated in antibiotic-free medium for one day. Afterwards they were incubated with 30 µM either ON-TARGETplus siRNA or Dharmacon ON-TARGETplus nontargeting siRNA as a control using Dharmafect (Fermentas, ST. Leon Rot, Germany) for two days followed by incubation in serum-free medium for another two days and a second incubation in serum-free medium for another two days.

### Antibodies and Western blotting

Non-commercial anti-Fat1 mouse monoclonal and rabbit polyclonal antibodies directed against the N-terminal or C-terminal domains of Fat1 were previously described [25]. Polyclonal antibodies against the extracellular domain of Fat1 (HPA001869 and HPA023882) and monoclonal antibodies against β-tubulin (T4026) were purchased from Sigma-Aldrich (St. Louis, MO, USA). ADAM10 antibodies were purchased from Calbiochem (Darmstadt, Germany) (rabbit, 735–749) and Millipore (Darmstadt, Germany) (rabbit AB19026). The E-cadherin antibody was purchased from Invitrogen (Darmstadt, Germany) (mouse, 13–1700).

For Western blot analysis, 8 µg total protein from secretomes, 50–75 µg from cell lysates and tissue or 12 µg serum proteins were separated using 3–8% Tris-Acetate gradient gels (Life Technologies, Darmstadt, Germany) as previously described [25] with some modifications. Briefly, proteins were transferred to nitrocellulose or PVDF membranes using semi-dry blotting and the membranes were blocked for 1 h with 5% skim milk powder in PBS. After incubation with the indicated primary antibodies, detection of immunoreactive bands was performed using appropriate secondary antibodies coupled with infrared fluorophores (Alexa-Fluor 680 (Life Technologies, Darmstadt, Germany) or DyLight 800 (Thermo Scientific, Schwerte, Germany)) or HRP (BioRad laboratories, München, Germany). Signals were detected using the Odyssey Infrared Imaging System (LI-COR Biosciences, Lincoln, NE) or a Fuji LAS-4000 imaging system (GE Healthcare, Braunschweig, Germany).

### Electrospray ionization-tandem mass spectrometry (ESI-MS/MS) analysis

For a detailed description see materials and methods S1. Briefly secretome and serum samples separated on PAGE gels were stained with Krypton (Pierce, Rockford, USA) and each protein lane cut into 29 slices and digested with trypsin. Tryptic peptide mixtures were separated using a nanoAcquity UPLC system (Waters GmbH, Eschborn, Germany) as previously described [19]. The nano UPLC system was coupled online to an LTQ Orbitrap XL mass spectrometer (Thermo Scientific, Bremen, Germany). The mass spectrometer was operated in the sensitive mode. Themgf-files generated by Xcalibur software (Thermo Scientific,Bremen, Germany) were used for database searches with the MASCOT search engine (Matrix Science, London, UK; version 2.2) against MSIPI database. Each slice was analyzed separately and MS/MS data were not merged prior to protein database search to maintain the information about molecular weight of each protein, peptide matches and identification score. In this way protein catalogs from human pancreatic cancer cell secretomes (A818-4, BxPC3, MiaPaca2, Paca44, Panc1 and HPDE (human pancreatic ductal epithelial)) were established.

### ELISA assay against secreted Fat1

96-well plates (white MaxiSorp flat-bottom 96-well plates (Nunc, Roskilde, Denmark) were first coated overnight at 4°C with 100 µL of anti-Fat mAb NTD-7 (raised against cadherin domains 11/12; [25]) at 50 µg/ml in carbonate buffer (pH 9.6). Between all incubation steps, plates were washed three times with phosphate buffered saline containing 0.1% Tween (v/v) (PBS-T). Plates were then blocked with 5% skimmed milk in PBS-T (2 h, RT) before application of indicated samples and further incubation (2 h, RT). Captured Fat1 antigen was detected by 0.05 µg/mL of biotinylated anti-Fat1 NTD-14 mAb (16 h, 4°C) (raised against a peptide in cadherin repeat 12 of Fat1 [25] diluted in 2% skimmed milk in PBST. Complexes were detected using 5 µg/mL Neutravidin-HRP (Pierce, Darmstadt, Germany) for 2 h at room temperature before shaking incubation of 50 µl substrate (SuperSignal ELISA FemtoMaximum Sensitivity).

## Results

### The giant cadherin Fat1 is a major component of the secretome of pancreatic cancer cells

As part of our biomarker discovery program we have catalogued the repertoire of proteins released from five human pancreatic cancer cell lines using mass spectrometry (A818-4, BxPC3, MiaPaca2, Paca44 and Panc1) and compared this to the secretome of immortalized human pancreatic ductal epithelial (HDPE) cells. Secretomes were prepared as previously described [12], [13], [19] and separated on a 1D-gradient gel. Each gel lane was cut into 29 slices. Tryptic digestion and mass spectrometry were performed separately for every gel slice and database searches led to the identification of more than 1000 proteins per secretome and over 3000 unique proteins in total (unpublished data).

Notably, numerous peptides mapping to the giant protocadherin Fat-1 were discovered in all six secretomes within gel slices two and three corresponding to proteins >400 kDa consistent with the predicted mass of Fat1 of ∼500 kDa (compare table 1). Indeed, in terms of numbers of peptides identified, which is a relative measure of protein abundance, Fat1 was amongst the most abundant proteins in the cancer cell secretomes representing up to 0.9% of all peptides and the most abundant derivative of any TM protein. The distribution of Fat1 peptides among the cell lines and peptides derived from other Fat proteins (Fat2 and Fat4) is shown in table 1.

**Table 1:** Fat1 cadherin is a major component of the secretome of pancreatic cancer cell lines.

	Secretomes					
	HPDE	A818-4	BxPc3	MiaPaCa2	Panc1	PaCa44
Identified gene symbols	972	1009	1144	1120	1060	1176
All peptide matches	23.935	19.454	24.051	19.785	26.105	23.859
Peptide matches Fat1	39	89	91	51	232	210
Rank/%	152/0.2	62/0.5	61/0.4	120/0.3	21/0.9	12/0.9
Peptide matches Fat2	92	0	170	0	0	0
Rank/%	78/0.4	0/0	28/0.7	0/0	0/0	0/0
Peptide matches Fat4	0	0	0	0	8	0
Rank/%	0/0	0/0	0/0	0/0	602/0.03	0/0

Abundance of Fat1 derived peptides in the secretome fraction of five pancreatic cancer cell lines (A818-4, BxPc3, MiaPaCa2, Panc1, PaCa44) compared to normal immortalized pancreatic cells (HDPE) determined using MS (see materials and methods S1). The table shows the number of identified Fat1 peptides for each cell line analyzed as well as the rank and the percentage of all identified matches.

We next profiled the mRNA expression of all four Fat family cadherins in the pancreatic cancer cell line panel using microarray analysis (table S1) followed by secondary confirmation using quantitative PCR (figure S1). Fat1 was expressed at high levels in all six cell lines, Fat2 and Fat4 at moderate levels in only two of the six cell lines. In contrast only background levels were obtained for Fat3 in this analysis.

The determined levels of mRNAs mostly corresponded with the proteomic data, however comparing cancer cells to HPDE cells there were some discrepancies. In particular, HDPE cells displayed high Fat1 mRNA expression but conversely gave the lowest number of Fat1 peptides in the secretome. Likewise, peptide numbers for Fat2 and Fat4 in the HPDE secretome are lower or even absent, respectively, when compared to the cancer cell lines that also express comparable levels of Fat2 and Fat4 mRNAs (BxPC3 and Panc1, respectively) suggesting that cancer cells release more Fat proteins than normal cells.

**Figure 1:**
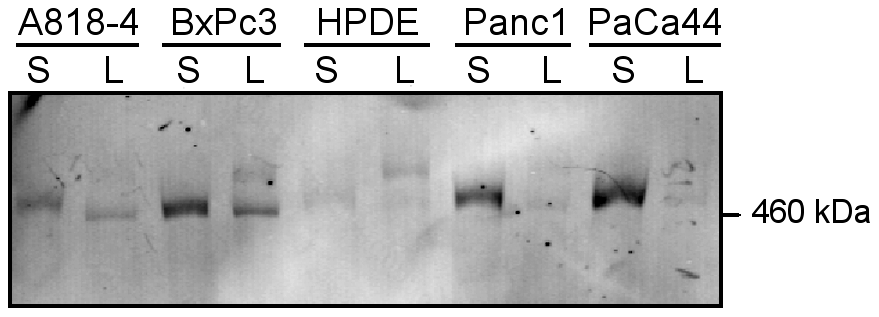
Fat1 cadherin in secretome and lysate fractions of pancreatic cancer cell lines. Western blot analysis of Fat1 in the respective secretome (S; 8 µg loaded) and lysate (L; 50 µg loaded) fractions from four pancreatic cell lines and HPDE. The blot was probed with an antiserum raised against the extracellular domain of Fat1 (ECD1).

To independently validate these findings we undertook Western blot analysis of the pancreatic cell secretomes and compared these with corresponding cell lysates. Detection with antibodies directed against the aminoterminus of the Fat1 protein revealed predominantly a single band slightly over 460 kDa in the secretomes with corresponding cell lysates from cancer cells displaying a band of similar mobility (figure 1). In contrast, HDPE cells exhibited little reactivity for Fat1 in the secretome and the main band detected in the lysates appeared larger suggesting there may be post-translational differences. Since the band intensities in the secretomes correlated with the peptide counts determined for each respective cell line (table 1), taken together these data provide good evidence that Fat1 is highly abundant in the secretomes of pancreatic cancer cell lines but less so in their normal cellular counterparts.

### Fat1 in the pancreatic cancer secretome consists of the shed extracellular domain

**Figure 2:**
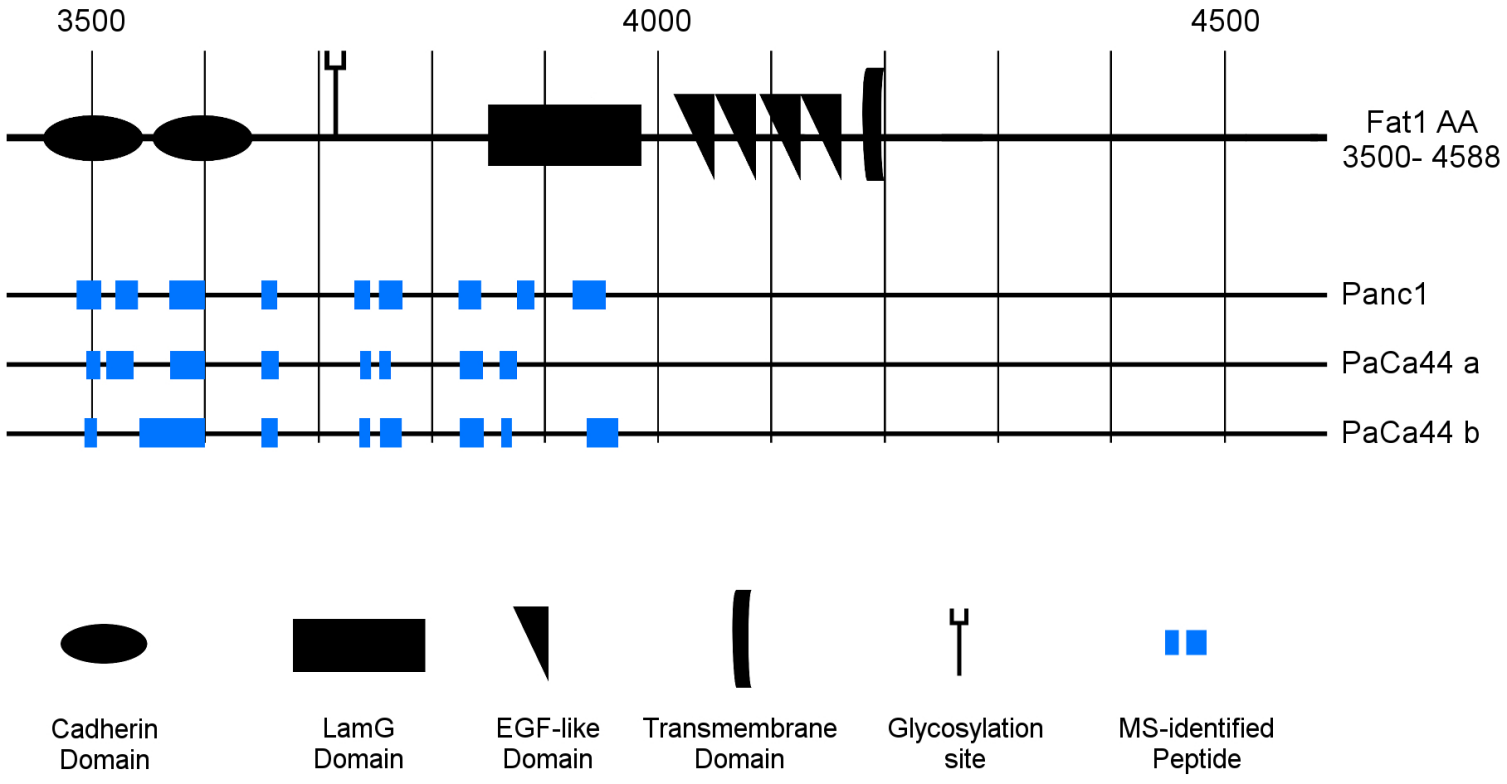
Mass spectrometric analysis of secretomes exclusively indicates peptides derived from the extracellular domain of Fat1. Mass spectrometric analyses were performed with all six secretomes and Fat1 specific peptides were detected beginning near the amino terminus down to and including the EGF domain (see file S1 for details). Shown here are alignments of Fat1 specific peptides from three exemplary results with high sequence coverage in the section of amino acids 3500 down to the C-terminus at AA4588 (according to HPRD).

The apparent molecular range (Mr) of the Fat1 band in pancreatic cancer secretomes is in the range of the predicted Mr of the full-length core Fat1 polypeptide of 505 kDa (figure 1). While nominally this suggests that secreted Fat1 might be intact, alignment of the identified peptides to the Fat1 amino acid sequence revealed that all peptides exclusively derive from the extracellular domains (ECD, amino acids 1–4180) with the most C-terminal amino acid identified in a peptide mapping to AA 3997 (fig. 2 and file S1).

Moreover, the existence of a larger band in the HPDE cell lysate suggested, that posttranslational modifications may contribute to the total size of the protein isoforms as often is observed for TM proteins including many cadherins. Indeed, deglycosylation experiments confirmed that approximately an additional 70 kDa of the total protein mass are due to glycosylation of the cellular forms and of the soluble form of Fat1 (figure S2). Concerning the signals in cell lysates, the very high Mr band present in HPDE cells suggests these predominantly express the full-length glycosylated protein. The slightly smaller band present in the cancer cell lysates may represent the N-terminal part of the recently described Fat1 heterodimer, that consists of an N-terminal gp 490 (p420) and a p85 C-terminal transmembrane fragment. This non-covalently bound heterodimer is produced by endoproteolytic S1-cleavage in the trans-Golgi network [25] before reaching the plasma membrane. To clarify the nature of Fat1 present in the secretome and to confirm the identities of bands observed in cell lysates, we undertook Western blot analyzes with domain specific antibodies [25].

**Figure 3:**
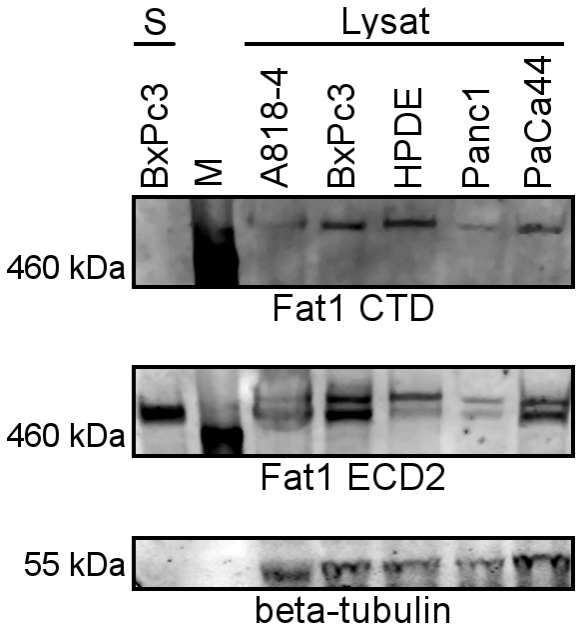
Western blotting indicates the two known forms of Fat1 and a new released form. Cell lysates (75 µg each) and a representative secretome (8 µg) were blotted together with a protein marker (M). The same blot was probed first, with an antiserum raised against the cytoplasmic domain (CTD) of Fat1 and subsequently with an antiserum raised against the extracellular domain (ECD 2). Beta-Tubulin, finally, was used as a loading control for cell lysate. The extracellular domain specific antibodies, only, detect the processed cellular form and the released Fat1 in the secretome.

Figure 3 compares a representative secretome fraction alongside cell lysates from cultured pancreatic cells. The blot was probed with antibodies directed against the intracellular domain of Fat1 that readily detects a single band of >500 kDa in the cell lysates in clear contrast to the secretome where no signals were evident. Next, the same blot was incubated with antibodies directed against an extracellular Fat1 domain where pancreatic cell lysates gave two closely spaced bands in cell lysates: the upper band being identical to the one detected by intracellular antibodies. The lack of reactivity of the lower band with intracellular domain antibodies identifies this doublet of bands as the full length and heterodimeric forms of Fat1, respectively (figure 3). Furthermore, as expected from the MS-results, extracellular domain antibodies gave strong signals in the secretome sample. Collectively, the results of both biochemical and mass spectrometric (MS) analyzes indicate that Fat1 present in the secretome entirely lacks C-terminal residues. Since both known forms of cellular Fat1 are TM proteins, the compelling conclusion from these data is that the Fat1 detectable in the secretome represents extracellular shedding through proteolytic cleavage in its extracellular domain downstream from the lamG domain. By Western blotting of cell lysates we could not find evidence for the presence of a C-terminal remnant fragment expected to arise from ectodomain shedding, neither did we detect the p85 C-terminal fragment from the heterodimer.

**Figure 4:**
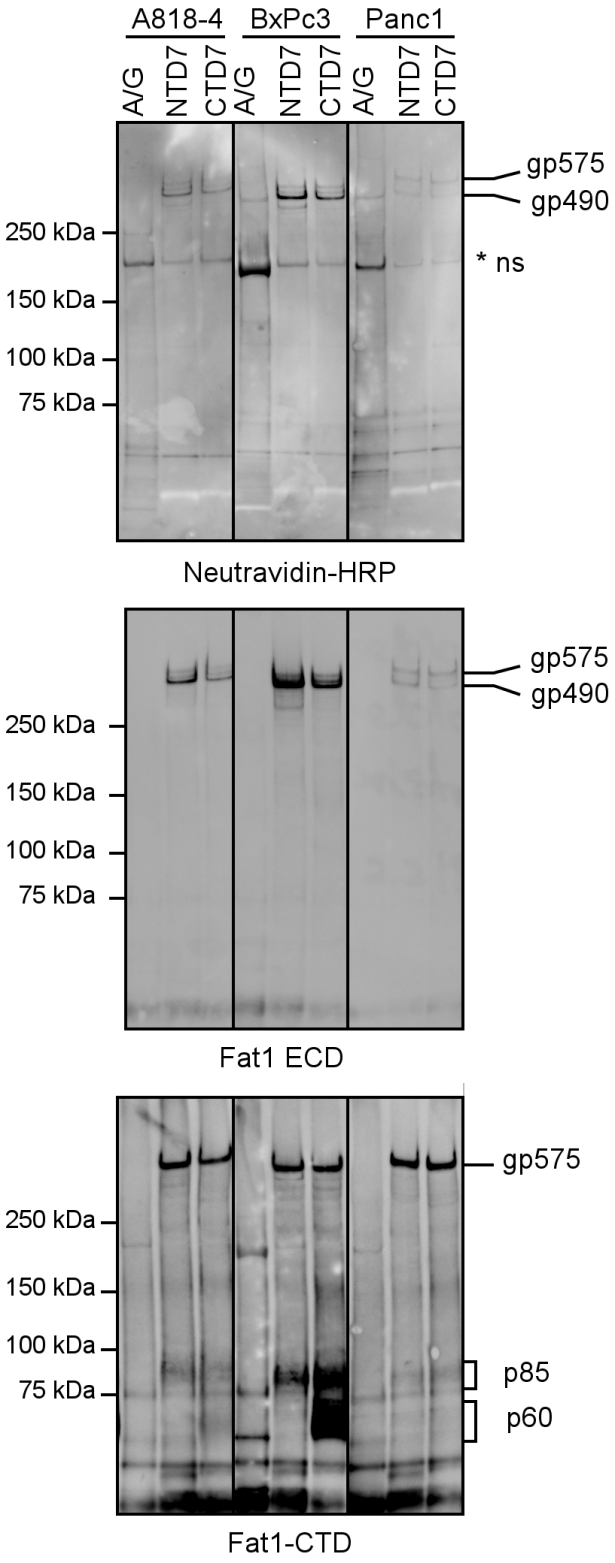
Immunoprecipitation followed by Western blotting indicates two different C-terminal remnant fragments. Cells (Panc1, BxPC3 and A818-4) were labeled with biotin, lysed and precipitated with Fat1 specific monoclonal antibodies (NTD7 and CTD7); protein A/G beads were used as a control. The first incubation of the blot with Neutravidin-HRP indicated bands with high molecular weight corresponding to the known high Mr forms of Fat1 and a presumably crossreactive protein at ≈200 kDa. Subsequent Western blotting with the extracellular domain specific antiserum again detects the two high Mr forms of Fat1. Finally, Western blotting with the intracellular domain specific antiserum detects the full-length protein and the p85 C-terminal remnant fragment derived from classical processing in both Fat1 precipitates, confirming the association of the N-terminal and C-terminal parts. An additional p60 is exclusively detected in the precipitate using the intracellular domain-specific antibody with strong signals in BcPC3 and week signals in A818-4 lysates. Immunoprecipitation (IP) analysis of Fat1 after cell surface labeling of Panc1, BxPC3 and A818-4 cells with biotin. IPs using domain specific mAbs against Fat1 along with a control (protein A/G beads incubated with lysates) were first probed with Neutravidin to decorate proteins on the cell surface followed by the polyclonal antibodies against the ECD and CTD as shown. Bands are labeled gp575, gp490 and p85 as per A) with p60 denoting a further proteolytic product.

Thus, we next performed immunoprecipitation analysis with domain specific monoclonal antibodies (mAbs) using three representative adenocarcinoma cell lines. Using biotinylation to label cell surface proteins, this analysis shows the presence of primarily two high Mr bands precipitated by N- and C-terminal specific mAbs in the majority of cell lines examined. Subsequent Western blotting with an extracellular domain specific antibody detects the larger band only, whereas the intracellular domain specific antibody marks both large cellular forms (figure 4). This provides further evidence of dual processing whereby both full length and heterodimeric forms of Fat1 occur on the cell surface of pancreatic adenocarcinoma cells as previously described for melanoma cells. Notably, the intracellular C-terminal domain specific antibody also showed the presence of the p85 band indicative of the heterodimer (figure 4). Most strikingly an ∼60 kDa band consistent with the expected ectodomain shedding cleavage site was observed in the BxPC3 cell lysate and to a lesser extent in PaCa44 cells. The p60 band was only precipitated with C-terminal-specific mAbs indicating it represents a product no longer in association with the extracellular domain. This is also consistent with previous observations in melanoma cells [25].

### Shedding of Fat1 cadherin involves ADAM10

Metalloproteases often regulate ectodomain shedding of TM receptors with membrane-anchored ADAM family metalloproteases frequently involved. mRNA profiling of the pancreatic cell lines revealed a restricted expression pattern of ADAM family members (table S2). ADAM10 in particular is the sheddase being largely responsible for ectodomain shedding of E-cadherin and LI-cadherin in colorectal cancer cells (unpublished data) and four of the five cancer cell lines displayed higher ADAM10 levels than HPDE cells. At the protein level, ADAM10 specific peptides were not detected in secretome catalogues but in the enriched exosomes by MS, namely 9 matches in HPDE exosomes and 62 matches in Panc1 exosomes; no other ADAMs were detected in these catalogues (data not shown). Together these data provide a rationale to examine the role of ADAM10 in Fat1 ectodomain shedding.

**Figure 5:**
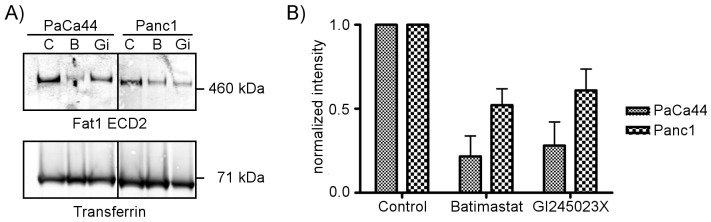
Metalloproteases are involved in Fat1 ectodomain shedding. The figure in A) shows a representative Western blot. PaCa44 and Panc1 cells were incubated in serum free medium for two days with the addition of either the broad spectrum metalloprotease inhibitor Batimastat (B, 10 µM), or the ADAM10-specific inhibitor GI245023X (GI, 5 µM) or solvent as a control (C, DMSO). Secretomes, 8 µg protein per sample, were analyzed by Western blot analysis using the Fat1 ECD2 polyclonal antibody. Blotting against transferrin was used as a loading control. (B) Fat1 specific signals from three independent experiments were quantified and normalized with the transferrin signals. The bar graphs show the mean values +/− S.E.M. from three experiments.

To assess the involvement of metalloproteinases and particularly ADAM10 in Fat1 ectodomain shedding, human pancreatic cancer cells were treated with the broad spectrum protease inhibitor Batimastat and the ADAM10 specific inhibitor GI245023X [28], [29]. Western blotting of Fat1 in secretomes in Paca44 and Panc1 cell lines showed Batimastat treatment reduced the levels of ectodomain shedding of Fat1 to below control levels. The GI254023X inhibitor was similarly effective reducing the levels of ectodomain shed Fat1 in both cell lines (figure 5A). Quantitative assessment of the effects of these agents in three successive experiments showed that Batimastat significantly inhibited Fat1 shedding in Panc1 and Paca44 cells (figure 5B) thereby confirming the role of metalloproteinases in this process. The effects of GI254023X were similar suggesting that ADAM10 is involved in Fat1 shedding in these cells. However, some reports have suggested that GI254023X at the concentrations used may also have some effect on matrix metalloproteases (MMPs), e.g. [27], and further experiments were conducted with RNAi against ADAM10.

**Figure 6:**
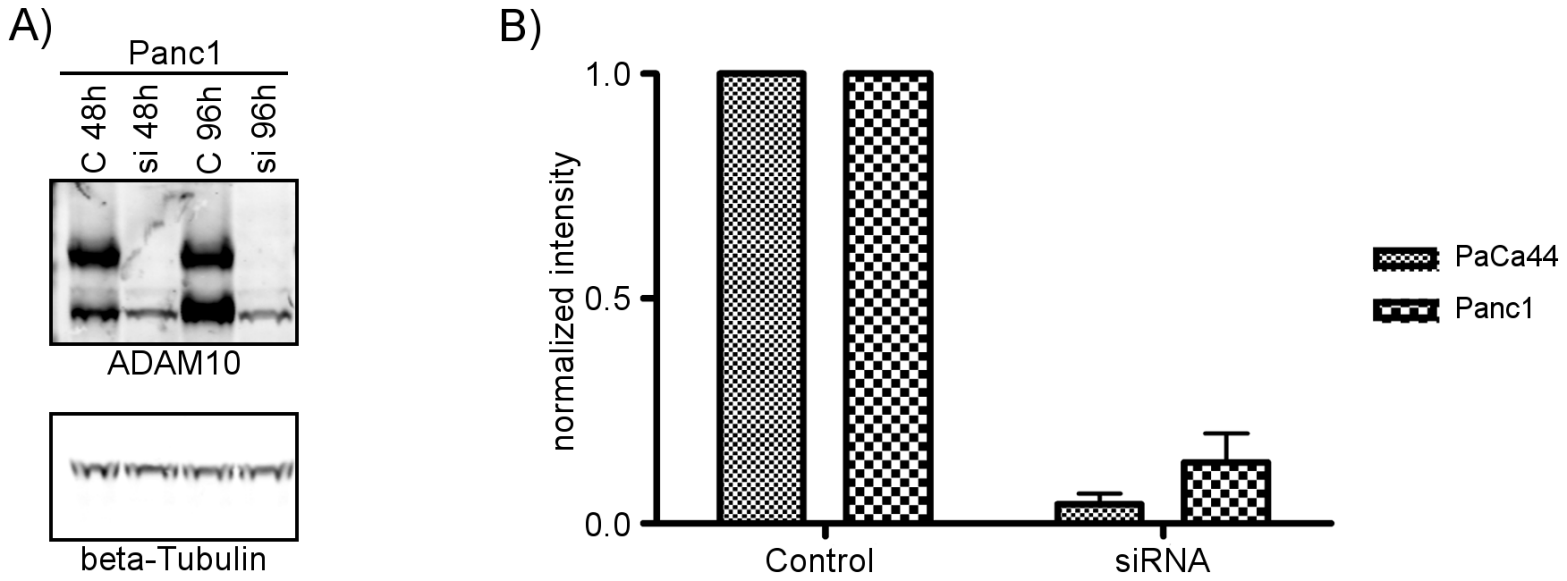
ADAM10 knockdown is efficient and stable for 96 h. ADAM10 expression was suppressed by siRNA mediated knockdown experiments using ADAM10-specific and control siRNA pool's (30 µM each). A) Western blot analysis (75 µg of cell lysates) confirms efficient knockdown of B) Two independent experiments provided similar results as indicated by the normalized quantification.

**Figure 7:**
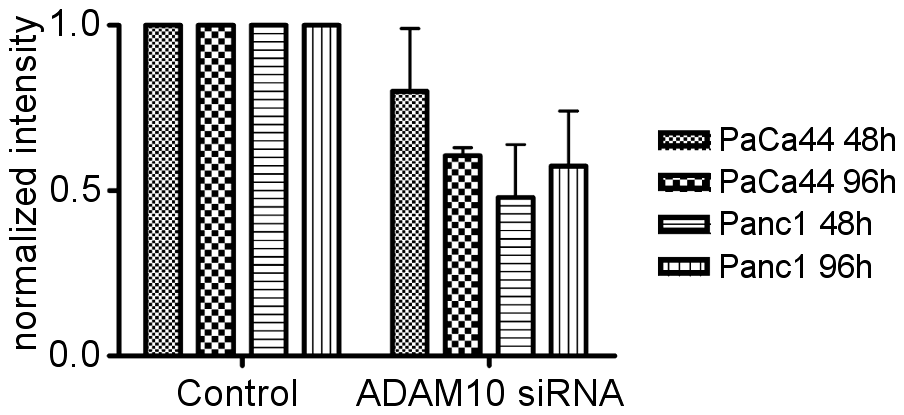
ADAM10 mediates Fat1 ectodomain shedding. Western blot analysis of secretome fractions (8 µg) from PaCa44 and Panc1 cells after ADAM10 knockdown (compare Figure 6) showed significant reductions in Fat1 ectodomain shedding. Again, the two experiments provided similar results as indicated by the normalized quantification.

**Figure 8:**
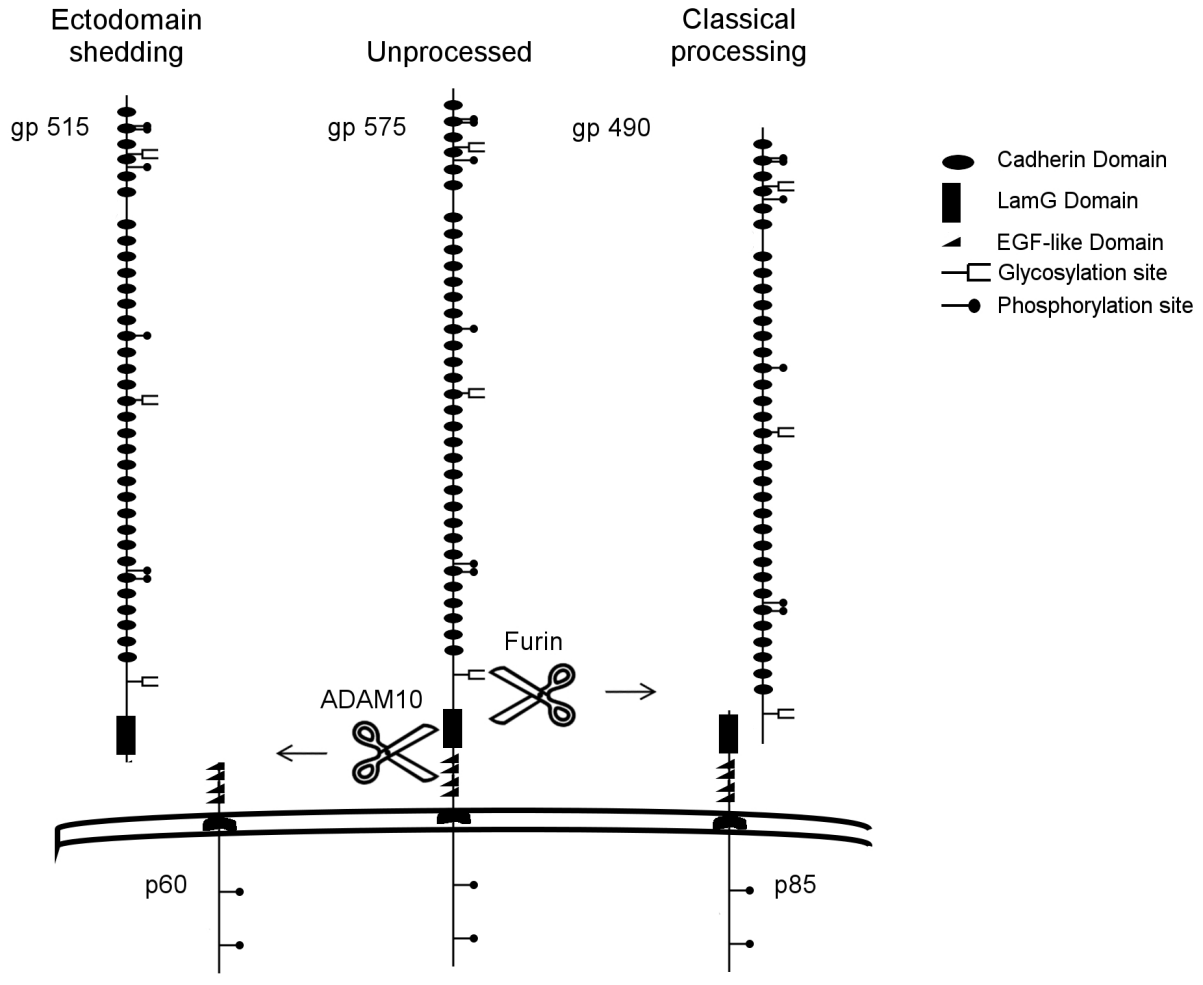
Alternative Fat1 processing in pancreatic cancer cells. Full-length Fat1 can be expressed on the cell surface; alternatively, the full length protein can be cut by a furin-type protease to yield the membrane-integrated heterodimer as described by Sadeqzadeh et al. and designated as “classical processing”. Another alternative is ectodomain shedding as described in this work, where the full-length protein is cut by ADAM10 into a p60 C-terminal remnant fragment (which may be further processed) and a gp515 soluble form released into the secretome.

Treatment of Paca44 and Panc1 pancreatic cancer cells with ADAM10 specific siRNA duplexes resulted in >90% knockdown of ADAM10 protein levels stable for 96 h (figure 6). Examination of the secretome showed a concomitant decrease in soluble Fat1 levels produced by Panc1 and Paca44 cells (figure 7). Even though ADAM10 levels were reduced to below 10% of control levels the maximal reduction in Fat1 secretion observed was around 50% of controls. Similar reductions in the secretion of E-cadherin, a known target of ADAM10, were observed in parallel experiments undertaken with the chemical inhibitors or ADAM10 siRNA duplexes (figure S3 A and B). Collectively this suggests that ADAM10 is a significant effector of Fat1 shedding but not the only one involved. In conclusion ADAM10 mediated ectodomain shedding of Fat1 represents a novel mechanism producing a soluble form of this giant protocadherin that appears to be abundantly produced by human pancreatic adenocarcinoma cells *in vitro* and less so by their normal counterparts (figure 8).

### Fat1 detection in the serum of pancreatic cancer patients

**Figure 9:**
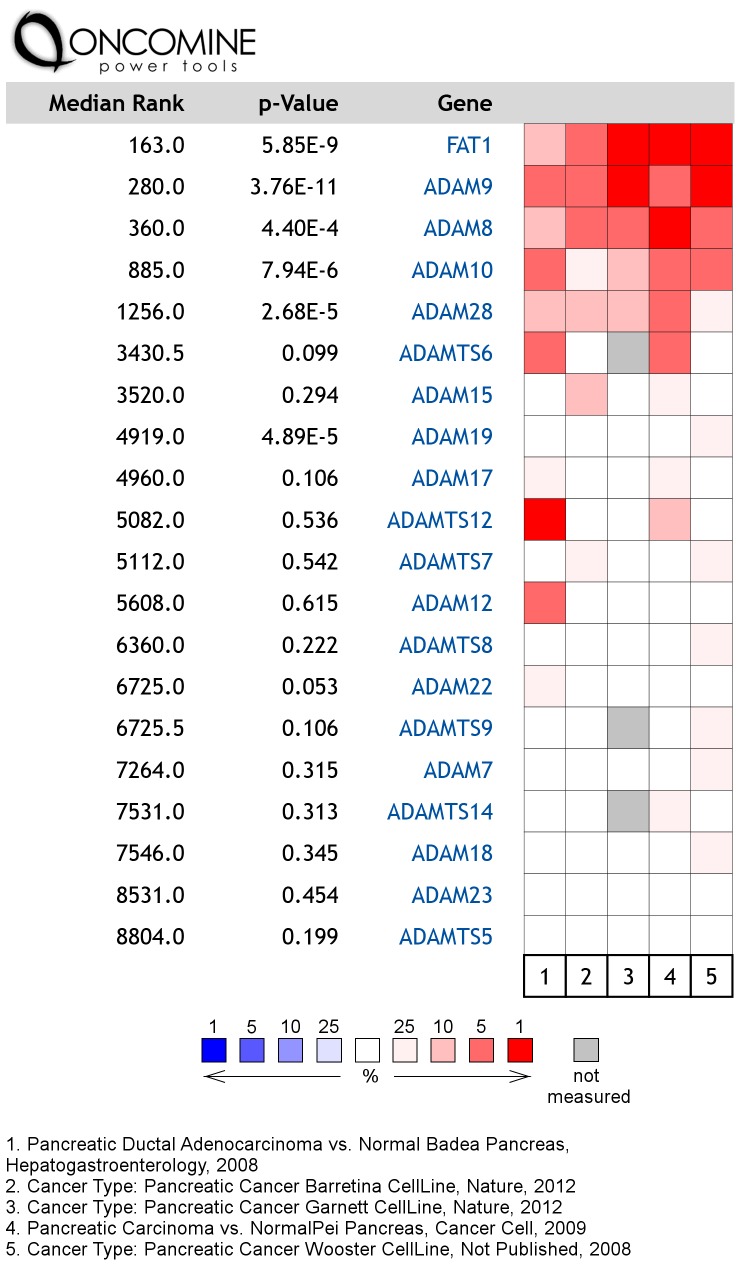
Fat1 and ADAM10 are overexpressed in pancreatic cancers. Oncomine analysis of five mRNA datasets indicate overexpression of Fat1 and of its sheddase ADAM10 in pancreatic cancer. Copyright 2008–11 Compendia Bioscience, Inc. Oncomine (Compendia Bioscience, Ann Arbor, MI) was used for analysis and visualization.

*In silico* analysis of publically available expression array data was used to determine whether Fat cadherins and ADAM10 were also expressed in pancreatic cancer tissues; Oncomine (Compendia Bioscience, Ann Arbor, MI) was used for analysis and visualization. Analysis of five large mRNA-expression datasets showed that Fat1 and ADAM10 mRNAs were significantly overexpressed. Particularly, Fat1 was ranked among the 1% highest overexpressed genes and ADAM10 among the 5% highest overexpressed genes in pancreatic adenocarcinoma in three of five studies, respectively, with overexpression, although less pronounced, also detected in the other two studies (figure 9). At the same time, however, expression levels between individual cases largely varied (figure S4).

**Figure 10:**
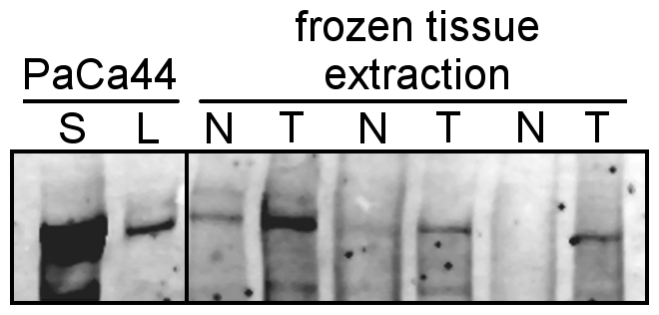
Western blot analysis of exemplary pancreatic cancer and normal tissue pairs confirms Fat1 protein-overexpression. Western blot analysis of Fat1 in extracts from three matched pairs of pancreatic cancer tissues and corresponding normal adjacent pancreas. Extracts were prepared from frozen fresh tissues (75 µg protein analysed); cell lysate (L, 75 µg) and secretome (S, 8 µg) fraction from PaCa44 cells are shown for comparison.

Increased expression levels of Fat1 in pancreatic adenocarcinoma as compared to corresponding normal pancreatic tissue could be confirmed at the protein level by Western blotting (Figure 10). The increased expression of Fat1 in combination with increased levels of the sheddase ADAM10 in clinically relevant cancer patients suggested that soluble Fat1 should be investigated for its utility as a potential serum biomarker. Therefore, we next determined whether the *in vitro* observation of increased Fat1 in secretome also translated into detectable Fat1 protein in the sera of pancreatic cancer patients. Using a centrifugation protocol to enrich for very large proteins and protein complexes, a Fat1 immunoreactive band could be identified in the serum of two out of 11 patients with pancreatic cancer but was absent in all normal control sera examined (n = 10) (figure S5). To corroborate this analysis, corresponding gel slices in the immunoreactive region then underwent tryptic digestion and mass spectrometry analysis. Results compiled in table S3 identified the presence of Fat1 specific peptides thereby confirming the presence of Fat1 extracellular domain present in the serum samples from pancreatic cancer patients.

**Figure 11:**
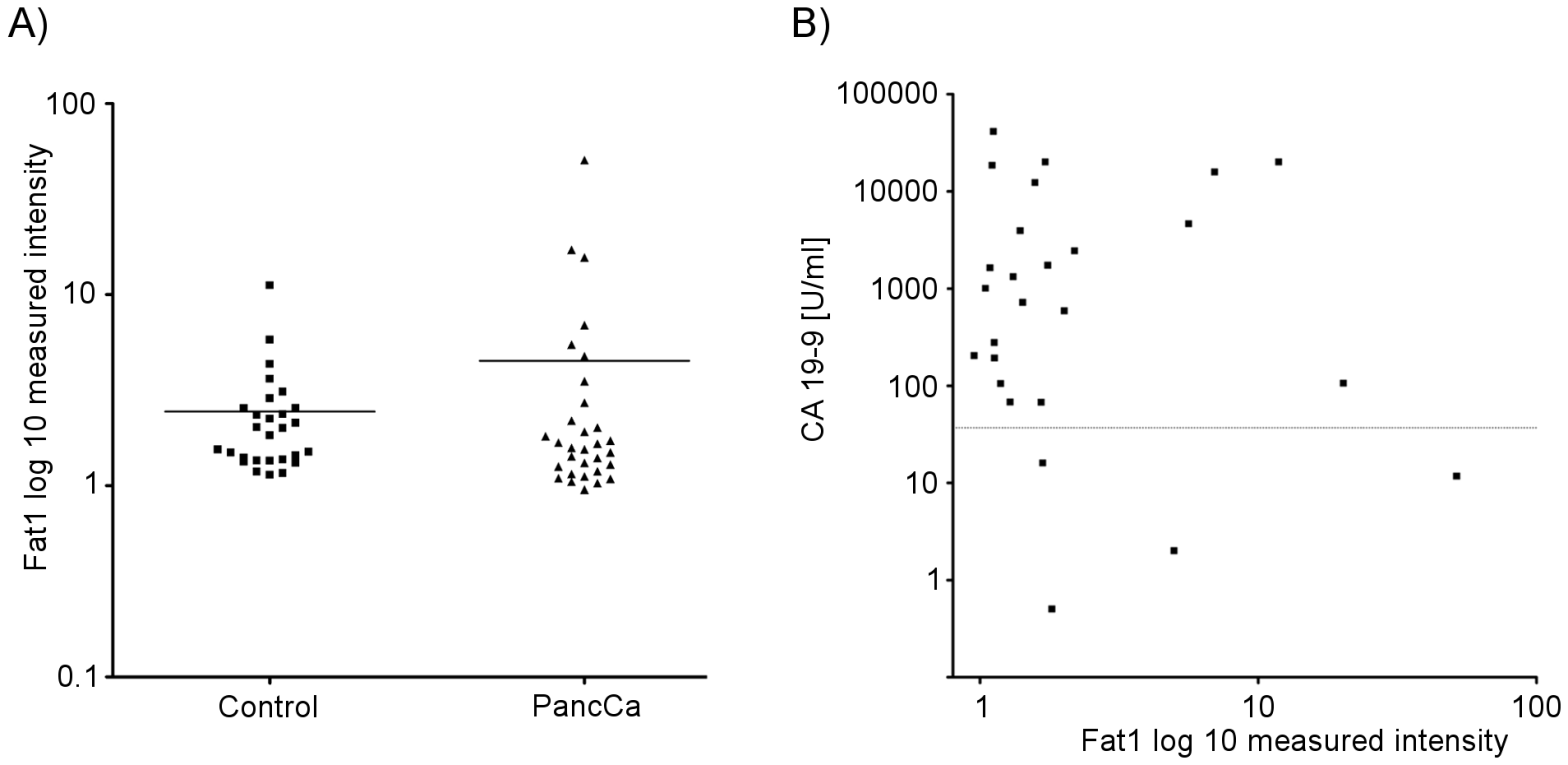
Serum level of soluble Fat1 in patients with pancreatic cancer. A) Levels of soluble Fat1 in serum as determined by ELISA (see Methods). Serum samples from 28 unaffected individuals are compared to the levels of Fat1 in 31 patients with pancreatic cancer (listed in table S4). B) Comparison of circulating Fat1 with CA19-9 levels in patients with pancreatic cancer. Samples are the subset of 26/30 patients with CA19-9 levels available.

To more readily evaluate soluble Fat1 levels in serum samples, a sandwich ELISA assay against the Fat1 extracellular domain was therefore developed as described in the Materials and Methods. The assay was first validated using samples where cells were treated with RNAi against Fat1 (figure S6) and after further optimization, the specificity of the ELISA was determined using the two pancreatic cancer serum samples displayed in figure S5. The soluble Fat1 ELISA assay was then applied to a cohort of 30 serum samples from pancreatic cancer patients (patient data are summarized in table S4) and 28 control samples with results showing that the mean levels of soluble Fat1 are increased in pancreatic cancer compared to the normal controls although the difference does not reach statistical significance in this cohort (p = 0.21) (figure 11A).

We then compared the ability to detect Fat1 in the serum of pancreatic cancer patients to that of the biomarker CA19-9, the only serum marker in clinical use for monitoring pancreatic cancer patients. Naturally, levels of the two markers do not correlate (r = 0.0069; figure 11B) because of the clearly different distributions. However, more importantly, two of the four patients with no or only modest levels of CA19-9 could be identified by high levels of soluble Fat1.

## Discussion

The major goal of our approach in analyzing the secretomes of human cancer cells *in vitro* is to identify potential novel biomarkers that may be also released into the circulation of cancer patients. In addition, identification of the repertoire of proteins that are released into the extracellular compartment can also contribute to our understanding of cell biology and tumor behavior. In our previous analysis of secretomes from colorectal cancer we found that soluble forms of cadherins were among the most abundant proteins [13]. In particular, the 80 kDa soluble E-cadherin in tissue is largely resistant towards further proteolytic processing by endogenous proteases and significant levels of soluble E-cadherin were detected in serum in patients with late stage colorectal cancer [20]. In the present report we describe the release of high levels of the ectodomain of the giant Fat1 cadherin into the secretomes of human pancreatic cancer cells *in vitro*, a process that is also recapitulated in the serum of patients with pancreatic cancer. We also report, that ADAM10 is involved in this ectodomain shedding that also generates a p60 C-terminal remnant fragment either residing in the plasma membrane or being processed further.

Cadherins represent one of the major classes of membrane proteins with the small family of evolutionary conserved Fat cadherins representing their largest members. The archetypal Fat gene was originally identified as a tumor suppressor in Drosophila [30] and is able to suppress growth through regulation of the Hippo pathway, a highly conserved kinase cascade [31], [32]. In the mouse, Fat1 knockout has shown its critical function in the slit diaphragm, a specialized adherens junction that functions in filtration in the kidney and also a role during brain development [33]. The first identified biochemical function of Fat1 involved control of actin dynamics through interactions with Ena/Vasp proteins [34], [35] although the list of interacting proteins and proposed functions has now grown substantially (reviewed in [23]).

Neither Fat1 nor any other mammalian Fat cadherin has previously been investigated in studies of pancreatic cancer or adult pancreatic tissues. The expression of Fat1 had been noted in human fetal pancreas [21] but its expression in the mature human pancreas has not been reported. In rodents, Fat1 expression is prominent during fetal development [36], [37] but its expression has generally been considered low or negligible in adult tissues with few exceptions, particularly brain and certain cells within tissues such as podocytes in the kidney (reviewed in [23]). Early *in silico* analyzes of Fat1 using profiling data have revealed its expression in a range of human cancers, including gastric, pancreatic, colorectal and breast cancers [38] but relatively few studies to date have provided a clear picture of whether Fat1 mRNA and protein are differentially expressed in these cancers compared to their normal tissue counterparts. However in an increasing number of reports involving human cancer Fat1 is noted to be altered. Studies have directly analyzed Fat1 expression and found overexpression in breast cancer [39], melanoma [25] and leukemia [40], suggesting a tumor promoting effect of Fat1. Other studies, however, pointed to a potential tumor suppressor role, as loss of heterozygosity and homozygous deletions were detected in astrocytic cancers and in oral tumors [41], [42], whereas loss of membraneous Fat1 was reported to correlate with more aggressive intrahepatic cholangiocarcinomas [43].

In the present study we found increased expression of Fat1 mRNA in pancreatic cancer over normal controls in five large microarray data sets. Furthermore in all cohorts there was also a significant increase in the levels of the ADAM10 sheddase in pancreatic cancer, an association that may have pathophysiological significance since we demonstrated that this particular metalloprotease was involved in ectodomain shedding of Fat1 *in vitro*. We also confirmed that Fat1 protein levels were similarly increased in pancreatic cancer relative to adjacent normal pancreatic tissue. Therefore the overexpression of Fat1 in concert with the apparent overexpression of the enzyme largely responsible for its cleavage (ADAM10) appears to provide some explanation for the abundance of the Fat1 protein in pancreatic cancer cell secretomes. Quite remarkably we have estimated the amount of Fat1 in the pancreatic cancer secretome to represent at least 1‰ of the total protein content as determined by quantitative MS using the AQUA method [44], [45]. Collectively these results prompted us to further investigate the mechanisms underpinning the release of Fat1 from pancreatic cancer cells and its potential as a serum biomarker of pancreatic cancer.

Fat1 could be detected as a single protein species via Western blotting in both cell culture supernatants and also in patient serum suggesting that the secreted Fat1 protein is relatively stable, an important prerequisite for candidate biomarkers to find applicability in robust assay formats. The large apparent mobility of soluble Fat1 together with the alignment of peptides identified by MS confirmed that the shed product is largely composed of the ECD almost in its entirety. The precise cleavage site(s) facilitating shedding has not been identified but the most distal peptides mapped to regions including the laminin G-like motif, suggesting the proteolytic processing of Fat1 occurs relatively close to the membrane. In addition to the shed ectodomain this process would also produce a membrane bound C-terminal fragment of about 60 kDa that in fact was identified in lysates of the BxPC3 cell line and at lower levels in Paca44 cells. Given the paradigm that ectodomain shedding is typically a two-step process consisting of an extracellular metalloprotease followed by a cleavage within the TM region (reviews [18], [46]) it may be anticipated that the 60 kDa C-terminal fragment would be subject to further proteolysis leading to reduced levels or even absence of this isoform. Indeed, using a chimeric construct in which the extracellular Fat1 sequences were replaced by E-cadherin extracellular domains, Magg *et al.* have demonstrated that γ-secretase, subsequent to ectodomain shedding, cleaved intracellular Fat1 sequences to produce a free C-terminal Fat1 product that was subsequently transported to the cell nucleus and postulated to be involved in transcriptional regulation [47]. However at present no studies have reported on the function of the Fat1 intracellular fragment localized to the cell nucleus. Similarly the biological significance of the shed Fat1 ectodomain is also unknown, but it is plausible to suggest that it may have *de novo* functions acting at distal sites, i.e. by homo- or heterodimerization. Such functions have certainly been demonstrated for other cleaved receptors, for example CD44 (reviewed in [48]) as well as for E-cadherin, whose shed ectodomain can promote carcinogenesis by disrupting cell junctions [49] and through increasing expression of proteases [50]. Nevertheless more work is required to establish what particular functions are imparted by the discrete proteolytic products of Fat1.

Towards investigating the use of Fat1 as a circulating biomarker we developed an ELISA test to measure circulating levels of Fat1. Application of this test against serum samples from control subjects and a preliminary cohort of pancreatic cancer patients found increased levels in some pancreatic cancer patients (6/30 patients). In light of the fact that significant overexpression of Fat1 and of its sheddase ADAM10 appears to be common in human pancreatic cancer it came as a surprise that the subpopulation of patients with increased levels of soluble Fat1 is restricted.

We do not find an association of soluble Fat1 levels with stage of the disease. As expected from the observed proportion of Fat1-postive cases, directly comparing the results of Fat1 measurements with CA19-9 levels showed that more patients have detectable high levels of CA19-9. Thus at face value, measurements of circulating Fat1 are unlikely to supplant CA19-9 measurements. However, of the four patients with no or only modest levels of CA19-9, half of these could be identified to display high circulating levels of soluble Fat1. As described in the Introduction not all patients' tumors express CA19-9 thereby promoting the concept that Fat1 can potentially substitute for CA19-9 in these patients. We therefore suggest our results warrant further validation in larger patient cohorts to better determine how the measurement of circulating Fat1 can be applied as a new biomarker to benefit pancreatic cancer patients, either complementary to CA19-9, or substituting for CA19-9 in monitoring pancreatic patients with the Lewis Lung-negative phenotype.

## Supporting Information

Figure S1 **Fat1 is the major fat cadherin expressed in pancreatic cancer cell lines.** Abundance of mRNA expression levels of each of the four fat genes in five pancreatic cancer cell lines A818-4, BxPc3-B3, MiaPaCa2, Panc1 and PaCa44 as determined by qRT-PCR. Total RNA was extracted using the Illustra RNA minispin kit (GE Healthcare, Sydney, Australia), and reverse transcription was performed on 1 µg of total RNA with random hexamers using BioScript (Bioline, Lonza, Australia) according to the manufacturer's instructions. Quantitative RT-PCR was performed using SensiMix SYBR kit (Bioline, Lonza, Australie) with the specific primers for each gene as shown in the accompanying table withing the figure. The reaction was carried out on 500 ng of total RNA on Applied Biosystems RT-PCR 7500 series system for 40 cycles as follows: 95°C for 15 s followed by 1 min at 60°C. Relative mRNA expression was determined using the ΔΔCt method referenced against GusB, HMBS and RPL19 housekeeping genes.
(TIFF)

Figure S2 **Fat1 protein is post-translationally glycosylated.** Cell lysates (L) and secretome fractions (S) from the pancreatic cancer cell line PaCa44 were subjected to deglycosylation of N- and O-linked carbohydrates as described in the materials and methods S1. The samples were analyzed by Western blotting against Fat1 (ECD1) comparing deglycosylated (+) samples to untreated samples (−) as a control.
(TIFF)

Figure S3 **E-Cadherin ectodomain-shedding is reduced by the chemical inhibitors (A) and by ADAM10 knockdown (B).** A) PaCa44 and Panc1 cells were incubated in serum free medium for two days anpassen wie in der Hauptfigure containing the broad range metalloprotease inhibitor Batimastat (10 µM), the ADAM10-specific inhibitor GI245023X (5 µM) or DMSO as control. After harvesting and concentrating the secretome, 8 µg protein per sample was analyzed for E-Cadherin by Western blot with transferrin used as an internal loading control. The graphs show the normalized results obtained using the Odyssey system (LiCor) to determine relative signal intensities. The results show that both protease inhibitors strongly decrease the levels of sE-Cadherin in PaCa44 but weakly in Panc1. B) Analysis of 8 µg secretome fractions from the indicated cell lines using Western blotting showed significant reductions in E-Cadherin ectodomain shedding. E-Cadherin is a known target of ADAM10 but other proteases may also be involved in E-cadherin shedding [51]. The two experiments provided similar results as indicated by the normalized quantification.
(TIFF)

Figure S4 **Fat1 is overexpressed in pancreatic cancer.** *In silico* analyses of microarray data from two independent studies show that Fat1 mRNA levels are increased in pancreatic cancer as compared to normal pancreatic tissue. Data are presented as a box-whisker plot of log2 data showing the minimum and maximum (dots), 10^th^ and 90^th^ percentiles (whiskers), 25^th^ and 75^th^ percentiles (boxes), and median (bar in boxes). Datasets from A) Badea [30] with 39 matched sets of normal and cancerous tissues and B) Pei et al. [29] comprising 16 cases of normal tissue and 36 cases of pancreatic cancer were analysed using the Oncomine platform (Compendia Bioscience, Ann Arbor, MI).
(TIFF)

Figure S5 **Fat1 ectodomain can be detected in the circulation of pancreatic cancer patients using Western blot analysis.** 2,5 ml serum from patients with pancreatic cancer or unaffected individuals were subjected to a centrifugation protocol to enrich huge proteins as described in the materials and methods S1. Western blot analysis of 25 µg protein sample using the Fat1 ECD2 antibody detected Fat1 in serum samples from cancer patients but not normal controls. The samples shown correspond to the samples with highest values in the ELISA as shown in figure 11.
(TIFF)

Figure S6 **Establishment and validation of the anti-Fat1 ELISA assay.** (A) HMT-3522 T4-2 breast carcinomas were transfected with siRNA duplexes against Fat1 or non-targeting controls at a final concentration of 50 nM as previously described (Sadeqzadeh et al, 2011).The lysates were analyzed with the ELISA 48 h prior to transfection. Complexes revealed after incubation with OPD substrate solution (Sigma) were measured at 495 nm as optical density using a Spectramax 250 plate reader (Molecular Devices)Results present the mean values ± S.E.M. As further controls, omission of the capture antibody in the presence of cell lysate (no primary) or substitution of lysates with lysis buffer (no lysate) were used. .B) A Western blot with 30 µg protein of either FAT1 k/d or control lysate was carried out as control for the ELISA. After incubation with the ECD2 antibody the results were obtained using an ECL-based detection system prior to densitometric analysis. (C) Supernatants of HMT-3522 T4-2 cells cultured in reduced serum media (Opti-MEM) collected one to five days prior to transfection (300 µl/well) were analyzed with the ELISA. (D) Day2 conditioned medium was diluted with PBS and applied to the ELISA as described for (C) using capture with the NTD7 mAb or the CTD7.
(TIFF)

Table S1 **Agilent mRNA Expression profiles of all Fat proteins.** The mRNA of the five pancreatic cancer cell lines A818, BxPc3, MiaPaCa2, Panc1 and PaCa44 and the control cell line HPDE were analyzed, using the Agilent software. A protein is expressed in the cell, when the normalized signal is >5.
(DOC)

Table S2 **Agilent mRNA Expression profiles of all ADAMs.** The mRNA of the five pancreatic cancer cell lines A818, BxPc3, MiaPaCa2, Panc1 and PaCa44 and the control cell line HPDE were analyzed, using the Agilent software. A protein is expressed in the cell, when the normalized signal is >5.
(DOC)

Table S3 **MS-analyses of the high Mr band from enriched serum samples confirm the presence of Fat1.** 2,5 ml serum from patients with pancreatic cancer or control subjects were subjected to the centrifugation protocol to enrich huge proteins as described in the materials and methods S1. 25 µg protein from the pancreatic cancer patient serum were separated using gel electrophoresis and the region corresponding to the Fat1-reactive band was cut out after krypton staining. The gel slices from both patient sera were found to contain Fat1-derived peptides. As a control the secretome from the PaCa44 cell line was analyzed in parallel and demonstrated good recovery of the Fat1 peptides.
(DOC)

Table S4 **Patient data from all ELISA samples.** A) Data from cancer patients including stage, sex, age and serum levels of Fat1 and CA19-9. The CA19-9 data were generated by the medical Clinic Bochum. B) Data from all non-cancer (control) patients collected as per A). Subjects were predominantly tested for cancer but had negative diagnostic findings.
(DOC)

Table S5 **Patient data from all Tissue samples.** Data from pancreatic cancer patients including stage, location of the tumor, sex and age. Normal tissue controls were from the same patients' adjacent healthy tissue.
(DOCX)

File S1 **Mascot search results of the fat1 peptides identified in the secretomes.**
(DOCX)

Materials and Methods S1 (DOCX)
