# Supplementary figures and images for: A Soluble Form of the Giant Cadherin Fat1 Is Released from Pancreatic Cancer Cells by ADAM10 Mediated Ectodomain Shedding
Source: PLoS One. 2014 Mar 13;9(3):e90461. doi: 10.1371/journal.pone.0090461 (PMC3953070; doi:10.1371/journal.pone.0090461)

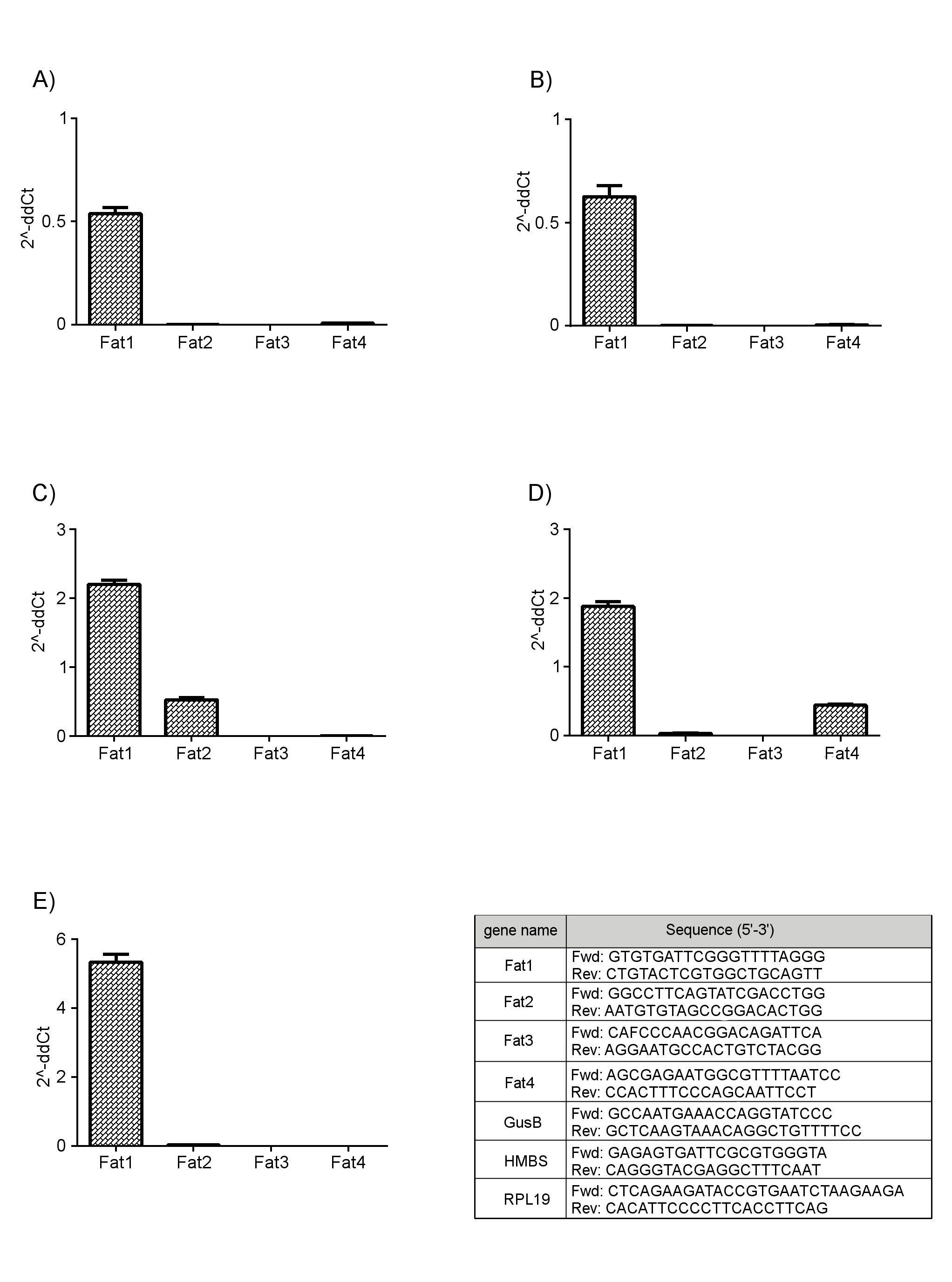

Supplement: Figure S1 — Fat1 is the major fat cadherin expressed in pancreatic cancer cell lines. Abundance of mRNA expression levels of each of the four fat genes in five pancreatic cancer cell lines A818-4, BxPc3-B3, MiaPaCa2, Panc1 and PaCa44 as determined by qRT-PCR. Total RNA was extracted using the Illustra RNA minispin kit (GE Healthcare, Sydney, Australia), and reverse transcription was performed on 1 µg of total RNA with random hexamers using BioScript (Bioline, Lonza, Australia) according to the manufacturer's instructions. Quantitative RT-PCR was performed using SensiMix SYBR kit (Bioline, Lonza, Australie) with the specific primers for each gene as shown in the accompanying table withing the figure. The reaction was carried out on 500 ng of total RNA on Applied Biosystems RT-PCR 7500 series system for 40 cycles as follows: 95°C for 15 s followed by 1 min at 60°C. Relative mRNA expression was determined using the ΔΔCt method referenced against GusB, HMBS and RPL19 housekeeping genes. (TIFF) [file pone.0090461.s001.tiff]

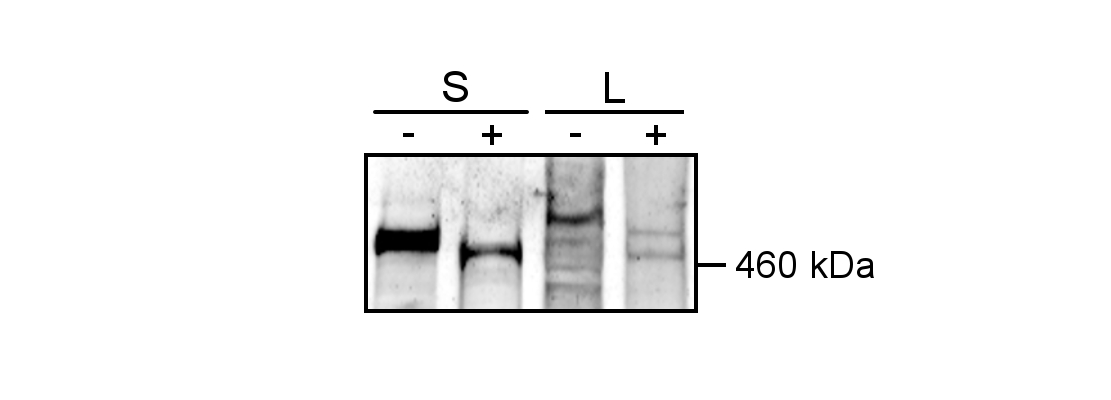

Supplement: Figure S2 — Fat1 protein is post-translationally glycosylated. Cell lysates (L) and secretome fractions (S) from the pancreatic cancer cell line PaCa44 were subjected to deglycosylation of N- and O-linked carbohydrates as described in the materials and methods S1. The samples were analyzed by Western blotting against Fat1 (ECD1) comparing deglycosylated (+) samples to untreated samples (−) as a control. (TIFF) [file pone.0090461.s002.tiff]

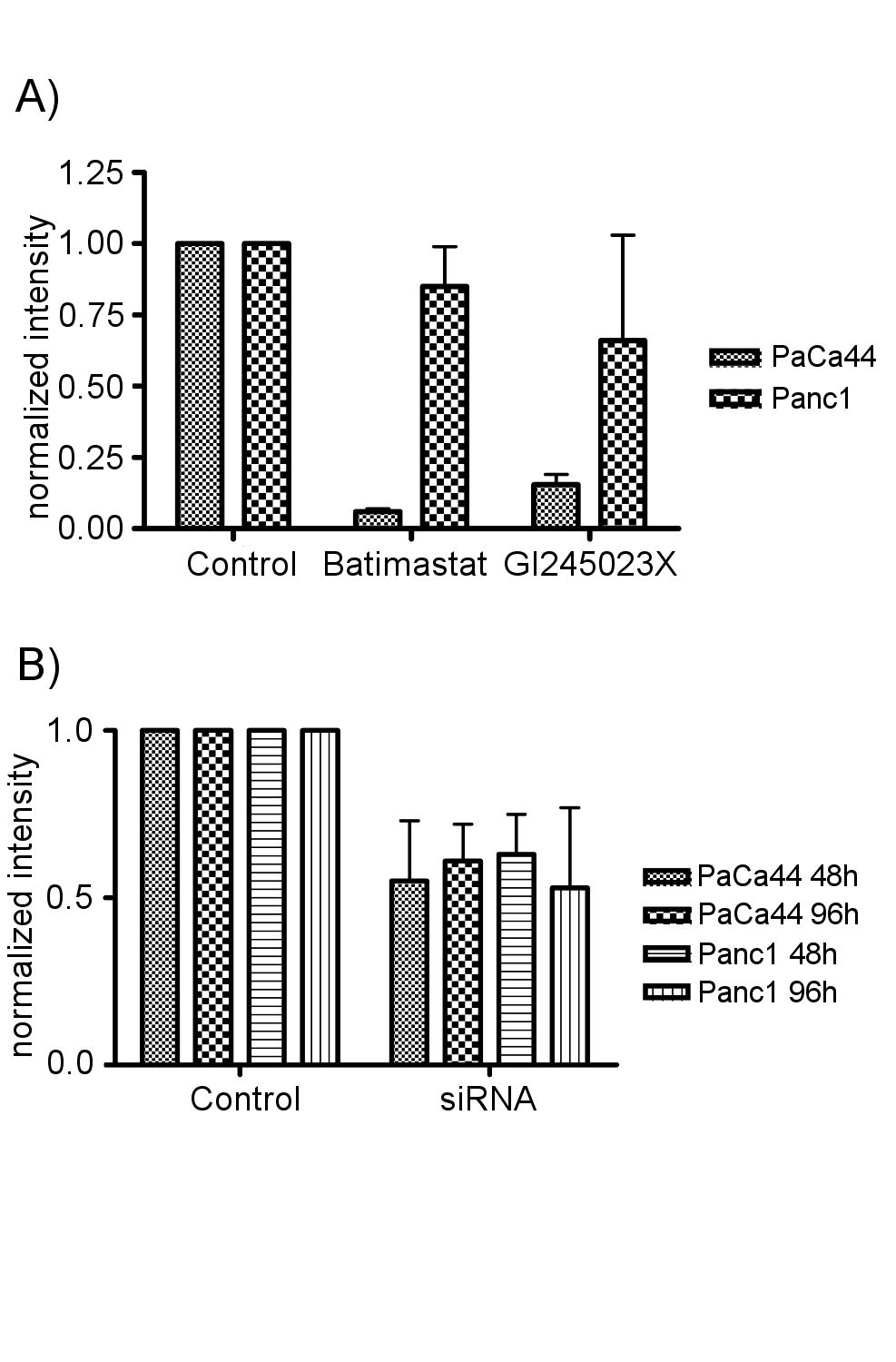

Supplement: Figure S3 — E-Cadherin ectodomain-shedding is reduced by the chemical inhibitors (A) and by ADAM10 knockdown (B). A) PaCa44 and Panc1 cells were incubated in serum free medium for two days anpassen wie in der Hauptfigure containing the broad range metalloprotease inhibitor Batimastat (10 µM), the ADAM10-specific inhibitor GI245023X (5 µM) or DMSO as control. After harvesting and concentrating the secretome, 8 µg protein per sample was analyzed for E-Cadherin by Western blot with transferrin used as an internal loading control. The graphs show the normalized results obtained using the Odyssey system (LiCor) to determine relative signal intensities. The results show that both protease inhibitors strongly decrease the levels of sE-Cadherin in PaCa44 but weakly in Panc1. B) Analysis of 8 µg secretome fractions from the indicated cell lines using Western blotting showed significant reductions in E-Cadherin ectodomain shedding. E-Cadherin is a known target of ADAM10 but other proteases may also be involved in E-cadherin shedding [51]. The two experiments provided similar results as indicated by the normalized quantification. (TIFF) [file pone.0090461.s003.tiff]

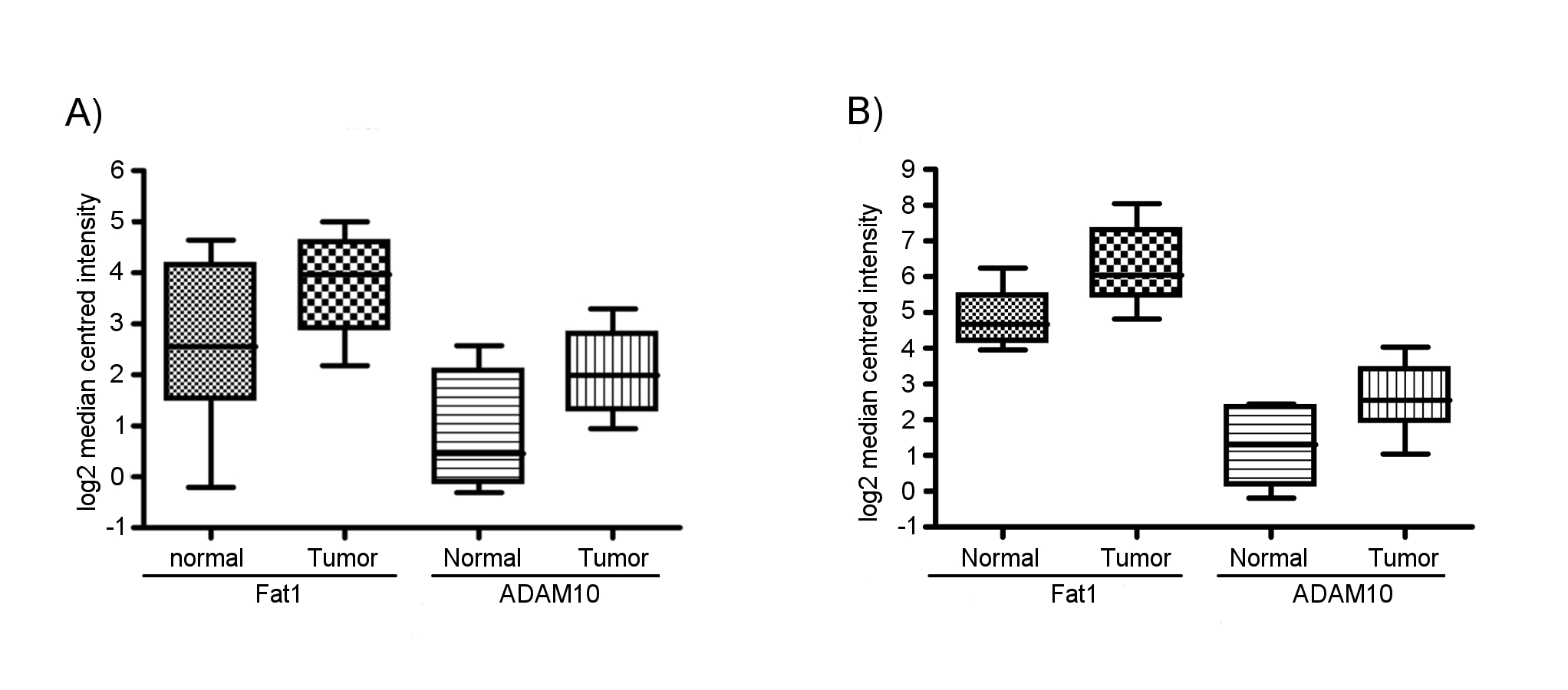

Supplement: Figure S4 — Fat1 is overexpressed in pancreatic cancer. In silico analyses of microarray data from two independent studies show that Fat1 mRNA levels are increased in pancreatic cancer as compared to normal pancreatic tissue. Data are presented as a box-whisker plot of log2 data showing the minimum and maximum (dots), 10th and 90th percentiles (whiskers), 25th and 75th percentiles (boxes), and median (bar in boxes). Datasets from A) Badea [30] with 39 matched sets of normal and cancerous tissues and B) Pei et al. [29] comprising 16 cases of normal tissue and 36 cases of pancreatic cancer were analysed using the Oncomine platform (Compendia Bioscience, Ann Arbor, MI). (TIFF) [file pone.0090461.s004.tiff]

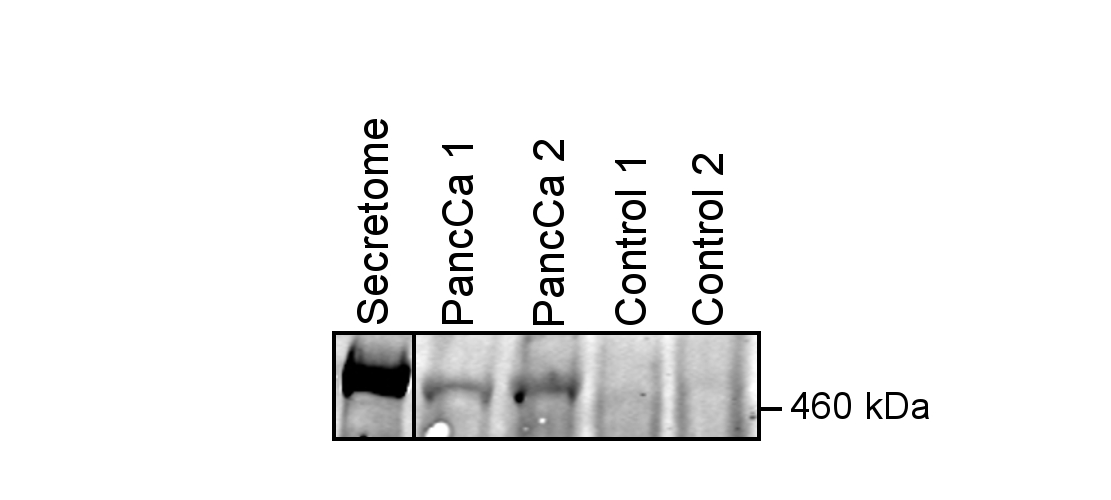

Supplement: Figure S5 — Fat1 ectodomain can be detected in the circulation of pancreatic cancer patients using Western blot analysis. 2,5 ml serum from patients with pancreatic cancer or unaffected individuals were subjected to a centrifugation protocol to enrich huge proteins as described in the materials and methods S1. Western blot analysis of 25 µg protein sample using the Fat1 ECD2 antibody detected Fat1 in serum samples from cancer patients but not normal controls. The samples shown correspond to the samples with highest values in the ELISA as shown in figure 11. (TIFF) [file pone.0090461.s005.tiff]

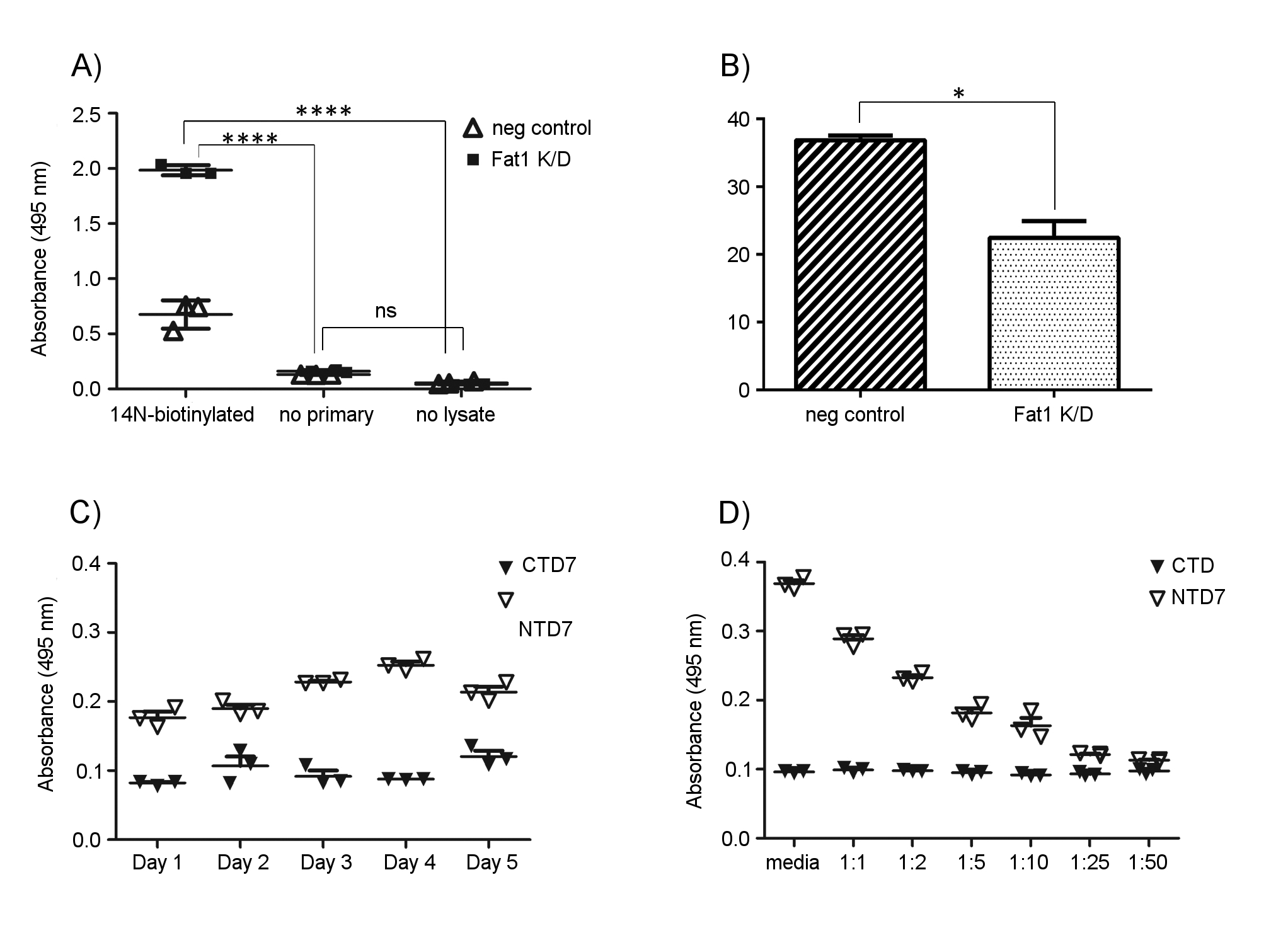

Supplement: Figure S6 — Establishment and validation of the anti-Fat1 ELISA assay. (A) HMT-3522 T4-2 breast carcinomas were transfected with siRNA duplexes against Fat1 or non-targeting controls at a final concentration of 50 nM as previously described (Sadeqzadeh et al, 2011).The lysates were analyzed with the ELISA 48 h prior to transfection. Complexes revealed after incubation with OPD substrate solution (Sigma) were measured at 495 nm as optical density using a Spectramax 250 plate reader (Molecular Devices)Results present the mean values ± S.E.M. As further controls, omission of the capture antibody in the presence of cell lysate (no primary) or substitution of lysates with lysis buffer (no lysate) were used. .B) A Western blot with 30 µg protein of either FAT1 k/d or control lysate was carried out as control for the ELISA. After incubation with the ECD2 antibody the results were obtained using an ECL-based detection system prior to densitometric analysis. (C) Supernatants of HMT-3522 T4-2 cells cultured in reduced serum media (Opti-MEM) collected one to five days prior to transfection (300 µl/well) were analyzed with the ELISA. (D) Day2 conditioned medium was diluted with PBS and applied to the ELISA as described for (C) using capture with the NTD7 mAb or the CTD7. (TIFF) [file pone.0090461.s006.tiff]
