# Supplementary material for: A Soluble Form of the Giant Cadherin Fat1 Is Released from Pancreatic Cancer Cells by ADAM10 Mediated Ectodomain Shedding
Source: PLoS One. 2014 Mar 13;9(3):e90461. doi: 10.1371/journal.pone.0090461 (PMC3953070; doi:10.1371/journal.pone.0090461)
Supplement: Table S1 — Agilent mRNA Expression profiles of all Fat proteins. The mRNA of the five pancreatic cancer cell lines A818, BxPc3, MiaPaCa2, Panc1 and PaCa44 and the control cell line HPDE were analyzed, using the Agilent software. A protein is expressed in the cell, when the normalized signal is >5. (DOC) [file pone.0090461.s007.doc]

**Supplementary Table 1:**

| **Gene symbol** | **HPDE** | **A1814** | **BxPc3** | **Panc1** | **Paca44** | **MiaPaCa2** |
| --- | --- | --- | --- | --- | --- | --- |
| Fat1 | 10.56 | 10.55 | 10.49 | 9.92 | 9.61 | 8.38 |
| Fat2 | 8.50 | 3.56 | 7.23 | 4.07 | 3.97 | 4.24 |
| Fat3 | 2.88 | 2.93 | 2.02 | 2.86 | 2.91 | 3.05 |
| Fat4 | 5.47 | 3.62 | 3.95 | 6.19 | 4.41 | 3.86 |
