# Supplementary material for: A Soluble Form of the Giant Cadherin Fat1 Is Released from Pancreatic Cancer Cells by ADAM10 Mediated Ectodomain Shedding
Source: PLoS One. 2014 Mar 13;9(3):e90461. doi: 10.1371/journal.pone.0090461 (PMC3953070; doi:10.1371/journal.pone.0090461)
Supplement: Table S3 — MS-analyses of the high Mr band from enriched serum samples confirm the presence of Fat1. 2,5 ml serum from patients with pancreatic cancer or control subjects were subjected to the centrifugation protocol to enrich huge proteins as described in the materials and methods S1. 25 µg protein from the pancreatic cancer patient serum were separated using gel electrophoresis and the region corresponding to the Fat1-reactive band was cut out after krypton staining. The gel slices from both patient sera were found to contain Fat1-derived peptides. As a control the secretome from the PaCa44 cell line was analyzed in parallel and demonstrated good recovery of the Fat1 peptides. (DOC) [file pone.0090461.s009.doc]

**Supplementary Table 3**

|  | PaCa44 (Control) | PancCa1 | PancCa2 |
| --- | --- | --- | --- |
| Fat1 peptide matches | 64 | 7 | 3 |
