# Supplementary material for: A Soluble Form of the Giant Cadherin Fat1 Is Released from Pancreatic Cancer Cells by ADAM10 Mediated Ectodomain Shedding
Source: PLoS One. 2014 Mar 13;9(3):e90461. doi: 10.1371/journal.pone.0090461 (PMC3953070; doi:10.1371/journal.pone.0090461)
Supplement: Table S4 — Patient data from all ELISA samples. A) Data from cancer patients including stage, sex, age and serum levels of Fat1 and CA19-9. The CA19-9 data were generated by the medical Clinic Bochum. B) Data from all non-cancer (control) patients collected as per A). Subjects were predominantly tested for cancer but had negative diagnostic findings. (DOC) [file pone.0090461.s010.doc]

a) Cancer patients

| Staging UICC | Fat1 [measured intensity] | Ca19-9 [U/ml] | age | sex |
| --- | --- | --- | --- | --- |
| IB | 0,96 | 202,6 | 66 | F |
| IIA | 1,19 | 104,8 | 46 | F |
| IIA | 1,26 |  | 64 | F |
| IIA | 2,02 | 585 | 62 | M |
| IIA | 1,66 | 67,3 | 76 | F |
| IIB | 50,85 | 11,7 | 68 | M |
| IIB | 4,01 | 2 | 82 | F |
| IIB | 19,14 | 105,6 | 66 | F |
| IIB | 1,05 | 1.000 | 73 | M |
| IIB | 0,98 | 1.977 | 66 | M |
| III | 1,29 |  | 54 | F |
| IIIB | 1,40 | 3.897,00 | 65 | F |
| IV | 5,54 | 4.605,50 | 71 | M |
| IV | 1,09 | 1.627 | 54 | M |
| IV | 11,41 | 20.001 | 61 | F |
| IV | 1,72 | 20.000 | 87 | M |
| IV | 1,43 | 716,7 | 48 | M |
| IV | 1,32 | 1.317 | 39 | F |
| IV | 1,82 | 2,3 | 33 | M |
| IV | 1,69 |  | 60 | M |
| IV | 1,17 | 191,9 | 82 | M |
| IV | 1,29 | 67,5 | 68 | M |
| IV | 1,50 | 1,729 | 76 | M |
| IV | 1,12 | 41.051,00 | 51 | M |
| IV | 2,20 | 2.438 | 23 | F |
| IV | 6,08 | 15.767 | 81 | M |
| IV | 1,11 | 18.378 | 53 | F |
| IV | 1,49 | 16 | 61 | M |
| IV a | 1,58 |  | 80 | M |
| IV | 1,58 | 12.223 | 82 | M |

**b) control subjects**

| Control | Fat1  [measured intensity] | Ca19-9 [U/ml] | age | sex |
| --- | --- | --- | --- | --- |
| C | 2,54 |  | 72 | M |
| C | 2,55 |  | 81 | F |
| C | 5,55 |  | 30 | M |
| C | 1,31 |  | 79 | M |
| C | 1,54 | 0,5 | 43 | M |
| C | 1,27 |  | 33 | M |
| C | 1,24 |  | 67 | M |
| C | 2,02 | 0,6 | 65 | M |
| C | 2,67 |  | 79 | M |
| C | 1,46 |  | 52 | M |
| C | 1,18 |  | 68 | F |
| C | 1,40 |  | 64 | M |
| C | 7,78 | 50,5 | 57 | M |
| C | 1,37 |  | 48 | M |
| C | 1,55 | 4,9 | 52 | M |
| C | 1,16 |  | 83 | F |
| C | 1,83 |  | 17 | F |
| C | 2,30 |  | 75 | F |
| C | 1,67 |  | 37 | F |
| C | 2,14 | 6,7 | 72 | F |
| C | 1,36 |  | 81 | M |
| C | 2,11 |  | 30 | F |
| C | 3,59 |  | 69 | F |
| C | 2,36 |  | 32 | M |
| C | 1,14 |  |  | F |
| C | 1,16 |  |  | M |
