# Supplementary material for: A Soluble Form of the Giant Cadherin Fat1 Is Released from Pancreatic Cancer Cells by ADAM10 Mediated Ectodomain Shedding
Source: PLoS One. 2014 Mar 13;9(3):e90461. doi: 10.1371/journal.pone.0090461 (PMC3953070; doi:10.1371/journal.pone.0090461)
Supplement: Table S5 — Patient data from all Tissue samples. Data from pancreatic cancer patients including stage, location of the tumor, sex and age. Normal tissue controls were from the same patients' adjacent healthy tissue. (DOCX) [file pone.0090461.s011.docx]

| Staging UICC | Localization of tumor | age | sex |
| --- | --- | --- | --- |
| IIB | Head of pancreas | 58 | M |
| IIB | Head of pancreas | 81 | M |
| IIB | Not specified | 46 | M |
