# Supplementary material for: A Soluble Form of the Giant Cadherin Fat1 Is Released from Pancreatic Cancer Cells by ADAM10 Mediated Ectodomain Shedding
Source: PLoS One. 2014 Mar 13;9(3):e90461. doi: 10.1371/journal.pone.0090461 (PMC3953070; doi:10.1371/journal.pone.0090461)
Supplement: File S1 — Mascot search results of the fat1 peptides identified in the secretomes. (DOCX) [file pone.0090461.s012.docx]

**Mascot Search Results**

**(The length of the sequence is due to possible sequence variants)**

**Panc1 A1**

Match to: **IPI00031411** Score: **40**

**Gene_Symbol=FAT1 Protocadherin Fat 1 lng=4591 # SP[4593,D,22,D]SNP[4646,g,1064,R]SNP[4677,r,1064,R]SNP[4713,i,1125,I]SNP[4791,l,1125,I]SNP[4844,i,1252,I]SNP[4856,h,1273,H]SNP[4889,r,1273,H]SNP[4931,l,1283,P]SNP[4969,p,1283,P]SNP[5000,l,129,V]SNP[5**

Found in search of C:\mgf\Orbidata\080911_ISW1295_Panc1_A1.mgf

Nominal mass (M_r_): **1068827**; Calculated pI value: **4.55**

NCBI BLAST search of [IPI00031411](http://www.ncbi.nlm.nih.gov/blast/Blast.cgi?ALIGNMENTS=50&ALIGNMENT_VIEW=Pairwise&AUTO_FORMAT=Semiauto&CDD_SEARCH=on&CLIENT=web&COMPOSITION_BASED_STATISTICS=on&DATABASE=nr&DESCRIPTIONS=100&ENTREZ_QUERY=(none)&EXPECT=10&FILTER=L&FORMAT_BLOCK_ON_RESPAGE=None&FORMAT_OBJECT=Alignment&FORMAT_TYPE=HTML&GAPCOSTS=11+1&I_THRESH=0.001&LAYOUT=TwoWindows&MATRIX_NAME=BLOSUM62&NCBI_GI=on&PAGE=Proteins&PROGRAM=blastp&QUERY=IPI00031411&SERVICE=plain&SET_DEFAULTS.x=21&SET_DEFAULTS.y=7&SHOW_OVERVIEW=on&WORD_SIZE=3&END_OF_HTTPGET=Yes) against nr

Unformatted [sequence string](http://mbp-mascot4/mascot/cgi/getseq.pl?IPI_human+IPI00031411+seq) for pasting into other applications

Fixed modifications: Carbamidomethyl (C)

Variable modifications: Oxidation (M)

Cleavage by TrypsinMSIPI, a mixture of enzymes:

cuts C-term side of KR unless next residue is P

cuts C-term side of J

cuts N-term side of J

Sequence Coverage: **0%**

Matched peptides shown in **Bold Red**

**1** MGRHLALLLL LLLLFQHFGD SDGSQRLEQT PLQFTHLEYN VTVQENSAAK

**51** TYVGHPVKMG VYITHPAWEV RYKIVSGDSE NLFKAEEYIL GDFCFLRIRT

**101** KGGNTAILNR EVKDHYTLIV KALEKNTNVE ARTKVRVQVL DTNDLRPLFS

**151** PTSYSVSLPE NTAIRTSIAR VSATDADIGT NGEFYYSFKD RTDMFAIHPT

**201** SGVIVLTGRL DYLETKLYEM EILAADRGMK LYGSSGISSM AKLTVHIEQA

**251** NECAPVITAV TLSPSELDRD PAYAIVTVDD CDQGANGDIA SLSIVAGDLL

**301** QQFRTVRSFP GSKEYKVKAI GGIDWDSHPF GYNLTLQAKD KGTPPQFSSV

**351** KVIHVTSPQF KAGPVKFEKD VYRAEISEFA PPNTPVVMVK AIPAYSHLRY

**401** VFKSTPGKAK FSLNYNTGLI SILEPVKRQQ AAHFELEVTT SDRKASTKVL

**451** VKVLGANSNP PEFTQTAYKA AFDENVPIGT TVMSLSAVDP DEGENGYVTY

**501** SIANLNHVPF AIDHFTGAVS TSENLDYELM PRVYTLRIRA SDWGLPYRRE

**551** VEVLATITLN NLNDNTPLFE KINCEGTIPR DLGVGEQITT VSAIDADELQ

**601** LVQYQIEAGN ELDFFSLNPN SGVLSLKRSL MDGLGAKVSF HSLRITATDG

**651** ENFATPLYIN ITVAASHKLV NLQCEETGVA KMLAEKLLQA NKLHNQGEVE

**701** DIFFDSHSVN AHIPQFRSTL PTGIQVKENQ PVGSSVIFMN STDLDTGFNG

**751** KLVYAVSGGN EDSCFMIDME TGMLKILSPL DRETTDKYTL NITVYDLGIP

**801** QKAAWRLLHV VVVDANDNPP EFLQESYFVE VSEDKEVHSE IIQVEATDKD

**851** LGPNGHVTYS IVTDTDTFSI DSVTGVVNIA RPLDRELQHE HSLKIEARDQ

**901** AREEPQLFST VVVKVSLEDV NDNPPTFIPP NYRVKVREDL PEGTVIMWLE

**951** AHDPDLGQSG QVRYSLLDHG EGNFDVDKLS GAVRIVQQLD FEKKQVYNLT

**1001** VRAKDKGKPV SLSSTCYVEV EVVDVNENLH PPVFSSFVEK GTVKEDAPVG

**1051** SLVMTVSAHD EDARRDGEIR YSIRDGSGVG VFKIGEETGV IETSDRLDRE

**1101** STSHYWLTVF ATDQGVVPLS SFIEIYIEVE DVNDNAPQTS EPVYYPEIME

**1151** NSPKDVSVVQ IEAFDPDSSS NDKLMYKITS GNPQGFFSIH PKTGLITTTS

**1201** RKLDREQQDE HILEVTVTDN GSPPKSTIAR VIVKILDEND NKPQFLQKFY

**1251** KIRLPEREKP DRERNARREP LYHVIATDKD EGPNAEISYS IEDGNEHGKF

**1301** FIEPKTGVVS SKRFSAAGEY DILSIKAVDN GRPQKSSTTR LHIEWISKPK

**1351** PSLEPISFEE SFFTFTVMES DPVAHMIGVI SVEPPGIPLW FDITGGNYDS

**1401** HFDVDKGTGT IIVAKPLDAE QKSNYNLTVE ATDGTTTILT QVFIKVIDTN

**1451** DHRPQFSTSK YEVVIPEDTA PETEILQISA VDQDEKNKLI YTLQSSRDPL

**1501** SLKKFRLDPA TGSLYTSEKL DHEAVHQHTL TVMVRDQDVP VKRNFARIVV

**1551** NVSDTNDHAP WFTASSYKGR VYESAAVGSV VLQVTALDKD KGKNAEVLYS

**1601** IESGTFGNIG NSFMIDPVLG SIKTAKELDR SNQAEYDLMV KATDKGSPPM

**1651** SEITSVRIFV TIADNASPKF TSKEYSVELS ETVSIGSFVG MVTAHSQSSV

**1701** VYEIKDGNTG DAFDINPHSG TIITQKALDF ETLPIYTLII QGTNMAGLST

**1751** NTTVLVHLQD ENDNAPVFMQ AEYTGLISES ASINSVVLTD RNVPLVIRAA

**1801** DADKDSNALL VYHIVEPSVH TYFAIDSSTG AIHTVLSLDY EETSIFHFTV

**1851** QVHDMGTPRL FAEYAANVTV HVIDINDCPP VFAKPLYEAS LLLPTYKGVK

**1901** VITVNATDAD SSAFSQLIYS ITEGNIGEKF SMDYKTGALT VQNTTQLRSR

**1951** YELTVRASDG RFAGLTSVKI NVKESKESHL K**FTQDVYSAV VK**ENSTEAET

**2001** LAVITAIGNP INEPLFYHIL NPDRRFKISR TSGVLSTTGT PFDREQQEAF

**2051** DVVVEVTEEH KPSAVAHVVV KVIVEDQNDN APVFVNLPYY AVVKVDTEVG

**2101** HVIRYVTAVD RDSGRNGEVH YYLKEHHEHF QIGPLGEISL KKQFELDTLN

**2151** KEYLVTVVAK DGGNPAFSAE VIVPITVMNK AMPVFEKPFY SAEIAESIQV

**2201** HSPVVHVQAN SPEGLKVFYS ITDGDPFSQF TINFNTGVIN VIAPLDFEAH

**2251** PAYKLSIRAT DSLTGAHAEV FVDIIVDDIN DNPPVFAQQS YAVTLSEASV

**2301** IGTSVVQVRA TDSDSEPNRG ISYQMFGNHS KSHDHFHVDS STGLISLLRT

**2351** LDYEQSRQHT IFVRAVDGGM PTLSSDVIVT VDVTDLNDNP PLFEQQIYEA

**2401** RISEHAPHGH FVTCVKAYDA DSSDIDKLQY SILSGNDHKH FVIDSATGII

**2451** TLSNLHRHAL KPFYSLNLSV SDGVFRSSTQ VHVTVIGGNL HSPAFLQNEY

**2501** EVELAENAPL HTLVMEVKTT DGDSGIYGHV TYHIVNDFAK DRFYINERGQ

**2551** IFTLEKLDRE TPAEKVISVR LMAKDAGGKV AFCTVNVILT DDNDNAPQFR

**2601** ATKYEVNIGS SAAKGTSVVK VLASDADEGS NADITYAIEA DSESVKENLE

**2651** INKLSGVITT KESLIGLENE FFTFFVRAVD NGSPSKESVV LVYVKILPPE

**2701** MQLPKFSEPF YTFTVSEDVP IGTEIDLIRA EHSGTVLYSL VKGNTPESNR

**2751** DESFVIDRQS GRLKLEKSLD HETTKWYQFS ILARCTQDDH EMVASVDVSI

**2801** QVKDANDNSP VFESSPYEAF IVENLPGGSR VIQIRASDAD SGTNGQVMYS

**2851** LDQSQSVEVI ESFAINMETG WITTLKELDH EKRDNYQIKV VASDHGEKIQ

**2901** LSSTAIVDVT VTDVNDSPPR FTAEIYKGTV SEDDPQGGVI AILSTTDADS

**2951** EEINRQVTYF ITGGDPLGQF AVETIQNEWK VYVKKPLDRE KRDNYLLTIT

**3001** ATDGTFSSKA IVEVKVLDAN DNSPVCEKTL YSDTIPEDVL PGKLIMQISA

**3051** TDADIRSNAE ITYTLLGSGA EKFKLNPDTG ELKTSTPLDR EEQAVYHLLV

**3101** RATDGGGRFC QASIVLTLED VNDNAPEFSA DPYAITVFEN TEPGTLLTRV

**3151** QATDADAGLN RKILYSLIDS ADGQFSINEL SGIIQLEKPL DRELQAVYTL

**3201** SLKAVDQGLP RRLTATGTVI VSVLDINDNP PVFEYREYGA TVSEDILVGT

**3251** EVLQVYAASR DIEANAEITY SIISGNEHGK FSIDSKTGAV FIIENLDYES

**3301** SHEYYLTVEA TDGGTPSLSD VATVNVNVTD INDNTPVFSQ DTYTTVISED

**3351** AVLEQSVITV MADDADGPSN SHIHYSIIDG NQGSSFTIDP VRGEVKVTKL

**3401** LDRETISGYT LTVQASDNGS PPRVNTTTVN IDVSDVNDNA PVFSRGNYSV

**3451** IIQENKPVGF SVLQLVVTDE DSSHNGPPFF FTIVTGNDEK AFEVNPQGVL

**3501** LTSSAIKRKE KDHYLLQVKV ADNGKPQLSS LTYIDIRVIE ESIYPPAILP

**3551** LEIFITSSGE EYSGGVIGKI HATDQDVYDT LTYSLDPQMD NLFSVSSTGG

**3601** KLIAHKKLDI GQYLLNVSVT DGKFTTVADI TVHIRQVTQE MLNHTIAIRF

**3651** ANLTPEEFVG DYWRNFQRAL RNILGVRRND IQIVSLQSSE PHPHLDVLLF

**3701** VEKPGSAQIS TKQLLHKINS SVTDIEEIIG VRILNVFQKL CAGLDCPWKF

**3751** CDEKVSVDES VMSTHSTARL SFVTPRHHRA AVCLCKEGRC PPVHHGCEDD

**3801** PCPEGSECVS DPWEEKHTCV CPSGRFGQCP GSSSMTLTGN SYVKYRLTEN

**3851** ENKLEMKLTM RLRTYSTHAV VMYARGTDYS ILEIHHGRLQ YKFDCGSGPG

**3901** IVSVQSIQVN DGQWHAVALE VNGNYARLVL DQVHTASGTA PGTLKTLNLD

**3951** NYVFFGGHIR QQGTRHGRSP QVGNGFRGCM DSIYLNGQEL PLNSKPRSYA

**4001** HIEESVDVSP GCFLTATEDC ASNPCQNGGV CNPSPAGGYY CKCSALYIGT

**4051** HCEISVNPCS SKPCLYGGTC VVDNGGFVCQ CRGLYTGQRC QLSPYCKDEP

**4101** CKNGGTCFDS LDGAVCQCDS GFRGERCQSD IDECSGNPCL HGALCENTHG

**4151** SYHCNCSHEY RGRHCEDAAP NQYVSTPWNI GLAEGIGIVV FVAGIFLLVV

**4201** VFVLCRKMIS RKKKHQAEPK DKHLGPATAF LQRPYFDSKL NKNIYSDIPP

**4251** QVPVRPISYT PSIPSDSRNN LDRNSFEGSA IPEHPEFSTF NPESVHGHRK

**4301** AVAVCSVAPN LPPPPPSNSP SDSDSIQKPS WDFDYDTKVV DLDPCLSKKP

**4351** LEEKPSQPYS ARESLSEVQS LSSFQSESCD DNGYHWDTSD WMPSVPLPDI

**4401** QEFPNYEVID EQTPLYSADP NAIDTDYYPG GYDIESDFPP PPEDFPAADE

**4451** LPPLPPEFSN QFESIHPPRD MPAAGSLGSS SRNRQRFNLN QYLPNFYPLD

**4501** MSEPQTKGTG ENSTCREPHA PYPPGYQRHF EAPAVESMPM SVYASTASCS

**4551** DVSACCEVES EVMMSDYESG DDGHFEEVTI PPLDSQQHTE VJDGSQRLEQ

**4601** TPLQFTHLEY NVTVQENSAA KJGTVKEDAP VGSLVMTVSA HDEDAGRDGE

**4651** IRJGTVKEDA PVGSLVMTVS AHDEDARRDG EIRJLDREST SHYWLTVFAT

**4701** DQGVVPLSSF IEIYIEVEDV NDNAPQTSEP VYYPEIMENS PKDVSVVQIE

**4751** AFDPDSSSND KJLDRESTSH YWLTVFATDQ GVVPLSSFIE LYIEVEDVND

**4801** NAPQTSEPVY YPEIMENSPK DVSVVQIEAF DPDSSSNDKJ FYKIRLPERJ

**4851** REPLYHVIAT DKDEGPNAEI SYSIEDGNEH GKJREPLYRV IATDKDEGPN

**4901** AEISYSIEDG NEHGKJEPLY HVIATDKDEG LNAEISYSIE DGNEHGKFFI

**4951** EPKJEPLYHV IATDKDEGPN AEISYSIEDG NEHGKFFIEP KJALEKNTNL

**5001** EARTKJALEK NTNVEARTKJ ALEKNTNVEA RTKJALEKNT NVEVRTKJFS

**5051** AAGEYDILSI KAVDNGRPQK SSTTRJFSAA GEYDILSIKA VDSGRPQKSS

**5101** TTRJNFARIV VNVSDTNDHA PWFTASSYKG RJNFARIVVN VSDTNDHAPW

**5151** FTTSSYKGRJ GKNAEVLYSI ESGDFGNIGN SFMIDPVLGS IKTAKJGKNA

**5201** EVLYSIESGN FGNIGNSFMI DPVLGSIKTA KJGKNAEVLY SIESGTIGNI

**5251** GNSFMIDPVL GSIKTAKJGS PPMSEITSVR IFVTNADNAS PKFTSKJGSP

**5301** PMSEITSVRI FVTSADNASP KFTSKJGSPP MSEITSVRIF VTIADNASTK

**5351** FTSKJALDFE TLPIYTLIIQ GTNMAGLSTN TTVLVHLQDE NDNAPVFMQA

**5401** EYTGLISESA SINSVVLTDR NVPQVIRAAD ADKJALDFET LPIYTLIIQG

**5451** TNMAGLSTNT TVLVHLQDEN DNAPVFMQAE YTGLISESAS INSVVLTDRN

**5501** VPRVIRAADA DKJAADADKD SNVLLVYHIV EPSVHTYFAI DSSTGAIHTV

**5551** LSLDYEETSI FHFTVQVHDM GTPRLFAEYA ANVTVHVIDI NDCPPVFAKP

**5601** LYEASLLLPT YKJFTQDVYS AVVKTNSTEA ETLAVITAIG NPINEPLFYH

**5651** ILNPDRRJTS GVLSTTGTPF DREAQEAFDV VVEVTEEHKP SAVAHVVVKV

**5701** IVEDQNDNAP VFVNLPYYAV VKJTDMFAIH PTSGVIVLTG RLDFLETKLY

**5751** EMEILAADRJ TDMFAIHPTS GVIVLTGRLD YLETKLYEME ILAADRJNGE

**5801** VHYYLKEHHE HFQIGPLGEK SLKKJDGGNP AFSAEVIVPI TVMNKAMPVF

**5851** EKPFYSAEIA ESIQVHSHVV HVQANSPEGL KVFYSITDGD PFSQFTINFN

**5901** TGVINVIAPL DFEAHPAYKJ AMPVFEKPFY SAEIAESIQV HSPVVHVQAN

**5951** SPEGLKVFYS ITDGDPFSQF TINFNTGVIN VIAPLDFPAH PAYKLSIRJL

**6001** SIRATDSLTG AHAEVFVDDI VDDINDNPPV FAQQSYAVTL SEASVIGTSV

**6051** VQVRATDSDS EPNRJLSIRA TDSLTGAHAE VFVDEIVDDI NDNPPVFAQQ

**6101** SYAVTLSEAS VIGTSVVQVR ATDSDSEPNR JTLDYEQSRQ HTIAVRAVDG

**6151** GMPTLSSDVI VTVDVTDLND NPPLFEQQIY EARJLYGSSG ISSMAKLTVH

**6201** IEQANECAPV ITAVTLSPSE LDRDPAYAIV TVDDCDQGAN GDIASLSIVA

**6251** GDLLQQFRJI LPPEMQLPKF SEPFYTFTVS EIVPIGTEID LIRAEHSGTV

**6301** LYSLVKJILP PEMQLPKFSE PFYTFTVSEV VPIGTEIDLI RAEHSGTVLY

**6351** SLVKJCTQDD HEMVASVDVS IQVKDANDNS PVFESSPYEA FIVENLPGGS

**6401** RVIQIRJCTQ DDHEMVASVD VSIQVKDASD NSPVFESSPY EAFIVENLPG

**6451** GSRVIQIRJV IQIRASDADN GTNGQVMYSL DQSQSVEVIE SFAINMETGW

**6501** ITTLKELDHE KJFTAEIYKG TVSELDPQGG VIAILSTTDA DSEEINRQVT

**6551** YFITGGDPLG QFAVETIQNE WKJFTAEIYK GTVSEPDPQG GVIAILSTTD

**6601** ADSEEINRQV TYFITGGDPL GQFAVETIQN EWKJFTAEIY KGTVSEQDPQ

**6651** GGVIAILSTT DADSEEINRQ VTYFITGGDP LGQFAVETIQ NEWKJGTVSE

**6701** DDPQGGVIAI LSTTDADSEE INRQVTYGIT GGDPLGQFAV ETIQNEWKVY

**6751** VKJGTVSEDD PQGGVIAILS TTDADSEEIN RQVTYSITGG DPLGQFAVET

**6801** IQNEWKVYVK JGTVSEDDPQ GGVIAILSTT DADSEEINRQ VTYFITGGDP

**6851** LAQFAVETIQ NEWKVYVKJA IVEVKVLDAN DNSPVCEYTL YSDTIPEDVL

**6901** PGKLIMQISA TDADIRJVLD ANDNSPVCEK TLYIDTIPED VLPGKLIMQI

**6951** SATDADIRJV LDANDNSPVC EKTLYTDTIP EDVLPGKLIM QISATDADIR

**7001** JATDGGGRFC QASIVLDLED VNDNAPEFSA DPYAITVFEN TEPGTLLTRV

**7051** QATDADAGLN RJKILYSLID SADGQFSINE LIGIIQLEKP LDRELQAVYT

**7101** LSLKJFSIDS KTGAVFIIEN LDYESSHEYY LTVEATDGGT PSLSDVATVN

**7151** VNVTDINDAT PVFSQDTYTT VISEDAVLEQ SVITVMADDA DGPSNSHIHY

**7201** SIIDGNQGSS FTIDPVRGEV KJFSIDSKTG AVFIIENLDY ESSHEYYLTV

**7251** EATDGGTPSL SDVATVNVNV TDINDVTPVF SQDTYTTVIS EDAVLEQSVI

**7301** TVMADDADGP SNSHIHYSII DGNQGSSFTI DPVRGEVKJV NTTTVNIDVS

**7351** DVNDNAPVFS RGNYSVIIQE NKPVGFSVLQ LVVTDEDSSH NGPPFFFTIV

**7401** TENDEKAFEV NPQGVLLTSS AIKJVNTTTV NIDVSDVNDN APVFSRGNYS

**7451** VIIQENKPVG FSVLQLVVTD EDSSHNGPPF FFTIVTGNDE KAFEVNPQGV

**7501** LLTSSAIKJG NYSVIIQENK PVGFSVLQLV VTDEDSSHNG PPFFFTIVTG

**7551** NDEKAFEVNA QGVLLTSSAI KRJGNYSVII QENKPVGFSV LQLVVTDEDS

**7601** SHNGPPFFFT IVTGNDEKAF EVNVQGVLLT SSAIKRJVAD NGKPQLSSLT

**7651** YIDIRVIEES IYPPAILPLE IAITSSGEEY SGGVIGKIHA TDQDVYDTLT

**7701** YSLDPQMDNL FSVSSTGGKJ VADNGKPQLS SLTYIDIRVI EESIYPPAIL

**7751** PLEISITSSG EEYSGGVIGK IHATDQDVYD TLTYSLDPQM DNLFSVSSTG

**7801** GKJVIHVTSP QFKAGPVKFE KJQLLHKIMS SVTDIEEIIG VRILNVFQKJ

**7851** QLLHKIVSSV TDIEEIIGVR ILNVFQKJEG RCPPVHHGCE DHPCPEGSEC

**7901** VSDPWEEKHT CVCPSGRJEG RCPPVHHGCE DPPCPEGSEC VSDPWEEKHT

**7951** CVCPSGRJHT CVCPSGRFGG CPGSSSMTLT GNSYVKYRJH TCVCPSGRFG

**8001** RCPGSSSMTL TGNSYVKYRJ FGQCPGSSSM TLTGNSYVKT RLTENENKJG

**8051** CMDSIYLNGQ ELPLNSKPRS YAHIEESVPV SPGCFLTATE DCASNPCQNG

**8101** GVCNPSPAGG YYCKCSALYI GTHCEISVNP CSSKPCLYGG TCVVDNGGFV

**8151** CQCRJYVFKR TPGKAKJYVF KSTPGKAKJS YAHIEESVDV SPGCFLTATE

**8201** DCASNPCQNG GVCNPSPAGG YYCKCSALYI GTHCEISVNP KSSKPCLYGG

**8251** TCVVDNGGFV CQCRGLYTGQ RJSYAHIEES VDVSPGCFLT ATEDCASNPC

**8301** QNGGVCNPSP AGGYYCKCSA LYIGTHCEIS VNPNSSKPCL YGGTCVVDNG

**8351** GFVCQCRGLY TGQRJDEPCK NGGTCFDSAD GAVCQCDSGF RGERJGRHCA

**8401** DAAPNQYVST PWNIGLAEGI GIVVFVAGIF LLVVVFVLCR KJGRHCEDAA

**8451** PNQYVSTPWN IGLAEGIGIV VFVAGIFLLV VVFVLCRKJN IYSDIPPQVP

**8501** VRYISYTPSI PSDSRNNLDR JKAVAVCSVA PNLPPPPPSN SPSDSDSIQK

**8551** PSWDFDYDTK VVDLDPCLSK JKPLEEKPSQ PYSARESLSS VQSLSSFQSE

**8601** SCDDNGYHWD TSDWMPSVPL PDIQEFPNYE VIDEQTPLYS ADPNAIDTDY

**8651** YPGGYDIESD FPPPPEDFPA ADELPPLPPE FSNQFESIHP PRDMPAAGSL

**8701** GSSSRJKPLE EKPSQPYSAR ESLSEVQSLS SFQSESCDDN GYHWDTSDWM

**8751** PSVPLQDIQE FPNYEVIDEQ TPLYSADPNA IDTDYYPGGY DIESDFPPPP

**8801** EDFPAADELP PLPPEFSNQF ESIHPPRDMP AAGSLGSSSR JKPLEEKPSQ

**8851** PYSARESLSE VQSLSSFQSE SCDDNGYHWD TSDWMPSVPL PDIQEFPNYE

**8901** VIDPQTPLYS ADPNAIDTDY YPGGYDIESD FPPPPEDFPA ADELPPLPPE

**8951** FSNQFESIHP PRDMPAAGSL GSSSRJESLS EVQSLSSFQS ESCDDNGYHW

**9001** DTSDWMPSVP LPDIQEFPNY EVIDEQTPLY SADPNAIDTD YYPGGYDIES

**9051** DFPPPPEDFP AADELPPLPP EFSNQFESIH PPRDMPAAGS LGSSRRNRJE

**9101** SLSEVQSLSS FQSESCDDNG YHWDTSDWMP SVPLPDIQEF PNYEVIDEQT

**9151** PLYSADPNAI DTDYYPGGYD IESDFPPPPE DFPAADELPP LPPEFSNQFE

**9201** SIHPPRDMPA AGSLGSSWRN RJVLGANSNP PEFTQTAYKA AFDENVPIGT

**9251** TIMSLSAVDP DEGENGYVTY SIANLNHVPF AIDHFTGAVS TSENLDYELM

**9301** PRVYTLRJVL GANSNPPEFT QTAYKAAFDE NVPIGTTVMS LSAVDPDEGE

**9351** NGYVTYSIAN LNHVPFAIDH FTGAVSTSEN LDYELMPRVY TLRJVLGANS

**9401** NPPEFTQTAY KAAFDENVPI GTTVMSLSAV DPDEGENGYV TYSIANLNHV

**9451** PFAIDHFTGA VSTSENLDYE LMPRVYTLRJ INCEGTIPRD LGVGEQITTV

**9501** SAIDADELQL VQYQIEAGNE LDFFSLNPNS GVLSLKRJIN CEGTIPRDLG

**9551** VGEQITTVSA IDADELQLVQ YQIEAGNELD LFSLNPNSGV LSLKRJINCE

**9601** GTIPRDLGVG EQITTVSAID ADELQLVQYQ IEAGNELDFF SLNPNSGVLS

**9651** LKRJEVHSEI IQVEATDKDL GPNGHVTYSI LTDTDTFSID SVTGVVNIAR

**9701** PLDRELQHEH SLKJEVHSEI IQVEATDKDL GPNGHVTYSI VTDTDTFSID

**9751** SVTGVVNIAR PLDRELQHEH SLK

**Start - End Observed Mr(expt) Mr(calc) ppm Miss Sequence**

**1982 - 1992 628.8301 1255.6457 1255.6449 1 0 K.FTQDVYSAVVK.E**  ([Ions score 40](http://mbp-mascot4/mascot/cgi/peptide_view.pl?file=../data/20091021/F006296.dat&query=1533&hit=1&index=IPI00031411&px=1&section=5&ave_thresh=22))

Formularbeginn

**Panc1 A2:**

Match to: **IPI00031411** Score: **6815**

**Gene_Symbol=FAT1 Protocadherin Fat 1 lng=4591 # SP[4593,D,22,D]SNP[4646,g,1064,R]SNP[4677,r,1064,R]SNP[4713,i,1125,I]SNP[4791,l,1125,I]SNP[4844,i,1252,I]SNP[4856,h,1273,H]SNP[4889,r,1273,H]SNP[4931,l,1283,P]SNP[4969,p,1283,P]SNP[5000,l,129,V]SNP[5**

Found in search of C:\mgf\Orbidata\080911_ISW1295_**Panc1_A2**.mgf

Nominal mass (M_r_): **1068827**; Calculated pI value: **4.55**

NCBI BLAST search of [IPI00031411](http://www.ncbi.nlm.nih.gov/blast/Blast.cgi?ALIGNMENTS=50&ALIGNMENT_VIEW=Pairwise&AUTO_FORMAT=Semiauto&CDD_SEARCH=on&CLIENT=web&COMPOSITION_BASED_STATISTICS=on&DATABASE=nr&DESCRIPTIONS=100&ENTREZ_QUERY=(none)&EXPECT=10&FILTER=L&FORMAT_BLOCK_ON_RESPAGE=None&FORMAT_OBJECT=Alignment&FORMAT_TYPE=HTML&GAPCOSTS=11+1&I_THRESH=0.001&LAYOUT=TwoWindows&MATRIX_NAME=BLOSUM62&NCBI_GI=on&PAGE=Proteins&PROGRAM=blastp&QUERY=IPI00031411&SERVICE=plain&SET_DEFAULTS.x=21&SET_DEFAULTS.y=7&SHOW_OVERVIEW=on&WORD_SIZE=3&END_OF_HTTPGET=Yes) against nr

Unformatted [sequence string](http://mbp-mascot4/mascot/cgi/getseq.pl?IPI_human+IPI00031411+seq) for pasting into other applications

Fixed modifications: Carbamidomethyl (C)

Variable modifications: Oxidation (M)

Cleavage by TrypsinMSIPI, a mixture of enzymes:

cuts C-term side of KR unless next residue is P

cuts C-term side of J

cuts N-term side of J

Sequence Coverage: **13%**

Matched peptides shown in **Bold Red**

**1** MGRHLALLLL LLLLFQHFGD SDGSQRLEQT PLQFTHLEYN VTVQENSAAK

**51** TYVGHPVK**MG VYITHPAWEV R**YK**IVSGDSE NLFKAEEYIL GDFCFLR**IRT

**101** KGGNTAILNR **EVKDHYTLIV K**ALEKNTNVE ARTKVR**VQVL DTNDLRPLFS**

**151 PTSYSVSLPE NTAIR**TSIAR VSATDADIGT NGEFYYSFK**D RTDMFAIHPT**

**201 SGVIVLTGR**L DYLETK**LYEM EILAADR**GMK LYGSSGISSM AK**LTVHIEQA**

**251 NECAPVITAV TLSPSELDR**D PAYAIVTVDD CDQGANGDIA SLSIVAGDLL

**301** QQFRTVRSFP GSKEYKVKAI GGIDWDSHPF GYNLTLQAKD KGTPPQFSSV

**351** K**VIHVTSPQF K**AGPVKFEKD VYR**AEISEFA PPNTPVVMVK** AIPAYSHLRY

**401** VFKSTPGKAK **FSLNYNTGLI SILEPVKR**QQ AAHFELEVTT SDRKASTKVL

**451** VK**VLGANSNP PEFTQTAYK**A AFDENVPIGT TVMSLSAVDP DEGENGYVTY

**501** SIANLNHVPF AIDHFTGAVS TSENLDYELM PRVYTLRIR**A SDWGLPYR**RE

**551** VEVLATITLN NLNDNTPLFE KINCEGTIPR DLGVGEQITT VSAIDADELQ

**601** LVQYQIEAGN ELDFFSLNPN SGVLSLKRSL MDGLGAKVSF HSLRITATDG

**651** ENFATPLYIN ITVAASHK**LV NLQCEETGVA K**MLAEKLLQA NK**LHNQGEVE**

**701 DIFFDSHSVN AHIPQFRSTL PTGIQVK**ENQ PVGSSVIFMN STDLDTGFNG

**751** KLVYAVSGGN EDSCFMIDME TGMLKILSPL DRETTDKYTL NITVYDLGIP

**801** QKAAWRLLHV VVVDANDNPP EFLQESYFVE VSEDKEVHSE IIQVEATDKD

**851** LGPNGHVTYS IVTDTDTFSI DSVTGVVNIA RPLDRELQHE HSLKIEAR**DQ**

**901 AREEPQLFST VVVKVSLEDV NDNPPTFIPP NYR**VK**VREDL PEGTVIMWLE**

**951 AHDPDLGQSG QVRYSLLDHG EGNFDVDKLS GAVRIVQQLD FEK**KQVYNLT

**1001** VRAKDKGKPV SLSSTCYVEV EVVDVNENLH PPVFSSFVEK GTVKEDAPVG

**1051** SLVMTVSAHD EDARRDGEIR YSIRDGSGVG VFK**IGEETGV IETSDRLDR**E

**1101** STSHYWLTVF ATDQGVVPLS SFIEIYIEVE DVNDNAPQTS EPVYYPEIME

**1151** NSPK**DVSVVQ IEAFDPDSSS NDK**LMYK**ITS GNPQGFFSIH PK**TGLITTTS

**1201** RKLDR**EQQDE HILEVTVTDN GSPPK**STIAR VIVK**ILDEND NKPQFLQK**FY

**1251** KIRLPEREKP DRERNARREP LYHVIATDKD EGPNAEISYS IEDGNEHGK**F**

**1301 FIEPK**TGVVS SKR**FSAAGEY DILSIK**AVDN GRPQKSSTTR LHIEWISKPK

**1351** PSLEPISFEE SFFTFTVMES DPVAHMIGVI SVEPPGIPLW FDITGGNYDS

**1401** HFDVDK**GTGT IIVAKPLDAE QK**SNYNLTVE ATDGTTTILT QVFIKVIDTN

**1451** DHRPQFSTSK **YEVVIPEDTA PETEILQISA VDQDEKNKLI YTLQSSR**DPL

**1501** SLKK**FRLDPA TGSLYTSEKL DHEAVHQHTL TVMVR**DQDVP VKRNFARIVV

**1551** NVSDTNDHAP WFTASSYKGR VYESAAVGSV VLQVTALDKD KGKNAEVLYS

**1601** IESGTFGNIG NSFMIDPVLG SIKTAKELDR **SNQAEYDLMV K**ATDK**GSPPM**

**1651 SEITSVRIFV TIADNASPK**F TSKEYSVELS ETVSIGSFVG MVTAHSQSSV

**1701** VYEIK**DGNTG DAFDINPHSG TIITQK**ALDF ETLPIYTLII QGTNMAGLST

**1751** NTTVLVHLQD ENDNAPVFMQ AEYTGLISES ASINSVVLTD RNVPLVIRAA

**1801** DADKDSNALL VYHIVEPSVH TYFAIDSSTG AIHTVLSLDY EETSIFHFTV

**1851** QVHDMGTPRL FAEYAANVTV HVIDINDCPP VFAKPLYEAS LLLPTYKGVK

**1901** VITVNATDAD SSAFSQLIYS ITEGNIGEKF SMDYKTGALT VQNTTQLRSR

**1951** YELTVRASDG RFAGLTSVKI NVKESKESHL K**FTQDVYSAV VK**ENSTEAET

**2001** LAVITAIGNP INEPLFYHIL NPDRRFKISR **TSGVLSTTGT PFDR**EQQEAF

**2051** DVVVEVTEEH KPSAVAHVVV K**VIVEDQNDN APVFVNLPYY AVVK**VDTEVG

**2101** HVIRYVTAVD RDSGRNGEVH YYLK**EHHEHF QIGPLGEISL KKQFELDTLN**

**2151 KEYLVTVVAK DGGNPAFSAE VIVPITVMNK** AMPVFEKPFY SAEIAESIQV

**2201** HSPVVHVQAN SPEGLKVFYS ITDGDPFSQF TINFNTGVIN VIAPLDFEAH

**2251** PAYKLSIRAT DSLTGAHAEV FVDIIVDDIN DNPPVFAQQS YAVTLSEASV

**2301** IGTSVVQVRA TDSDSEPNRG ISYQMFGNHS K**SHDHFHVDS STGLISLLR**T

**2351** LDYEQSRQHT IFVRAVDGGM PTLSSDVIVT VDVTDLNDNP PLFEQQIYEA

**2401** R**ISEHAPHGH FVTCVKAYDA DSSDIDKLQY SILSGNDHKH FVIDSATGII**

**2451 TLSNLHRHAL KPFYSLNLSV SDGVFR**SSTQ VHVTVIGGNL HSPAFLQNEY

**2501** EVELAENAPL HTLVMEVKTT DGDSGIYGHV TYHIVNDFAK DRFYINERGQ

**2551** IFTLEKLDRE TPAEKVISVR LMAKDAGGK**V AFCTVNVILT DDNDNAPQFR**

**2601** ATK**YEVNIGS SAAK**GTSVVK **VLASDADEGS NADITYAIEA DSESVKENLE**

**2651 INK**LSGVITT KESLIGLENE FFTFFVRAVD NGSPSK**ESVV LVYVK**ILPPE

**2701** MQLPKFSEPF YTFTVSEDVP IGTEIDLIR**A EHSGTVLYSL VKGNTPESNR**

**2751 DESFVIDR**QS GRLKLEKSLD HETTK**WYQFS ILAR**CTQDDH EMVASVDVSI

**2801** QVK**DANDNSP VFESSPYEAF IVENLPGGSR** VIQIRASDAD SGTNGQVMYS

**2851** LDQSQSVEVI ESFAINMETG WITTLKELDH EKRDNYQIKV VASDHGEKIQ

**2901** LSSTAIVDVT VTDVNDSPPR FTAEIYKGTV SEDDPQGGVI AILSTTDADS

**2951** EEINRQVTYF ITGGDPLGQF AVETIQNEWK VYVKKPLDRE KR**DNYLLTIT**

**3001 ATDGTFSSK**A IVEVK**VLDAN DNSPVCEKTL YSDTIPEDVL PGKLIMQISA**

**3051 TDADIRSNAE ITYTLLGSGA EK**FKLNPDTG ELK**TSTPLDR EEQAVYHLLV**

**3101 R**ATDGGGRFC QASIVLTLED VNDNAPEFSA DPYAITVFEN TEPGTLLTRV

**3151** QATDADAGLN RKILYSLIDS ADGQFSINEL SGIIQLEKPL DRELQAVYTL

**3201** SLKAVDQGLP RRLTATGTVI VSVLDINDNP PVFEYREYGA TVSEDILVGT

**3251** EVLQVYAASR **DIEANAEITY SIISGNEHGK** FSIDSKTGAV FIIENLDYES

**3301** SHEYYLTVEA TDGGTPSLSD VATVNVNVTD INDNTPVFSQ DTYTTVISED

**3351** AVLEQSVITV MADDADGPSN SHIHYSIIDG NQGSSFTIDP VRGEVKVTKL

**3401** LDRETISGYT LTVQASDNGS PPRVNTTTVN IDVSDVNDNA PVFSRGNYSV

**3451** IIQENKPVGF SVLQLVVTDE DSSHNGPPFF FTIVTGNDEK **AFEVNPQGVL**

**3501 LTSSAIK**RKE KDHYLLQVK**V ADNGKPQLSS LTYIDIR**VIE ESIYPPAILP

**3551** LEIFITSSGE EYSGGVIGK**I HATDQDVYDT LTYSLDPQMD NLFSVSSTGG**

**3601 K**LIAHKKLDI GQYLLNVSVT DGKFTTVADI TVHIRQVTQE MLNHTIAIR**F**

**3651 ANLTPEEFVG DYWR**NFQRAL RNILGVRRND IQIVSLQSSE PHPHLDVLLF

**3701** VEKPGSAQIS TKQLLHKINS SVTDIEEIIG VR**ILNVFQKL CAGLDCPWK**F

**3751** CDEK**VSVDES VMSTHSTARL SFVTPR**HHRA AVCLCKEGRC PPVHHGCEDD

**3801** PCPEGSECVS DPWEEKHTCV CPSGR**FGQCP GSSSMTLTGN SYVK**YRLTEN

**3851** ENKLEMKLTM RLRTYSTHAV VMYAR**GTDYS ILEIHHGR**LQ YKFDCGSGPG

**3901** IVSVQSIQVN DGQWHAVALE VNGNYAR**LVL DQVHTASGTA PGTLKTLNLD**

**3951 NYVFFGGHIR** QQGTRHGRSP QVGNGFR**GCM DSIYLNGQEL PLNSKPR**SYA

**4001** HIEESVDVSP GCFLTATEDC ASNPCQNGGV CNPSPAGGYY CKCSALYIGT

**4051** HCEISVNPCS SKPCLYGGTC VVDNGGFVCQ CRGLYTGQRC QLSPYCKDEP

**4101** CKNGGTCFDS LDGAVCQCDS GFRGERCQSD IDECSGNPCL HGALCENTHG

**4151** SYHCNCSHEY RGRHCEDAAP NQYVSTPWNI GLAEGIGIVV FVAGIFLLVV

**4201** VFVLCRKMIS RKKKHQAEPK DKHLGPATAF LQRPYFDSKL NKNIYSDIPP

**4251** QVPVRPISYT PSIPSDSRNN LDRNSFEGSA IPEHPEFSTF NPESVHGHRK

**4301** AVAVCSVAPN LPPPPPSNSP SDSDSIQKPS WDFDYDTKVV DLDPCLSKKP

**4351** LEEKPSQPYS ARESLSEVQS LSSFQSESCD DNGYHWDTSD WMPSVPLPDI

**4401** QEFPNYEVID EQTPLYSADP NAIDTDYYPG GYDIESDFPP PPEDFPAADE

**4451** LPPLPPEFSN QFESIHPPRD MPAAGSLGSS SRNRQRFNLN QYLPNFYPLD

**4501** MSEPQTKGTG ENSTCREPHA PYPPGYQRHF EAPAVESMPM SVYASTASCS

**4551** DVSACCEVES EVMMSDYESG DDGHFEEVTI PPLDSQQHTE VJDGSQRLEQ

**4601** TPLQFTHLEY NVTVQENSAA KJGTVK**EDAP VGSLVMTVSA HDEDAGR**DGE

**4651** IRJGTVKEDA PVGSLVMTVS AHDEDARRDG EIRJLDREST SHYWLTVFAT

**4701** DQGVVPLSSF IEIYIEVEDV NDNAPQTSEP VYYPEIMENS PKDVSVVQIE

**4751** AFDPDSSSND KJLDRESTSH YWLTVFATDQ GVVPLSSFIE LYIEVEDVND

**4801** NAPQTSEPVY YPEIMENSPK DVSVVQIEAF DPDSSSNDKJ FYKIRLPERJ

**4851** REPLYHVIAT DKDEGPNAEI SYSIEDGNEH GKJREPLYR**V IATDKDEGPN**

**4901 AEISYSIEDG NEHGK**JEPLY HVIATDKDEG LNAEISYSIE DGNEHGKFFI

**4951** EPKJEPLYHV IATDKDEGPN AEISYSIEDG NEHGKFFIEP KJALEKNTNL

**5001** EARTKJALEK NTNVEARTKJ ALEKNTNVEA RTKJALEKNT NVEVRTKJFS

**5051** AAGEYDILSI KAVDNGRPQK SSTTRJFSAA GEYDILSIKA VDSGRPQKSS

**5101** TTRJNFARIV VNVSDTNDHA PWFTASSYKG RJNFARIVVN VSDTNDHAPW

**5151** FTTSSYKGRJ GKNAEVLYSI ESGDFGNIGN SFMIDPVLGS IKTAKJGKNA

**5201** EVLYSIESGN FGNIGNSFMI DPVLGSIKTA KJGKNAEVLY SIESGTIGNI

**5251** GNSFMIDPVL GSIKTAKJGS PPMSEITSVR IFVTNADNAS PKFTSKJGSP

**5301** PMSEITSVRI FVTSADNASP KFTSKJGSPP MSEITSVRIF VTIADNASTK

**5351** FTSKJALDFE TLPIYTLIIQ GTNMAGLSTN TTVLVHLQDE NDNAPVFMQA

**5401** EYTGLISESA SINSVVLTDR NVPQVIRAAD ADKJALDFET LPIYTLIIQG

**5451** TNMAGLSTNT TVLVHLQDEN DNAPVFMQAE YTGLISESAS INSVVLTDRN

**5501** VPRVIRAADA DKJAADADKD SNVLLVYHIV EPSVHTYFAI DSSTGAIHTV

**5551** LSLDYEETSI FHFTVQVHDM GTPRLFAEYA ANVTVHVIDI NDCPPVFAKP

**5601** LYEASLLLPT YKJFTQDVYS AVVKTNSTEA ETLAVITAIG NPINEPLFYH

**5651** ILNPDRRJTS GVLSTTGTPF DREAQEAFDV VVEVTEEHKP SAVAHVVVKV

**5701** IVEDQNDNAP VFVNLPYYAV VKJTDMFAIH PTSGVIVLTG RLDFLETKLY

**5751** EMEILAADRJ TDMFAIHPTS GVIVLTGRLD YLETKLYEME ILAADRJNGE

**5801** VHYYLKEHHE HFQIGPLGEK SLKKJDGGNP AFSAEVIVPI TVMNKAMPVF

**5851** EKPFYSAEIA ESIQVHSHVV HVQANSPEGL KVFYSITDGD PFSQFTINFN

**5901** TGVINVIAPL DFEAHPAYKJ AMPVFEKPFY SAEIAESIQV HSPVVHVQAN

**5951** SPEGLKVFYS ITDGDPFSQF TINFNTGVIN VIAPLDFPAH PAYKLSIRJL

**6001** SIRATDSLTG AHAEVFVDDI VDDINDNPPV FAQQSYAVTL SEASVIGTSV

**6051** VQVRATDSDS EPNRJLSIRA TDSLTGAHAE VFVDEIVDDI NDNPPVFAQQ

**6101** SYAVTLSEAS VIGTSVVQVR ATDSDSEPNR JTLDYEQSRQ HTIAVRAVDG

**6151** GMPTLSSDVI VTVDVTDLND NPPLFEQQIY EARJLYGSSG ISSMAKLTVH

**6201** IEQANECAPV ITAVTLSPSE LDRDPAYAIV TVDDCDQGAN GDIASLSIVA

**6251** GDLLQQFRJI LPPEMQLPKF SEPFYTFTVS EIVPIGTEID LIRAEHSGTV

**6301** LYSLVKJILP PEMQLPKFSE PFYTFTVSEV VPIGTEIDLI RAEHSGTVLY

**6351** SLVKJCTQDD HEMVASVDVS IQVKDANDNS PVFESSPYEA FIVENLPGGS

**6401** RVIQIRJCTQ DDHEMVASVD VSIQVKDASD NSPVFESSPY EAFIVENLPG

**6451** GSRVIQIRJV IQIRASDADN GTNGQVMYSL DQSQSVEVIE SFAINMETGW

**6501** ITTLKELDHE KJFTAEIYKG TVSELDPQGG VIAILSTTDA DSEEINRQVT

**6551** YFITGGDPLG QFAVETIQNE WKJFTAEIYK GTVSEPDPQG GVIAILSTTD

**6601** ADSEEINRQV TYFITGGDPL GQFAVETIQN EWKJFTAEIY KGTVSEQDPQ

**6651** GGVIAILSTT DADSEEINRQ VTYFITGGDP LGQFAVETIQ NEWKJGTVSE

**6701** DDPQGGVIAI LSTTDADSEE INRQVTYGIT GGDPLGQFAV ETIQNEWKVY

**6751** VKJGTVSEDD PQGGVIAILS TTDADSEEIN RQVTYSITGG DPLGQFAVET

**6801** IQNEWKVYVK JGTVSEDDPQ GGVIAILSTT DADSEEINRQ VTYFITGGDP

**6851** LAQFAVETIQ NEWKVYVKJA IVEVKVLDAN DNSPVCEYTL YSDTIPEDVL

**6901** PGKLIMQISA TDADIRJVLD ANDNSPVCEK TLYIDTIPED VLPGKLIMQI

**6951** SATDADIRJV LDANDNSPVC EKTLYTDTIP EDVLPGKLIM QISATDADIR

**7001** JATDGGGRFC QASIVLDLED VNDNAPEFSA DPYAITVFEN TEPGTLLTRV

**7051** QATDADAGLN RJKILYSLID SADGQFSINE LIGIIQLEKP LDRELQAVYT

**7101** LSLKJFSIDS KTGAVFIIEN LDYESSHEYY LTVEATDGGT PSLSDVATVN

**7151** VNVTDINDAT PVFSQDTYTT VISEDAVLEQ SVITVMADDA DGPSNSHIHY

**7201** SIIDGNQGSS FTIDPVRGEV KJFSIDSKTG AVFIIENLDY ESSHEYYLTV

**7251** EATDGGTPSL SDVATVNVNV TDINDVTPVF SQDTYTTVIS EDAVLEQSVI

**7301** TVMADDADGP SNSHIHYSII DGNQGSSFTI DPVRGEVKJV NTTTVNIDVS

**7351** DVNDNAPVFS RGNYSVIIQE NKPVGFSVLQ LVVTDEDSSH NGPPFFFTIV

**7401** TENDEKAFEV NPQGVLLTSS AIKJVNTTTV NIDVSDVNDN APVFSRGNYS

**7451** VIIQENKPVG FSVLQLVVTD EDSSHNGPPF FFTIVTGNDE KAFEVNPQGV

**7501** LLTSSAIKJG NYSVIIQENK PVGFSVLQLV VTDEDSSHNG PPFFFTIVTG

**7551** NDEKAFEVNA QGVLLTSSAI KRJGNYSVII QENKPVGFSV LQLVVTDEDS

**7601** SHNGPPFFFT IVTGNDEKAF EVNVQGVLLT SSAIKRJVAD NGKPQLSSLT

**7651** YIDIRVIEES IYPPAILPLE IAITSSGEEY SGGVIGKIHA TDQDVYDTLT

**7701** YSLDPQMDNL FSVSSTGGKJ VADNGKPQLS SLTYIDIRVI EESIYPPAIL

**7751** PLEISITSSG EEYSGGVIGK IHATDQDVYD TLTYSLDPQM DNLFSVSSTG

**7801** GKJVIHVTSP QFKAGPVKFE KJQLLHKIMS SVTDIEEIIG VRILNVFQKJ

**7851** QLLHKIVSSV TDIEEIIGVR ILNVFQKJEG RCPPVHHGCE DHPCPEGSEC

**7901** VSDPWEEKHT CVCPSGRJEG RCPPVHHGCE DPPCPEGSEC VSDPWEEKHT

**7951** CVCPSGRJHT CVCPSGRFGG CPGSSSMTLT GNSYVKYRJH TCVCPSGRFG

**8001** RCPGSSSMTL TGNSYVKYRJ FGQCPGSSSM TLTGNSYVKT RLTENENKJG

**8051** CMDSIYLNGQ ELPLNSKPRS YAHIEESVPV SPGCFLTATE DCASNPCQNG

**8101** GVCNPSPAGG YYCKCSALYI GTHCEISVNP CSSKPCLYGG TCVVDNGGFV

**8151** CQCRJYVFKR TPGKAKJYVF KSTPGKAKJS YAHIEESVDV SPGCFLTATE

**8201** DCASNPCQNG GVCNPSPAGG YYCKCSALYI GTHCEISVNP KSSKPCLYGG

**8251** TCVVDNGGFV CQCRGLYTGQ RJSYAHIEES VDVSPGCFLT ATEDCASNPC

**8301** QNGGVCNPSP AGGYYCKCSA LYIGTHCEIS VNPNSSKPCL YGGTCVVDNG

**8351** GFVCQCRGLY TGQRJDEPCK NGGTCFDSAD GAVCQCDSGF RGERJGRHCA

**8401** DAAPNQYVST PWNIGLAEGI GIVVFVAGIF LLVVVFVLCR KJGRHCEDAA

**8451** PNQYVSTPWN IGLAEGIGIV VFVAGIFLLV VVFVLCRKJN IYSDIPPQVP

**8501** VRYISYTPSI PSDSRNNLDR JKAVAVCSVA PNLPPPPPSN SPSDSDSIQK

**8551** PSWDFDYDTK VVDLDPCLSK JKPLEEKPSQ PYSARESLSS VQSLSSFQSE

**8601** SCDDNGYHWD TSDWMPSVPL PDIQEFPNYE VIDEQTPLYS ADPNAIDTDY

**8651** YPGGYDIESD FPPPPEDFPA ADELPPLPPE FSNQFESIHP PRDMPAAGSL

**8701** GSSSRJKPLE EKPSQPYSAR ESLSEVQSLS SFQSESCDDN GYHWDTSDWM

**8751** PSVPLQDIQE FPNYEVIDEQ TPLYSADPNA IDTDYYPGGY DIESDFPPPP

**8801** EDFPAADELP PLPPEFSNQF ESIHPPRDMP AAGSLGSSSR JKPLEEKPSQ

**8851** PYSARESLSE VQSLSSFQSE SCDDNGYHWD TSDWMPSVPL PDIQEFPNYE

**8901** VIDPQTPLYS ADPNAIDTDY YPGGYDIESD FPPPPEDFPA ADELPPLPPE

**8951** FSNQFESIHP PRDMPAAGSL GSSSRJESLS EVQSLSSFQS ESCDDNGYHW

**9001** DTSDWMPSVP LPDIQEFPNY EVIDEQTPLY SADPNAIDTD YYPGGYDIES

**9051** DFPPPPEDFP AADELPPLPP EFSNQFESIH PPRDMPAAGS LGSSRRNRJE

**9101** SLSEVQSLSS FQSESCDDNG YHWDTSDWMP SVPLPDIQEF PNYEVIDEQT

**9151** PLYSADPNAI DTDYYPGGYD IESDFPPPPE DFPAADELPP LPPEFSNQFE

**9201** SIHPPRDMPA AGSLGSSWRN RJVLGANSNP PEFTQTAYKA AFDENVPIGT

**9251** TIMSLSAVDP DEGENGYVTY SIANLNHVPF AIDHFTGAVS TSENLDYELM

**9301** PRVYTLRJVL GANSNPPEFT QTAYKAAFDE NVPIGTTVMS LSAVDPDEGE

**9351** NGYVTYSIAN LNHVPFAIDH FTGAVSTSEN LDYELMPRVY TLRJVLGANS

**9401** NPPEFTQTAY KAAFDENVPI GTTVMSLSAV DPDEGENGYV TYSIANLNHV

**9451** PFAIDHFTGA VSTSENLDYE LMPRVYTLRJ INCEGTIPRD LGVGEQITTV

**9501** SAIDADELQL VQYQIEAGNE LDFFSLNPNS GVLSLKRJIN CEGTIPRDLG

**9551** VGEQITTVSA IDADELQLVQ YQIEAGNELD LFSLNPNSGV LSLKRJINCE

**9601** GTIPRDLGVG EQITTVSAID ADELQLVQYQ IEAGNELDFF SLNPNSGVLS

**9651** LKRJEVHSEI IQVEATDKDL GPNGHVTYSI LTDTDTFSID SVTGVVNIAR

**9701** PLDRELQHEH SLKJEVHSEI IQVEATDKDL GPNGHVTYSI VTDTDTFSID

**9751** SVTGVVNIAR PLDRELQHEH SLK

**Start - End Observed Mr(expt) Mr(calc) ppm Miss Sequence**

**59 - 71 520.2664 1557.7774 1557.7762 1 0 K.MGVYITHPAWEVR.Y**  ([Ions score 32](http://mbp-mascot4/mascot/cgi/peptide_view.pl?file=../data/20091021/F006297.dat&query=1930&hit=1&index=IPI00031411&px=1&section=5&ave_thresh=22))

**59 - 71 520.2670 1557.7791 1557.7762 2 0 K.MGVYITHPAWEVR.Y**  ([Ions score 33](http://mbp-mascot4/mascot/cgi/peptide_view.pl?file=../data/20091021/F006297.dat&query=1931&hit=1&index=IPI00031411&px=1&section=5&ave_thresh=22))

**59 - 71 525.5978 1573.7717 1573.7711 0 0 K.MGVYITHPAWEVR.Y**  Oxidation (M) ([Ions score 40](http://mbp-mascot4/mascot/cgi/peptide_view.pl?file=../data/20091021/F006297.dat&query=1963&hit=1&index=IPI00031411&px=1&section=5&ave_thresh=22))

**59 - 71 525.5981 1573.7724 1573.7711 1 0 K.MGVYITHPAWEVR.Y**  Oxidation (M) ([Ions score 35](http://mbp-mascot4/mascot/cgi/peptide_view.pl?file=../data/20091021/F006297.dat&query=1964&hit=1&index=IPI00031411&px=1&section=5&ave_thresh=22))

**74 - 84 604.8119 1207.6092 1207.6085 1 0 K.IVSGDSENLFK.A**  ([Ions score 43](http://mbp-mascot4/mascot/cgi/peptide_view.pl?file=../data/20091021/F006297.dat&query=1137&hit=1&index=IPI00031411&px=1&section=5&ave_thresh=22))

**74 - 84 604.8120 1207.6095 1207.6085 1 0 K.IVSGDSENLFK.A**  ([Ions score 47](http://mbp-mascot4/mascot/cgi/peptide_view.pl?file=../data/20091021/F006297.dat&query=1138&hit=1&index=IPI00031411&px=1&section=5&ave_thresh=22))

**85 - 97 816.8901 1631.7656 1631.7654 0 0 K.AEEYILGDFCFLR.I**  ([Ions score 90](http://mbp-mascot4/mascot/cgi/peptide_view.pl?file=../data/20091021/F006297.dat&query=2071&hit=1&index=IPI00031411&px=1&section=5&ave_thresh=22))

**85 - 97 816.8905 1631.7664 1631.7654 1 0 K.AEEYILGDFCFLR.I**  ([Ions score 75](http://mbp-mascot4/mascot/cgi/peptide_view.pl?file=../data/20091021/F006297.dat&query=2072&hit=1&index=IPI00031411&px=1&section=5&ave_thresh=22))

**111 - 121 448.9227 1343.7463 1343.7449 1 1 R.EVKDHYTLIVK.A**  ([Ions score 32](http://mbp-mascot4/mascot/cgi/peptide_view.pl?file=../data/20091021/F006297.dat&query=1618&hit=1&index=IPI00031411&px=1&section=5&ave_thresh=22))

**137 - 165 1078.2319 3231.6740 3231.6776 -1 0 R.VQVLDTNDLRPLFSPTSYSVSLPENTAIR.T**  ([Ions score 40](http://mbp-mascot4/mascot/cgi/peptide_view.pl?file=../data/20091021/F006297.dat&query=3844&hit=1&index=IPI00031411&px=1&section=5&ave_thresh=22))

**190 - 209 734.7161 2201.1265 2201.1263 0 1 K.DRTDMFAIHPTSGVIVLTGR.L**  Oxidation (M) ([Ions score 36](http://mbp-mascot4/mascot/cgi/peptide_view.pl?file=../data/20091021/F006297.dat&query=3068&hit=1&index=IPI00031411&px=1&section=5&ave_thresh=22))

**192 - 209 639.0087 1914.0042 1914.0033 0 0 R.TDMFAIHPTSGVIVLTGR.L**  ([Ions score 48](http://mbp-mascot4/mascot/cgi/peptide_view.pl?file=../data/20091021/F006297.dat&query=2645&hit=1&index=IPI00031411&px=1&section=5&ave_thresh=22))

**192 - 209 639.0088 1914.0047 1914.0033 1 0 R.TDMFAIHPTSGVIVLTGR.L**  ([Ions score 48](http://mbp-mascot4/mascot/cgi/peptide_view.pl?file=../data/20091021/F006297.dat&query=2646&hit=1&index=IPI00031411&px=1&section=5&ave_thresh=22))

**192 - 209 644.3405 1929.9997 1929.9983 1 0 R.TDMFAIHPTSGVIVLTGR.L**  Oxidation (M) ([Ions score 52](http://mbp-mascot4/mascot/cgi/peptide_view.pl?file=../data/20091021/F006297.dat&query=2665&hit=1&index=IPI00031411&px=1&section=5&ave_thresh=22))

**192 - 209 644.3409 1930.0008 1929.9983 1 0 R.TDMFAIHPTSGVIVLTGR.L**  Oxidation (M) ([Ions score 58](http://mbp-mascot4/mascot/cgi/peptide_view.pl?file=../data/20091021/F006297.dat&query=2666&hit=1&index=IPI00031411&px=1&section=5&ave_thresh=22))

**217 - 227 662.3350 1322.6555 1322.6540 1 0 K.LYEMEILAADR.G**  ([Ions score 63](http://mbp-mascot4/mascot/cgi/peptide_view.pl?file=../data/20091021/F006297.dat&query=1577&hit=1&index=IPI00031411&px=1&section=5&ave_thresh=22))

**217 - 227 670.3316 1338.6486 1338.6489 -0 0 K.LYEMEILAADR.G**  Oxidation (M) ([Ions score 93](http://mbp-mascot4/mascot/cgi/peptide_view.pl?file=../data/20091021/F006297.dat&query=1603&hit=1&index=IPI00031411&px=1&section=5&ave_thresh=22))

**217 - 227 670.3318 1338.6490 1338.6489 0 0 K.LYEMEILAADR.G**  Oxidation (M) ([Ions score 80](http://mbp-mascot4/mascot/cgi/peptide_view.pl?file=../data/20091021/F006297.dat&query=1604&hit=1&index=IPI00031411&px=1&section=5&ave_thresh=22))

**243 - 269 988.5092 2962.5058 2962.5070 -0 0 K.LTVHIEQANECAPVITAVTLSPSELDR.D**  ([Ions score 40](http://mbp-mascot4/mascot/cgi/peptide_view.pl?file=../data/20091021/F006297.dat&query=3752&hit=1&index=IPI00031411&px=1&section=5&ave_thresh=22))

**243 - 269 988.5101 2962.5086 2962.5070 1 0 K.LTVHIEQANECAPVITAVTLSPSELDR.D**  ([Ions score 26](http://mbp-mascot4/mascot/cgi/peptide_view.pl?file=../data/20091021/F006297.dat&query=3753&hit=1&index=IPI00031411&px=1&section=5&ave_thresh=22))

**352 - 361 578.3303 1154.6460 1154.6448 1 0 K.VIHVTSPQFK.A**  ([Ions score 36](http://mbp-mascot4/mascot/cgi/peptide_view.pl?file=../data/20091021/F006297.dat&query=1018&hit=1&index=IPI00031411&px=1&section=5&ave_thresh=22))

**374 - 390 914.9791 1827.9436 1827.9441 -0 0 R.AEISEFAPPNTPVVMVK.A**  ([Ions score 51](http://mbp-mascot4/mascot/cgi/peptide_view.pl?file=../data/20091021/F006297.dat&query=2446&hit=1&index=IPI00031411&px=1&section=5&ave_thresh=22))

**374 - 390 914.9794 1827.9443 1827.9441 0 0 R.AEISEFAPPNTPVVMVK.A**  ([Ions score 49](http://mbp-mascot4/mascot/cgi/peptide_view.pl?file=../data/20091021/F006297.dat&query=2447&hit=1&index=IPI00031411&px=1&section=5&ave_thresh=22))

**374 - 390 922.9769 1843.9393 1843.9390 0 0 R.AEISEFAPPNTPVVMVK.A**  Oxidation (M) ([Ions score 56](http://mbp-mascot4/mascot/cgi/peptide_view.pl?file=../data/20091021/F006297.dat&query=2491&hit=1&index=IPI00031411&px=1&section=5&ave_thresh=22))

**374 - 390 922.9769 1843.9393 1843.9390 0 0 R.AEISEFAPPNTPVVMVK.A**  Oxidation (M) ([Ions score 63](http://mbp-mascot4/mascot/cgi/peptide_view.pl?file=../data/20091021/F006297.dat&query=2492&hit=1&index=IPI00031411&px=1&section=5&ave_thresh=22))

**411 - 428 688.7208 2063.1404 2063.1415 -1 1 K.FSLNYNTGLISILEPVKR.Q**  ([Ions score 35](http://mbp-mascot4/mascot/cgi/peptide_view.pl?file=../data/20091021/F006297.dat&query=2860&hit=1&index=IPI00031411&px=1&section=5&ave_thresh=22))

**411 - 428 688.7212 2063.1419 2063.1415 0 1 K.FSLNYNTGLISILEPVKR.Q**  ([Ions score 32](http://mbp-mascot4/mascot/cgi/peptide_view.pl?file=../data/20091021/F006297.dat&query=2861&hit=1&index=IPI00031411&px=1&section=5&ave_thresh=22))

**453 - 469 918.9600 1835.9055 1835.9054 0 0 K.VLGANSNPPEFTQTAYK.A**  ([Ions score 96](http://mbp-mascot4/mascot/cgi/peptide_view.pl?file=../data/20091021/F006297.dat&query=2469&hit=1&index=IPI00031411&px=1&section=5&ave_thresh=22))

**453 - 469 918.9601 1835.9056 1835.9054 0 0 K.VLGANSNPPEFTQTAYK.A**  ([Ions score 81](http://mbp-mascot4/mascot/cgi/peptide_view.pl?file=../data/20091021/F006297.dat&query=2470&hit=1&index=IPI00031411&px=1&section=5&ave_thresh=22))

**540 - 548 532.7616 1063.5086 1063.5087 -0 0 R.ASDWGLPYR.R**  ([Ions score 64](http://mbp-mascot4/mascot/cgi/peptide_view.pl?file=../data/20091021/F006297.dat&query=729&hit=1&index=IPI00031411&px=1&section=5&ave_thresh=22))

**540 - 548 532.7617 1063.5088 1063.5087 0 0 R.ASDWGLPYR.R**  ([Ions score 64](http://mbp-mascot4/mascot/cgi/peptide_view.pl?file=../data/20091021/F006297.dat&query=730&hit=1&index=IPI00031411&px=1&section=5&ave_thresh=22))

**669 - 681 730.8747 1459.7349 1459.7341 1 0 K.LVNLQCEETGVAK.M**  ([Ions score 77](http://mbp-mascot4/mascot/cgi/peptide_view.pl?file=../data/20091021/F006297.dat&query=1812&hit=1&index=IPI00031411&px=1&section=5&ave_thresh=22))

**669 - 681 730.8758 1459.7370 1459.7341 2 0 K.LVNLQCEETGVAK.M**  ([Ions score 94](http://mbp-mascot4/mascot/cgi/peptide_view.pl?file=../data/20091021/F006297.dat&query=1813&hit=1&index=IPI00031411&px=1&section=5&ave_thresh=22))

**693 - 717 734.8577 2935.4018 2935.4002 1 0 K.LHNQGEVEDIFFDSHSVNAHIPQFR.S**  ([Ions score 56](http://mbp-mascot4/mascot/cgi/peptide_view.pl?file=../data/20091021/F006297.dat&query=3716&hit=1&index=IPI00031411&px=1&section=5&ave_thresh=22))

**693 - 717 734.8578 2935.4023 2935.4002 1 0 K.LHNQGEVEDIFFDSHSVNAHIPQFR.S**  ([Ions score 48](http://mbp-mascot4/mascot/cgi/peptide_view.pl?file=../data/20091021/F006297.dat&query=3717&hit=1&index=IPI00031411&px=1&section=5&ave_thresh=22))

**718 - 727 522.3085 1042.6024 1042.6023 0 0 R.STLPTGIQVK.E**  ([Ions score 47](http://mbp-mascot4/mascot/cgi/peptide_view.pl?file=../data/20091021/F006297.dat&query=669&hit=1&index=IPI00031411&px=1&section=5&ave_thresh=22))

**718 - 727 522.3085 1042.6025 1042.6023 0 0 R.STLPTGIQVK.E**  ([Ions score 33](http://mbp-mascot4/mascot/cgi/peptide_view.pl?file=../data/20091021/F006297.dat&query=670&hit=1&index=IPI00031411&px=1&section=5&ave_thresh=22))

**899 - 914 615.9955 1844.9646 1844.9632 1 1 R.DQAREEPQLFSTVVVK.V**  ([Ions score 37](http://mbp-mascot4/mascot/cgi/peptide_view.pl?file=../data/20091021/F006297.dat&query=2495&hit=1&index=IPI00031411&px=1&section=5&ave_thresh=22))

**899 - 914 615.9957 1844.9653 1844.9632 1 1 R.DQAREEPQLFSTVVVK.V**  ([Ions score 55](http://mbp-mascot4/mascot/cgi/peptide_view.pl?file=../data/20091021/F006297.dat&query=2496&hit=1&index=IPI00031411&px=1&section=5&ave_thresh=22))

**903 - 914 688.3771 1374.7396 1374.7395 0 0 R.EEPQLFSTVVVK.V**  ([Ions score 45](http://mbp-mascot4/mascot/cgi/peptide_view.pl?file=../data/20091021/F006297.dat&query=1673&hit=1&index=IPI00031411&px=1&section=5&ave_thresh=22))

**903 - 914 688.3775 1374.7404 1374.7395 1 0 R.EEPQLFSTVVVK.V**  ([Ions score 46](http://mbp-mascot4/mascot/cgi/peptide_view.pl?file=../data/20091021/F006297.dat&query=1674&hit=1&index=IPI00031411&px=1&section=5&ave_thresh=22))

**915 - 933 1094.0393 2186.0640 2186.0644 -0 0 K.VSLEDVNDNPPTFIPPNYR.V**  ([Ions score 54](http://mbp-mascot4/mascot/cgi/peptide_view.pl?file=../data/20091021/F006297.dat&query=3036&hit=1&index=IPI00031411&px=1&section=5&ave_thresh=22))

**915 - 933 1094.0403 2186.0660 2186.0644 1 0 K.VSLEDVNDNPPTFIPPNYR.V**  ([Ions score 55](http://mbp-mascot4/mascot/cgi/peptide_view.pl?file=../data/20091021/F006297.dat&query=3037&hit=1&index=IPI00031411&px=1&section=5&ave_thresh=22))

**936 - 963 787.6435 3146.5451 3146.5455 -0 1 K.VREDLPEGTVIMWLEAHDPDLGQSGQVR.Y**  ([Ions score 39](http://mbp-mascot4/mascot/cgi/peptide_view.pl?file=../data/20091021/F006297.dat&query=3824&hit=1&index=IPI00031411&px=1&section=5&ave_thresh=22))

**936 - 963 787.6443 3146.5483 3146.5455 1 1 K.VREDLPEGTVIMWLEAHDPDLGQSGQVR.Y**  ([Ions score 48](http://mbp-mascot4/mascot/cgi/peptide_view.pl?file=../data/20091021/F006297.dat&query=3825&hit=1&index=IPI00031411&px=1&section=5&ave_thresh=22))

**936 - 963 791.6428 3162.5422 3162.5404 1 1 K.VREDLPEGTVIMWLEAHDPDLGQSGQVR.Y**  Oxidation (M) ([Ions score 23](http://mbp-mascot4/mascot/cgi/peptide_view.pl?file=../data/20091021/F006297.dat&query=3832&hit=1&index=IPI00031411&px=1&section=5&ave_thresh=22))

**936 - 963 791.6430 3162.5429 3162.5404 1 1 K.VREDLPEGTVIMWLEAHDPDLGQSGQVR.Y**  Oxidation (M) ([Ions score 54](http://mbp-mascot4/mascot/cgi/peptide_view.pl?file=../data/20091021/F006297.dat&query=3833&hit=1&index=IPI00031411&px=1&section=5&ave_thresh=22))

**964 - 978 570.2658 1707.7756 1707.7740 1 0 R.YSLLDHGEGNFDVDK.L**  ([Ions score 36](http://mbp-mascot4/mascot/cgi/peptide_view.pl?file=../data/20091021/F006297.dat&query=2181&hit=1&index=IPI00031411&px=1&section=5&ave_thresh=22))

**964 - 978 570.2660 1707.7761 1707.7740 1 0 R.YSLLDHGEGNFDVDK.L**  ([Ions score 42](http://mbp-mascot4/mascot/cgi/peptide_view.pl?file=../data/20091021/F006297.dat&query=2182&hit=1&index=IPI00031411&px=1&section=5&ave_thresh=22))

**964 - 984 764.7133 2291.1179 2291.1182 -0 1 R.YSLLDHGEGNFDVDKLSGAVR.I**  ([Ions score 65](http://mbp-mascot4/mascot/cgi/peptide_view.pl?file=../data/20091021/F006297.dat&query=3212&hit=1&index=IPI00031411&px=1&section=5&ave_thresh=22))

**964 - 984 573.7872 2291.1195 2291.1182 1 1 R.YSLLDHGEGNFDVDKLSGAVR.I**  ([Ions score 24](http://mbp-mascot4/mascot/cgi/peptide_view.pl?file=../data/20091021/F006297.dat&query=3213&hit=1&index=IPI00031411&px=1&section=5&ave_thresh=22))

**964 - 984 573.7872 2291.1198 2291.1182 1 1 R.YSLLDHGEGNFDVDKLSGAVR.I**  ([Ions score 33](http://mbp-mascot4/mascot/cgi/peptide_view.pl?file=../data/20091021/F006297.dat&query=3214&hit=1&index=IPI00031411&px=1&section=5&ave_thresh=22))

**964 - 984 764.7140 2291.1201 2291.1182 1 1 R.YSLLDHGEGNFDVDKLSGAVR.I**  ([Ions score 64](http://mbp-mascot4/mascot/cgi/peptide_view.pl?file=../data/20091021/F006297.dat&query=3215&hit=1&index=IPI00031411&px=1&section=5&ave_thresh=22))

**985 - 993 560.3061 1118.5976 1118.5972 0 0 R.IVQQLDFEK.K**  ([Ions score 52](http://mbp-mascot4/mascot/cgi/peptide_view.pl?file=../data/20091021/F006297.dat&query=913&hit=1&index=IPI00031411&px=1&section=5&ave_thresh=22))

**985 - 993 560.3063 1118.5981 1118.5972 1 0 R.IVQQLDFEK.K**  ([Ions score 52](http://mbp-mascot4/mascot/cgi/peptide_view.pl?file=../data/20091021/F006297.dat&query=914&hit=1&index=IPI00031411&px=1&section=5&ave_thresh=22))

**1084 - 1096 703.3433 1404.6720 1404.6732 -1 0 K.IGEETGVIETSDR.L**  ([Ions score 72](http://mbp-mascot4/mascot/cgi/peptide_view.pl?file=../data/20091021/F006297.dat&query=1740&hit=1&index=IPI00031411&px=1&section=5&ave_thresh=22))

**1084 - 1096 703.3439 1404.6732 1404.6732 -0 0 K.IGEETGVIETSDR.L**  ([Ions score 69](http://mbp-mascot4/mascot/cgi/peptide_view.pl?file=../data/20091021/F006297.dat&query=1741&hit=1&index=IPI00031411&px=1&section=5&ave_thresh=22))

**1084 - 1099 597.3025 1788.8856 1788.8854 0 1 K.IGEETGVIETSDRLDR.E**  ([Ions score 39](http://mbp-mascot4/mascot/cgi/peptide_view.pl?file=../data/20091021/F006297.dat&query=2373&hit=1&index=IPI00031411&px=1&section=5&ave_thresh=22))

**1084 - 1099 597.3026 1788.8860 1788.8854 0 1 K.IGEETGVIETSDRLDR.E**  ([Ions score 54](http://mbp-mascot4/mascot/cgi/peptide_view.pl?file=../data/20091021/F006297.dat&query=2374&hit=1&index=IPI00031411&px=1&section=5&ave_thresh=22))

**1155 - 1173 1026.4739 2050.9332 2050.9331 0 0 K.DVSVVQIEAFDPDSSSNDK.L**  ([Ions score 91](http://mbp-mascot4/mascot/cgi/peptide_view.pl?file=../data/20091021/F006297.dat&query=2848&hit=1&index=IPI00031411&px=1&section=5&ave_thresh=22))

**1155 - 1173 1026.4746 2050.9347 2050.9331 1 0 K.DVSVVQIEAFDPDSSSNDK.L**  ([Ions score 96](http://mbp-mascot4/mascot/cgi/peptide_view.pl?file=../data/20091021/F006297.dat&query=2849&hit=1&index=IPI00031411&px=1&section=5&ave_thresh=22))

**1178 - 1192 543.9515 1628.8328 1628.8311 1 0 K.ITSGNPQGFFSIHPK.T**  ([Ions score 36](http://mbp-mascot4/mascot/cgi/peptide_view.pl?file=../data/20091021/F006297.dat&query=2065&hit=1&index=IPI00031411&px=1&section=5&ave_thresh=22))

**1206 - 1225 746.0295 2235.0666 2235.0655 0 0 R.EQQDEHILEVTVTDNGSPPK.S**  ([Ions score 26](http://mbp-mascot4/mascot/cgi/peptide_view.pl?file=../data/20091021/F006297.dat&query=3123&hit=1&index=IPI00031411&px=1&section=5&ave_thresh=22))

**1235 - 1248 567.9653 1700.8740 1700.8733 0 0 K.ILDENDNKPQFLQK.F**  ([Ions score 52](http://mbp-mascot4/mascot/cgi/peptide_view.pl?file=../data/20091021/F006297.dat&query=2156&hit=1&index=IPI00031411&px=1&section=5&ave_thresh=22))

**1235 - 1248 567.9654 1700.8745 1700.8733 1 0 K.ILDENDNKPQFLQK.F**  ([Ions score 43](http://mbp-mascot4/mascot/cgi/peptide_view.pl?file=../data/20091021/F006297.dat&query=2157&hit=1&index=IPI00031411&px=1&section=5&ave_thresh=22))

**1300 - 1305 390.7187 779.4229 779.4218 1 0 K.FFIEPK.T**  ([Ions score 22](http://mbp-mascot4/mascot/cgi/peptide_view.pl?file=../data/20091021/F006297.dat&query=3&hit=1&index=IPI00031411&px=1&section=5&ave_thresh=22))

**1300 - 1305 390.7187 779.4229 779.4218 2 0 K.FFIEPK.T**  ([Ions score 25](http://mbp-mascot4/mascot/cgi/peptide_view.pl?file=../data/20091021/F006297.dat&query=4&hit=1&index=IPI00031411&px=1&section=5&ave_thresh=22))

**1314 - 1326 707.3666 1412.7187 1412.7187 -0 0 R.FSAAGEYDILSIK.A**  ([Ions score 92](http://mbp-mascot4/mascot/cgi/peptide_view.pl?file=../data/20091021/F006297.dat&query=1749&hit=1&index=IPI00031411&px=1&section=5&ave_thresh=22))

**1314 - 1326 707.3667 1412.7188 1412.7187 0 0 R.FSAAGEYDILSIK.A**  ([Ions score 82](http://mbp-mascot4/mascot/cgi/peptide_view.pl?file=../data/20091021/F006297.dat&query=1750&hit=1&index=IPI00031411&px=1&section=5&ave_thresh=22))

**1407 - 1422 547.6454 1639.9143 1639.9145 -0 0 K.GTGTIIVAKPLDAEQK.S**  ([Ions score 38](http://mbp-mascot4/mascot/cgi/peptide_view.pl?file=../data/20091021/F006297.dat&query=2078&hit=1&index=IPI00031411&px=1&section=5&ave_thresh=22))

**1407 - 1422 820.9654 1639.9162 1639.9145 1 0 K.GTGTIIVAKPLDAEQK.S**  ([Ions score 52](http://mbp-mascot4/mascot/cgi/peptide_view.pl?file=../data/20091021/F006297.dat&query=2079&hit=1&index=IPI00031411&px=1&section=5&ave_thresh=22))

**1407 - 1422 547.6460 1639.9163 1639.9145 1 0 K.GTGTIIVAKPLDAEQK.S**  ([Ions score 36](http://mbp-mascot4/mascot/cgi/peptide_view.pl?file=../data/20091021/F006297.dat&query=2080&hit=1&index=IPI00031411&px=1&section=5&ave_thresh=22))

**1407 - 1422 820.9655 1639.9163 1639.9145 1 0 K.GTGTIIVAKPLDAEQK.S**  ([Ions score 55](http://mbp-mascot4/mascot/cgi/peptide_view.pl?file=../data/20091021/F006297.dat&query=2081&hit=1&index=IPI00031411&px=1&section=5&ave_thresh=22))

**1461 - 1488 1058.5294 3172.5664 3172.5663 0 1 K.YEVVIPEDTAPETEILQISAVDQDEKNK.L**  ([Ions score 37](http://mbp-mascot4/mascot/cgi/peptide_view.pl?file=../data/20091021/F006297.dat&query=3834&hit=1&index=IPI00031411&px=1&section=5&ave_thresh=22))

**1489 - 1497 540.8060 1079.5974 1079.5975 -0 0 K.LIYTLQSSR.D**  ([Ions score 69](http://mbp-mascot4/mascot/cgi/peptide_view.pl?file=../data/20091021/F006297.dat&query=774&hit=1&index=IPI00031411&px=1&section=5&ave_thresh=22))

**1489 - 1497 540.8063 1079.5980 1079.5975 0 0 K.LIYTLQSSR.D**  ([Ions score 60](http://mbp-mascot4/mascot/cgi/peptide_view.pl?file=../data/20091021/F006297.dat&query=775&hit=1&index=IPI00031411&px=1&section=5&ave_thresh=22))

**1505 - 1519 842.9307 1683.8468 1683.8468 -0 1 K.FRLDPATGSLYTSEK.L**  ([Ions score 70](http://mbp-mascot4/mascot/cgi/peptide_view.pl?file=../data/20091021/F006297.dat&query=2136&hit=1&index=IPI00031411&px=1&section=5&ave_thresh=22))

**1505 - 1519 562.2899 1683.8477 1683.8468 1 1 K.FRLDPATGSLYTSEK.L**  ([Ions score 58](http://mbp-mascot4/mascot/cgi/peptide_view.pl?file=../data/20091021/F006297.dat&query=2137&hit=1&index=IPI00031411&px=1&section=5&ave_thresh=22))

**1505 - 1519 562.2900 1683.8483 1683.8468 1 1 K.FRLDPATGSLYTSEK.L**  ([Ions score 43](http://mbp-mascot4/mascot/cgi/peptide_view.pl?file=../data/20091021/F006297.dat&query=2138&hit=1&index=IPI00031411&px=1&section=5&ave_thresh=22))

**1507 - 1519 691.3459 1380.6773 1380.6773 0 0 R.LDPATGSLYTSEK.L**  ([Ions score 85](http://mbp-mascot4/mascot/cgi/peptide_view.pl?file=../data/20091021/F006297.dat&query=1687&hit=1&index=IPI00031411&px=1&section=5&ave_thresh=22))

**1507 - 1519 691.3463 1380.6781 1380.6773 1 0 R.LDPATGSLYTSEK.L**  ([Ions score 102](http://mbp-mascot4/mascot/cgi/peptide_view.pl?file=../data/20091021/F006297.dat&query=1688&hit=1&index=IPI00031411&px=1&section=5&ave_thresh=22))

**1520 - 1535 472.2483 1884.9642 1884.9629 1 0 K.LDHEAVHQHTLTVMVR.D**  ([Ions score 30](http://mbp-mascot4/mascot/cgi/peptide_view.pl?file=../data/20091021/F006297.dat&query=2601&hit=1&index=IPI00031411&px=1&section=5&ave_thresh=22))

**1631 - 1641 649.3085 1296.6024 1296.6020 0 0 R.SNQAEYDLMVK.A**  ([Ions score 68](http://mbp-mascot4/mascot/cgi/peptide_view.pl?file=../data/20091021/F006297.dat&query=1449&hit=1&index=IPI00031411&px=1&section=5&ave_thresh=22))

**1631 - 1641 649.3086 1296.6027 1296.6020 1 0 R.SNQAEYDLMVK.A**  ([Ions score 65](http://mbp-mascot4/mascot/cgi/peptide_view.pl?file=../data/20091021/F006297.dat&query=1450&hit=1&index=IPI00031411&px=1&section=5&ave_thresh=22))

**1631 - 1641 657.3065 1312.5985 1312.5969 1 0 R.SNQAEYDLMVK.A**  Oxidation (M) ([Ions score 79](http://mbp-mascot4/mascot/cgi/peptide_view.pl?file=../data/20091021/F006297.dat&query=1511&hit=1&index=IPI00031411&px=1&section=5&ave_thresh=22))

**1646 - 1657 638.8137 1275.6129 1275.6129 -0 0 K.GSPPMSEITSVR.I**  Oxidation (M) ([Ions score 55](http://mbp-mascot4/mascot/cgi/peptide_view.pl?file=../data/20091021/F006297.dat&query=1377&hit=1&index=IPI00031411&px=1&section=5&ave_thresh=22))

**1646 - 1657 638.8138 1275.6130 1275.6129 0 0 K.GSPPMSEITSVR.I**  Oxidation (M) ([Ions score 74](http://mbp-mascot4/mascot/cgi/peptide_view.pl?file=../data/20091021/F006297.dat&query=1378&hit=1&index=IPI00031411&px=1&section=5&ave_thresh=22))

**1658 - 1669 638.3506 1274.6867 1274.6870 -0 0 R.IFVTIADNASPK.F**  ([Ions score 83](http://mbp-mascot4/mascot/cgi/peptide_view.pl?file=../data/20091021/F006297.dat&query=1375&hit=1&index=IPI00031411&px=1&section=5&ave_thresh=22))

**1658 - 1669 638.3507 1274.6869 1274.6870 -0 0 R.IFVTIADNASPK.F**  ([Ions score 85](http://mbp-mascot4/mascot/cgi/peptide_view.pl?file=../data/20091021/F006297.dat&query=1376&hit=1&index=IPI00031411&px=1&section=5&ave_thresh=22))

**1706 - 1726 734.3539 2200.0400 2200.0397 0 0 K.DGNTGDAFDINPHSGTIITQK.A**  ([Ions score 62](http://mbp-mascot4/mascot/cgi/peptide_view.pl?file=../data/20091021/F006297.dat&query=3065&hit=1&index=IPI00031411&px=1&section=5&ave_thresh=22))

**1706 - 1726 734.3541 2200.0404 2200.0397 0 0 K.DGNTGDAFDINPHSGTIITQK.A**  ([Ions score 52](http://mbp-mascot4/mascot/cgi/peptide_view.pl?file=../data/20091021/F006297.dat&query=3066&hit=1&index=IPI00031411&px=1&section=5&ave_thresh=22))

**1982 - 1992 628.8293 1255.6440 1255.6449 -1 0 K.FTQDVYSAVVK.E**  ([Ions score 74](http://mbp-mascot4/mascot/cgi/peptide_view.pl?file=../data/20091021/F006297.dat&query=1315&hit=1&index=IPI00031411&px=1&section=5&ave_thresh=22))

**1982 - 1992 628.8297 1255.6449 1255.6449 -0 0 K.FTQDVYSAVVK.E**  ([Ions score 81](http://mbp-mascot4/mascot/cgi/peptide_view.pl?file=../data/20091021/F006297.dat&query=1316&hit=1&index=IPI00031411&px=1&section=5&ave_thresh=22))

**2031 - 2044 719.8624 1437.7102 1437.7100 0 0 R.TSGVLSTTGTPFDR.E**  ([Ions score 101](http://mbp-mascot4/mascot/cgi/peptide_view.pl?file=../data/20091021/F006297.dat&query=1778&hit=1&index=IPI00031411&px=1&section=5&ave_thresh=22))

**2031 - 2044 719.8625 1437.7104 1437.7100 0 0 R.TSGVLSTTGTPFDR.E**  ([Ions score 96](http://mbp-mascot4/mascot/cgi/peptide_view.pl?file=../data/20091021/F006297.dat&query=1779&hit=1&index=IPI00031411&px=1&section=5&ave_thresh=22))

**2072 - 2094 1303.6771 2605.3397 2605.3428 -1 0 K.VIVEDQNDNAPVFVNLPYYAVVK.V**  ([Ions score 55](http://mbp-mascot4/mascot/cgi/peptide_view.pl?file=../data/20091021/F006297.dat&query=3554&hit=1&index=IPI00031411&px=1&section=5&ave_thresh=22))

**2072 - 2094 869.4550 2605.3430 2605.3428 0 0 K.VIVEDQNDNAPVFVNLPYYAVVK.V**  ([Ions score 50](http://mbp-mascot4/mascot/cgi/peptide_view.pl?file=../data/20091021/F006297.dat&query=3555&hit=1&index=IPI00031411&px=1&section=5&ave_thresh=22))

**2072 - 2094 869.4551 2605.3434 2605.3428 0 0 K.VIVEDQNDNAPVFVNLPYYAVVK.V**  ([Ions score 56](http://mbp-mascot4/mascot/cgi/peptide_view.pl?file=../data/20091021/F006297.dat&query=3556&hit=1&index=IPI00031411&px=1&section=5&ave_thresh=22))

**2072 - 2094 1303.6802 2605.3458 2605.3428 1 0 K.VIVEDQNDNAPVFVNLPYYAVVK.V**  ([Ions score 55](http://mbp-mascot4/mascot/cgi/peptide_view.pl?file=../data/20091021/F006297.dat&query=3557&hit=1&index=IPI00031411&px=1&section=5&ave_thresh=22))

**2125 - 2141 493.5080 1970.0027 1970.0010 1 0 K.EHHEHFQIGPLGEISLK.K**  ([Ions score 26](http://mbp-mascot4/mascot/cgi/peptide_view.pl?file=../data/20091021/F006297.dat&query=2731&hit=1&index=IPI00031411&px=1&section=5&ave_thresh=22))

**2125 - 2141 493.5080 1970.0029 1970.0010 1 0 K.EHHEHFQIGPLGEISLK.K**  ([Ions score 30](http://mbp-mascot4/mascot/cgi/peptide_view.pl?file=../data/20091021/F006297.dat&query=2732&hit=1&index=IPI00031411&px=1&section=5&ave_thresh=22))

**2125 - 2142 525.5317 2098.0976 2098.0959 1 1 K.EHHEHFQIGPLGEISLKK.Q**  ([Ions score 39](http://mbp-mascot4/mascot/cgi/peptide_view.pl?file=../data/20091021/F006297.dat&query=2898&hit=1&index=IPI00031411&px=1&section=5&ave_thresh=22))

**2143 - 2151 554.2877 1106.5609 1106.5608 0 0 K.QFELDTLNK.E**  ([Ions score 53](http://mbp-mascot4/mascot/cgi/peptide_view.pl?file=../data/20091021/F006297.dat&query=900&hit=1&index=IPI00031411&px=1&section=5&ave_thresh=22))

**2143 - 2151 554.2878 1106.5611 1106.5608 0 0 K.QFELDTLNK.E**  ([Ions score 52](http://mbp-mascot4/mascot/cgi/peptide_view.pl?file=../data/20091021/F006297.dat&query=901&hit=1&index=IPI00031411&px=1&section=5&ave_thresh=22))

**2143 - 2160 704.0532 2109.1378 2109.1358 1 1 K.QFELDTLNKEYLVTVVAK.D**  ([Ions score 67](http://mbp-mascot4/mascot/cgi/peptide_view.pl?file=../data/20091021/F006297.dat&query=2924&hit=1&index=IPI00031411&px=1&section=5&ave_thresh=22))

**2143 - 2160 704.0532 2109.1378 2109.1358 1 1 K.QFELDTLNKEYLVTVVAK.D**  ([Ions score 62](http://mbp-mascot4/mascot/cgi/peptide_view.pl?file=../data/20091021/F006297.dat&query=2925&hit=1&index=IPI00031411&px=1&section=5&ave_thresh=22))

**2152 - 2160 511.3003 1020.5860 1020.5855 0 0 K.EYLVTVVAK.D**  ([Ions score 34](http://mbp-mascot4/mascot/cgi/peptide_view.pl?file=../data/20091021/F006297.dat&query=604&hit=1&index=IPI00031411&px=1&section=5&ave_thresh=22))

**2152 - 2160 511.3004 1020.5863 1020.5855 1 0 K.EYLVTVVAK.D**  ([Ions score 33](http://mbp-mascot4/mascot/cgi/peptide_view.pl?file=../data/20091021/F006297.dat&query=605&hit=1&index=IPI00031411&px=1&section=5&ave_thresh=22))

**2161 - 2180 1030.0288 2058.0431 2058.0456 -1 0 K.DGGNPAFSAEVIVPITVMNK.A**  ([Ions score 69](http://mbp-mascot4/mascot/cgi/peptide_view.pl?file=../data/20091021/F006297.dat&query=2854&hit=1&index=IPI00031411&px=1&section=5&ave_thresh=22))

**2332 - 2349 506.0112 2020.0155 2020.0126 1 0 K.SHDHFHVDSSTGLISLLR.T**  ([Ions score 40](http://mbp-mascot4/mascot/cgi/peptide_view.pl?file=../data/20091021/F006297.dat&query=2807&hit=1&index=IPI00031411&px=1&section=5&ave_thresh=22))

**2402 - 2416 430.4668 1717.8382 1717.8359 1 0 R.ISEHAPHGHFVTCVK.A**  ([Ions score 27](http://mbp-mascot4/mascot/cgi/peptide_view.pl?file=../data/20091021/F006297.dat&query=2209&hit=1&index=IPI00031411&px=1&section=5&ave_thresh=22))

**2417 - 2439 852.4023 2554.1850 2554.1823 1 1 K.AYDADSSDIDKLQYSILSGNDHK.H**  ([Ions score 59](http://mbp-mascot4/mascot/cgi/peptide_view.pl?file=../data/20091021/F006297.dat&query=3511&hit=1&index=IPI00031411&px=1&section=5&ave_thresh=22))

**2440 - 2457 665.3657 1993.0751 1993.0745 0 0 K.HFVIDSATGIITLSNLHR.H**  ([Ions score 85](http://mbp-mascot4/mascot/cgi/peptide_view.pl?file=../data/20091021/F006297.dat&query=2776&hit=1&index=IPI00031411&px=1&section=5&ave_thresh=22))

**2440 - 2457 665.3658 1993.0755 1993.0745 0 0 K.HFVIDSATGIITLSNLHR.H**  ([Ions score 77](http://mbp-mascot4/mascot/cgi/peptide_view.pl?file=../data/20091021/F006297.dat&query=2777&hit=1&index=IPI00031411&px=1&section=5&ave_thresh=22))

**2458 - 2476 717.3853 2149.1341 2149.1320 1 0 R.HALKPFYSLNLSVSDGVFR.S**  ([Ions score 46](http://mbp-mascot4/mascot/cgi/peptide_view.pl?file=../data/20091021/F006297.dat&query=2985&hit=1&index=IPI00031411&px=1&section=5&ave_thresh=22))

**2458 - 2476 717.3857 2149.1354 2149.1320 2 0 R.HALKPFYSLNLSVSDGVFR.S**  ([Ions score 48](http://mbp-mascot4/mascot/cgi/peptide_view.pl?file=../data/20091021/F006297.dat&query=2986&hit=1&index=IPI00031411&px=1&section=5&ave_thresh=22))

**2580 - 2600 803.7211 2408.1415 2408.1431 -1 0 K.VAFCTVNVILTDDNDNAPQFR.A**  ([Ions score 44](http://mbp-mascot4/mascot/cgi/peptide_view.pl?file=../data/20091021/F006297.dat&query=3418&hit=1&index=IPI00031411&px=1&section=5&ave_thresh=22))

**2580 - 2600 803.7222 2408.1448 2408.1431 1 0 K.VAFCTVNVILTDDNDNAPQFR.A**  ([Ions score 61](http://mbp-mascot4/mascot/cgi/peptide_view.pl?file=../data/20091021/F006297.dat&query=3419&hit=1&index=IPI00031411&px=1&section=5&ave_thresh=22))

**2580 - 2600 1205.0797 2408.1449 2408.1431 1 0 K.VAFCTVNVILTDDNDNAPQFR.A**  ([Ions score 111](http://mbp-mascot4/mascot/cgi/peptide_view.pl?file=../data/20091021/F006297.dat&query=3420&hit=1&index=IPI00031411&px=1&section=5&ave_thresh=22))

**2604 - 2614 569.7906 1137.5667 1137.5666 0 0 K.YEVNIGSSAAK.G**  ([Ions score 70](http://mbp-mascot4/mascot/cgi/peptide_view.pl?file=../data/20091021/F006297.dat&query=989&hit=1&index=IPI00031411&px=1&section=5&ave_thresh=22))

**2604 - 2614 569.7908 1137.5670 1137.5666 0 0 K.YEVNIGSSAAK.G**  ([Ions score 57](http://mbp-mascot4/mascot/cgi/peptide_view.pl?file=../data/20091021/F006297.dat&query=990&hit=1&index=IPI00031411&px=1&section=5&ave_thresh=22))

**2621 - 2646 890.7472 2669.2197 2669.2191 0 0 K.VLASDADEGSNADITYAIEADSESVK.E**  ([Ions score 39](http://mbp-mascot4/mascot/cgi/peptide_view.pl?file=../data/20091021/F006297.dat&query=3605&hit=1&index=IPI00031411&px=1&section=5&ave_thresh=22))

**2621 - 2653 1170.8910 3509.6511 3509.6532 -1 1 K.VLASDADEGSNADITYAIEADSESVKENLEINK.L**  ([Ions score 122](http://mbp-mascot4/mascot/cgi/peptide_view.pl?file=../data/20091021/F006297.dat&query=4036&hit=1&index=IPI00031411&px=1&section=5&ave_thresh=22))

**2621 - 2653 1170.8912 3509.6519 3509.6532 -0 1 K.VLASDADEGSNADITYAIEADSESVKENLEINK.L**  ([Ions score 123](http://mbp-mascot4/mascot/cgi/peptide_view.pl?file=../data/20091021/F006297.dat&query=4037&hit=1&index=IPI00031411&px=1&section=5&ave_thresh=22))

**2687 - 2695 518.3083 1034.6020 1034.6012 1 0 K.ESVVLVYVK.I**  ([Ions score 54](http://mbp-mascot4/mascot/cgi/peptide_view.pl?file=../data/20091021/F006297.dat&query=627&hit=1&index=IPI00031411&px=1&section=5&ave_thresh=22))

**2687 - 2695 518.3083 1034.6020 1034.6012 1 0 K.ESVVLVYVK.I**  ([Ions score 49](http://mbp-mascot4/mascot/cgi/peptide_view.pl?file=../data/20091021/F006297.dat&query=628&hit=1&index=IPI00031411&px=1&section=5&ave_thresh=22))

**2730 - 2742 702.3798 1402.7450 1402.7456 -0 0 R.AEHSGTVLYSLVK.G**  ([Ions score 67](http://mbp-mascot4/mascot/cgi/peptide_view.pl?file=../data/20091021/F006297.dat&query=1733&hit=1&index=IPI00031411&px=1&section=5&ave_thresh=22))

**2730 - 2742 702.3800 1402.7454 1402.7456 -0 0 R.AEHSGTVLYSLVK.G**  ([Ions score 66](http://mbp-mascot4/mascot/cgi/peptide_view.pl?file=../data/20091021/F006297.dat&query=1734&hit=1&index=IPI00031411&px=1&section=5&ave_thresh=22))

**2743 - 2758 612.6226 1834.8458 1834.8446 1 1 K.GNTPESNRDESFVIDR.Q**  ([Ions score 26](http://mbp-mascot4/mascot/cgi/peptide_view.pl?file=../data/20091021/F006297.dat&query=2460&hit=1&index=IPI00031411&px=1&section=5&ave_thresh=22))

**2743 - 2758 612.6226 1834.8460 1834.8446 1 1 K.GNTPESNRDESFVIDR.Q**  ([Ions score 25](http://mbp-mascot4/mascot/cgi/peptide_view.pl?file=../data/20091021/F006297.dat&query=2461&hit=1&index=IPI00031411&px=1&section=5&ave_thresh=22))

**2776 - 2784 592.3164 1182.6182 1182.6186 -0 0 K.WYQFSILAR.C**  ([Ions score 57](http://mbp-mascot4/mascot/cgi/peptide_view.pl?file=../data/20091021/F006297.dat&query=1070&hit=1&index=IPI00031411&px=1&section=5&ave_thresh=22))

**2776 - 2784 592.3166 1182.6187 1182.6186 0 0 K.WYQFSILAR.C**  ([Ions score 66](http://mbp-mascot4/mascot/cgi/peptide_view.pl?file=../data/20091021/F006297.dat&query=1071&hit=1&index=IPI00031411&px=1&section=5&ave_thresh=22))

**2804 - 2830 971.1169 2910.3290 2910.3308 -1 0 K.DANDNSPVFESSPYEAFIVENLPGGSR.V**  ([Ions score 65](http://mbp-mascot4/mascot/cgi/peptide_view.pl?file=../data/20091021/F006297.dat&query=3704&hit=1&index=IPI00031411&px=1&section=5&ave_thresh=22))

**2804 - 2830 971.1180 2910.3323 2910.3308 1 0 K.DANDNSPVFESSPYEAFIVENLPGGSR.V**  ([Ions score 65](http://mbp-mascot4/mascot/cgi/peptide_view.pl?file=../data/20091021/F006297.dat&query=3705&hit=1&index=IPI00031411&px=1&section=5&ave_thresh=22))

**2804 - 2830 1456.1742 2910.3338 2910.3308 1 0 K.DANDNSPVFESSPYEAFIVENLPGGSR.V**  ([Ions score 74](http://mbp-mascot4/mascot/cgi/peptide_view.pl?file=../data/20091021/F006297.dat&query=3706&hit=1&index=IPI00031411&px=1&section=5&ave_thresh=22))

**2993 - 3009 923.9568 1845.8991 1845.8996 -0 0 R.DNYLLTITATDGTFSSK.A**  ([Ions score 109](http://mbp-mascot4/mascot/cgi/peptide_view.pl?file=../data/20091021/F006297.dat&query=2497&hit=1&index=IPI00031411&px=1&section=5&ave_thresh=22))

**2993 - 3009 923.9576 1845.9007 1845.8996 1 0 R.DNYLLTITATDGTFSSK.A**  ([Ions score 112](http://mbp-mascot4/mascot/cgi/peptide_view.pl?file=../data/20091021/F006297.dat&query=2498&hit=1&index=IPI00031411&px=1&section=5&ave_thresh=22))

**3016 - 3028 730.8385 1459.6624 1459.6613 1 0 K.VLDANDNSPVCEK.T**  ([Ions score 56](http://mbp-mascot4/mascot/cgi/peptide_view.pl?file=../data/20091021/F006297.dat&query=1810&hit=1&index=IPI00031411&px=1&section=5&ave_thresh=22))

**3016 - 3028 730.8387 1459.6628 1459.6613 1 0 K.VLDANDNSPVCEK.T**  ([Ions score 67](http://mbp-mascot4/mascot/cgi/peptide_view.pl?file=../data/20091021/F006297.dat&query=1811&hit=1&index=IPI00031411&px=1&section=5&ave_thresh=22))

**3029 - 3043 824.4277 1646.8408 1646.8403 0 0 K.TLYSDTIPEDVLPGK.L**  ([Ions score 60](http://mbp-mascot4/mascot/cgi/peptide_view.pl?file=../data/20091021/F006297.dat&query=2086&hit=1&index=IPI00031411&px=1&section=5&ave_thresh=22))

**3029 - 3043 824.4278 1646.8410 1646.8403 0 0 K.TLYSDTIPEDVLPGK.L**  ([Ions score 82](http://mbp-mascot4/mascot/cgi/peptide_view.pl?file=../data/20091021/F006297.dat&query=2087&hit=1&index=IPI00031411&px=1&section=5&ave_thresh=22))

**3044 - 3056 723.8847 1445.7548 1445.7548 0 0 K.LIMQISATDADIR.S**  ([Ions score 99](http://mbp-mascot4/mascot/cgi/peptide_view.pl?file=../data/20091021/F006297.dat&query=1794&hit=1&index=IPI00031411&px=1&section=5&ave_thresh=22))

**3044 - 3056 723.8854 1445.7563 1445.7548 1 0 K.LIMQISATDADIR.S**  ([Ions score 94](http://mbp-mascot4/mascot/cgi/peptide_view.pl?file=../data/20091021/F006297.dat&query=1795&hit=1&index=IPI00031411&px=1&section=5&ave_thresh=22))

**3044 - 3056 731.8822 1461.7498 1461.7497 0 0 K.LIMQISATDADIR.S**  Oxidation (M) ([Ions score 73](http://mbp-mascot4/mascot/cgi/peptide_view.pl?file=../data/20091021/F006297.dat&query=1818&hit=1&index=IPI00031411&px=1&section=5&ave_thresh=22))

**3044 - 3056 731.8823 1461.7500 1461.7497 0 0 K.LIMQISATDADIR.S**  Oxidation (M) ([Ions score 86](http://mbp-mascot4/mascot/cgi/peptide_view.pl?file=../data/20091021/F006297.dat&query=1819&hit=1&index=IPI00031411&px=1&section=5&ave_thresh=22))

**3057 - 3072 827.4202 1652.8258 1652.8257 0 0 R.SNAEITYTLLGSGAEK.F**  ([Ions score 138](http://mbp-mascot4/mascot/cgi/peptide_view.pl?file=../data/20091021/F006297.dat&query=2095&hit=1&index=IPI00031411&px=1&section=5&ave_thresh=22))

**3057 - 3072 827.4212 1652.8278 1652.8257 1 0 R.SNAEITYTLLGSGAEK.F**  ([Ions score 103](http://mbp-mascot4/mascot/cgi/peptide_view.pl?file=../data/20091021/F006297.dat&query=2096&hit=1&index=IPI00031411&px=1&section=5&ave_thresh=22))

**3084 - 3101 709.7118 2126.1135 2126.1120 1 1 K.TSTPLDREEQAVYHLLVR.A**  ([Ions score 49](http://mbp-mascot4/mascot/cgi/peptide_view.pl?file=../data/20091021/F006297.dat&query=2946&hit=1&index=IPI00031411&px=1&section=5&ave_thresh=22))

**3084 - 3101 709.7123 2126.1150 2126.1120 1 1 K.TSTPLDREEQAVYHLLVR.A**  ([Ions score 50](http://mbp-mascot4/mascot/cgi/peptide_view.pl?file=../data/20091021/F006297.dat&query=2948&hit=1&index=IPI00031411&px=1&section=5&ave_thresh=22))

**3261 - 3280 721.0189 2160.0347 2160.0334 1 0 R.DIEANAEITYSIISGNEHGK.F**  ([Ions score 25](http://mbp-mascot4/mascot/cgi/peptide_view.pl?file=../data/20091021/F006297.dat&query=3001&hit=1&index=IPI00031411&px=1&section=5&ave_thresh=22))

**3491 - 3507 887.4913 1772.9681 1772.9672 0 0 K.AFEVNPQGVLLTSSAIK.R**  ([Ions score 122](http://mbp-mascot4/mascot/cgi/peptide_view.pl?file=../data/20091021/F006297.dat&query=2342&hit=1&index=IPI00031411&px=1&section=5&ave_thresh=22))

**3491 - 3507 887.4914 1772.9683 1772.9672 1 0 K.AFEVNPQGVLLTSSAIK.R**  ([Ions score 121](http://mbp-mascot4/mascot/cgi/peptide_view.pl?file=../data/20091021/F006297.dat&query=2343&hit=1&index=IPI00031411&px=1&section=5&ave_thresh=22))

**3520 - 3537 664.0252 1989.0538 1989.0531 0 0 K.VADNGKPQLSSLTYIDIR.V**  ([Ions score 33](http://mbp-mascot4/mascot/cgi/peptide_view.pl?file=../data/20091021/F006297.dat&query=2767&hit=1&index=IPI00031411&px=1&section=5&ave_thresh=22))

**3520 - 3537 664.0255 1989.0547 1989.0531 1 0 K.VADNGKPQLSSLTYIDIR.V**  ([Ions score 32](http://mbp-mascot4/mascot/cgi/peptide_view.pl?file=../data/20091021/F006297.dat&query=2768&hit=1&index=IPI00031411&px=1&section=5&ave_thresh=22))

**3570 - 3601 1173.5466 3517.6180 3517.6195 -0 0 K.IHATDQDVYDTLTYSLDPQMDNLFSVSSTGGK.L**  ([Ions score 72](http://mbp-mascot4/mascot/cgi/peptide_view.pl?file=../data/20091021/F006297.dat&query=4043&hit=1&index=IPI00031411&px=1&section=5&ave_thresh=22))

**3570 - 3601 1173.5471 3517.6195 3517.6195 -0 0 K.IHATDQDVYDTLTYSLDPQMDNLFSVSSTGGK.L**  ([Ions score 68](http://mbp-mascot4/mascot/cgi/peptide_view.pl?file=../data/20091021/F006297.dat&query=4044&hit=1&index=IPI00031411&px=1&section=5&ave_thresh=22))

**3570 - 3601 1178.8787 3533.6141 3533.6145 -0 0 K.IHATDQDVYDTLTYSLDPQMDNLFSVSSTGGK.L**  Oxidation (M) ([Ions score 76](http://mbp-mascot4/mascot/cgi/peptide_view.pl?file=../data/20091021/F006297.dat&query=4056&hit=1&index=IPI00031411&px=1&section=5&ave_thresh=22))

**3650 - 3664 922.4354 1842.8562 1842.8577 -1 0 R.FANLTPEEFVGDYWR.N**  ([Ions score 54](http://mbp-mascot4/mascot/cgi/peptide_view.pl?file=../data/20091021/F006297.dat&query=2489&hit=1&index=IPI00031411&px=1&section=5&ave_thresh=22))

**3650 - 3664 922.4363 1842.8580 1842.8577 0 0 R.FANLTPEEFVGDYWR.N**  ([Ions score 70](http://mbp-mascot4/mascot/cgi/peptide_view.pl?file=../data/20091021/F006297.dat&query=2490&hit=1&index=IPI00031411&px=1&section=5&ave_thresh=22))

**3733 - 3739 431.2635 860.5125 860.5120 1 0 R.ILNVFQK.L**  ([Ions score 29](http://mbp-mascot4/mascot/cgi/peptide_view.pl?file=../data/20091021/F006297.dat&query=196&hit=1&index=IPI00031411&px=1&section=5&ave_thresh=22))

**3733 - 3739 431.2638 860.5130 860.5120 1 0 R.ILNVFQK.L**  ([Ions score 32](http://mbp-mascot4/mascot/cgi/peptide_view.pl?file=../data/20091021/F006297.dat&query=197&hit=1&index=IPI00031411&px=1&section=5&ave_thresh=22))

**3740 - 3749 610.2836 1218.5526 1218.5526 0 0 K.LCAGLDCPWK.F**  ([Ions score 46](http://mbp-mascot4/mascot/cgi/peptide_view.pl?file=../data/20091021/F006297.dat&query=1179&hit=1&index=IPI00031411&px=1&section=5&ave_thresh=22))

**3740 - 3749 610.2837 1218.5528 1218.5526 0 0 K.LCAGLDCPWK.F**  ([Ions score 38](http://mbp-mascot4/mascot/cgi/peptide_view.pl?file=../data/20091021/F006297.dat&query=1180&hit=1&index=IPI00031411&px=1&section=5&ave_thresh=22))

**3755 - 3769 535.9230 1604.7473 1604.7464 1 0 K.VSVDESVMSTHSTAR.L**  ([Ions score 46](http://mbp-mascot4/mascot/cgi/peptide_view.pl?file=../data/20091021/F006297.dat&query=2020&hit=1&index=IPI00031411&px=1&section=5&ave_thresh=22))

**3755 - 3769 535.9231 1604.7476 1604.7464 1 0 K.VSVDESVMSTHSTAR.L**  ([Ions score 51](http://mbp-mascot4/mascot/cgi/peptide_view.pl?file=../data/20091021/F006297.dat&query=2021&hit=1&index=IPI00031411&px=1&section=5&ave_thresh=22))

**3770 - 3776 410.2401 818.4656 818.4650 1 0 R.LSFVTPR.H**  ([Ions score 41](http://mbp-mascot4/mascot/cgi/peptide_view.pl?file=../data/20091021/F006297.dat&query=67&hit=1&index=IPI00031411&px=1&section=5&ave_thresh=22))

**3770 - 3776 410.2401 818.4657 818.4650 1 0 R.LSFVTPR.H**  ([Ions score 39](http://mbp-mascot4/mascot/cgi/peptide_view.pl?file=../data/20091021/F006297.dat&query=68&hit=1&index=IPI00031411&px=1&section=5&ave_thresh=22))

**3826 - 3844 1010.9592 2019.9039 2019.9030 0 0 R.FGQCPGSSSMTLTGNSYVK.Y**  ([Ions score 96](http://mbp-mascot4/mascot/cgi/peptide_view.pl?file=../data/20091021/F006297.dat&query=2805&hit=1&index=IPI00031411&px=1&section=5&ave_thresh=22))

**3826 - 3844 1018.9575 2035.9005 2035.8979 1 0 R.FGQCPGSSSMTLTGNSYVK.Y**  Oxidation (M) ([Ions score 97](http://mbp-mascot4/mascot/cgi/peptide_view.pl?file=../data/20091021/F006297.dat&query=2832&hit=1&index=IPI00031411&px=1&section=5&ave_thresh=22))

**3876 - 3888 499.9202 1496.7387 1496.7372 1 0 R.GTDYSILEIHHGR.L**  ([Ions score 50](http://mbp-mascot4/mascot/cgi/peptide_view.pl?file=../data/20091021/F006297.dat&query=1853&hit=1&index=IPI00031411&px=1&section=5&ave_thresh=22))

**3876 - 3888 499.9203 1496.7389 1496.7372 1 0 R.GTDYSILEIHHGR.L**  ([Ions score 44](http://mbp-mascot4/mascot/cgi/peptide_view.pl?file=../data/20091021/F006297.dat&query=1854&hit=1&index=IPI00031411&px=1&section=5&ave_thresh=22))

**3928 - 3945 904.4993 1806.9841 1806.9840 0 0 R.LVLDQVHTASGTAPGTLK.T**  ([Ions score 80](http://mbp-mascot4/mascot/cgi/peptide_view.pl?file=../data/20091021/F006297.dat&query=2404&hit=1&index=IPI00031411&px=1&section=5&ave_thresh=22))

**3928 - 3945 603.3354 1806.9843 1806.9840 0 0 R.LVLDQVHTASGTAPGTLK.T**  ([Ions score 41](http://mbp-mascot4/mascot/cgi/peptide_view.pl?file=../data/20091021/F006297.dat&query=2405&hit=1&index=IPI00031411&px=1&section=5&ave_thresh=22))

**3928 - 3945 904.4996 1806.9846 1806.9840 0 0 R.LVLDQVHTASGTAPGTLK.T**  ([Ions score 76](http://mbp-mascot4/mascot/cgi/peptide_view.pl?file=../data/20091021/F006297.dat&query=2406&hit=1&index=IPI00031411&px=1&section=5&ave_thresh=22))

**3928 - 3945 603.3356 1806.9851 1806.9840 1 0 R.LVLDQVHTASGTAPGTLK.T**  ([Ions score 52](http://mbp-mascot4/mascot/cgi/peptide_view.pl?file=../data/20091021/F006297.dat&query=2407&hit=1&index=IPI00031411&px=1&section=5&ave_thresh=22))

**3946 - 3960 589.3055 1764.8948 1764.8948 0 0 K.TLNLDNYVFFGGHIR.Q**  ([Ions score 51](http://mbp-mascot4/mascot/cgi/peptide_view.pl?file=../data/20091021/F006297.dat&query=2325&hit=1&index=IPI00031411&px=1&section=5&ave_thresh=22))

**3946 - 3960 589.3058 1764.8955 1764.8948 0 0 K.TLNLDNYVFFGGHIR.Q**  ([Ions score 47](http://mbp-mascot4/mascot/cgi/peptide_view.pl?file=../data/20091021/F006297.dat&query=2326&hit=1&index=IPI00031411&px=1&section=5&ave_thresh=22))

**3978 - 3997 770.0405 2307.0996 2307.0987 0 0 R.GCMDSIYLNGQELPLNSKPR.S**  Oxidation (M) ([Ions score 54](http://mbp-mascot4/mascot/cgi/peptide_view.pl?file=../data/20091021/F006297.dat&query=3246&hit=1&index=IPI00031411&px=1&section=5&ave_thresh=22))

**4627 - 4647 719.3361 2154.9863 2154.9852 1 0 K.EDAPVGSLVMTVSAHDEDAGR.D**  ([Ions score 35](http://mbp-mascot4/mascot/cgi/peptide_view.pl?file=../data/20091021/F006297.dat&query=3000&hit=1&index=IPI00031411&px=1&section=5&ave_thresh=22))

**4890 - 4915 930.1016 2787.2830 2787.2835 -0 1 R.VIATDKDEGPNAEISYSIEDGNEHGK.J**  ([Ions score 65](http://mbp-mascot4/mascot/cgi/peptide_view.pl?file=../data/20091021/F006297.dat&query=3652&hit=1&index=IPI00031411&px=1&section=5&ave_thresh=22))

**4890 - 4915 697.8284 2787.2846 2787.2835 0 1 R.VIATDKDEGPNAEISYSIEDGNEHGK.J**  ([Ions score 29](http://mbp-mascot4/mascot/cgi/peptide_view.pl?file=../data/20091021/F006297.dat&query=3653&hit=1&index=IPI00031411&px=1&section=5&ave_thresh=22))

**4890 - 4915 697.8284 2787.2846 2787.2835 0 1 R.VIATDKDEGPNAEISYSIEDGNEHGK.J**  ([Ions score 31](http://mbp-mascot4/mascot/cgi/peptide_view.pl?file=../data/20091021/F006297.dat&query=3654&hit=1&index=IPI00031411&px=1&section=5&ave_thresh=22))

**Panc1 A3**

Match to: **IPI00031411** Score: **1081**

**Gene_Symbol=FAT1 Protocadherin Fat 1 lng=4591 # SP[4593,D,22,D]SNP[4646,g,1064,R]SNP[4677,r,1064,R]SNP[4713,i,1125,I]SNP[4791,l,1125,I]SNP[4844,i,1252,I]SNP[4856,h,1273,H]SNP[4889,r,1273,H]SNP[4931,l,1283,P]SNP[4969,p,1283,P]SNP[5000,l,129,V]SNP[5**

Found in search of C:\mgf\Orbidata\080911_ISW1295_Panc1_A3.mgf

Nominal mass (M_r_): **1068827**; Calculated pI value: **4.55**

NCBI BLAST search of [IPI00031411](http://www.ncbi.nlm.nih.gov/blast/Blast.cgi?ALIGNMENTS=50&ALIGNMENT_VIEW=Pairwise&AUTO_FORMAT=Semiauto&CDD_SEARCH=on&CLIENT=web&COMPOSITION_BASED_STATISTICS=on&DATABASE=nr&DESCRIPTIONS=100&ENTREZ_QUERY=(none)&EXPECT=10&FILTER=L&FORMAT_BLOCK_ON_RESPAGE=None&FORMAT_OBJECT=Alignment&FORMAT_TYPE=HTML&GAPCOSTS=11+1&I_THRESH=0.001&LAYOUT=TwoWindows&MATRIX_NAME=BLOSUM62&NCBI_GI=on&PAGE=Proteins&PROGRAM=blastp&QUERY=IPI00031411&SERVICE=plain&SET_DEFAULTS.x=21&SET_DEFAULTS.y=7&SHOW_OVERVIEW=on&WORD_SIZE=3&END_OF_HTTPGET=Yes) against nr

Unformatted [sequence string](http://mbp-mascot4/mascot/cgi/getseq.pl?IPI_human+IPI00031411+seq) for pasting into other applications

Fixed modifications: Carbamidomethyl (C)

Variable modifications: Oxidation (M)

Cleavage by TrypsinMSIPI, a mixture of enzymes:

cuts C-term side of KR unless next residue is P

cuts C-term side of J

cuts N-term side of J

Sequence Coverage: **3%**

Matched peptides shown in **Bold Red**

**1** MGRHLALLLL LLLLFQHFGD SDGSQRLEQT PLQFTHLEYN VTVQENSAAK

**51** TYVGHPVKMG VYITHPAWEV RYKIVSGDSE NLFK**AEEYIL GDFCFLR**IRT

**101** KGGNTAILNR EVKDHYTLIV KALEKNTNVE ARTKVRVQVL DTNDLRPLFS

**151** PTSYSVSLPE NTAIRTSIAR VSATDADIGT NGEFYYSFKD RTDMFAIHPT

**201** SGVIVLTGRL DYLETKLYEM EILAADRGMK LYGSSGISSM AK**LTVHIEQA**

**251 NECAPVITAV TLSPSELDR**D PAYAIVTVDD CDQGANGDIA SLSIVAGDLL

**301** QQFRTVRSFP GSKEYKVKAI GGIDWDSHPF GYNLTLQAKD KGTPPQFSSV

**351** KVIHVTSPQF KAGPVKFEKD VYRAEISEFA PPNTPVVMVK AIPAYSHLRY

**401** VFKSTPGKAK **FSLNYNTGLI SILEPVKR**QQ AAHFELEVTT SDRKASTKVL

**451** VK**VLGANSNP PEFTQTAYK**A AFDENVPIGT TVMSLSAVDP DEGENGYVTY

**501** SIANLNHVPF AIDHFTGAVS TSENLDYELM PRVYTLRIRA SDWGLPYRRE

**551** VEVLATITLN NLNDNTPLFE KINCEGTIPR DLGVGEQITT VSAIDADELQ

**601** LVQYQIEAGN ELDFFSLNPN SGVLSLKRSL MDGLGAKVSF HSLRITATDG

**651** ENFATPLYIN ITVAASHK**LV NLQCEETGVA K**MLAEKLLQA NKLHNQGEVE

**701** DIFFDSHSVN AHIPQFRSTL PTGIQVKENQ PVGSSVIFMN STDLDTGFNG

**751** KLVYAVSGGN EDSCFMIDME TGMLKILSPL DRETTDKYTL NITVYDLGIP

**801** QKAAWRLLHV VVVDANDNPP EFLQESYFVE VSEDKEVHSE IIQVEATDKD

**851** LGPNGHVTYS IVTDTDTFSI DSVTGVVNIA RPLDRELQHE HSLKIEARDQ

**901** AR**EEPQLFST VVVK**VSLEDV NDNPPTFIPP NYRVKVREDL PEGTVIMWLE

**951** AHDPDLGQSG QVR**YSLLDHG EGNFDVDK**LS GAVRIVQQLD FEKKQVYNLT

**1001** VRAKDKGKPV SLSSTCYVEV EVVDVNENLH PPVFSSFVEK GTVKEDAPVG

**1051** SLVMTVSAHD EDARRDGEIR YSIRDGSGVG VFKIGEETGV IETSDRLDRE

**1101** STSHYWLTVF ATDQGVVPLS SFIEIYIEVE DVNDNAPQTS EPVYYPEIME

**1151** NSPKDVSVVQ IEAFDPDSSS NDKLMYK**ITS GNPQGFFSIH PK**TGLITTTS

**1201** RKLDR**EQQDE HILEVTVTDN GSPPK**STIAR VIVK**ILDEND NKPQFLQK**FY

**1251** KIRLPEREKP DRERNARREP LYHVIATDKD EGPNAEISYS IEDGNEHGKF

**1301** FIEPKTGVVS SKRFSAAGEY DILSIKAVDN GRPQKSSTTR LHIEWISKPK

**1351** PSLEPISFEE SFFTFTVMES DPVAHMIGVI SVEPPGIPLW FDITGGNYDS

**1401** HFDVDKGTGT IIVAKPLDAE QKSNYNLTVE ATDGTTTILT QVFIKVIDTN

**1451** DHRPQFSTSK YEVVIPEDTA PETEILQISA VDQDEKNKLI YTLQSSRDPL

**1501** SLKKFRLDPA TGSLYTSEK**L DHEAVHQHTL TVMVR**DQDVP VKRNFARIVV

**1551** NVSDTNDHAP WFTASSYKGR VYESAAVGSV VLQVTALDKD KGKNAEVLYS

**1601** IESGTFGNIG NSFMIDPVLG SIKTAKELDR SNQAEYDLMV KATDKGSPPM

**1651** SEITSVR**IFV TIADNASPK**F TSKEYSVELS ETVSIGSFVG MVTAHSQSSV

**1701** VYEIK**DGNTG DAFDINPHSG TIITQK**ALDF ETLPIYTLII QGTNMAGLST

**1751** NTTVLVHLQD ENDNAPVFMQ AEYTGLISES ASINSVVLTD RNVPLVIRAA

**1801** DADKDSNALL VYHIVEPSVH TYFAIDSSTG AIHTVLSLDY EETSIFHFTV

**1851** QVHDMGTPRL FAEYAANVTV HVIDINDCPP VFAKPLYEAS LLLPTYKGVK

**1901** VITVNATDAD SSAFSQLIYS ITEGNIGEKF SMDYKTGALT VQNTTQLRSR

**1951** YELTVRASDG RFAGLTSVKI NVKESKESHL K**FTQDVYSAV VK**ENSTEAET

**2001** LAVITAIGNP INEPLFYHIL NPDRRFKISR **TSGVLSTTGT PFDR**EQQEAF

**2051** DVVVEVTEEH KPSAVAHVVV K**VIVEDQNDN APVFVNLPYY AVVK**VDTEVG

**2101** HVIRYVTAVD RDSGRNGEVH YYLK**EHHEHF QIGPLGEISL K**KQFELDTLN

**2151** K**EYLVTVVAK** DGGNPAFSAE VIVPITVMNK AMPVFEKPFY SAEIAESIQV

**2201** HSPVVHVQAN SPEGLKVFYS ITDGDPFSQF TINFNTGVIN VIAPLDFEAH

**2251** PAYKLSIRAT DSLTGAHAEV FVDIIVDDIN DNPPVFAQQS YAVTLSEASV

**2301** IGTSVVQVRA TDSDSEPNRG ISYQMFGNHS K**SHDHFHVDS STGLISLLR**T

**2351** LDYEQSRQHT IFVRAVDGGM PTLSSDVIVT VDVTDLNDNP PLFEQQIYEA

**2401** RISEHAPHGH FVTCVKAYDA DSSDIDKLQY SILSGNDHK**H FVIDSATGII**

**2451 TLSNLHR**HAL KPFYSLNLSV SDGVFRSSTQ VHVTVIGGNL HSPAFLQNEY

**2501** EVELAENAPL HTLVMEVKTT DGDSGIYGHV TYHIVNDFAK DRFYINERGQ

**2551** IFTLEKLDRE TPAEKVISVR LMAKDAGGKV AFCTVNVILT DDNDNAPQFR

**2601** ATKYEVNIGS SAAKGTSVVK VLASDADEGS NADITYAIEA DSESVKENLE

**2651** INKLSGVITT KESLIGLENE FFTFFVRAVD NGSPSK**ESVV LVYVK**ILPPE

**2701** MQLPKFSEPF YTFTVSEDVP IGTEIDLIRA EHSGTVLYSL VKGNTPESNR

**2751** DESFVIDRQS GRLKLEKSLD HETTK**WYQFS ILAR**CTQDDH EMVASVDVSI

**2801** QVKDANDNSP VFESSPYEAF IVENLPGGSR VIQIRASDAD SGTNGQVMYS

**2851** LDQSQSVEVI ESFAINMETG WITTLKELDH EKRDNYQIKV VASDHGEKIQ

**2901** LSSTAIVDVT VTDVNDSPPR FTAEIYKGTV SEDDPQGGVI AILSTTDADS

**2951** EEINRQVTYF ITGGDPLGQF AVETIQNEWK VYVKKPLDRE KRDNYLLTIT

**3001** ATDGTFSSKA IVEVKVLDAN DNSPVCEKTL YSDTIPEDVL PGKLIMQISA

**3051** TDADIR**SNAE ITYTLLGSGA EK**FKLNPDTG ELK**TSTPLDR EEQAVYHLLV**

**3101 R**ATDGGGRFC QASIVLTLED VNDNAPEFSA DPYAITVFEN TEPGTLLTRV

**3151** QATDADAGLN RKILYSLIDS ADGQFSINEL SGIIQLEKPL DRELQAVYTL

**3201** SLKAVDQGLP RRLTATGTVI VSVLDINDNP PVFEYREYGA TVSEDILVGT

**3251** EVLQVYAASR DIEANAEITY SIISGNEHGK FSIDSKTGAV FIIENLDYES

**3301** SHEYYLTVEA TDGGTPSLSD VATVNVNVTD INDNTPVFSQ DTYTTVISED

**3351** AVLEQSVITV MADDADGPSN SHIHYSIIDG NQGSSFTIDP VRGEVKVTKL

**3401** LDRETISGYT LTVQASDNGS PPRVNTTTVN IDVSDVNDNA PVFSRGNYSV

**3451** IIQENKPVGF SVLQLVVTDE DSSHNGPPFF FTIVTGNDEK AFEVNPQGVL

**3501** LTSSAIKRKE KDHYLLQVKV ADNGKPQLSS LTYIDIRVIE ESIYPPAILP

**3551** LEIFITSSGE EYSGGVIGKI HATDQDVYDT LTYSLDPQMD NLFSVSSTGG

**3601** KLIAHKKLDI GQYLLNVSVT DGKFTTVADI TVHIRQVTQE MLNHTIAIRF

**3651** ANLTPEEFVG DYWRNFQRAL RNILGVRRND IQIVSLQSSE PHPHLDVLLF

**3701** VEKPGSAQIS TKQLLHKINS SVTDIEEIIG VRILNVFQKL CAGLDCPWKF

**3751** CDEKVSVDES VMSTHSTARL SFVTPRHHRA AVCLCKEGRC PPVHHGCEDD

**3801** PCPEGSECVS DPWEEKHTCV CPSGRFGQCP GSSSMTLTGN SYVKYRLTEN

**3851** ENKLEMKLTM RLRTYSTHAV VMYARGTDYS ILEIHHGRLQ YKFDCGSGPG

**3901** IVSVQSIQVN DGQWHAVALE VNGNYARLVL DQVHTASGTA PGTLK**TLNLD**

**3951 NYVFFGGHIR** QQGTRHGRSP QVGNGFRGCM DSIYLNGQEL PLNSKPRSYA

**4001** HIEESVDVSP GCFLTATEDC ASNPCQNGGV CNPSPAGGYY CKCSALYIGT

**4051** HCEISVNPCS SKPCLYGGTC VVDNGGFVCQ CRGLYTGQRC QLSPYCKDEP

**4101** CKNGGTCFDS LDGAVCQCDS GFRGERCQSD IDECSGNPCL HGALCENTHG

**4151** SYHCNCSHEY RGRHCEDAAP NQYVSTPWNI GLAEGIGIVV FVAGIFLLVV

**4201** VFVLCRKMIS RKKKHQAEPK DKHLGPATAF LQRPYFDSKL NKNIYSDIPP

**4251** QVPVRPISYT PSIPSDSRNN LDRNSFEGSA IPEHPEFSTF NPESVHGHRK

**4301** AVAVCSVAPN LPPPPPSNSP SDSDSIQKPS WDFDYDTKVV DLDPCLSKKP

**4351** LEEKPSQPYS ARESLSEVQS LSSFQSESCD DNGYHWDTSD WMPSVPLPDI

**4401** QEFPNYEVID EQTPLYSADP NAIDTDYYPG GYDIESDFPP PPEDFPAADE

**4451** LPPLPPEFSN QFESIHPPRD MPAAGSLGSS SRNRQRFNLN QYLPNFYPLD

**4501** MSEPQTKGTG ENSTCREPHA PYPPGYQRHF EAPAVESMPM SVYASTASCS

**4551** DVSACCEVES EVMMSDYESG DDGHFEEVTI PPLDSQQHTE VJDGSQRLEQ

**4601** TPLQFTHLEY NVTVQENSAA KJGTVKEDAP VGSLVMTVSA HDEDAGRDGE

**4651** IRJGTVKEDA PVGSLVMTVS AHDEDARRDG EIRJLDREST SHYWLTVFAT

**4701** DQGVVPLSSF IEIYIEVEDV NDNAPQTSEP VYYPEIMENS PKDVSVVQIE

**4751** AFDPDSSSND KJLDRESTSH YWLTVFATDQ GVVPLSSFIE LYIEVEDVND

**4801** NAPQTSEPVY YPEIMENSPK DVSVVQIEAF DPDSSSNDKJ FYKIRLPERJ

**4851** REPLYHVIAT DKDEGPNAEI SYSIEDGNEH GKJREPLYRV IATDKDEGPN

**4901** AEISYSIEDG NEHGKJEPLY HVIATDKDEG LNAEISYSIE DGNEHGKFFI

**4951** EPKJEPLYHV IATDKDEGPN AEISYSIEDG NEHGKFFIEP KJALEKNTNL

**5001** EARTKJALEK NTNVEARTKJ ALEKNTNVEA RTKJALEKNT NVEVRTKJFS

**5051** AAGEYDILSI KAVDNGRPQK SSTTRJFSAA GEYDILSIKA VDSGRPQKSS

**5101** TTRJNFARIV VNVSDTNDHA PWFTASSYKG RJNFARIVVN VSDTNDHAPW

**5151** FTTSSYKGRJ GKNAEVLYSI ESGDFGNIGN SFMIDPVLGS IKTAKJGKNA

**5201** EVLYSIESGN FGNIGNSFMI DPVLGSIKTA KJGKNAEVLY SIESGTIGNI

**5251** GNSFMIDPVL GSIKTAKJGS PPMSEITSVR IFVTNADNAS PKFTSKJGSP

**5301** PMSEITSVRI FVTSADNASP KFTSKJGSPP MSEITSVRIF VTIADNASTK

**5351** FTSKJALDFE TLPIYTLIIQ GTNMAGLSTN TTVLVHLQDE NDNAPVFMQA

**5401** EYTGLISESA SINSVVLTDR NVPQVIRAAD ADKJALDFET LPIYTLIIQG

**5451** TNMAGLSTNT TVLVHLQDEN DNAPVFMQAE YTGLISESAS INSVVLTDRN

**5501** VPRVIRAADA DKJAADADKD SNVLLVYHIV EPSVHTYFAI DSSTGAIHTV

**5551** LSLDYEETSI FHFTVQVHDM GTPRLFAEYA ANVTVHVIDI NDCPPVFAKP

**5601** LYEASLLLPT YKJFTQDVYS AVVKTNSTEA ETLAVITAIG NPINEPLFYH

**5651** ILNPDRRJTS GVLSTTGTPF DREAQEAFDV VVEVTEEHKP SAVAHVVVKV

**5701** IVEDQNDNAP VFVNLPYYAV VKJTDMFAIH PTSGVIVLTG RLDFLETKLY

**5751** EMEILAADRJ TDMFAIHPTS GVIVLTGRLD YLETKLYEME ILAADRJNGE

**5801** VHYYLKEHHE HFQIGPLGEK SLKKJDGGNP AFSAEVIVPI TVMNKAMPVF

**5851** EKPFYSAEIA ESIQVHSHVV HVQANSPEGL KVFYSITDGD PFSQFTINFN

**5901** TGVINVIAPL DFEAHPAYKJ AMPVFEKPFY SAEIAESIQV HSPVVHVQAN

**5951** SPEGLKVFYS ITDGDPFSQF TINFNTGVIN VIAPLDFPAH PAYKLSIRJL

**6001** SIRATDSLTG AHAEVFVDDI VDDINDNPPV FAQQSYAVTL SEASVIGTSV

**6051** VQVRATDSDS EPNRJLSIRA TDSLTGAHAE VFVDEIVDDI NDNPPVFAQQ

**6101** SYAVTLSEAS VIGTSVVQVR ATDSDSEPNR JTLDYEQSRQ HTIAVRAVDG

**6151** GMPTLSSDVI VTVDVTDLND NPPLFEQQIY EARJLYGSSG ISSMAKLTVH

**6201** IEQANECAPV ITAVTLSPSE LDRDPAYAIV TVDDCDQGAN GDIASLSIVA

**6251** GDLLQQFRJI LPPEMQLPKF SEPFYTFTVS EIVPIGTEID LIRAEHSGTV

**6301** LYSLVKJILP PEMQLPKFSE PFYTFTVSEV VPIGTEIDLI RAEHSGTVLY

**6351** SLVKJCTQDD HEMVASVDVS IQVKDANDNS PVFESSPYEA FIVENLPGGS

**6401** RVIQIRJCTQ DDHEMVASVD VSIQVKDASD NSPVFESSPY EAFIVENLPG

**6451** GSRVIQIRJV IQIRASDADN GTNGQVMYSL DQSQSVEVIE SFAINMETGW

**6501** ITTLKELDHE KJFTAEIYKG TVSELDPQGG VIAILSTTDA DSEEINRQVT

**6551** YFITGGDPLG QFAVETIQNE WKJFTAEIYK GTVSEPDPQG GVIAILSTTD

**6601** ADSEEINRQV TYFITGGDPL GQFAVETIQN EWKJFTAEIY KGTVSEQDPQ

**6651** GGVIAILSTT DADSEEINRQ VTYFITGGDP LGQFAVETIQ NEWKJGTVSE

**6701** DDPQGGVIAI LSTTDADSEE INRQVTYGIT GGDPLGQFAV ETIQNEWKVY

**6751** VKJGTVSEDD PQGGVIAILS TTDADSEEIN RQVTYSITGG DPLGQFAVET

**6801** IQNEWKVYVK JGTVSEDDPQ GGVIAILSTT DADSEEINRQ VTYFITGGDP

**6851** LAQFAVETIQ NEWKVYVKJA IVEVKVLDAN DNSPVCEYTL YSDTIPEDVL

**6901** PGKLIMQISA TDADIRJVLD ANDNSPVCEK TLYIDTIPED VLPGKLIMQI

**6951** SATDADIRJV LDANDNSPVC EKTLYTDTIP EDVLPGKLIM QISATDADIR

**7001** JATDGGGRFC QASIVLDLED VNDNAPEFSA DPYAITVFEN TEPGTLLTRV

**7051** QATDADAGLN RJKILYSLID SADGQFSINE LIGIIQLEKP LDRELQAVYT

**7101** LSLKJFSIDS KTGAVFIIEN LDYESSHEYY LTVEATDGGT PSLSDVATVN

**7151** VNVTDINDAT PVFSQDTYTT VISEDAVLEQ SVITVMADDA DGPSNSHIHY

**7201** SIIDGNQGSS FTIDPVRGEV KJFSIDSKTG AVFIIENLDY ESSHEYYLTV

**7251** EATDGGTPSL SDVATVNVNV TDINDVTPVF SQDTYTTVIS EDAVLEQSVI

**7301** TVMADDADGP SNSHIHYSII DGNQGSSFTI DPVRGEVKJV NTTTVNIDVS

**7351** DVNDNAPVFS RGNYSVIIQE NKPVGFSVLQ LVVTDEDSSH NGPPFFFTIV

**7401** TENDEKAFEV NPQGVLLTSS AIKJVNTTTV NIDVSDVNDN APVFSRGNYS

**7451** VIIQENKPVG FSVLQLVVTD EDSSHNGPPF FFTIVTGNDE KAFEVNPQGV

**7501** LLTSSAIKJG NYSVIIQENK PVGFSVLQLV VTDEDSSHNG PPFFFTIVTG

**7551** NDEKAFEVNA QGVLLTSSAI KRJGNYSVII QENKPVGFSV LQLVVTDEDS

**7601** SHNGPPFFFT IVTGNDEKAF EVNVQGVLLT SSAIKRJVAD NGKPQLSSLT

**7651** YIDIRVIEES IYPPAILPLE IAITSSGEEY SGGVIGKIHA TDQDVYDTLT

**7701** YSLDPQMDNL FSVSSTGGKJ VADNGKPQLS SLTYIDIRVI EESIYPPAIL

**7751** PLEISITSSG EEYSGGVIGK IHATDQDVYD TLTYSLDPQM DNLFSVSSTG

**7801** GKJVIHVTSP QFKAGPVKFE KJQLLHKIMS SVTDIEEIIG VRILNVFQKJ

**7851** QLLHKIVSSV TDIEEIIGVR ILNVFQKJEG RCPPVHHGCE DHPCPEGSEC

**7901** VSDPWEEKHT CVCPSGRJEG RCPPVHHGCE DPPCPEGSEC VSDPWEEKHT

**7951** CVCPSGRJHT CVCPSGRFGG CPGSSSMTLT GNSYVKYRJH TCVCPSGRFG

**8001** RCPGSSSMTL TGNSYVKYRJ FGQCPGSSSM TLTGNSYVKT RLTENENKJG

**8051** CMDSIYLNGQ ELPLNSKPRS YAHIEESVPV SPGCFLTATE DCASNPCQNG

**8101** GVCNPSPAGG YYCKCSALYI GTHCEISVNP CSSKPCLYGG TCVVDNGGFV

**8151** CQCRJYVFKR TPGKAKJYVF KSTPGKAKJS YAHIEESVDV SPGCFLTATE

**8201** DCASNPCQNG GVCNPSPAGG YYCKCSALYI GTHCEISVNP KSSKPCLYGG

**8251** TCVVDNGGFV CQCRGLYTGQ RJSYAHIEES VDVSPGCFLT ATEDCASNPC

**8301** QNGGVCNPSP AGGYYCKCSA LYIGTHCEIS VNPNSSKPCL YGGTCVVDNG

**8351** GFVCQCRGLY TGQRJDEPCK NGGTCFDSAD GAVCQCDSGF RGERJGRHCA

**8401** DAAPNQYVST PWNIGLAEGI GIVVFVAGIF LLVVVFVLCR KJGRHCEDAA

**8451** PNQYVSTPWN IGLAEGIGIV VFVAGIFLLV VVFVLCRKJN IYSDIPPQVP

**8501** VRYISYTPSI PSDSRNNLDR JKAVAVCSVA PNLPPPPPSN SPSDSDSIQK

**8551** PSWDFDYDTK VVDLDPCLSK JKPLEEKPSQ PYSARESLSS VQSLSSFQSE

**8601** SCDDNGYHWD TSDWMPSVPL PDIQEFPNYE VIDEQTPLYS ADPNAIDTDY

**8651** YPGGYDIESD FPPPPEDFPA ADELPPLPPE FSNQFESIHP PRDMPAAGSL

**8701** GSSSRJKPLE EKPSQPYSAR ESLSEVQSLS SFQSESCDDN GYHWDTSDWM

**8751** PSVPLQDIQE FPNYEVIDEQ TPLYSADPNA IDTDYYPGGY DIESDFPPPP

**8801** EDFPAADELP PLPPEFSNQF ESIHPPRDMP AAGSLGSSSR JKPLEEKPSQ

**8851** PYSARESLSE VQSLSSFQSE SCDDNGYHWD TSDWMPSVPL PDIQEFPNYE

**8901** VIDPQTPLYS ADPNAIDTDY YPGGYDIESD FPPPPEDFPA ADELPPLPPE

**8951** FSNQFESIHP PRDMPAAGSL GSSSRJESLS EVQSLSSFQS ESCDDNGYHW

**9001** DTSDWMPSVP LPDIQEFPNY EVIDEQTPLY SADPNAIDTD YYPGGYDIES

**9051** DFPPPPEDFP AADELPPLPP EFSNQFESIH PPRDMPAAGS LGSSRRNRJE

**9101** SLSEVQSLSS FQSESCDDNG YHWDTSDWMP SVPLPDIQEF PNYEVIDEQT

**9151** PLYSADPNAI DTDYYPGGYD IESDFPPPPE DFPAADELPP LPPEFSNQFE

**9201** SIHPPRDMPA AGSLGSSWRN RJVLGANSNP PEFTQTAYKA AFDENVPIGT

**9251** TIMSLSAVDP DEGENGYVTY SIANLNHVPF AIDHFTGAVS TSENLDYELM

**9301** PRVYTLRJVL GANSNPPEFT QTAYKAAFDE NVPIGTTVMS LSAVDPDEGE

**9351** NGYVTYSIAN LNHVPFAIDH FTGAVSTSEN LDYELMPRVY TLRJVLGANS

**9401** NPPEFTQTAY KAAFDENVPI GTTVMSLSAV DPDEGENGYV TYSIANLNHV

**9451** PFAIDHFTGA VSTSENLDYE LMPRVYTLRJ INCEGTIPRD LGVGEQITTV

**9501** SAIDADELQL VQYQIEAGNE LDFFSLNPNS GVLSLKRJIN CEGTIPRDLG

**9551** VGEQITTVSA IDADELQLVQ YQIEAGNELD LFSLNPNSGV LSLKRJINCE

**9601** GTIPRDLGVG EQITTVSAID ADELQLVQYQ IEAGNELDFF SLNPNSGVLS

**9651** LKRJEVHSEI IQVEATDKDL GPNGHVTYSI LTDTDTFSID SVTGVVNIAR

**9701** PLDRELQHEH SLKJEVHSEI IQVEATDKDL GPNGHVTYSI VTDTDTFSID

**9751** SVTGVVNIAR PLDRELQHEH SLK

**Start - End Observed Mr(expt) Mr(calc) ppm Miss Sequence**

**85 - 97 816.8899 1631.7653 1631.7654 -0 0 K.AEEYILGDFCFLR.I**  ([Ions score 53](http://mbp-mascot4/mascot/cgi/peptide_view.pl?file=../data/20091023/F006439.dat&query=2356&hit=1&index=IPI00031411&px=1&section=5&ave_thresh=22))

**85 - 97 816.8901 1631.7657 1631.7654 0 0 K.AEEYILGDFCFLR.I**  ([Ions score 68](http://mbp-mascot4/mascot/cgi/peptide_view.pl?file=../data/20091023/F006439.dat&query=2357&hit=1&index=IPI00031411&px=1&section=5&ave_thresh=22))

**243 - 269 988.5097 2962.5073 2962.5070 0 0 K.LTVHIEQANECAPVITAVTLSPSELDR.D**  ([Ions score 25](http://mbp-mascot4/mascot/cgi/peptide_view.pl?file=../data/20091023/F006439.dat&query=3849&hit=1&index=IPI00031411&px=1&section=5&ave_thresh=22))

**411 - 428 688.7206 2063.1401 2063.1415 -1 1 K.FSLNYNTGLISILEPVKR.Q**  ([Ions score 34](http://mbp-mascot4/mascot/cgi/peptide_view.pl?file=../data/20091023/F006439.dat&query=3077&hit=1&index=IPI00031411&px=1&section=5&ave_thresh=22))

**411 - 428 688.7207 2063.1403 2063.1415 -1 1 K.FSLNYNTGLISILEPVKR.Q**  ([Ions score 32](http://mbp-mascot4/mascot/cgi/peptide_view.pl?file=../data/20091023/F006439.dat&query=3078&hit=1&index=IPI00031411&px=1&section=5&ave_thresh=22))

**453 - 469 918.9601 1835.9057 1835.9054 0 0 K.VLGANSNPPEFTQTAYK.A**  ([Ions score 62](http://mbp-mascot4/mascot/cgi/peptide_view.pl?file=../data/20091023/F006439.dat&query=2732&hit=1&index=IPI00031411&px=1&section=5&ave_thresh=22))

**669 - 681 730.8753 1459.7360 1459.7341 1 0 K.LVNLQCEETGVAK.M**  ([Ions score 77](http://mbp-mascot4/mascot/cgi/peptide_view.pl?file=../data/20091023/F006439.dat&query=2032&hit=1&index=IPI00031411&px=1&section=5&ave_thresh=22))

**903 - 914 688.3770 1374.7395 1374.7395 -0 0 R.EEPQLFSTVVVK.V**  ([Ions score 49](http://mbp-mascot4/mascot/cgi/peptide_view.pl?file=../data/20091023/F006439.dat&query=1864&hit=1&index=IPI00031411&px=1&section=5&ave_thresh=22))

**964 - 978 570.2654 1707.7745 1707.7740 0 0 R.YSLLDHGEGNFDVDK.L**  ([Ions score 39](http://mbp-mascot4/mascot/cgi/peptide_view.pl?file=../data/20091023/F006439.dat&query=2492&hit=1&index=IPI00031411&px=1&section=5&ave_thresh=22))

**964 - 978 570.2655 1707.7747 1707.7740 0 0 R.YSLLDHGEGNFDVDK.L**  ([Ions score 40](http://mbp-mascot4/mascot/cgi/peptide_view.pl?file=../data/20091023/F006439.dat&query=2493&hit=1&index=IPI00031411&px=1&section=5&ave_thresh=22))

**1178 - 1192 543.9511 1628.8315 1628.8311 0 0 K.ITSGNPQGFFSIHPK.T**  ([Ions score 26](http://mbp-mascot4/mascot/cgi/peptide_view.pl?file=../data/20091023/F006439.dat&query=2350&hit=1&index=IPI00031411&px=1&section=5&ave_thresh=22))

**1206 - 1225 746.0295 2235.0668 2235.0655 1 0 R.EQQDEHILEVTVTDNGSPPK.S**  ([Ions score 24](http://mbp-mascot4/mascot/cgi/peptide_view.pl?file=../data/20091023/F006439.dat&query=3287&hit=1&index=IPI00031411&px=1&section=5&ave_thresh=22))

**1235 - 1248 567.9652 1700.8738 1700.8733 0 0 K.ILDENDNKPQFLQK.F**  ([Ions score 30](http://mbp-mascot4/mascot/cgi/peptide_view.pl?file=../data/20091023/F006439.dat&query=2477&hit=1&index=IPI00031411&px=1&section=5&ave_thresh=22))

**1235 - 1248 567.9653 1700.8740 1700.8733 0 0 K.ILDENDNKPQFLQK.F**  ([Ions score 40](http://mbp-mascot4/mascot/cgi/peptide_view.pl?file=../data/20091023/F006439.dat&query=2478&hit=1&index=IPI00031411&px=1&section=5&ave_thresh=22))

**1520 - 1535 472.2483 1884.9640 1884.9629 1 0 K.LDHEAVHQHTLTVMVR.D**  ([Ions score 23](http://mbp-mascot4/mascot/cgi/peptide_view.pl?file=../data/20091023/F006439.dat&query=2824&hit=1&index=IPI00031411&px=1&section=5&ave_thresh=22))

**1658 - 1669 638.3507 1274.6869 1274.6870 -0 0 R.IFVTIADNASPK.F**  ([Ions score 62](http://mbp-mascot4/mascot/cgi/peptide_view.pl?file=../data/20091023/F006439.dat&query=1539&hit=1&index=IPI00031411&px=1&section=5&ave_thresh=22))

**1658 - 1669 638.3510 1274.6875 1274.6870 0 0 R.IFVTIADNASPK.F**  ([Ions score 50](http://mbp-mascot4/mascot/cgi/peptide_view.pl?file=../data/20091023/F006439.dat&query=1540&hit=1&index=IPI00031411&px=1&section=5&ave_thresh=22))

**1706 - 1726 734.3541 2200.0404 2200.0397 0 0 K.DGNTGDAFDINPHSGTIITQK.A**  ([Ions score 50](http://mbp-mascot4/mascot/cgi/peptide_view.pl?file=../data/20091023/F006439.dat&query=3224&hit=1&index=IPI00031411&px=1&section=5&ave_thresh=22))

**1982 - 1992 628.8294 1255.6442 1255.6449 -0 0 K.FTQDVYSAVVK.E**  ([Ions score 62](http://mbp-mascot4/mascot/cgi/peptide_view.pl?file=../data/20091023/F006439.dat&query=1489&hit=1&index=IPI00031411&px=1&section=5&ave_thresh=22))

**1982 - 1992 628.8297 1255.6449 1255.6449 -0 0 K.FTQDVYSAVVK.E**  ([Ions score 82](http://mbp-mascot4/mascot/cgi/peptide_view.pl?file=../data/20091023/F006439.dat&query=1490&hit=1&index=IPI00031411&px=1&section=5&ave_thresh=22))

**2031 - 2044 719.8621 1437.7096 1437.7100 -0 0 R.TSGVLSTTGTPFDR.E**  ([Ions score 65](http://mbp-mascot4/mascot/cgi/peptide_view.pl?file=../data/20091023/F006439.dat&query=1992&hit=1&index=IPI00031411&px=1&section=5&ave_thresh=22))

**2031 - 2044 719.8623 1437.7100 1437.7100 0 0 R.TSGVLSTTGTPFDR.E**  ([Ions score 70](http://mbp-mascot4/mascot/cgi/peptide_view.pl?file=../data/20091023/F006439.dat&query=1993&hit=1&index=IPI00031411&px=1&section=5&ave_thresh=22))

**2072 - 2094 869.4543 2605.3412 2605.3428 -1 0 K.VIVEDQNDNAPVFVNLPYYAVVK.V**  ([Ions score 42](http://mbp-mascot4/mascot/cgi/peptide_view.pl?file=../data/20091023/F006439.dat&query=3655&hit=1&index=IPI00031411&px=1&section=5&ave_thresh=22))

**2072 - 2094 869.4552 2605.3438 2605.3428 0 0 K.VIVEDQNDNAPVFVNLPYYAVVK.V**  ([Ions score 44](http://mbp-mascot4/mascot/cgi/peptide_view.pl?file=../data/20091023/F006439.dat&query=3656&hit=1&index=IPI00031411&px=1&section=5&ave_thresh=22))

**2125 - 2141 493.5081 1970.0033 1970.0010 1 0 K.EHHEHFQIGPLGEISLK.K**  ([Ions score 30](http://mbp-mascot4/mascot/cgi/peptide_view.pl?file=../data/20091023/F006439.dat&query=2961&hit=1&index=IPI00031411&px=1&section=5&ave_thresh=22))

**2152 - 2160 511.3000 1020.5854 1020.5855 -0 0 K.EYLVTVVAK.D**  ([Ions score 33](http://mbp-mascot4/mascot/cgi/peptide_view.pl?file=../data/20091023/F006439.dat&query=776&hit=1&index=IPI00031411&px=1&section=5&ave_thresh=22))

**2152 - 2160 511.3001 1020.5856 1020.5855 0 0 K.EYLVTVVAK.D**  ([Ions score 47](http://mbp-mascot4/mascot/cgi/peptide_view.pl?file=../data/20091023/F006439.dat&query=777&hit=1&index=IPI00031411&px=1&section=5&ave_thresh=22))

**2332 - 2349 506.0106 2020.0132 2020.0126 0 0 K.SHDHFHVDSSTGLISLLR.T**  ([Ions score 41](http://mbp-mascot4/mascot/cgi/peptide_view.pl?file=../data/20091023/F006439.dat&query=3021&hit=1&index=IPI00031411&px=1&section=5&ave_thresh=22))

**2332 - 2349 506.0113 2020.0162 2020.0126 2 0 K.SHDHFHVDSSTGLISLLR.T**  ([Ions score 29](http://mbp-mascot4/mascot/cgi/peptide_view.pl?file=../data/20091023/F006439.dat&query=3022&hit=1&index=IPI00031411&px=1&section=5&ave_thresh=22))

**2440 - 2457 665.3655 1993.0746 1993.0745 0 0 K.HFVIDSATGIITLSNLHR.H**  ([Ions score 84](http://mbp-mascot4/mascot/cgi/peptide_view.pl?file=../data/20091023/F006439.dat&query=2995&hit=1&index=IPI00031411&px=1&section=5&ave_thresh=22))

**2440 - 2457 665.3658 1993.0755 1993.0745 0 0 K.HFVIDSATGIITLSNLHR.H**  ([Ions score 94](http://mbp-mascot4/mascot/cgi/peptide_view.pl?file=../data/20091023/F006439.dat&query=2996&hit=1&index=IPI00031411&px=1&section=5&ave_thresh=22))

**2687 - 2695 518.3080 1034.6014 1034.6012 0 0 K.ESVVLVYVK.I**  ([Ions score 34](http://mbp-mascot4/mascot/cgi/peptide_view.pl?file=../data/20091023/F006439.dat&query=802&hit=1&index=IPI00031411&px=1&section=5&ave_thresh=22))

**2776 - 2784 592.3171 1182.6197 1182.6186 1 0 K.WYQFSILAR.C**  ([Ions score 44](http://mbp-mascot4/mascot/cgi/peptide_view.pl?file=../data/20091023/F006439.dat&query=1260&hit=1&index=IPI00031411&px=1&section=5&ave_thresh=22))

**2776 - 2784 592.3176 1182.6206 1182.6186 2 0 K.WYQFSILAR.C**  ([Ions score 57](http://mbp-mascot4/mascot/cgi/peptide_view.pl?file=../data/20091023/F006439.dat&query=1261&hit=1&index=IPI00031411&px=1&section=5&ave_thresh=22))

**3057 - 3072 827.4202 1652.8258 1652.8257 0 0 R.SNAEITYTLLGSGAEK.F**  ([Ions score 98](http://mbp-mascot4/mascot/cgi/peptide_view.pl?file=../data/20091023/F006439.dat&query=2403&hit=1&index=IPI00031411&px=1&section=5&ave_thresh=22))

**3084 - 3101 709.7115 2126.1128 2126.1120 0 1 K.TSTPLDREEQAVYHLLVR.A**  ([Ions score 29](http://mbp-mascot4/mascot/cgi/peptide_view.pl?file=../data/20091023/F006439.dat&query=3123&hit=1&index=IPI00031411&px=1&section=5&ave_thresh=22))

**3084 - 3101 709.7117 2126.1132 2126.1120 1 1 K.TSTPLDREEQAVYHLLVR.A**  ([Ions score 45](http://mbp-mascot4/mascot/cgi/peptide_view.pl?file=../data/20091023/F006439.dat&query=3124&hit=1&index=IPI00031411&px=1&section=5&ave_thresh=22))

**3946 - 3960 589.3058 1764.8957 1764.8948 1 0 K.TLNLDNYVFFGGHIR.Q**  ([Ions score 43](http://mbp-mascot4/mascot/cgi/peptide_view.pl?file=../data/20091023/F006439.dat&query=2594&hit=1&index=IPI00031411&px=1&section=5&ave_thresh=22))

**3946 - 3960 589.3058 1764.8957 1764.8948 1 0 K.TLNLDNYVFFGGHIR.Q**  ([Ions score 26](http://mbp-mascot4/mascot/cgi/peptide_view.pl?file=../data/20091023/F006439.dat&query=2595&hit=1&index=IPI00031411&px=1&section=5&ave_thresh=22))

**PaCa44 A2**

Formularbeginn

Match to: **IPI00031411** Score: **2321**

**Gene_Symbol=FAT1 Protocadherin Fat 1 lng=4591 # SP[4593,D,22,D]SNP[4646,g,1064,R]SNP[4677,r,1064,R]SNP[4713,i,1125,I]SNP[4791,l,1125,I]SNP[4844,i,1252,I]SNP[4856,h,1273,H]SNP[4889,r,1273,H]SNP[4931,l,1283,P]SNP[4969,p,1283,P]SNP[5000,l,129,V]SNP[5**

Found in search of C:\mgf\Orbidata\081103_ISW1295_**Paca44_A2.**mgf

Nominal mass (M_r_): **1068827**; Calculated pI value: **4.55**

NCBI BLAST search of [IPI00031411](http://www.ncbi.nlm.nih.gov/blast/Blast.cgi?ALIGNMENTS=50&ALIGNMENT_VIEW=Pairwise&AUTO_FORMAT=Semiauto&CDD_SEARCH=on&CLIENT=web&COMPOSITION_BASED_STATISTICS=on&DATABASE=nr&DESCRIPTIONS=100&ENTREZ_QUERY=(none)&EXPECT=10&FILTER=L&FORMAT_BLOCK_ON_RESPAGE=None&FORMAT_OBJECT=Alignment&FORMAT_TYPE=HTML&GAPCOSTS=11+1&I_THRESH=0.001&LAYOUT=TwoWindows&MATRIX_NAME=BLOSUM62&NCBI_GI=on&PAGE=Proteins&PROGRAM=blastp&QUERY=IPI00031411&SERVICE=plain&SET_DEFAULTS.x=21&SET_DEFAULTS.y=7&SHOW_OVERVIEW=on&WORD_SIZE=3&END_OF_HTTPGET=Yes) against nr

Unformatted [sequence string](http://mbp-mascot4/mascot/cgi/getseq.pl?IPI_human+IPI00031411+seq) for pasting into other applications

Fixed modifications: Carbamidomethyl (C)

Variable modifications: Oxidation (M)

Cleavage by TrypsinMSIPI, a mixture of enzymes:

cuts C-term side of KR unless next residue is P

cuts C-term side of J

cuts N-term side of J

Sequence Coverage: **9%**

Matched peptides shown in **Bold Red**

**1** MGRHLALLLL LLLLFQHFGD SDGSQRLEQT PLQFTHLEYN VTVQENSAAK

**51** TYVGHPVKMG VYITHPAWEV RYKIVSGDSE NLFK**AEEYIL GDFCFLR**IRT

**101** KGGNTAILNR EVK**DHYTLIV K**ALEKNTNVE ARTKVR**VQVL DTNDLRPLFS**

**151 PTSYSVSLPE NTAIR**TSIAR VSATDADIGT NGEFYYSFKD R**TDMFAIHPT**

**201 SGVIVLTGR**L DYLETK**LYEM EILAADR**GMK **LYGSSGISSM AKLTVHIEQA**

**251 NECAPVITAV TLSPSELDR**D PAYAIVTVDD CDQGANGDIA SLSIVAGDLL

**301** QQFRTVRSFP GSKEYKVKAI GGIDWDSHPF GYNLTLQAKD KGTPPQFSSV

**351** K**VIHVTSPQF K**AGPVKFEKD VYR**AEISEFA PPNTPVVMVK** AIPAYSHLRY

**401** VFKSTPGKAK **FSLNYNTGLI SILEPVK**RQQ AAHFELEVTT SDRKASTKVL

**451** VK**VLGANSNP PEFTQTAYK**A AFDENVPIGT TVMSLSAVDP DEGENGYVTY

**501** SIANLNHVPF AIDHFTGAVS TSENLDYELM PRVYTLRIR**A SDWGLPYR**RE

**551** VEVLATITLN NLNDNTPLFE KINCEGTIPR DLGVGEQITT VSAIDADELQ

**601** LVQYQIEAGN ELDFFSLNPN SGVLSLKRSL MDGLGAKVSF HSLRITATDG

**651** ENFATPLYIN ITVAASHK**LV NLQCEETGVA K**MLAEKLLQA NK**LHNQGEVE**

**701 DIFFDSHSVN AHIPQFRSTL PTGIQVK**ENQ PVGSSVIFMN STDLDTGFNG

**751** KLVYAVSGGN EDSCFMIDME TGMLK**ILSPL DR**ETTDKYTL NITVYDLGIP

**801** QKAAWRLLHV VVVDANDNPP EFLQESYFVE VSEDKEVHSE IIQVEATDKD

**851** LGPNGHVTYS IVTDTDTFSI DSVTGVVNIA RPLDRELQHE HSLKIEARDQ

**901** AREEPQLFST VVVK**VSLEDV NDNPPTFIPP NYR**VKVREDL PEGTVIMWLE

**951** AHDPDLGQSG QVRYSLLDHG EGNFDVDKLS GAVRIVQQLD FEKKQVYNLT

**1001** VRAKDKGKPV SLSSTCYVEV EVVDVNENLH PPVFSSFVEK GTVKEDAPVG

**1051** SLVMTVSAHD EDARRDGEIR YSIRDGSGVG VFK**IGEETGV IETSDR**LDRE

**1101** STSHYWLTVF ATDQGVVPLS SFIEIYIEVE DVNDNAPQTS EPVYYPEIME

**1151** NSPKDVSVVQ IEAFDPDSSS NDKLMYK**ITS GNPQGFFSIH PK**TGLITTTS

**1201** RKLDREQQDE HILEVTVTDN GSPPKSTIAR VIVK**ILDEND NKPQFLQK**FY

**1251** KIRLPEREKP DRERNARREP LYHVIATDKD EGPNAEISYS IEDGNEHGKF

**1301** FIEPKTGVVS SKR**FSAAGEY DILSIK**AVDN GRPQKSSTTR LHIEWISKPK

**1351** PSLEPISFEE SFFTFTVMES DPVAHMIGVI SVEPPGIPLW FDITGGNYDS

**1401** HFDVDK**GTGT IIVAKPLDAE QK**SNYNLTVE ATDGTTTILT QVFIKVIDTN

**1451** DHRPQFSTSK YEVVIPEDTA PETEILQISA VDQDEKNK**LI YTLQSSR**DPL

**1501** SLKKFR**LDPA TGSLYTSEKL DHEAVHQHTL TVMVR**DQDVP VKRNFARIVV

**1551** NVSDTNDHAP WFTASSYKGR VYESAAVGSV VLQVTALDKD KGKNAEVLYS

**1601** IESGTFGNIG NSFMIDPVLG SIKTAKELDR **SNQAEYDLMV K**ATDK**GSPPM**

**1651 SEITSVRIFV TIADNASPK**F TSKEYSVELS ETVSIGSFVG MVTAHSQSSV

**1701** VYEIK**DGNTG DAFDINPHSG TIITQK**ALDF ETLPIYTLII QGTNMAGLST

**1751** NTTVLVHLQD ENDNAPVFMQ AEYTGLISES ASINSVVLTD RNVPLVIRAA

**1801** DADKDSNALL VYHIVEPSVH TYFAIDSSTG AIHTVLSLDY EETSIFHFTV

**1851** QVHDMGTPRL FAEYAANVTV HVIDINDCPP VFAKPLYEAS LLLPTYKGVK

**1901** VITVNATDAD SSAFSQLIYS ITEGNIGEKF SMDYKTGALT VQNTTQLRSR

**1951** YELTVRASDG R**FAGLTSVK**I NVKESKESHL K**FTQDVYSAV VK**ENSTEAET

**2001** LAVITAIGNP INEPLFYHIL NPDRRFKISR **TSGVLSTTGT PFDR**EQQEAF

**2051** DVVVEVTEEH KPSAVAHVVV K**VIVEDQNDN APVFVNLPYY AVVKVDTEVG**

**2101 HVIR**YVTAVD RDSGRNGEVH YYLKEHHEHF QIGPLGEISL KKQFELDTLN

**2151** KEYLVTVVAK **DGGNPAFSAE VIVPITVMNK** AMPVFEKPFY SAEIAESIQV

**2201** HSPVVHVQAN SPEGLKVFYS ITDGDPFSQF TINFNTGVIN VIAPLDFEAH

**2251** PAYKLSIRAT DSLTGAHAEV FVDIIVDDIN DNPPVFAQQS YAVTLSEASV

**2301** IGTSVVQVRA TDSDSEPNRG ISYQMFGNHS KSHDHFHVDS STGLISLLRT

**2351** LDYEQSRQHT IFVRAVDGGM PTLSSDVIVT VDVTDLNDNP PLFEQQIYEA

**2401** R**ISEHAPHGH FVTCVK**AYDA DSSDIDKLQY SILSGNDHK**H FVIDSATGII**

**2451 TLSNLHR**HAL KPFYSLNLSV SDGVFRSSTQ VHVTVIGGNL HSPAFLQNEY

**2501** EVELAENAPL HTLVMEVK**TT DGDSGIYGHV TYHIVNDFAK** DRFYINERGQ

**2551** IFTLEKLDRE TPAEKVISVR LMAKDAGGKV AFCTVNVILT DDNDNAPQFR

**2601** ATK**YEVNIGS SAAK**GTSVVK **VLASDADEGS NADITYAIEA DSESVKENLE**

**2651 INK**LSGVITT KESLIGLENE FFTFFVRAVD NGSPSK**ESVV LVYVK**ILPPE

**2701** MQLPKFSEPF YTFTVSEDVP IGTEIDLIRA EHSGTVLYSL VKGNTPESNR

**2751** DESFVIDRQS GRLKLEKSLD HETTK**WYQFS ILAR**CTQDDH EMVASVDVSI

**2801** QVK**DANDNSP VFESSPYEAF IVENLPGGSR** VIQIRASDAD SGTNGQVMYS

**2851** LDQSQSVEVI ESFAINMETG WITTLKELDH EKRDNYQIKV VASDHGEKIQ

**2901** LSSTAIVDVT VTDVNDSPPR FTAEIYKGTV SEDDPQGGVI AILSTTDADS

**2951** EEINRQVTYF ITGGDPLGQF AVETIQNEWK VYVKKPLDRE KR**DNYLLTIT**

**3001 ATDGTFSSK**A IVEVK**VLDAN DNSPVCEKTL YSDTIPEDVL PGKLIMQISA**

**3051 TDADIRSNAE ITYTLLGSGA EK**FKLNPDTG ELK**TSTPLDR EEQAVYHLLV**

**3101 R**ATDGGGRFC QASIVLTLED VNDNAPEFSA DPYAITVFEN TEPGTLLTRV

**3151** QATDADAGLN RKILYSLIDS ADGQFSINEL SGIIQLEKPL DRELQAVYTL

**3201** SLKAVDQGLP RRLTATGTVI VSVLDINDNP PVFEYREYGA TVSEDILVGT

**3251** EVLQVYAASR DIEANAEITY SIISGNEHGK FSIDSKTGAV FIIENLDYES

**3301** SHEYYLTVEA TDGGTPSLSD VATVNVNVTD INDNTPVFSQ DTYTTVISED

**3351** AVLEQSVITV MADDADGPSN SHIHYSIIDG NQGSSFTIDP VRGEVKVTKL

**3401** LDRETISGYT LTVQASDNGS PPRVNTTTVN IDVSDVNDNA PVFSRGNYSV

**3451** IIQENKPVGF SVLQLVVTDE DSSHNGPPFF FTIVTGNDEK **AFEVNPQGVL**

**3501 LTSSAIK**RKE K**DHYLLQVKV ADNGKPQLSS LTYIDIR**VIE ESIYPPAILP

**3551** LEIFITSSGE EYSGGVIGK**I HATDQDVYDT LTYSLDPQMD NLFSVSSTGG**

**3601 K**LIAHKKLDI GQYLLNVSVT DGKFTTVADI TVHIRQVTQE MLNHTIAIR**F**

**3651 ANLTPEEFVG DYWR**NFQRAL RNILGVRRND IQIVSLQSSE PHPHLDVLLF

**3701** VEKPGSAQIS TKQLLHKINS SVTDIEEIIG VRILNVFQK**L CAGLDCPWK**F

**3751** CDEK**VSVDES VMSTHSTAR**L SFVTPRHHRA AVCLCKEGRC PPVHHGCEDD

**3801** PCPEGSECVS DPWEEKHTCV CPSGR**FGQCP GSSSMTLTGN SYVK**YRLTEN

**3851** ENKLEMKLTM RLR**TYSTHAV VMYAR**GTDYS ILEIHHGRLQ YKFDCGSGPG

**3901** IVSVQSIQVN DGQWHAVALE VNGNYARLVL DQVHTASGTA PGTLKTLNLD

**3951** NYVFFGGHIR QQGTRHGRSP QVGNGFRGCM DSIYLNGQEL PLNSKPRSYA

**4001** HIEESVDVSP GCFLTATEDC ASNPCQNGGV CNPSPAGGYY CKCSALYIGT

**4051** HCEISVNPCS SKPCLYGGTC VVDNGGFVCQ CRGLYTGQRC QLSPYCKDEP

**4101** CKNGGTCFDS LDGAVCQCDS GFRGERCQSD IDECSGNPCL HGALCENTHG

**4151** SYHCNCSHEY RGRHCEDAAP NQYVSTPWNI GLAEGIGIVV FVAGIFLLVV

**4201** VFVLCRKMIS RKKKHQAEPK DKHLGPATAF LQRPYFDSKL NKNIYSDIPP

**4251** QVPVRPISYT PSIPSDSRNN LDRNSFEGSA IPEHPEFSTF NPESVHGHRK

**4301** AVAVCSVAPN LPPPPPSNSP SDSDSIQKPS WDFDYDTKVV DLDPCLSKKP

**4351** LEEKPSQPYS ARESLSEVQS LSSFQSESCD DNGYHWDTSD WMPSVPLPDI

**4401** QEFPNYEVID EQTPLYSADP NAIDTDYYPG GYDIESDFPP PPEDFPAADE

**4451** LPPLPPEFSN QFESIHPPRD MPAAGSLGSS SRNRQRFNLN QYLPNFYPLD

**4501** MSEPQTKGTG ENSTCREPHA PYPPGYQRHF EAPAVESMPM SVYASTASCS

**4551** DVSACCEVES EVMMSDYESG DDGHFEEVTI PPLDSQQHTE VJDGSQRLEQ

**4601** TPLQFTHLEY NVTVQENSAA KJGTVKEDAP VGSLVMTVSA HDEDAGRDGE

**4651** IRJGTVKEDA PVGSLVMTVS AHDEDARRDG EIRJLDREST SHYWLTVFAT

**4701** DQGVVPLSSF IEIYIEVEDV NDNAPQTSEP VYYPEIMENS PKDVSVVQIE

**4751** AFDPDSSSND KJLDRESTSH YWLTVFATDQ GVVPLSSFIE LYIEVEDVND

**4801** NAPQTSEPVY YPEIMENSPK DVSVVQIEAF DPDSSSNDKJ FYKIRLPERJ

**4851** REPLYHVIAT DKDEGPNAEI SYSIEDGNEH GKJREPLYRV IATDKDEGPN

**4901** AEISYSIEDG NEHGKJEPLY HVIATDKDEG LNAEISYSIE DGNEHGKFFI

**4951** EPKJEPLYHV IATDKDEGPN AEISYSIEDG NEHGKFFIEP KJALEKNTNL

**5001** EARTKJALEK NTNVEARTKJ ALEKNTNVEA RTKJALEKNT NVEVRTKJFS

**5051** AAGEYDILSI KAVDNGRPQK SSTTRJFSAA GEYDILSIKA VDSGRPQKSS

**5101** TTRJNFARIV VNVSDTNDHA PWFTASSYKG RJNFARIVVN VSDTNDHAPW

**5151** FTTSSYKGRJ GKNAEVLYSI ESGDFGNIGN SFMIDPVLGS IKTAKJGKNA

**5201** EVLYSIESGN FGNIGNSFMI DPVLGSIKTA KJGKNAEVLY SIESGTIGNI

**5251** GNSFMIDPVL GSIKTAKJGS PPMSEITSVR IFVTNADNAS PKFTSKJGSP

**5301** PMSEITSVRI FVTSADNASP KFTSKJGSPP MSEITSVRIF VTIADNASTK

**5351** FTSKJALDFE TLPIYTLIIQ GTNMAGLSTN TTVLVHLQDE NDNAPVFMQA

**5401** EYTGLISESA SINSVVLTDR NVPQVIRAAD ADKJALDFET LPIYTLIIQG

**5451** TNMAGLSTNT TVLVHLQDEN DNAPVFMQAE YTGLISESAS INSVVLTDRN

**5501** VPRVIRAADA DKJAADADKD SNVLLVYHIV EPSVHTYFAI DSSTGAIHTV

**5551** LSLDYEETSI FHFTVQVHDM GTPRLFAEYA ANVTVHVIDI NDCPPVFAKP

**5601** LYEASLLLPT YKJFTQDVYS AVVKTNSTEA ETLAVITAIG NPINEPLFYH

**5651** ILNPDRRJTS GVLSTTGTPF DREAQEAFDV VVEVTEEHKP SAVAHVVVKV

**5701** IVEDQNDNAP VFVNLPYYAV VKJTDMFAIH PTSGVIVLTG RLDFLETKLY

**5751** EMEILAADRJ TDMFAIHPTS GVIVLTGRLD YLETKLYEME ILAADRJNGE

**5801** VHYYLKEHHE HFQIGPLGEK SLKKJDGGNP AFSAEVIVPI TVMNKAMPVF

**5851** EKPFYSAEIA ESIQVHSHVV HVQANSPEGL KVFYSITDGD PFSQFTINFN

**5901** TGVINVIAPL DFEAHPAYKJ AMPVFEKPFY SAEIAESIQV HSPVVHVQAN

**5951** SPEGLKVFYS ITDGDPFSQF TINFNTGVIN VIAPLDFPAH PAYKLSIRJL

**6001** SIRATDSLTG AHAEVFVDDI VDDINDNPPV FAQQSYAVTL SEASVIGTSV

**6051** VQVRATDSDS EPNRJLSIRA TDSLTGAHAE VFVDEIVDDI NDNPPVFAQQ

**6101** SYAVTLSEAS VIGTSVVQVR ATDSDSEPNR JTLDYEQSRQ HTIAVRAVDG

**6151** GMPTLSSDVI VTVDVTDLND NPPLFEQQIY EARJLYGSSG ISSMAKLTVH

**6201** IEQANECAPV ITAVTLSPSE LDRDPAYAIV TVDDCDQGAN GDIASLSIVA

**6251** GDLLQQFRJI LPPEMQLPKF SEPFYTFTVS EIVPIGTEID LIRAEHSGTV

**6301** LYSLVKJILP PEMQLPKFSE PFYTFTVSEV VPIGTEIDLI RAEHSGTVLY

**6351** SLVKJCTQDD HEMVASVDVS IQVKDANDNS PVFESSPYEA FIVENLPGGS

**6401** RVIQIRJCTQ DDHEMVASVD VSIQVKDASD NSPVFESSPY EAFIVENLPG

**6451** GSRVIQIRJV IQIRASDADN GTNGQVMYSL DQSQSVEVIE SFAINMETGW

**6501** ITTLKELDHE KJFTAEIYKG TVSELDPQGG VIAILSTTDA DSEEINRQVT

**6551** YFITGGDPLG QFAVETIQNE WKJFTAEIYK GTVSEPDPQG GVIAILSTTD

**6601** ADSEEINRQV TYFITGGDPL GQFAVETIQN EWKJFTAEIY KGTVSEQDPQ

**6651** GGVIAILSTT DADSEEINRQ VTYFITGGDP LGQFAVETIQ NEWKJGTVSE

**6701** DDPQGGVIAI LSTTDADSEE INRQVTYGIT GGDPLGQFAV ETIQNEWKVY

**6751** VKJGTVSEDD PQGGVIAILS TTDADSEEIN RQVTYSITGG DPLGQFAVET

**6801** IQNEWKVYVK JGTVSEDDPQ GGVIAILSTT DADSEEINRQ VTYFITGGDP

**6851** LAQFAVETIQ NEWKVYVKJA IVEVKVLDAN DNSPVCEYTL YSDTIPEDVL

**6901** PGKLIMQISA TDADIRJVLD ANDNSPVCEK TLYIDTIPED VLPGKLIMQI

**6951** SATDADIRJV LDANDNSPVC EKTLYTDTIP EDVLPGKLIM QISATDADIR

**7001** JATDGGGRFC QASIVLDLED VNDNAPEFSA DPYAITVFEN TEPGTLLTRV

**7051** QATDADAGLN RJKILYSLID SADGQFSINE LIGIIQLEKP LDRELQAVYT

**7101** LSLKJFSIDS KTGAVFIIEN LDYESSHEYY LTVEATDGGT PSLSDVATVN

**7151** VNVTDINDAT PVFSQDTYTT VISEDAVLEQ SVITVMADDA DGPSNSHIHY

**7201** SIIDGNQGSS FTIDPVRGEV KJFSIDSKTG AVFIIENLDY ESSHEYYLTV

**7251** EATDGGTPSL SDVATVNVNV TDINDVTPVF SQDTYTTVIS EDAVLEQSVI

**7301** TVMADDADGP SNSHIHYSII DGNQGSSFTI DPVRGEVKJV NTTTVNIDVS

**7351** DVNDNAPVFS RGNYSVIIQE NKPVGFSVLQ LVVTDEDSSH NGPPFFFTIV

**7401** TENDEKAFEV NPQGVLLTSS AIKJVNTTTV NIDVSDVNDN APVFSRGNYS

**7451** VIIQENKPVG FSVLQLVVTD EDSSHNGPPF FFTIVTGNDE KAFEVNPQGV

**7501** LLTSSAIKJG NYSVIIQENK PVGFSVLQLV VTDEDSSHNG PPFFFTIVTG

**7551** NDEKAFEVNA QGVLLTSSAI KRJGNYSVII QENKPVGFSV LQLVVTDEDS

**7601** SHNGPPFFFT IVTGNDEKAF EVNVQGVLLT SSAIKRJVAD NGKPQLSSLT

**7651** YIDIRVIEES IYPPAILPLE IAITSSGEEY SGGVIGKIHA TDQDVYDTLT

**7701** YSLDPQMDNL FSVSSTGGKJ VADNGKPQLS SLTYIDIRVI EESIYPPAIL

**7751** PLEISITSSG EEYSGGVIGK IHATDQDVYD TLTYSLDPQM DNLFSVSSTG

**7801** GKJVIHVTSP QFKAGPVKFE KJQLLHKIMS SVTDIEEIIG VRILNVFQKJ

**7851** QLLHKIVSSV TDIEEIIGVR ILNVFQKJEG RCPPVHHGCE DHPCPEGSEC

**7901** VSDPWEEKHT CVCPSGRJEG RCPPVHHGCE DPPCPEGSEC VSDPWEEKHT

**7951** CVCPSGRJHT CVCPSGRFGG CPGSSSMTLT GNSYVKYRJH TCVCPSGRFG

**8001** RCPGSSSMTL TGNSYVKYRJ FGQCPGSSSM TLTGNSYVKT RLTENENKJG

**8051** CMDSIYLNGQ ELPLNSKPRS YAHIEESVPV SPGCFLTATE DCASNPCQNG

**8101** GVCNPSPAGG YYCKCSALYI GTHCEISVNP CSSKPCLYGG TCVVDNGGFV

**8151** CQCRJYVFKR TPGKAKJYVF KSTPGKAKJS YAHIEESVDV SPGCFLTATE

**8201** DCASNPCQNG GVCNPSPAGG YYCKCSALYI GTHCEISVNP KSSKPCLYGG

**8251** TCVVDNGGFV CQCRGLYTGQ RJSYAHIEES VDVSPGCFLT ATEDCASNPC

**8301** QNGGVCNPSP AGGYYCKCSA LYIGTHCEIS VNPNSSKPCL YGGTCVVDNG

**8351** GFVCQCRGLY TGQRJDEPCK NGGTCFDSAD GAVCQCDSGF RGERJGRHCA

**8401** DAAPNQYVST PWNIGLAEGI GIVVFVAGIF LLVVVFVLCR KJGRHCEDAA

**8451** PNQYVSTPWN IGLAEGIGIV VFVAGIFLLV VVFVLCRKJN IYSDIPPQVP

**8501** VRYISYTPSI PSDSRNNLDR JKAVAVCSVA PNLPPPPPSN SPSDSDSIQK

**8551** PSWDFDYDTK VVDLDPCLSK JKPLEEKPSQ PYSARESLSS VQSLSSFQSE

**8601** SCDDNGYHWD TSDWMPSVPL PDIQEFPNYE VIDEQTPLYS ADPNAIDTDY

**8651** YPGGYDIESD FPPPPEDFPA ADELPPLPPE FSNQFESIHP PRDMPAAGSL

**8701** GSSSRJKPLE EKPSQPYSAR ESLSEVQSLS SFQSESCDDN GYHWDTSDWM

**8751** PSVPLQDIQE FPNYEVIDEQ TPLYSADPNA IDTDYYPGGY DIESDFPPPP

**8801** EDFPAADELP PLPPEFSNQF ESIHPPRDMP AAGSLGSSSR JKPLEEKPSQ

**8851** PYSARESLSE VQSLSSFQSE SCDDNGYHWD TSDWMPSVPL PDIQEFPNYE

**8901** VIDPQTPLYS ADPNAIDTDY YPGGYDIESD FPPPPEDFPA ADELPPLPPE

**8951** FSNQFESIHP PRDMPAAGSL GSSSRJESLS EVQSLSSFQS ESCDDNGYHW

**9001** DTSDWMPSVP LPDIQEFPNY EVIDEQTPLY SADPNAIDTD YYPGGYDIES

**9051** DFPPPPEDFP AADELPPLPP EFSNQFESIH PPRDMPAAGS LGSSRRNRJE

**9101** SLSEVQSLSS FQSESCDDNG YHWDTSDWMP SVPLPDIQEF PNYEVIDEQT

**9151** PLYSADPNAI DTDYYPGGYD IESDFPPPPE DFPAADELPP LPPEFSNQFE

**9201** SIHPPRDMPA AGSLGSSWRN RJVLGANSNP PEFTQTAYKA AFDENVPIGT

**9251** TIMSLSAVDP DEGENGYVTY SIANLNHVPF AIDHFTGAVS TSENLDYELM

**9301** PRVYTLRJVL GANSNPPEFT QTAYKAAFDE NVPIGTTVMS LSAVDPDEGE

**9351** NGYVTYSIAN LNHVPFAIDH FTGAVSTSEN LDYELMPRVY TLRJVLGANS

**9401** NPPEFTQTAY KAAFDENVPI GTTVMSLSAV DPDEGENGYV TYSIANLNHV

**9451** PFAIDHFTGA VSTSENLDYE LMPRVYTLRJ INCEGTIPRD LGVGEQITTV

**9501** SAIDADELQL VQYQIEAGNE LDFFSLNPNS GVLSLKRJIN CEGTIPRDLG

**9551** VGEQITTVSA IDADELQLVQ YQIEAGNELD LFSLNPNSGV LSLKRJINCE

**9601** GTIPRDLGVG EQITTVSAID ADELQLVQYQ IEAGNELDFF SLNPNSGVLS

**9651** LKRJEVHSEI IQVEATDKDL GPNGHVTYSI LTDTDTFSID SVTGVVNIAR

**9701** PLDRELQHEH SLKJEVHSEI IQVEATDKDL GPNGHVTYSI VTDTDTFSID

**9751** SVTGVVNIAR PLDRELQHEH SLK

**Start - End Observed Mr(expt) Mr(calc) ppm Miss Sequence**

**85 - 97 816.8895 1631.7645 1631.7654 -1 0 K.AEEYILGDFCFLR.I**  ([Ions score 55](http://mbp-mascot4/mascot/cgi/peptide_view.pl?file=../data/20091021/F006304.dat&query=3531&hit=1&index=IPI00031411&px=1&section=5&ave_thresh=22))

**85 - 97 816.8898 1631.7650 1631.7654 -0 0 K.AEEYILGDFCFLR.I**  ([Ions score 68](http://mbp-mascot4/mascot/cgi/peptide_view.pl?file=../data/20091021/F006304.dat&query=3532&hit=1&index=IPI00031411&px=1&section=5&ave_thresh=22))

**114 - 121 494.7768 987.5390 987.5389 0 0 K.DHYTLIVK.A**  ([Ions score 25](http://mbp-mascot4/mascot/cgi/peptide_view.pl?file=../data/20091021/F006304.dat&query=1376&hit=1&index=IPI00031411&px=1&section=5&ave_thresh=22))

**137 - 165 1078.2331 3231.6776 3231.6776 0 0 R.VQVLDTNDLRPLFSPTSYSVSLPENTAIR.T**  ([Ions score 35](http://mbp-mascot4/mascot/cgi/peptide_view.pl?file=../data/20091021/F006304.dat&query=4730&hit=1&index=IPI00031411&px=1&section=5&ave_thresh=22))

**192 - 209 639.0080 1914.0021 1914.0033 -1 0 R.TDMFAIHPTSGVIVLTGR.L**  ([Ions score 38](http://mbp-mascot4/mascot/cgi/peptide_view.pl?file=../data/20091021/F006304.dat&query=3991&hit=1&index=IPI00031411&px=1&section=5&ave_thresh=22))

**217 - 227 662.3339 1322.6532 1322.6540 -1 0 K.LYEMEILAADR.G**  ([Ions score 48](http://mbp-mascot4/mascot/cgi/peptide_view.pl?file=../data/20091021/F006304.dat&query=2932&hit=1&index=IPI00031411&px=1&section=5&ave_thresh=22))

**217 - 227 662.3344 1322.6543 1322.6540 0 0 K.LYEMEILAADR.G**  ([Ions score 23](http://mbp-mascot4/mascot/cgi/peptide_view.pl?file=../data/20091021/F006304.dat&query=2933&hit=1&index=IPI00031411&px=1&section=5&ave_thresh=22))

**231 - 242 600.7999 1199.5852 1199.5856 -0 0 K.LYGSSGISSMAK.L**  ([Ions score 49](http://mbp-mascot4/mascot/cgi/peptide_view.pl?file=../data/20091021/F006304.dat&query=2394&hit=1&index=IPI00031411&px=1&section=5&ave_thresh=22))

**231 - 242 600.8008 1199.5871 1199.5856 1 0 K.LYGSSGISSMAK.L**  ([Ions score 69](http://mbp-mascot4/mascot/cgi/peptide_view.pl?file=../data/20091021/F006304.dat&query=2395&hit=1&index=IPI00031411&px=1&section=5&ave_thresh=22))

**243 - 269 988.5089 2962.5049 2962.5070 -1 0 K.LTVHIEQANECAPVITAVTLSPSELDR.D**  ([Ions score 34](http://mbp-mascot4/mascot/cgi/peptide_view.pl?file=../data/20091021/F006304.dat&query=4655&hit=1&index=IPI00031411&px=1&section=5&ave_thresh=22))

**243 - 269 988.5106 2962.5098 2962.5070 1 0 K.LTVHIEQANECAPVITAVTLSPSELDR.D**  ([Ions score 33](http://mbp-mascot4/mascot/cgi/peptide_view.pl?file=../data/20091021/F006304.dat&query=4656&hit=1&index=IPI00031411&px=1&section=5&ave_thresh=22))

**352 - 361 578.3299 1154.6452 1154.6448 0 0 K.VIHVTSPQFK.A**  ([Ions score 29](http://mbp-mascot4/mascot/cgi/peptide_view.pl?file=../data/20091021/F006304.dat&query=2203&hit=1&index=IPI00031411&px=1&section=5&ave_thresh=22))

**374 - 390 914.9797 1827.9449 1827.9441 0 0 R.AEISEFAPPNTPVVMVK.A**  ([Ions score 37](http://mbp-mascot4/mascot/cgi/peptide_view.pl?file=../data/20091021/F006304.dat&query=3844&hit=1&index=IPI00031411&px=1&section=5&ave_thresh=22))

**374 - 390 922.9761 1843.9377 1843.9390 -1 0 R.AEISEFAPPNTPVVMVK.A**  Oxidation (M) ([Ions score 36](http://mbp-mascot4/mascot/cgi/peptide_view.pl?file=../data/20091021/F006304.dat&query=3879&hit=1&index=IPI00031411&px=1&section=5&ave_thresh=22))

**411 - 427 954.5265 1907.0385 1907.0404 -1 0 K.FSLNYNTGLISILEPVK.R**  ([Ions score 89](http://mbp-mascot4/mascot/cgi/peptide_view.pl?file=../data/20091021/F006304.dat&query=3977&hit=1&index=IPI00031411&px=1&section=5&ave_thresh=22))

**411 - 427 954.5279 1907.0412 1907.0404 0 0 K.FSLNYNTGLISILEPVK.R**  ([Ions score 69](http://mbp-mascot4/mascot/cgi/peptide_view.pl?file=../data/20091021/F006304.dat&query=3978&hit=1&index=IPI00031411&px=1&section=5&ave_thresh=22))

**453 - 469 918.9591 1835.9036 1835.9054 -1 0 K.VLGANSNPPEFTQTAYK.A**  ([Ions score 32](http://mbp-mascot4/mascot/cgi/peptide_view.pl?file=../data/20091021/F006304.dat&query=3863&hit=1&index=IPI00031411&px=1&section=5&ave_thresh=22))

**453 - 469 918.9593 1835.9040 1835.9054 -1 0 K.VLGANSNPPEFTQTAYK.A**  ([Ions score 93](http://mbp-mascot4/mascot/cgi/peptide_view.pl?file=../data/20091021/F006304.dat&query=3864&hit=1&index=IPI00031411&px=1&section=5&ave_thresh=22))

**540 - 548 532.7613 1063.5080 1063.5087 -1 0 R.ASDWGLPYR.R**  ([Ions score 36](http://mbp-mascot4/mascot/cgi/peptide_view.pl?file=../data/20091021/F006304.dat&query=1727&hit=1&index=IPI00031411&px=1&section=5&ave_thresh=22))

**540 - 548 532.7613 1063.5081 1063.5087 -1 0 R.ASDWGLPYR.R**  ([Ions score 36](http://mbp-mascot4/mascot/cgi/peptide_view.pl?file=../data/20091021/F006304.dat&query=1728&hit=1&index=IPI00031411&px=1&section=5&ave_thresh=22))

**669 - 681 730.8733 1459.7320 1459.7341 -1 0 K.LVNLQCEETGVAK.M**  ([Ions score 60](http://mbp-mascot4/mascot/cgi/peptide_view.pl?file=../data/20091021/F006304.dat&query=3249&hit=1&index=IPI00031411&px=1&section=5&ave_thresh=22))

**669 - 681 730.8750 1459.7354 1459.7341 1 0 K.LVNLQCEETGVAK.M**  ([Ions score 44](http://mbp-mascot4/mascot/cgi/peptide_view.pl?file=../data/20091021/F006304.dat&query=3250&hit=1&index=IPI00031411&px=1&section=5&ave_thresh=22))

**693 - 717 734.8573 2935.4001 2935.4002 -0 0 K.LHNQGEVEDIFFDSHSVNAHIPQFR.S**  ([Ions score 37](http://mbp-mascot4/mascot/cgi/peptide_view.pl?file=../data/20091021/F006304.dat&query=4638&hit=1&index=IPI00031411&px=1&section=5&ave_thresh=22))

**718 - 727 522.3088 1042.6030 1042.6023 1 0 R.STLPTGIQVK.E**  ([Ions score 35](http://mbp-mascot4/mascot/cgi/peptide_view.pl?file=../data/20091021/F006304.dat&query=1621&hit=1&index=IPI00031411&px=1&section=5&ave_thresh=22))

**776 - 782 407.2456 812.4766 812.4756 1 0 K.ILSPLDR.E**  ([Ions score 25](http://mbp-mascot4/mascot/cgi/peptide_view.pl?file=../data/20091021/F006304.dat&query=347&hit=1&index=IPI00031411&px=1&section=5&ave_thresh=22))

**776 - 782 407.2456 812.4766 812.4756 1 0 K.ILSPLDR.E**  ([Ions score 24](http://mbp-mascot4/mascot/cgi/peptide_view.pl?file=../data/20091021/F006304.dat&query=348&hit=2&index=IPI00031411&px=1&section=5&ave_thresh=22))

**915 - 933 1094.0382 2186.0619 2186.0644 -1 0 K.VSLEDVNDNPPTFIPPNYR.V**  ([Ions score 35](http://mbp-mascot4/mascot/cgi/peptide_view.pl?file=../data/20091021/F006304.dat&query=4190&hit=1&index=IPI00031411&px=1&section=5&ave_thresh=22))

**1084 - 1096 703.3433 1404.6720 1404.6732 -1 0 K.IGEETGVIETSDR.L**  ([Ions score 62](http://mbp-mascot4/mascot/cgi/peptide_view.pl?file=../data/20091021/F006304.dat&query=3128&hit=1&index=IPI00031411&px=1&section=5&ave_thresh=22))

**1084 - 1096 703.3435 1404.6724 1404.6732 -1 0 K.IGEETGVIETSDR.L**  ([Ions score 77](http://mbp-mascot4/mascot/cgi/peptide_view.pl?file=../data/20091021/F006304.dat&query=3129&hit=1&index=IPI00031411&px=1&section=5&ave_thresh=22))

**1178 - 1192 543.9510 1628.8313 1628.8311 0 0 K.ITSGNPQGFFSIHPK.T**  ([Ions score 27](http://mbp-mascot4/mascot/cgi/peptide_view.pl?file=../data/20091021/F006304.dat&query=3521&hit=1&index=IPI00031411&px=1&section=5&ave_thresh=22))

**1235 - 1248 851.4443 1700.8740 1700.8733 0 0 K.ILDENDNKPQFLQK.F**  ([Ions score 26](http://mbp-mascot4/mascot/cgi/peptide_view.pl?file=../data/20091021/F006304.dat&query=3637&hit=1&index=IPI00031411&px=1&section=5&ave_thresh=22))

**1235 - 1248 567.9656 1700.8749 1700.8733 1 0 K.ILDENDNKPQFLQK.F**  ([Ions score 37](http://mbp-mascot4/mascot/cgi/peptide_view.pl?file=../data/20091021/F006304.dat&query=3638&hit=1&index=IPI00031411&px=1&section=5&ave_thresh=22))

**1235 - 1248 567.9658 1700.8754 1700.8733 1 0 K.ILDENDNKPQFLQK.F**  ([Ions score 26](http://mbp-mascot4/mascot/cgi/peptide_view.pl?file=../data/20091021/F006304.dat&query=3639&hit=1&index=IPI00031411&px=1&section=5&ave_thresh=22))

**1314 - 1326 707.3658 1412.7170 1412.7187 -1 0 R.FSAAGEYDILSIK.A**  ([Ions score 50](http://mbp-mascot4/mascot/cgi/peptide_view.pl?file=../data/20091021/F006304.dat&query=3142&hit=1&index=IPI00031411&px=1&section=5&ave_thresh=22))

**1314 - 1326 707.3671 1412.7197 1412.7187 1 0 R.FSAAGEYDILSIK.A**  ([Ions score 61](http://mbp-mascot4/mascot/cgi/peptide_view.pl?file=../data/20091021/F006304.dat&query=3143&hit=1&index=IPI00031411&px=1&section=5&ave_thresh=22))

**1407 - 1422 820.9623 1639.9101 1639.9145 -3 0 K.GTGTIIVAKPLDAEQK.S**  ([Ions score 55](http://mbp-mascot4/mascot/cgi/peptide_view.pl?file=../data/20091021/F006304.dat&query=3550&hit=1&index=IPI00031411&px=1&section=5&ave_thresh=22))

**1489 - 1497 540.8059 1079.5973 1079.5975 -0 0 K.LIYTLQSSR.D**  ([Ions score 37](http://mbp-mascot4/mascot/cgi/peptide_view.pl?file=../data/20091021/F006304.dat&query=1787&hit=1&index=IPI00031411&px=1&section=5&ave_thresh=22))

**1489 - 1497 540.8061 1079.5976 1079.5975 0 0 K.LIYTLQSSR.D**  ([Ions score 22](http://mbp-mascot4/mascot/cgi/peptide_view.pl?file=../data/20091021/F006304.dat&query=1788&hit=1&index=IPI00031411&px=1&section=5&ave_thresh=22))

**1507 - 1519 691.3453 1380.6760 1380.6773 -1 0 R.LDPATGSLYTSEK.L**  ([Ions score 84](http://mbp-mascot4/mascot/cgi/peptide_view.pl?file=../data/20091021/F006304.dat&query=3053&hit=1&index=IPI00031411&px=1&section=5&ave_thresh=22))

**1507 - 1519 691.3467 1380.6789 1380.6773 1 0 R.LDPATGSLYTSEK.L**  ([Ions score 73](http://mbp-mascot4/mascot/cgi/peptide_view.pl?file=../data/20091021/F006304.dat&query=3054&hit=1&index=IPI00031411&px=1&section=5&ave_thresh=22))

**1520 - 1535 472.2485 1884.9648 1884.9629 1 0 K.LDHEAVHQHTLTVMVR.D**  ([Ions score 25](http://mbp-mascot4/mascot/cgi/peptide_view.pl?file=../data/20091021/F006304.dat&query=3953&hit=1&index=IPI00031411&px=1&section=5&ave_thresh=22))

**1520 - 1535 472.2487 1884.9656 1884.9629 1 0 K.LDHEAVHQHTLTVMVR.D**  ([Ions score 22](http://mbp-mascot4/mascot/cgi/peptide_view.pl?file=../data/20091021/F006304.dat&query=3954&hit=1&index=IPI00031411&px=1&section=5&ave_thresh=22))

**1631 - 1641 649.3079 1296.6013 1296.6020 -1 0 R.SNQAEYDLMVK.A**  ([Ions score 41](http://mbp-mascot4/mascot/cgi/peptide_view.pl?file=../data/20091021/F006304.dat&query=2786&hit=1&index=IPI00031411&px=1&section=5&ave_thresh=22))

**1631 - 1641 657.3043 1312.5940 1312.5969 -2 0 R.SNQAEYDLMVK.A**  Oxidation (M) ([Ions score 39](http://mbp-mascot4/mascot/cgi/peptide_view.pl?file=../data/20091021/F006304.dat&query=2853&hit=1&index=IPI00031411&px=1&section=5&ave_thresh=22))

**1646 - 1657 630.8159 1259.6173 1259.6180 -1 0 K.GSPPMSEITSVR.I**  ([Ions score 31](http://mbp-mascot4/mascot/cgi/peptide_view.pl?file=../data/20091021/F006304.dat&query=2657&hit=1&index=IPI00031411&px=1&section=5&ave_thresh=22))

**1646 - 1657 630.8162 1259.6178 1259.6180 -0 0 K.GSPPMSEITSVR.I**  ([Ions score 36](http://mbp-mascot4/mascot/cgi/peptide_view.pl?file=../data/20091021/F006304.dat&query=2658&hit=1&index=IPI00031411&px=1&section=5&ave_thresh=22))

**1658 - 1669 638.3502 1274.6858 1274.6870 -1 0 R.IFVTIADNASPK.F**  ([Ions score 46](http://mbp-mascot4/mascot/cgi/peptide_view.pl?file=../data/20091021/F006304.dat&query=2716&hit=1&index=IPI00031411&px=1&section=5&ave_thresh=22))

**1658 - 1669 638.3511 1274.6877 1274.6870 1 0 R.IFVTIADNASPK.F**  ([Ions score 53](http://mbp-mascot4/mascot/cgi/peptide_view.pl?file=../data/20091021/F006304.dat&query=2717&hit=1&index=IPI00031411&px=1&section=5&ave_thresh=22))

**1706 - 1726 734.3544 2200.0415 2200.0397 1 0 K.DGNTGDAFDINPHSGTIITQK.A**  ([Ions score 28](http://mbp-mascot4/mascot/cgi/peptide_view.pl?file=../data/20091021/F006304.dat&query=4199&hit=1&index=IPI00031411&px=1&section=5&ave_thresh=22))

**1962 - 1969 411.7400 821.4654 821.4647 1 0 R.FAGLTSVK.I**  ([Ions score 23](http://mbp-mascot4/mascot/cgi/peptide_view.pl?file=../data/20091021/F006304.dat&query=372&hit=1&index=IPI00031411&px=1&section=5&ave_thresh=22))

**1982 - 1992 628.8298 1255.6450 1255.6449 0 0 K.FTQDVYSAVVK.E**  ([Ions score 60](http://mbp-mascot4/mascot/cgi/peptide_view.pl?file=../data/20091021/F006304.dat&query=2636&hit=1&index=IPI00031411&px=1&section=5&ave_thresh=22))

**1982 - 1992 628.8301 1255.6456 1255.6449 1 0 K.FTQDVYSAVVK.E**  ([Ions score 45](http://mbp-mascot4/mascot/cgi/peptide_view.pl?file=../data/20091021/F006304.dat&query=2637&hit=1&index=IPI00031411&px=1&section=5&ave_thresh=22))

**2031 - 2044 719.8622 1437.7098 1437.7100 -0 0 R.TSGVLSTTGTPFDR.E**  ([Ions score 63](http://mbp-mascot4/mascot/cgi/peptide_view.pl?file=../data/20091021/F006304.dat&query=3200&hit=1&index=IPI00031411&px=1&section=5&ave_thresh=22))

**2031 - 2044 719.8630 1437.7115 1437.7100 1 0 R.TSGVLSTTGTPFDR.E**  ([Ions score 43](http://mbp-mascot4/mascot/cgi/peptide_view.pl?file=../data/20091021/F006304.dat&query=3201&hit=1&index=IPI00031411&px=1&section=5&ave_thresh=22))

**2072 - 2094 869.4546 2605.3421 2605.3428 -0 0 K.VIVEDQNDNAPVFVNLPYYAVVK.V**  ([Ions score 35](http://mbp-mascot4/mascot/cgi/peptide_view.pl?file=../data/20091021/F006304.dat&query=4482&hit=1&index=IPI00031411&px=1&section=5&ave_thresh=22))

**2095 - 2104 375.5406 1123.6001 1123.5986 1 0 K.VDTEVGHVIR.Y**  ([Ions score 27](http://mbp-mascot4/mascot/cgi/peptide_view.pl?file=../data/20091021/F006304.dat&query=2052&hit=1&index=IPI00031411&px=1&section=5&ave_thresh=22))

**2095 - 2104 375.5410 1123.6012 1123.5986 2 0 K.VDTEVGHVIR.Y**  ([Ions score 24](http://mbp-mascot4/mascot/cgi/peptide_view.pl?file=../data/20091021/F006304.dat&query=2053&hit=1&index=IPI00031411&px=1&section=5&ave_thresh=22))

**2161 - 2180 1030.0292 2058.0438 2058.0456 -1 0 K.DGGNPAFSAEVIVPITVMNK.A**  ([Ions score 42](http://mbp-mascot4/mascot/cgi/peptide_view.pl?file=../data/20091021/F006304.dat&query=4095&hit=1&index=IPI00031411&px=1&section=5&ave_thresh=22))

**2402 - 2416 573.6196 1717.8370 1717.8359 1 0 R.ISEHAPHGHFVTCVK.A**  ([Ions score 24](http://mbp-mascot4/mascot/cgi/peptide_view.pl?file=../data/20091021/F006304.dat&query=3669&hit=1&index=IPI00031411&px=1&section=5&ave_thresh=22))

**2440 - 2457 665.3650 1993.0733 1993.0745 -1 0 K.HFVIDSATGIITLSNLHR.H**  ([Ions score 77](http://mbp-mascot4/mascot/cgi/peptide_view.pl?file=../data/20091021/F006304.dat&query=4062&hit=1&index=IPI00031411&px=1&section=5&ave_thresh=22))

**2519 - 2540 804.0477 2409.1212 2409.1237 -1 0 K.TTDGDSGIYGHVTYHIVNDFAK.D**  ([Ions score 45](http://mbp-mascot4/mascot/cgi/peptide_view.pl?file=../data/20091021/F006304.dat&query=4421&hit=1&index=IPI00031411&px=1&section=5&ave_thresh=22))

**2604 - 2614 569.7907 1137.5669 1137.5666 0 0 K.YEVNIGSSAAK.G**  ([Ions score 44](http://mbp-mascot4/mascot/cgi/peptide_view.pl?file=../data/20091021/F006304.dat&query=2124&hit=1&index=IPI00031411&px=1&section=5&ave_thresh=22))

**2604 - 2614 569.7913 1137.5680 1137.5666 1 0 K.YEVNIGSSAAK.G**  ([Ions score 23](http://mbp-mascot4/mascot/cgi/peptide_view.pl?file=../data/20091021/F006304.dat&query=2125&hit=1&index=IPI00031411&px=1&section=5&ave_thresh=22))

**2621 - 2653 1170.8938 3509.6596 3509.6532 2 1 K.VLASDADEGSNADITYAIEADSESVKENLEINK.L**  ([Ions score 97](http://mbp-mascot4/mascot/cgi/peptide_view.pl?file=../data/20091021/F006304.dat&query=4838&hit=1&index=IPI00031411&px=1&section=5&ave_thresh=22))

**2687 - 2695 518.3088 1034.6031 1034.6012 2 0 K.ESVVLVYVK.I**  ([Ions score 27](http://mbp-mascot4/mascot/cgi/peptide_view.pl?file=../data/20091021/F006304.dat&query=1598&hit=1&index=IPI00031411&px=1&section=5&ave_thresh=22))

**2776 - 2784 592.3162 1182.6178 1182.6186 -1 0 K.WYQFSILAR.C**  ([Ions score 43](http://mbp-mascot4/mascot/cgi/peptide_view.pl?file=../data/20091021/F006304.dat&query=2308&hit=1&index=IPI00031411&px=1&section=5&ave_thresh=22))

**2776 - 2784 592.3175 1182.6204 1182.6186 2 0 K.WYQFSILAR.C**  ([Ions score 24](http://mbp-mascot4/mascot/cgi/peptide_view.pl?file=../data/20091021/F006304.dat&query=2309&hit=1&index=IPI00031411&px=1&section=5&ave_thresh=22))

**2804 - 2830 971.1169 2910.3288 2910.3308 -1 0 K.DANDNSPVFESSPYEAFIVENLPGGSR.V**  ([Ions score 48](http://mbp-mascot4/mascot/cgi/peptide_view.pl?file=../data/20091021/F006304.dat&query=4626&hit=1&index=IPI00031411&px=1&section=5&ave_thresh=22))

**2804 - 2830 971.1172 2910.3299 2910.3308 -0 0 K.DANDNSPVFESSPYEAFIVENLPGGSR.V**  ([Ions score 53](http://mbp-mascot4/mascot/cgi/peptide_view.pl?file=../data/20091021/F006304.dat&query=4627&hit=1&index=IPI00031411&px=1&section=5&ave_thresh=22))

**2993 - 3009 923.9581 1845.9017 1845.8996 1 0 R.DNYLLTITATDGTFSSK.A**  ([Ions score 51](http://mbp-mascot4/mascot/cgi/peptide_view.pl?file=../data/20091021/F006304.dat&query=3882&hit=1&index=IPI00031411&px=1&section=5&ave_thresh=22))

**3016 - 3028 730.8375 1459.6605 1459.6613 -1 0 K.VLDANDNSPVCEK.T**  ([Ions score 73](http://mbp-mascot4/mascot/cgi/peptide_view.pl?file=../data/20091021/F006304.dat&query=3248&hit=1&index=IPI00031411&px=1&section=5&ave_thresh=22))

**3029 - 3043 824.4271 1646.8397 1646.8403 -0 0 K.TLYSDTIPEDVLPGK.L**  ([Ions score 37](http://mbp-mascot4/mascot/cgi/peptide_view.pl?file=../data/20091021/F006304.dat&query=3560&hit=1&index=IPI00031411&px=1&section=5&ave_thresh=22))

**3044 - 3056 723.8835 1445.7524 1445.7548 -2 0 K.LIMQISATDADIR.S**  ([Ions score 84](http://mbp-mascot4/mascot/cgi/peptide_view.pl?file=../data/20091021/F006304.dat&query=3225&hit=1&index=IPI00031411&px=1&section=5&ave_thresh=22))

**3044 - 3056 731.8833 1461.7520 1461.7497 2 0 K.LIMQISATDADIR.S**  Oxidation (M) ([Ions score 62](http://mbp-mascot4/mascot/cgi/peptide_view.pl?file=../data/20091021/F006304.dat&query=3256&hit=1&index=IPI00031411&px=1&section=5&ave_thresh=22))

**3057 - 3072 827.4192 1652.8238 1652.8257 -1 0 R.SNAEITYTLLGSGAEK.F**  ([Ions score 91](http://mbp-mascot4/mascot/cgi/peptide_view.pl?file=../data/20091021/F006304.dat&query=3571&hit=1&index=IPI00031411&px=1&section=5&ave_thresh=22))

**3084 - 3101 709.7114 2126.1123 2126.1120 0 1 K.TSTPLDREEQAVYHLLVR.A**  ([Ions score 40](http://mbp-mascot4/mascot/cgi/peptide_view.pl?file=../data/20091021/F006304.dat&query=4142&hit=1&index=IPI00031411&px=1&section=5&ave_thresh=22))

**3084 - 3101 709.7114 2126.1124 2126.1120 0 1 K.TSTPLDREEQAVYHLLVR.A**  ([Ions score 47](http://mbp-mascot4/mascot/cgi/peptide_view.pl?file=../data/20091021/F006304.dat&query=4143&hit=1&index=IPI00031411&px=1&section=5&ave_thresh=22))

**3491 - 3507 887.4900 1772.9654 1772.9672 -1 0 K.AFEVNPQGVLLTSSAIK.R**  ([Ions score 84](http://mbp-mascot4/mascot/cgi/peptide_view.pl?file=../data/20091021/F006304.dat&query=3747&hit=1&index=IPI00031411&px=1&section=5&ave_thresh=22))

**3512 - 3519 508.2824 1014.5503 1014.5498 0 0 K.DHYLLQVK.V**  ([Ions score 26](http://mbp-mascot4/mascot/cgi/peptide_view.pl?file=../data/20091021/F006304.dat&query=1500&hit=1&index=IPI00031411&px=1&section=5&ave_thresh=22))

**3520 - 3537 664.0250 1989.0532 1989.0531 0 0 K.VADNGKPQLSSLTYIDIR.V**  ([Ions score 23](http://mbp-mascot4/mascot/cgi/peptide_view.pl?file=../data/20091021/F006304.dat&query=4057&hit=1&index=IPI00031411&px=1&section=5&ave_thresh=22))

**3570 - 3601 1173.5471 3517.6195 3517.6195 -0 0 K.IHATDQDVYDTLTYSLDPQMDNLFSVSSTGGK.L**  ([Ions score 75](http://mbp-mascot4/mascot/cgi/peptide_view.pl?file=../data/20091021/F006304.dat&query=4840&hit=1&index=IPI00031411&px=1&section=5&ave_thresh=22))

**3650 - 3664 922.4342 1842.8538 1842.8577 -2 0 R.FANLTPEEFVGDYWR.N**  ([Ions score 38](http://mbp-mascot4/mascot/cgi/peptide_view.pl?file=../data/20091021/F006304.dat&query=3876&hit=1&index=IPI00031411&px=1&section=5&ave_thresh=22))

**3650 - 3664 922.4346 1842.8546 1842.8577 -2 0 R.FANLTPEEFVGDYWR.N**  ([Ions score 35](http://mbp-mascot4/mascot/cgi/peptide_view.pl?file=../data/20091021/F006304.dat&query=3877&hit=1&index=IPI00031411&px=1&section=5&ave_thresh=22))

**3740 - 3749 610.2837 1218.5528 1218.5526 0 0 K.LCAGLDCPWK.F**  ([Ions score 35](http://mbp-mascot4/mascot/cgi/peptide_view.pl?file=../data/20091021/F006304.dat&query=2475&hit=1&index=IPI00031411&px=1&section=5&ave_thresh=22))

**3755 - 3769 803.3805 1604.7465 1604.7464 0 0 K.VSVDESVMSTHSTAR.L**  ([Ions score 74](http://mbp-mascot4/mascot/cgi/peptide_view.pl?file=../data/20091021/F006304.dat&query=3468&hit=1&index=IPI00031411&px=1&section=5&ave_thresh=22))

**3755 - 3769 535.9231 1604.7474 1604.7464 1 0 K.VSVDESVMSTHSTAR.L**  ([Ions score 33](http://mbp-mascot4/mascot/cgi/peptide_view.pl?file=../data/20091021/F006304.dat&query=3469&hit=1&index=IPI00031411&px=1&section=5&ave_thresh=22))

**3755 - 3769 535.9232 1604.7478 1604.7464 1 0 K.VSVDESVMSTHSTAR.L**  ([Ions score 37](http://mbp-mascot4/mascot/cgi/peptide_view.pl?file=../data/20091021/F006304.dat&query=3470&hit=1&index=IPI00031411&px=1&section=5&ave_thresh=22))

**3826 - 3844 1010.9579 2019.9013 2019.9030 -1 0 R.FGQCPGSSSMTLTGNSYVK.Y**  ([Ions score 107](http://mbp-mascot4/mascot/cgi/peptide_view.pl?file=../data/20091021/F006304.dat&query=4076&hit=1&index=IPI00031411&px=1&section=5&ave_thresh=22))

**3826 - 3844 1018.9578 2035.9011 2035.8979 2 0 R.FGQCPGSSSMTLTGNSYVK.Y**  Oxidation (M) ([Ions score 45](http://mbp-mascot4/mascot/cgi/peptide_view.pl?file=../data/20091021/F006304.dat&query=4089&hit=1&index=IPI00031411&px=1&section=5&ave_thresh=22))

**3864 - 3875 699.8458 1397.6771 1397.6762 1 0 R.TYSTHAVVMYAR.G**  ([Ions score 63](http://mbp-mascot4/mascot/cgi/peptide_view.pl?file=../data/20091021/F006304.dat&query=3112&hit=1&index=IPI00031411&px=1&section=5&ave_thresh=22))

**3864 - 3875 466.9003 1397.6790 1397.6762 2 0 R.TYSTHAVVMYAR.G**  ([Ions score 24](http://mbp-mascot4/mascot/cgi/peptide_view.pl?file=../data/20091021/F006304.dat&query=3114&hit=1&index=IPI00031411&px=1&section=5&ave_thresh=22))

**3864 - 3875 472.2314 1413.6725 1413.6711 1 0 R.TYSTHAVVMYAR.G**  Oxidation (M) ([Ions score 25](http://mbp-mascot4/mascot/cgi/peptide_view.pl?file=../data/20091021/F006304.dat&query=3144&hit=1&index=IPI00031411&px=1&section=5&ave_thresh=22))

**PaCa44 A3**

Formularbeginn

Match to: **IPI00031411** Score: **2452**

**Gene_Symbol=FAT1 Protocadherin Fat 1 lng=4591 # SP[4593,D,22,D]SNP[4646,g,1064,R]SNP[4677,r,1064,R]SNP[4713,i,1125,I]SNP[4791,l,1125,I]SNP[4844,i,1252,I]SNP[4856,h,1273,H]SNP[4889,r,1273,H]SNP[4931,l,1283,P]SNP[4969,p,1283,P]SNP[5000,l,129,V]SNP[5**

Found in search of C:\mgf\Orbidata\081103_ISW1295_**Paca44_A3**.mgf

Nominal mass (M_r_): **1068827**; Calculated pI value: **4.55**

NCBI BLAST search of [IPI00031411](http://www.ncbi.nlm.nih.gov/blast/Blast.cgi?ALIGNMENTS=50&ALIGNMENT_VIEW=Pairwise&AUTO_FORMAT=Semiauto&CDD_SEARCH=on&CLIENT=web&COMPOSITION_BASED_STATISTICS=on&DATABASE=nr&DESCRIPTIONS=100&ENTREZ_QUERY=(none)&EXPECT=10&FILTER=L&FORMAT_BLOCK_ON_RESPAGE=None&FORMAT_OBJECT=Alignment&FORMAT_TYPE=HTML&GAPCOSTS=11+1&I_THRESH=0.001&LAYOUT=TwoWindows&MATRIX_NAME=BLOSUM62&NCBI_GI=on&PAGE=Proteins&PROGRAM=blastp&QUERY=IPI00031411&SERVICE=plain&SET_DEFAULTS.x=21&SET_DEFAULTS.y=7&SHOW_OVERVIEW=on&WORD_SIZE=3&END_OF_HTTPGET=Yes) against nr

Unformatted [sequence string](http://mbp-mascot4/mascot/cgi/getseq.pl?IPI_human+IPI00031411+seq) for pasting into other applications

Fixed modifications: Carbamidomethyl (C)

Variable modifications: Oxidation (M)

Cleavage by TrypsinMSIPI, a mixture of enzymes:

cuts C-term side of KR unless next residue is P

cuts C-term side of J

cuts N-term side of J

Sequence Coverage: **10%**

Matched peptides shown in **Bold Red**

**1** MGRHLALLLL LLLLFQHFGD SDGSQRLEQT PLQFTHLEYN VTVQENSAAK

**51** TYVGHPVKMG VYITHPAWEV RYKIVSGDSE NLFK**AEEYIL GDFCFLR**IRT

**101** KGGNTAILNR EVKDHYTLIV KALEKNTNVE ARTKVR**VQVL DTNDLRPLFS**

**151 PTSYSVSLPE NTAIR**TSIAR VSATDADIGT NGEFYYSFKD R**TDMFAIHPT**

**201 SGVIVLTGR**L DYLETK**LYEM EILAADR**GMK **LYGSSGISSM AKLTVHIEQA**

**251 NECAPVITAV TLSPSELDR**D PAYAIVTVDD CDQGANGDIA SLSIVAGDLL

**301** QQFRTVRSFP GSKEYKVKAI GGIDWDSHPF GYNLTLQAKD KGTPPQFSSV

**351** K**VIHVTSPQF K**AGPVKFEKD VYR**AEISEFA PPNTPVVMVK** AIPAYSHLRY

**401** VFKSTPGKAK **FSLNYNTGLI SILEPVK**RQQ AAHFELEVTT SDRKASTKVL

**451** VK**VLGANSNP PEFTQTAYK**A AFDENVPIGT TVMSLSAVDP DEGENGYVTY

**501** SIANLNHVPF AIDHFTGAVS TSENLDYELM PRVYTLRIR**A SDWGLPYR**RE

**551** VEVLATITLN NLNDNTPLFE KINCEGTIPR DLGVGEQITT VSAIDADELQ

**601** LVQYQIEAGN ELDFFSLNPN SGVLSLKRSL MDGLGAKVSF HSLRITATDG

**651** ENFATPLYIN ITVAASHK**LV NLQCEETGVA K**MLAEKLLQA NKLHNQGEVE

**701** DIFFDSHSVN AHIPQFR**STL PTGIQVK**ENQ PVGSSVIFMN STDLDTGFNG

**751** KLVYAVSGGN EDSCFMIDME TGMLKILSPL DRETTDKYTL NITVYDLGIP

**801** QKAAWRLLHV VVVDANDNPP EFLQESYFVE VSEDKEVHSE IIQVEATDKD

**851** LGPNGHVTYS IVTDTDTFSI DSVTGVVNIA RPLDRELQHE HSLKIEARDQ

**901** AR**EEPQLFST VVVKVSLEDV NDNPPTFIPP NYR**VKVREDL PEGTVIMWLE

**951** AHDPDLGQSG QVR**YSLLDHG EGNFDVDK**LS GAVR**IVQQLD FEKK**QVYNLT

**1001** VRAKDKGKPV SLSSTCYVEV EVVDVNENLH PPVFSSFVEK GTVKEDAPVG

**1051** SLVMTVSAHD EDARRDGEIR YSIRDGSGVG VFK**IGEETGV IETSDR**LDRE

**1101** STSHYWLTVF ATDQGVVPLS SFIEIYIEVE DVNDNAPQTS EPVYYPEIME

**1151** NSPKDVSVVQ IEAFDPDSSS NDKLMYK**ITS GNPQGFFSIH PK**TGLITTTS

**1201** RKLDREQQDE HILEVTVTDN GSPPKSTIAR VIVK**ILDEND NKPQFLQK**FY

**1251** KIRLPEREKP DRERNARREP LYHVIATDKD EGPNAEISYS IEDGNEHGKF

**1301** FIEPKTGVVS SKR**FSAAGEY DILSIK**AVDN GRPQKSSTTR LHIEWISKPK

**1351** PSLEPISFEE SFFTFTVMES DPVAHMIGVI SVEPPGIPLW FDITGGNYDS

**1401** HFDVDK**GTGT IIVAKPLDAE QK**SNYNLTVE ATDGTTTILT QVFIK**VIDTN**

**1451 DHRPQFSTSK** YEVVIPEDTA PETEILQISA VDQDEKNK**LI YTLQSSR**DPL

**1501** SLKK**FRLDPA TGSLYTSEKL DHEAVHQHTL TVMVR**DQDVP VKRNFARIVV

**1551** NVSDTNDHAP WFTASSYKGR VYESAAVGSV VLQVTALDKD KGKNAEVLYS

**1601** IESGTFGNIG NSFMIDPVLG SIKTAKELDR **SNQAEYDLMV K**ATDK**GSPPM**

**1651 SEITSVRIFV TIADNASPK**F TSKEYSVELS ETVSIGSFVG MVTAHSQSSV

**1701** VYEIK**DGNTG DAFDINPHSG TIITQK**ALDF ETLPIYTLII QGTNMAGLST

**1751** NTTVLVHLQD ENDNAPVFMQ AEYTGLISES ASINSVVLTD RNVPLVIRAA

**1801** DADKDSNALL VYHIVEPSVH TYFAIDSSTG AIHTVLSLDY EETSIFHFTV

**1851** QVHDMGTPRL FAEYAANVTV HVIDINDCPP VFAKPLYEAS LLLPTYKGVK

**1901** VITVNATDAD SSAFSQLIYS ITEGNIGEKF SMDYKTGALT VQNTTQLRSR

**1951** YELTVRASDG R**FAGLTSVK**I NVKESKESHL KFTQDVYSAV VKENSTEAET

**2001** LAVITAIGNP INEPLFYHIL NPDRRFKISR **TSGVLSTTGT PFDR**EQQEAF

**2051** DVVVEVTEEH KPSAVAHVVV K**VIVEDQNDN APVFVNLPYY AVVK**VDTEVG

**2101** HVIRYVTAVD RDSGRNGEVH YYLKEHHEHF QIGPLGEISL KKQFELDTLN

**2151** KEYLVTVVAK **DGGNPAFSAE VIVPITVMNK** AMPVFEKPFY SAEIAESIQV

**2201** HSPVVHVQAN SPEGLKVFYS ITDGDPFSQF TINFNTGVIN VIAPLDFEAH

**2251** PAYKLSIRAT DSLTGAHAEV FVDIIVDDIN DNPPVFAQQS YAVTLSEASV

**2301** IGTSVVQVRA TDSDSEPNRG ISYQMFGNHS KSHDHFHVDS STGLISLLRT

**2351** LDYEQSRQHT IFVRAVDGGM PTLSSDVIVT VDVTDLNDNP PLFEQQIYEA

**2401** R**ISEHAPHGH FVTCVKAYDA DSSDIDKLQY SILSGNDHKH FVIDSATGII**

**2451 TLSNLHR**HAL KPFYSLNLSV SDGVFRSSTQ VHVTVIGGNL HSPAFLQNEY

**2501** EVELAENAPL HTLVMEVK**TT DGDSGIYGHV TYHIVNDFAK** DRFYINER**GQ**

**2551 IFTLEK**LDRE TPAEKVISVR LMAKDAGGKV AFCTVNVILT DDNDNAPQFR

**2601** ATK**YEVNIGS SAAK**GTSVVK **VLASDADEGS NADITYAIEA DSESVKENLE**

**2651 INK**LSGVITT KESLIGLENE FFTFFVRAVD NGSPSKESVV LVYVKILPPE

**2701** MQLPK**FSEPF YTFTVSEDVP IGTEIDLIR**A EHSGTVLYSL VKGNTPESNR

**2751** DESFVIDRQS GRLKLEKSLD HETTK**WYQFS ILAR**CTQDDH EMVASVDVSI

**2801** QVK**DANDNSP VFESSPYEAF IVENLPGGSR** VIQIRASDAD SGTNGQVMYS

**2851** LDQSQSVEVI ESFAINMETG WITTLKELDH EKRDNYQIKV VASDHGEKIQ

**2901** LSSTAIVDVT VTDVNDSPPR FTAEIYKGTV SEDDPQGGVI AILSTTDADS

**2951** EEINR**QVTYF ITGGDPLGQF AVETIQNEWK** VYVKKPLDRE KRDNYLLTIT

**3001** ATDGTFSSKA IVEVKVLDAN DNSPVCEK**TL YSDTIPEDVL PGKLIMQISA**

**3051 TDADIRSNAE ITYTLLGSGA EK**FKLNPDTG ELK**TSTPLDR EEQAVYHLLV**

**3101 R**ATDGGGRFC QASIVLTLED VNDNAPEFSA DPYAITVFEN TEPGTLLTRV

**3151** QATDADAGLN RKILYSLIDS ADGQFSINEL SGIIQLEKPL DR**ELQAVYTL**

**3201 SLK**AVDQGLP RRLTATGTVI VSVLDINDNP PVFEYREYGA TVSEDILVGT

**3251** EVLQVYAASR DIEANAEITY SIISGNEHGK FSIDSKTGAV FIIENLDYES

**3301** SHEYYLTVEA TDGGTPSLSD VATVNVNVTD INDNTPVFSQ DTYTTVISED

**3351** AVLEQSVITV MADDADGPSN SHIHYSIIDG NQGSSFTIDP VRGEVKVTKL

**3401** LDRETISGYT LTVQASDNGS PPRVNTTTVN IDVSDVNDNA PVFSRGNYSV

**3451** IIQENKPVGF SVLQLVVTDE DSSHNGPPFF FTIVTGNDEK **AFEVNPQGVL**

**3501 LTSSAIK**RKE KDHYLLQVKV ADNGKPQLSS LTYIDIR**VIE ESIYPPAILP**

**3551 LEIFITSSGE EYSGGVIGKI HATDQDVYDT LTYSLDPQMD NLFSVSSTGG**

**3601 K**LIAHKKLDI GQYLLNVSVT DGKFTTVADI TVHIRQVTQE MLNHTIAIR**F**

**3651 ANLTPEEFVG DYWR**NFQRAL RNILGVRRND IQIVSLQSSE PHPHLDVLLF

**3701** VEKPGSAQIS TKQLLHKINS SVTDIEEIIG VRILNVFQK**L CAGLDCPWK**F

**3751** CDEK**VSVDES VMSTHSTARL SFVTPR**HHRA AVCLCKEGRC PPVHHGCEDD

**3801** PCPEGSECVS DPWEEKHTCV CPSGR**FGQCP GSSSMTLTGN SYVK**YRLTEN

**3851** ENKLEMKLTM RLR**TYSTHAV VMYAR**GTDYS ILEIHHGRLQ YKFDCGSGPG

**3901** IVSVQSIQVN DGQWHAVALE VNGNYAR**LVL DQVHTASGTA PGTLKTLNLD**

**3951 NYVFFGGHIR** QQGTRHGRSP QVGNGFRGCM DSIYLNGQEL PLNSKPRSYA

**4001** HIEESVDVSP GCFLTATEDC ASNPCQNGGV CNPSPAGGYY CKCSALYIGT

**4051** HCEISVNPCS SKPCLYGGTC VVDNGGFVCQ CRGLYTGQRC QLSPYCKDEP

**4101** CKNGGTCFDS LDGAVCQCDS GFRGERCQSD IDECSGNPCL HGALCENTHG

**4151** SYHCNCSHEY RGRHCEDAAP NQYVSTPWNI GLAEGIGIVV FVAGIFLLVV

**4201** VFVLCRKMIS RKKKHQAEPK DKHLGPATAF LQRPYFDSKL NKNIYSDIPP

**4251** QVPVRPISYT PSIPSDSRNN LDRNSFEGSA IPEHPEFSTF NPESVHGHRK

**4301** AVAVCSVAPN LPPPPPSNSP SDSDSIQKPS WDFDYDTKVV DLDPCLSKKP

**4351** LEEKPSQPYS ARESLSEVQS LSSFQSESCD DNGYHWDTSD WMPSVPLPDI

**4401** QEFPNYEVID EQTPLYSADP NAIDTDYYPG GYDIESDFPP PPEDFPAADE

**4451** LPPLPPEFSN QFESIHPPRD MPAAGSLGSS SRNRQRFNLN QYLPNFYPLD

**4501** MSEPQTKGTG ENSTCREPHA PYPPGYQRHF EAPAVESMPM SVYASTASCS

**4551** DVSACCEVES EVMMSDYESG DDGHFEEVTI PPLDSQQHTE VJDGSQRLEQ

**4601** TPLQFTHLEY NVTVQENSAA KJGTVKEDAP VGSLVMTVSA HDEDAGRDGE

**4651** IRJGTVKEDA PVGSLVMTVS AHDEDARRDG EIRJLDREST SHYWLTVFAT

**4701** DQGVVPLSSF IEIYIEVEDV NDNAPQTSEP VYYPEIMENS PKDVSVVQIE

**4751** AFDPDSSSND KJLDRESTSH YWLTVFATDQ GVVPLSSFIE LYIEVEDVND

**4801** NAPQTSEPVY YPEIMENSPK DVSVVQIEAF DPDSSSNDKJ FYKIRLPERJ

**4851** REPLYHVIAT DKDEGPNAEI SYSIEDGNEH GKJREPLYRV IATDKDEGPN

**4901** AEISYSIEDG NEHGKJEPLY HVIATDKDEG LNAEISYSIE DGNEHGKFFI

**4951** EPKJEPLYHV IATDKDEGPN AEISYSIEDG NEHGKFFIEP KJALEKNTNL

**5001** EARTKJALEK NTNVEARTKJ ALEKNTNVEA RTKJALEKNT NVEVRTKJFS

**5051** AAGEYDILSI KAVDNGRPQK SSTTRJFSAA GEYDILSIKA VDSGRPQKSS

**5101** TTRJNFARIV VNVSDTNDHA PWFTASSYKG RJNFARIVVN VSDTNDHAPW

**5151** FTTSSYKGRJ GKNAEVLYSI ESGDFGNIGN SFMIDPVLGS IKTAKJGKNA

**5201** EVLYSIESGN FGNIGNSFMI DPVLGSIKTA KJGKNAEVLY SIESGTIGNI

**5251** GNSFMIDPVL GSIKTAKJGS PPMSEITSVR IFVTNADNAS PKFTSKJGSP

**5301** PMSEITSVRI FVTSADNASP KFTSKJGSPP MSEITSVRIF VTIADNASTK

**5351** FTSKJALDFE TLPIYTLIIQ GTNMAGLSTN TTVLVHLQDE NDNAPVFMQA

**5401** EYTGLISESA SINSVVLTDR NVPQVIRAAD ADKJALDFET LPIYTLIIQG

**5451** TNMAGLSTNT TVLVHLQDEN DNAPVFMQAE YTGLISESAS INSVVLTDRN

**5501** VPRVIRAADA DKJAADADKD SNVLLVYHIV EPSVHTYFAI DSSTGAIHTV

**5551** LSLDYEETSI FHFTVQVHDM GTPRLFAEYA ANVTVHVIDI NDCPPVFAKP

**5601** LYEASLLLPT YKJFTQDVYS AVVKTNSTEA ETLAVITAIG NPINEPLFYH

**5651** ILNPDRRJTS GVLSTTGTPF DREAQEAFDV VVEVTEEHKP SAVAHVVVKV

**5701** IVEDQNDNAP VFVNLPYYAV VKJTDMFAIH PTSGVIVLTG RLDFLETKLY

**5751** EMEILAADRJ TDMFAIHPTS GVIVLTGRLD YLETKLYEME ILAADRJNGE

**5801** VHYYLKEHHE HFQIGPLGEK SLKKJDGGNP AFSAEVIVPI TVMNKAMPVF

**5851** EKPFYSAEIA ESIQVHSHVV HVQANSPEGL KVFYSITDGD PFSQFTINFN

**5901** TGVINVIAPL DFEAHPAYKJ AMPVFEKPFY SAEIAESIQV HSPVVHVQAN

**5951** SPEGLKVFYS ITDGDPFSQF TINFNTGVIN VIAPLDFPAH PAYKLSIRJL

**6001** SIRATDSLTG AHAEVFVDDI VDDINDNPPV FAQQSYAVTL SEASVIGTSV

**6051** VQVRATDSDS EPNRJLSIRA TDSLTGAHAE VFVDEIVDDI NDNPPVFAQQ

**6101** SYAVTLSEAS VIGTSVVQVR ATDSDSEPNR JTLDYEQSRQ HTIAVRAVDG

**6151** GMPTLSSDVI VTVDVTDLND NPPLFEQQIY EARJLYGSSG ISSMAKLTVH

**6201** IEQANECAPV ITAVTLSPSE LDRDPAYAIV TVDDCDQGAN GDIASLSIVA

**6251** GDLLQQFRJI LPPEMQLPKF SEPFYTFTVS EIVPIGTEID LIRAEHSGTV

**6301** LYSLVKJILP PEMQLPKFSE PFYTFTVSEV VPIGTEIDLI RAEHSGTVLY

**6351** SLVKJCTQDD HEMVASVDVS IQVKDANDNS PVFESSPYEA FIVENLPGGS

**6401** RVIQIRJCTQ DDHEMVASVD VSIQVKDASD NSPVFESSPY EAFIVENLPG

**6451** GSRVIQIRJV IQIRASDADN GTNGQVMYSL DQSQSVEVIE SFAINMETGW

**6501** ITTLKELDHE KJFTAEIYKG TVSELDPQGG VIAILSTTDA DSEEINRQVT

**6551** YFITGGDPLG QFAVETIQNE WKJFTAEIYK GTVSEPDPQG GVIAILSTTD

**6601** ADSEEINRQV TYFITGGDPL GQFAVETIQN EWKJFTAEIY KGTVSEQDPQ

**6651** GGVIAILSTT DADSEEINRQ VTYFITGGDP LGQFAVETIQ NEWKJGTVSE

**6701** DDPQGGVIAI LSTTDADSEE INRQVTYGIT GGDPLGQFAV ETIQNEWKVY

**6751** VKJGTVSEDD PQGGVIAILS TTDADSEEIN RQVTYSITGG DPLGQFAVET

**6801** IQNEWKVYVK JGTVSEDDPQ GGVIAILSTT DADSEEINRQ VTYFITGGDP

**6851** LAQFAVETIQ NEWKVYVKJA IVEVKVLDAN DNSPVCEYTL YSDTIPEDVL

**6901** PGKLIMQISA TDADIRJVLD ANDNSPVCEK TLYIDTIPED VLPGKLIMQI

**6951** SATDADIRJV LDANDNSPVC EKTLYTDTIP EDVLPGKLIM QISATDADIR

**7001** JATDGGGRFC QASIVLDLED VNDNAPEFSA DPYAITVFEN TEPGTLLTRV

**7051** QATDADAGLN RJKILYSLID SADGQFSINE LIGIIQLEKP LDRELQAVYT

**7101** LSLKJFSIDS KTGAVFIIEN LDYESSHEYY LTVEATDGGT PSLSDVATVN

**7151** VNVTDINDAT PVFSQDTYTT VISEDAVLEQ SVITVMADDA DGPSNSHIHY

**7201** SIIDGNQGSS FTIDPVRGEV KJFSIDSKTG AVFIIENLDY ESSHEYYLTV

**7251** EATDGGTPSL SDVATVNVNV TDINDVTPVF SQDTYTTVIS EDAVLEQSVI

**7301** TVMADDADGP SNSHIHYSII DGNQGSSFTI DPVRGEVKJV NTTTVNIDVS

**7351** DVNDNAPVFS RGNYSVIIQE NKPVGFSVLQ LVVTDEDSSH NGPPFFFTIV

**7401** TENDEKAFEV NPQGVLLTSS AIKJVNTTTV NIDVSDVNDN APVFSRGNYS

**7451** VIIQENKPVG FSVLQLVVTD EDSSHNGPPF FFTIVTGNDE KAFEVNPQGV

**7501** LLTSSAIKJG NYSVIIQENK PVGFSVLQLV VTDEDSSHNG PPFFFTIVTG

**7551** NDEKAFEVNA QGVLLTSSAI KRJGNYSVII QENKPVGFSV LQLVVTDEDS

**7601** SHNGPPFFFT IVTGNDEKAF EVNVQGVLLT SSAIKRJVAD NGKPQLSSLT

**7651** YIDIRVIEES IYPPAILPLE IAITSSGEEY SGGVIGKIHA TDQDVYDTLT

**7701** YSLDPQMDNL FSVSSTGGKJ VADNGKPQLS SLTYIDIRVI EESIYPPAIL

**7751** PLEISITSSG EEYSGGVIGK IHATDQDVYD TLTYSLDPQM DNLFSVSSTG

**7801** GKJVIHVTSP QFKAGPVKFE KJQLLHKIMS SVTDIEEIIG VRILNVFQKJ

**7851** QLLHKIVSSV TDIEEIIGVR ILNVFQKJEG RCPPVHHGCE DHPCPEGSEC

**7901** VSDPWEEKHT CVCPSGRJEG RCPPVHHGCE DPPCPEGSEC VSDPWEEKHT

**7951** CVCPSGRJHT CVCPSGRFGG CPGSSSMTLT GNSYVKYRJH TCVCPSGRFG

**8001** RCPGSSSMTL TGNSYVKYRJ FGQCPGSSSM TLTGNSYVKT RLTENENKJG

**8051** CMDSIYLNGQ ELPLNSKPRS YAHIEESVPV SPGCFLTATE DCASNPCQNG

**8101** GVCNPSPAGG YYCKCSALYI GTHCEISVNP CSSKPCLYGG TCVVDNGGFV

**8151** CQCRJYVFKR TPGKAKJYVF KSTPGKAKJS YAHIEESVDV SPGCFLTATE

**8201** DCASNPCQNG GVCNPSPAGG YYCKCSALYI GTHCEISVNP KSSKPCLYGG

**8251** TCVVDNGGFV CQCRGLYTGQ RJSYAHIEES VDVSPGCFLT ATEDCASNPC

**8301** QNGGVCNPSP AGGYYCKCSA LYIGTHCEIS VNPNSSKPCL YGGTCVVDNG

**8351** GFVCQCRGLY TGQRJDEPCK NGGTCFDSAD GAVCQCDSGF RGERJGRHCA

**8401** DAAPNQYVST PWNIGLAEGI GIVVFVAGIF LLVVVFVLCR KJGRHCEDAA

**8451** PNQYVSTPWN IGLAEGIGIV VFVAGIFLLV VVFVLCRKJN IYSDIPPQVP

**8501** VRYISYTPSI PSDSRNNLDR JKAVAVCSVA PNLPPPPPSN SPSDSDSIQK

**8551** PSWDFDYDTK VVDLDPCLSK JKPLEEKPSQ PYSARESLSS VQSLSSFQSE

**8601** SCDDNGYHWD TSDWMPSVPL PDIQEFPNYE VIDEQTPLYS ADPNAIDTDY

**8651** YPGGYDIESD FPPPPEDFPA ADELPPLPPE FSNQFESIHP PRDMPAAGSL

**8701** GSSSRJKPLE EKPSQPYSAR ESLSEVQSLS SFQSESCDDN GYHWDTSDWM

**8751** PSVPLQDIQE FPNYEVIDEQ TPLYSADPNA IDTDYYPGGY DIESDFPPPP

**8801** EDFPAADELP PLPPEFSNQF ESIHPPRDMP AAGSLGSSSR JKPLEEKPSQ

**8851** PYSARESLSE VQSLSSFQSE SCDDNGYHWD TSDWMPSVPL PDIQEFPNYE

**8901** VIDPQTPLYS ADPNAIDTDY YPGGYDIESD FPPPPEDFPA ADELPPLPPE

**8951** FSNQFESIHP PRDMPAAGSL GSSSRJESLS EVQSLSSFQS ESCDDNGYHW

**9001** DTSDWMPSVP LPDIQEFPNY EVIDEQTPLY SADPNAIDTD YYPGGYDIES

**9051** DFPPPPEDFP AADELPPLPP EFSNQFESIH PPRDMPAAGS LGSSRRNRJE

**9101** SLSEVQSLSS FQSESCDDNG YHWDTSDWMP SVPLPDIQEF PNYEVIDEQT

**9151** PLYSADPNAI DTDYYPGGYD IESDFPPPPE DFPAADELPP LPPEFSNQFE

**9201** SIHPPRDMPA AGSLGSSWRN RJVLGANSNP PEFTQTAYKA AFDENVPIGT

**9251** TIMSLSAVDP DEGENGYVTY SIANLNHVPF AIDHFTGAVS TSENLDYELM

**9301** PRVYTLRJVL GANSNPPEFT QTAYKAAFDE NVPIGTTVMS LSAVDPDEGE

**9351** NGYVTYSIAN LNHVPFAIDH FTGAVSTSEN LDYELMPRVY TLRJVLGANS

**9401** NPPEFTQTAY KAAFDENVPI GTTVMSLSAV DPDEGENGYV TYSIANLNHV

**9451** PFAIDHFTGA VSTSENLDYE LMPRVYTLRJ INCEGTIPRD LGVGEQITTV

**9501** SAIDADELQL VQYQIEAGNE LDFFSLNPNS GVLSLKRJIN CEGTIPRDLG

**9551** VGEQITTVSA IDADELQLVQ YQIEAGNELD LFSLNPNSGV LSLKRJINCE

**9601** GTIPRDLGVG EQITTVSAID ADELQLVQYQ IEAGNELDFF SLNPNSGVLS

**9651** LKRJEVHSEI IQVEATDKDL GPNGHVTYSI LTDTDTFSID SVTGVVNIAR

**9701** PLDRELQHEH SLKJEVHSEI IQVEATDKDL GPNGHVTYSI VTDTDTFSID

**9751** SVTGVVNIAR PLDRELQHEH SLK

**Start - End Observed Mr(expt) Mr(calc) ppm Miss Sequence**

**85 - 97 816.8888 1631.7630 1631.7654 -1 0 K.AEEYILGDFCFLR.I**  ([Ions score 49](http://mbp-mascot4/mascot/cgi/peptide_view.pl?file=../data/20091021/F006305.dat&query=3842&hit=1&index=IPI00031411&px=1&section=5&ave_thresh=22))

**85 - 97 816.8892 1631.7638 1631.7654 -1 0 K.AEEYILGDFCFLR.I**  ([Ions score 60](http://mbp-mascot4/mascot/cgi/peptide_view.pl?file=../data/20091021/F006305.dat&query=3843&hit=1&index=IPI00031411&px=1&section=5&ave_thresh=22))

**137 - 165 1078.2311 3231.6714 3231.6776 -2 0 R.VQVLDTNDLRPLFSPTSYSVSLPENTAIR.T**  ([Ions score 38](http://mbp-mascot4/mascot/cgi/peptide_view.pl?file=../data/20091021/F006305.dat&query=5327&hit=1&index=IPI00031411&px=1&section=5&ave_thresh=22))

**192 - 209 639.0081 1914.0023 1914.0033 -1 0 R.TDMFAIHPTSGVIVLTGR.L**  ([Ions score 23](http://mbp-mascot4/mascot/cgi/peptide_view.pl?file=../data/20091021/F006305.dat&query=4414&hit=1&index=IPI00031411&px=1&section=5&ave_thresh=22))

**217 - 227 662.3338 1322.6530 1322.6540 -1 0 K.LYEMEILAADR.G**  ([Ions score 56](http://mbp-mascot4/mascot/cgi/peptide_view.pl?file=../data/20091021/F006305.dat&query=3113&hit=1&index=IPI00031411&px=1&section=5&ave_thresh=22))

**231 - 242 600.8001 1199.5857 1199.5856 0 0 K.LYGSSGISSMAK.L**  ([Ions score 58](http://mbp-mascot4/mascot/cgi/peptide_view.pl?file=../data/20091021/F006305.dat&query=2480&hit=1&index=IPI00031411&px=1&section=5&ave_thresh=22))

**231 - 242 600.8008 1199.5871 1199.5856 1 0 K.LYGSSGISSMAK.L**  ([Ions score 71](http://mbp-mascot4/mascot/cgi/peptide_view.pl?file=../data/20091021/F006305.dat&query=2481&hit=1&index=IPI00031411&px=1&section=5&ave_thresh=22))

**243 - 269 988.5088 2962.5047 2962.5070 -1 0 K.LTVHIEQANECAPVITAVTLSPSELDR.D**  ([Ions score 23](http://mbp-mascot4/mascot/cgi/peptide_view.pl?file=../data/20091021/F006305.dat&query=5237&hit=1&index=IPI00031411&px=1&section=5&ave_thresh=22))

**243 - 269 988.5089 2962.5049 2962.5070 -1 0 K.LTVHIEQANECAPVITAVTLSPSELDR.D**  ([Ions score 28](http://mbp-mascot4/mascot/cgi/peptide_view.pl?file=../data/20091021/F006305.dat&query=5238&hit=1&index=IPI00031411&px=1&section=5&ave_thresh=22))

**352 - 361 578.3294 1154.6442 1154.6448 -0 0 K.VIHVTSPQFK.A**  ([Ions score 32](http://mbp-mascot4/mascot/cgi/peptide_view.pl?file=../data/20091021/F006305.dat&query=2253&hit=1&index=IPI00031411&px=1&section=5&ave_thresh=22))

**374 - 390 914.9793 1827.9441 1827.9441 -0 0 R.AEISEFAPPNTPVVMVK.A**  ([Ions score 49](http://mbp-mascot4/mascot/cgi/peptide_view.pl?file=../data/20091021/F006305.dat&query=4237&hit=1&index=IPI00031411&px=1&section=5&ave_thresh=22))

**411 - 427 954.5265 1907.0385 1907.0404 -1 0 K.FSLNYNTGLISILEPVK.R**  ([Ions score 80](http://mbp-mascot4/mascot/cgi/peptide_view.pl?file=../data/20091021/F006305.dat&query=4405&hit=1&index=IPI00031411&px=1&section=5&ave_thresh=22))

**453 - 469 918.9582 1835.9018 1835.9054 -2 0 K.VLGANSNPPEFTQTAYK.A**  ([Ions score 37](http://mbp-mascot4/mascot/cgi/peptide_view.pl?file=../data/20091021/F006305.dat&query=4267&hit=1&index=IPI00031411&px=1&section=5&ave_thresh=22))

**453 - 469 918.9594 1835.9043 1835.9054 -1 0 K.VLGANSNPPEFTQTAYK.A**  ([Ions score 73](http://mbp-mascot4/mascot/cgi/peptide_view.pl?file=../data/20091021/F006305.dat&query=4268&hit=1&index=IPI00031411&px=1&section=5&ave_thresh=22))

**540 - 548 532.7621 1063.5096 1063.5087 1 0 R.ASDWGLPYR.R**  ([Ions score 45](http://mbp-mascot4/mascot/cgi/peptide_view.pl?file=../data/20091021/F006305.dat&query=1740&hit=1&index=IPI00031411&px=1&section=5&ave_thresh=22))

**540 - 548 532.7622 1063.5099 1063.5087 1 0 R.ASDWGLPYR.R**  ([Ions score 36](http://mbp-mascot4/mascot/cgi/peptide_view.pl?file=../data/20091021/F006305.dat&query=1741&hit=1&index=IPI00031411&px=1&section=5&ave_thresh=22))

**669 - 681 730.8747 1459.7348 1459.7341 1 0 K.LVNLQCEETGVAK.M**  ([Ions score 60](http://mbp-mascot4/mascot/cgi/peptide_view.pl?file=../data/20091021/F006305.dat&query=3521&hit=1&index=IPI00031411&px=1&section=5&ave_thresh=22))

**718 - 727 522.3089 1042.6032 1042.6023 1 0 R.STLPTGIQVK.E**  ([Ions score 46](http://mbp-mascot4/mascot/cgi/peptide_view.pl?file=../data/20091021/F006305.dat&query=1626&hit=1&index=IPI00031411&px=1&section=5&ave_thresh=22))

**903 - 914 688.3765 1374.7385 1374.7395 -1 0 R.EEPQLFSTVVVK.V**  ([Ions score 39](http://mbp-mascot4/mascot/cgi/peptide_view.pl?file=../data/20091021/F006305.dat&query=3266&hit=1&index=IPI00031411&px=1&section=5&ave_thresh=22))

**903 - 914 688.3769 1374.7392 1374.7395 -0 0 R.EEPQLFSTVVVK.V**  ([Ions score 35](http://mbp-mascot4/mascot/cgi/peptide_view.pl?file=../data/20091021/F006305.dat&query=3267&hit=1&index=IPI00031411&px=1&section=5&ave_thresh=22))

**915 - 933 1094.0395 2186.0645 2186.0644 0 0 K.VSLEDVNDNPPTFIPPNYR.V**  ([Ions score 34](http://mbp-mascot4/mascot/cgi/peptide_view.pl?file=../data/20091021/F006305.dat&query=4669&hit=1&index=IPI00031411&px=1&section=5&ave_thresh=22))

**964 - 978 570.2654 1707.7745 1707.7740 0 0 R.YSLLDHGEGNFDVDK.L**  ([Ions score 25](http://mbp-mascot4/mascot/cgi/peptide_view.pl?file=../data/20091021/F006305.dat&query=4001&hit=1&index=IPI00031411&px=1&section=5&ave_thresh=22))

**985 - 994 624.3535 1246.6925 1246.6921 0 1 R.IVQQLDFEKK.Q**  ([Ions score 30](http://mbp-mascot4/mascot/cgi/peptide_view.pl?file=../data/20091021/F006305.dat&query=2683&hit=1&index=IPI00031411&px=1&section=5&ave_thresh=22))

**1084 - 1096 703.3428 1404.6710 1404.6732 -2 0 K.IGEETGVIETSDR.L**  ([Ions score 54](http://mbp-mascot4/mascot/cgi/peptide_view.pl?file=../data/20091021/F006305.dat&query=3366&hit=1&index=IPI00031411&px=1&section=5&ave_thresh=22))

**1084 - 1096 703.3439 1404.6733 1404.6732 0 0 K.IGEETGVIETSDR.L**  ([Ions score 78](http://mbp-mascot4/mascot/cgi/peptide_view.pl?file=../data/20091021/F006305.dat&query=3367&hit=1&index=IPI00031411&px=1&section=5&ave_thresh=22))

**1178 - 1192 543.9510 1628.8313 1628.8311 0 0 K.ITSGNPQGFFSIHPK.T**  ([Ions score 38](http://mbp-mascot4/mascot/cgi/peptide_view.pl?file=../data/20091021/F006305.dat&query=3836&hit=1&index=IPI00031411&px=1&section=5&ave_thresh=22))

**1235 - 1248 851.4437 1700.8729 1700.8733 -0 0 K.ILDENDNKPQFLQK.F**  ([Ions score 26](http://mbp-mascot4/mascot/cgi/peptide_view.pl?file=../data/20091021/F006305.dat&query=3986&hit=1&index=IPI00031411&px=1&section=5&ave_thresh=22))

**1235 - 1248 567.9652 1700.8738 1700.8733 0 0 K.ILDENDNKPQFLQK.F**  ([Ions score 38](http://mbp-mascot4/mascot/cgi/peptide_view.pl?file=../data/20091021/F006305.dat&query=3987&hit=1&index=IPI00031411&px=1&section=5&ave_thresh=22))

**1235 - 1248 567.9653 1700.8742 1700.8733 0 0 K.ILDENDNKPQFLQK.F**  ([Ions score 31](http://mbp-mascot4/mascot/cgi/peptide_view.pl?file=../data/20091021/F006305.dat&query=3988&hit=1&index=IPI00031411&px=1&section=5&ave_thresh=22))

**1314 - 1326 707.3659 1412.7172 1412.7187 -1 0 R.FSAAGEYDILSIK.A**  ([Ions score 62](http://mbp-mascot4/mascot/cgi/peptide_view.pl?file=../data/20091021/F006305.dat&query=3387&hit=1&index=IPI00031411&px=1&section=5&ave_thresh=22))

**1314 - 1326 707.3664 1412.7182 1412.7187 -0 0 R.FSAAGEYDILSIK.A**  ([Ions score 68](http://mbp-mascot4/mascot/cgi/peptide_view.pl?file=../data/20091021/F006305.dat&query=3388&hit=1&index=IPI00031411&px=1&section=5&ave_thresh=22))

**1407 - 1422 820.9639 1639.9132 1639.9145 -1 0 K.GTGTIIVAKPLDAEQK.S**  ([Ions score 48](http://mbp-mascot4/mascot/cgi/peptide_view.pl?file=../data/20091021/F006305.dat&query=3877&hit=1&index=IPI00031411&px=1&section=5&ave_thresh=22))

**1446 - 1460 582.2922 1743.8549 1743.8540 0 0 K.VIDTNDHRPQFSTSK.Y**  ([Ions score 29](http://mbp-mascot4/mascot/cgi/peptide_view.pl?file=../data/20091021/F006305.dat&query=4053&hit=1&index=IPI00031411&px=1&section=5&ave_thresh=22))

**1489 - 1497 540.8060 1079.5975 1079.5975 0 0 K.LIYTLQSSR.D**  ([Ions score 38](http://mbp-mascot4/mascot/cgi/peptide_view.pl?file=../data/20091021/F006305.dat&query=1806&hit=1&index=IPI00031411&px=1&section=5&ave_thresh=22))

**1505 - 1519 562.2897 1683.8474 1683.8468 0 1 K.FRLDPATGSLYTSEK.L**  ([Ions score 42](http://mbp-mascot4/mascot/cgi/peptide_view.pl?file=../data/20091021/F006305.dat&query=3964&hit=1&index=IPI00031411&px=1&section=5&ave_thresh=22))

**1507 - 1519 691.3466 1380.6787 1380.6773 1 0 R.LDPATGSLYTSEK.L**  ([Ions score 34](http://mbp-mascot4/mascot/cgi/peptide_view.pl?file=../data/20091021/F006305.dat&query=3278&hit=1&index=IPI00031411&px=1&section=5&ave_thresh=22))

**1520 - 1535 629.3273 1884.9600 1884.9629 -2 0 K.LDHEAVHQHTLTVMVR.D**  ([Ions score 26](http://mbp-mascot4/mascot/cgi/peptide_view.pl?file=../data/20091021/F006305.dat&query=4369&hit=1&index=IPI00031411&px=1&section=5&ave_thresh=22))

**1631 - 1641 649.3084 1296.6023 1296.6020 0 0 R.SNQAEYDLMVK.A**  ([Ions score 45](http://mbp-mascot4/mascot/cgi/peptide_view.pl?file=../data/20091021/F006305.dat&query=2928&hit=1&index=IPI00031411&px=1&section=5&ave_thresh=22))

**1646 - 1657 630.8160 1259.6174 1259.6180 -0 0 K.GSPPMSEITSVR.I**  ([Ions score 39](http://mbp-mascot4/mascot/cgi/peptide_view.pl?file=../data/20091021/F006305.dat&query=2752&hit=1&index=IPI00031411&px=1&section=5&ave_thresh=22))

**1646 - 1657 630.8160 1259.6174 1259.6180 -0 0 K.GSPPMSEITSVR.I**  ([Ions score 35](http://mbp-mascot4/mascot/cgi/peptide_view.pl?file=../data/20091021/F006305.dat&query=2753&hit=1&index=IPI00031411&px=1&section=5&ave_thresh=22))

**1646 - 1657 638.8134 1275.6121 1275.6129 -1 0 K.GSPPMSEITSVR.I**  Oxidation (M) ([Ions score 28](http://mbp-mascot4/mascot/cgi/peptide_view.pl?file=../data/20091021/F006305.dat&query=2841&hit=1&index=IPI00031411&px=1&section=5&ave_thresh=22))

**1658 - 1669 638.3509 1274.6872 1274.6870 0 0 R.IFVTIADNASPK.F**  ([Ions score 52](http://mbp-mascot4/mascot/cgi/peptide_view.pl?file=../data/20091021/F006305.dat&query=2839&hit=1&index=IPI00031411&px=1&section=5&ave_thresh=22))

**1658 - 1669 638.3512 1274.6878 1274.6870 1 0 R.IFVTIADNASPK.F**  ([Ions score 58](http://mbp-mascot4/mascot/cgi/peptide_view.pl?file=../data/20091021/F006305.dat&query=2840&hit=1&index=IPI00031411&px=1&section=5&ave_thresh=22))

**1706 - 1726 734.3540 2200.0402 2200.0397 0 0 K.DGNTGDAFDINPHSGTIITQK.A**  ([Ions score 53](http://mbp-mascot4/mascot/cgi/peptide_view.pl?file=../data/20091021/F006305.dat&query=4690&hit=1&index=IPI00031411&px=1&section=5&ave_thresh=22))

**1962 - 1969 411.7401 821.4657 821.4647 1 0 R.FAGLTSVK.I**  ([Ions score 33](http://mbp-mascot4/mascot/cgi/peptide_view.pl?file=../data/20091021/F006305.dat&query=368&hit=1&index=IPI00031411&px=1&section=5&ave_thresh=22))

**2031 - 2044 719.8619 1437.7092 1437.7100 -1 0 R.TSGVLSTTGTPFDR.E**  ([Ions score 79](http://mbp-mascot4/mascot/cgi/peptide_view.pl?file=../data/20091021/F006305.dat&query=3462&hit=1&index=IPI00031411&px=1&section=5&ave_thresh=22))

**2072 - 2094 869.4534 2605.3384 2605.3428 -2 0 K.VIVEDQNDNAPVFVNLPYYAVVK.V**  ([Ions score 34](http://mbp-mascot4/mascot/cgi/peptide_view.pl?file=../data/20091021/F006305.dat&query=5046&hit=1&index=IPI00031411&px=1&section=5&ave_thresh=22))

**2072 - 2094 869.4536 2605.3390 2605.3428 -1 0 K.VIVEDQNDNAPVFVNLPYYAVVK.V**  ([Ions score 30](http://mbp-mascot4/mascot/cgi/peptide_view.pl?file=../data/20091021/F006305.dat&query=5047&hit=1&index=IPI00031411&px=1&section=5&ave_thresh=22))

**2161 - 2180 1030.0297 2058.0448 2058.0456 -0 0 K.DGGNPAFSAEVIVPITVMNK.A**  ([Ions score 77](http://mbp-mascot4/mascot/cgi/peptide_view.pl?file=../data/20091021/F006305.dat&query=4558&hit=1&index=IPI00031411&px=1&section=5&ave_thresh=22))

**2402 - 2416 573.6198 1717.8376 1717.8359 1 0 R.ISEHAPHGHFVTCVK.A**  ([Ions score 37](http://mbp-mascot4/mascot/cgi/peptide_view.pl?file=../data/20091021/F006305.dat&query=4017&hit=1&index=IPI00031411&px=1&section=5&ave_thresh=22))

**2417 - 2439 852.4011 2554.1815 2554.1823 -0 1 K.AYDADSSDIDKLQYSILSGNDHK.H**  ([Ions score 51](http://mbp-mascot4/mascot/cgi/peptide_view.pl?file=../data/20091021/F006305.dat&query=5020&hit=1&index=IPI00031411&px=1&section=5&ave_thresh=22))

**2440 - 2457 665.3652 1993.0737 1993.0745 -0 0 K.HFVIDSATGIITLSNLHR.H**  ([Ions score 67](http://mbp-mascot4/mascot/cgi/peptide_view.pl?file=../data/20091021/F006305.dat&query=4517&hit=1&index=IPI00031411&px=1&section=5&ave_thresh=22))

**2440 - 2457 665.3663 1993.0770 1993.0745 1 0 K.HFVIDSATGIITLSNLHR.H**  ([Ions score 70](http://mbp-mascot4/mascot/cgi/peptide_view.pl?file=../data/20091021/F006305.dat&query=4518&hit=1&index=IPI00031411&px=1&section=5&ave_thresh=22))

**2519 - 2540 804.0480 2409.1223 2409.1237 -1 0 K.TTDGDSGIYGHVTYHIVNDFAK.D**  ([Ions score 33](http://mbp-mascot4/mascot/cgi/peptide_view.pl?file=../data/20091021/F006305.dat&query=4944&hit=1&index=IPI00031411&px=1&section=5&ave_thresh=22))

**2549 - 2556 468.2635 934.5125 934.5124 0 0 R.GQIFTLEK.L**  ([Ions score 31](http://mbp-mascot4/mascot/cgi/peptide_view.pl?file=../data/20091021/F006305.dat&query=1125&hit=1&index=IPI00031411&px=1&section=5&ave_thresh=22))

**2604 - 2614 569.7912 1137.5678 1137.5666 1 0 K.YEVNIGSSAAK.G**  ([Ions score 27](http://mbp-mascot4/mascot/cgi/peptide_view.pl?file=../data/20091021/F006305.dat&query=2154&hit=1&index=IPI00031411&px=1&section=5&ave_thresh=22))

**2621 - 2646 890.7463 2669.2172 2669.2191 -1 0 K.VLASDADEGSNADITYAIEADSESVK.E**  ([Ions score 39](http://mbp-mascot4/mascot/cgi/peptide_view.pl?file=../data/20091021/F006305.dat&query=5095&hit=1&index=IPI00031411&px=1&section=5&ave_thresh=22))

**2621 - 2653 1170.8899 3509.6478 3509.6532 -2 1 K.VLASDADEGSNADITYAIEADSESVKENLEINK.L**  ([Ions score 95](http://mbp-mascot4/mascot/cgi/peptide_view.pl?file=../data/20091021/F006305.dat&query=5423&hit=1&index=IPI00031411&px=1&section=5&ave_thresh=22))

**2706 - 2729 925.7982 2774.3728 2774.3691 1 0 K.FSEPFYTFTVSEDVPIGTEIDLIR.A**  ([Ions score 42](http://mbp-mascot4/mascot/cgi/peptide_view.pl?file=../data/20091021/F006305.dat&query=5148&hit=1&index=IPI00031411&px=1&section=5&ave_thresh=22))

**2776 - 2784 592.3170 1182.6193 1182.6186 1 0 K.WYQFSILAR.C**  ([Ions score 25](http://mbp-mascot4/mascot/cgi/peptide_view.pl?file=../data/20091021/F006305.dat&query=2389&hit=1&index=IPI00031411&px=1&section=5&ave_thresh=22))

**2804 - 2830 971.1172 2910.3299 2910.3308 -0 0 K.DANDNSPVFESSPYEAFIVENLPGGSR.V**  ([Ions score 54](http://mbp-mascot4/mascot/cgi/peptide_view.pl?file=../data/20091021/F006305.dat&query=5206&hit=1&index=IPI00031411&px=1&section=5&ave_thresh=22))

**2804 - 2830 971.1179 2910.3317 2910.3308 0 0 K.DANDNSPVFESSPYEAFIVENLPGGSR.V**  ([Ions score 32](http://mbp-mascot4/mascot/cgi/peptide_view.pl?file=../data/20091021/F006305.dat&query=5207&hit=1&index=IPI00031411&px=1&section=5&ave_thresh=22))

**2956 - 2980 947.8097 2840.4074 2840.4021 2 0 R.QVTYFITGGDPLGQFAVETIQNEWK.V**  ([Ions score 47](http://mbp-mascot4/mascot/cgi/peptide_view.pl?file=../data/20091021/F006305.dat&query=5176&hit=1&index=IPI00031411&px=1&section=5&ave_thresh=22))

**3029 - 3043 824.4277 1646.8408 1646.8403 0 0 K.TLYSDTIPEDVLPGK.L**  ([Ions score 47](http://mbp-mascot4/mascot/cgi/peptide_view.pl?file=../data/20091021/F006305.dat&query=3885&hit=1&index=IPI00031411&px=1&section=5&ave_thresh=22))

**3044 - 3056 723.8835 1445.7524 1445.7548 -2 0 K.LIMQISATDADIR.S**  ([Ions score 76](http://mbp-mascot4/mascot/cgi/peptide_view.pl?file=../data/20091021/F006305.dat&query=3485&hit=1&index=IPI00031411&px=1&section=5&ave_thresh=22))

**3044 - 3056 731.8805 1461.7465 1461.7497 -2 0 K.LIMQISATDADIR.S**  Oxidation (M) ([Ions score 48](http://mbp-mascot4/mascot/cgi/peptide_view.pl?file=../data/20091021/F006305.dat&query=3530&hit=1&index=IPI00031411&px=1&section=5&ave_thresh=22))

**3057 - 3072 827.4183 1652.8220 1652.8257 -2 0 R.SNAEITYTLLGSGAEK.F**  ([Ions score 106](http://mbp-mascot4/mascot/cgi/peptide_view.pl?file=../data/20091021/F006305.dat&query=3903&hit=1&index=IPI00031411&px=1&section=5&ave_thresh=22))

**3057 - 3072 827.4202 1652.8259 1652.8257 0 0 R.SNAEITYTLLGSGAEK.F**  ([Ions score 114](http://mbp-mascot4/mascot/cgi/peptide_view.pl?file=../data/20091021/F006305.dat&query=3904&hit=1&index=IPI00031411&px=1&section=5&ave_thresh=22))

**3084 - 3101 709.7112 2126.1119 2126.1120 -0 1 K.TSTPLDREEQAVYHLLVR.A**  ([Ions score 46](http://mbp-mascot4/mascot/cgi/peptide_view.pl?file=../data/20091021/F006305.dat&query=4610&hit=1&index=IPI00031411&px=1&section=5&ave_thresh=22))

**3084 - 3101 709.7114 2126.1124 2126.1120 0 1 K.TSTPLDREEQAVYHLLVR.A**  ([Ions score 44](http://mbp-mascot4/mascot/cgi/peptide_view.pl?file=../data/20091021/F006305.dat&query=4611&hit=1&index=IPI00031411&px=1&section=5&ave_thresh=22))

**3084 - 3101 709.7134 2126.1183 2126.1120 3 1 K.TSTPLDREEQAVYHLLVR.A**  ([Ions score 27](http://mbp-mascot4/mascot/cgi/peptide_view.pl?file=../data/20091021/F006305.dat&query=4615&hit=1&index=IPI00031411&px=1&section=5&ave_thresh=22))

**3193 - 3203 632.8603 1263.7060 1263.7074 -1 0 R.ELQAVYTLSLK.A**  ([Ions score 39](http://mbp-mascot4/mascot/cgi/peptide_view.pl?file=../data/20091021/F006305.dat&query=2779&hit=1&index=IPI00031411&px=1&section=5&ave_thresh=22))

**3193 - 3203 632.8604 1263.7063 1263.7074 -1 0 R.ELQAVYTLSLK.A**  ([Ions score 27](http://mbp-mascot4/mascot/cgi/peptide_view.pl?file=../data/20091021/F006305.dat&query=2780&hit=1&index=IPI00031411&px=1&section=5&ave_thresh=22))

**3491 - 3507 887.4887 1772.9629 1772.9672 -2 0 K.AFEVNPQGVLLTSSAIK.R**  ([Ions score 76](http://mbp-mascot4/mascot/cgi/peptide_view.pl?file=../data/20091021/F006305.dat&query=4120&hit=1&index=IPI00031411&px=1&section=5&ave_thresh=22))

**3491 - 3507 887.4897 1772.9648 1772.9672 -1 0 K.AFEVNPQGVLLTSSAIK.R**  ([Ions score 91](http://mbp-mascot4/mascot/cgi/peptide_view.pl?file=../data/20091021/F006305.dat&query=4121&hit=1&index=IPI00031411&px=1&section=5&ave_thresh=22))

**3538 - 3569 1136.5998 3406.7777 3406.7799 -1 0 R.VIEESIYPPAILPLEIFITSSGEEYSGGVIGK.I**  ([Ions score 29](http://mbp-mascot4/mascot/cgi/peptide_view.pl?file=../data/20091021/F006305.dat&query=5414&hit=1&index=IPI00031411&px=1&section=5&ave_thresh=22))

**3570 - 3601 1173.5487 3517.6243 3517.6195 1 0 K.IHATDQDVYDTLTYSLDPQMDNLFSVSSTGGK.L**  ([Ions score 77](http://mbp-mascot4/mascot/cgi/peptide_view.pl?file=../data/20091021/F006305.dat&query=5424&hit=1&index=IPI00031411&px=1&section=5&ave_thresh=22))

**3650 - 3664 922.4348 1842.8551 1842.8577 -1 0 R.FANLTPEEFVGDYWR.N**  ([Ions score 51](http://mbp-mascot4/mascot/cgi/peptide_view.pl?file=../data/20091021/F006305.dat&query=4280&hit=1&index=IPI00031411&px=1&section=5&ave_thresh=22))

**3650 - 3664 922.4353 1842.8560 1842.8577 -1 0 R.FANLTPEEFVGDYWR.N**  ([Ions score 42](http://mbp-mascot4/mascot/cgi/peptide_view.pl?file=../data/20091021/F006305.dat&query=4281&hit=1&index=IPI00031411&px=1&section=5&ave_thresh=22))

**3740 - 3749 610.2838 1218.5531 1218.5526 0 0 K.LCAGLDCPWK.F**  ([Ions score 38](http://mbp-mascot4/mascot/cgi/peptide_view.pl?file=../data/20091021/F006305.dat&query=2560&hit=1&index=IPI00031411&px=1&section=5&ave_thresh=22))

**3740 - 3749 610.2840 1218.5534 1218.5526 1 0 K.LCAGLDCPWK.F**  ([Ions score 30](http://mbp-mascot4/mascot/cgi/peptide_view.pl?file=../data/20091021/F006305.dat&query=2561&hit=1&index=IPI00031411&px=1&section=5&ave_thresh=22))

**3755 - 3769 803.3803 1604.7461 1604.7464 -0 0 K.VSVDESVMSTHSTAR.L**  ([Ions score 86](http://mbp-mascot4/mascot/cgi/peptide_view.pl?file=../data/20091021/F006305.dat&query=3785&hit=1&index=IPI00031411&px=1&section=5&ave_thresh=22))

**3755 - 3769 535.9237 1604.7493 1604.7464 2 0 K.VSVDESVMSTHSTAR.L**  ([Ions score 37](http://mbp-mascot4/mascot/cgi/peptide_view.pl?file=../data/20091021/F006305.dat&query=3788&hit=1&index=IPI00031411&px=1&section=5&ave_thresh=22))

**3770 - 3776 410.2401 818.4657 818.4650 1 0 R.LSFVTPR.H**  ([Ions score 27](http://mbp-mascot4/mascot/cgi/peptide_view.pl?file=../data/20091021/F006305.dat&query=356&hit=1&index=IPI00031411&px=1&section=5&ave_thresh=22))

**3826 - 3844 1010.9581 2019.9016 2019.9030 -1 0 R.FGQCPGSSSMTLTGNSYVK.Y**  ([Ions score 69](http://mbp-mascot4/mascot/cgi/peptide_view.pl?file=../data/20091021/F006305.dat&query=4531&hit=1&index=IPI00031411&px=1&section=5&ave_thresh=22))

**3864 - 3875 699.8444 1397.6742 1397.6762 -1 0 R.TYSTHAVVMYAR.G**  ([Ions score 63](http://mbp-mascot4/mascot/cgi/peptide_view.pl?file=../data/20091021/F006305.dat&query=3343&hit=1&index=IPI00031411&px=1&section=5&ave_thresh=22))

**3864 - 3875 466.9003 1397.6791 1397.6762 2 0 R.TYSTHAVVMYAR.G**  ([Ions score 36](http://mbp-mascot4/mascot/cgi/peptide_view.pl?file=../data/20091021/F006305.dat&query=3344&hit=1&index=IPI00031411&px=1&section=5&ave_thresh=22))

**3928 - 3945 904.4984 1806.9823 1806.9840 -1 0 R.LVLDQVHTASGTAPGTLK.T**  ([Ions score 43](http://mbp-mascot4/mascot/cgi/peptide_view.pl?file=../data/20091021/F006305.dat&query=4188&hit=1&index=IPI00031411&px=1&section=5&ave_thresh=22))

**3928 - 3945 603.3351 1806.9836 1806.9840 -0 0 R.LVLDQVHTASGTAPGTLK.T**  ([Ions score 41](http://mbp-mascot4/mascot/cgi/peptide_view.pl?file=../data/20091021/F006305.dat&query=4189&hit=1&index=IPI00031411&px=1&section=5&ave_thresh=22))

**3946 - 3960 589.3060 1764.8962 1764.8948 1 0 K.TLNLDNYVFFGGHIR.Q**  ([Ions score 33](http://mbp-mascot4/mascot/cgi/peptide_view.pl?file=../data/20091021/F006305.dat&query=4106&hit=1&index=IPI00031411&px=1&section=5&ave_thresh=22))

**PaCa44 A4**

Match to: **IPI00031411** Score: **79**

**Gene_Symbol=FAT1 Protocadherin Fat 1 lng=4591 # SP[4593,D,22,D]SNP[4646,g,1064,R]SNP[4677,r,1064,R]SNP[4713,i,1125,I]SNP[4791,l,1125,I]SNP[4844,i,1252,I]SNP[4856,h,1273,H]SNP[4889,r,1273,H]SNP[4931,l,1283,P]SNP[4969,p,1283,P]SNP[5000,l,129,V]SNP[5**

Found in search of C:\mgf\Orbidata\081103_ISW1295_Paca44_A4.mgf

Nominal mass (M_r_): **1068827**; Calculated pI value: **4.55**

NCBI BLAST search of [IPI00031411](http://www.ncbi.nlm.nih.gov/blast/Blast.cgi?ALIGNMENTS=50&ALIGNMENT_VIEW=Pairwise&AUTO_FORMAT=Semiauto&CDD_SEARCH=on&CLIENT=web&COMPOSITION_BASED_STATISTICS=on&DATABASE=nr&DESCRIPTIONS=100&ENTREZ_QUERY=(none)&EXPECT=10&FILTER=L&FORMAT_BLOCK_ON_RESPAGE=None&FORMAT_OBJECT=Alignment&FORMAT_TYPE=HTML&GAPCOSTS=11+1&I_THRESH=0.001&LAYOUT=TwoWindows&MATRIX_NAME=BLOSUM62&NCBI_GI=on&PAGE=Proteins&PROGRAM=blastp&QUERY=IPI00031411&SERVICE=plain&SET_DEFAULTS.x=21&SET_DEFAULTS.y=7&SHOW_OVERVIEW=on&WORD_SIZE=3&END_OF_HTTPGET=Yes) against nr

Unformatted [sequence string](http://mbp-mascot4/mascot/cgi/getseq.pl?IPI_human+IPI00031411+seq) for pasting into other applications

Fixed modifications: Carbamidomethyl (C)

Variable modifications: Oxidation (M)

Cleavage by TrypsinMSIPI, a mixture of enzymes:

cuts C-term side of KR unless next residue is P

cuts C-term side of J

cuts N-term side of J

Sequence Coverage: **0%**

Matched peptides shown in **Bold Red**

**1** MGRHLALLLL LLLLFQHFGD SDGSQRLEQT PLQFTHLEYN VTVQENSAAK

**51** TYVGHPVKMG VYITHPAWEV RYKIVSGDSE NLFKAEEYIL GDFCFLRIRT

**101** KGGNTAILNR EVKDHYTLIV KALEKNTNVE ARTKVRVQVL DTNDLRPLFS

**151** PTSYSVSLPE NTAIRTSIAR VSATDADIGT NGEFYYSFKD RTDMFAIHPT

**201** SGVIVLTGRL DYLETKLYEM EILAADRGMK LYGSSGISSM AKLTVHIEQA

**251** NECAPVITAV TLSPSELDRD PAYAIVTVDD CDQGANGDIA SLSIVAGDLL

**301** QQFRTVRSFP GSKEYKVKAI GGIDWDSHPF GYNLTLQAKD KGTPPQFSSV

**351** KVIHVTSPQF KAGPVKFEKD VYRAEISEFA PPNTPVVMVK AIPAYSHLRY

**401** VFKSTPGKAK FSLNYNTGLI SILEPVKRQQ AAHFELEVTT SDRKASTKVL

**451** VK**VLGANSNP PEFTQTAYK**A AFDENVPIGT TVMSLSAVDP DEGENGYVTY

**501** SIANLNHVPF AIDHFTGAVS TSENLDYELM PRVYTLRIRA SDWGLPYRRE

**551** VEVLATITLN NLNDNTPLFE KINCEGTIPR DLGVGEQITT VSAIDADELQ

**601** LVQYQIEAGN ELDFFSLNPN SGVLSLKRSL MDGLGAKVSF HSLRITATDG

**651** ENFATPLYIN ITVAASHK**LV NLQCEETGVA K**MLAEKLLQA NKLHNQGEVE

**701** DIFFDSHSVN AHIPQFRSTL PTGIQVKENQ PVGSSVIFMN STDLDTGFNG

**751** KLVYAVSGGN EDSCFMIDME TGMLKILSPL DRETTDKYTL NITVYDLGIP

**801** QKAAWRLLHV VVVDANDNPP EFLQESYFVE VSEDKEVHSE IIQVEATDKD

**851** LGPNGHVTYS IVTDTDTFSI DSVTGVVNIA RPLDRELQHE HSLKIEARDQ

**901** AREEPQLFST VVVKVSLEDV NDNPPTFIPP NYRVKVREDL PEGTVIMWLE

**951** AHDPDLGQSG QVRYSLLDHG EGNFDVDKLS GAVRIVQQLD FEKKQVYNLT

**1001** VRAKDKGKPV SLSSTCYVEV EVVDVNENLH PPVFSSFVEK GTVKEDAPVG

**1051** SLVMTVSAHD EDARRDGEIR YSIRDGSGVG VFKIGEETGV IETSDRLDRE

**1101** STSHYWLTVF ATDQGVVPLS SFIEIYIEVE DVNDNAPQTS EPVYYPEIME

**1151** NSPKDVSVVQ IEAFDPDSSS NDKLMYKITS GNPQGFFSIH PKTGLITTTS

**1201** RKLDREQQDE HILEVTVTDN GSPPKSTIAR VIVK**ILDEND NKPQFLQK**FY

**1251** KIRLPEREKP DRERNARREP LYHVIATDKD EGPNAEISYS IEDGNEHGKF

**1301** FIEPKTGVVS SKRFSAAGEY DILSIKAVDN GRPQKSSTTR LHIEWISKPK

**1351** PSLEPISFEE SFFTFTVMES DPVAHMIGVI SVEPPGIPLW FDITGGNYDS

**1401** HFDVDKGTGT IIVAKPLDAE QKSNYNLTVE ATDGTTTILT QVFIKVIDTN

**1451** DHRPQFSTSK YEVVIPEDTA PETEILQISA VDQDEKNKLI YTLQSSRDPL

**1501** SLKKFRLDPA TGSLYTSEKL DHEAVHQHTL TVMVRDQDVP VKRNFARIVV

**1551** NVSDTNDHAP WFTASSYKGR VYESAAVGSV VLQVTALDKD KGKNAEVLYS

**1601** IESGTFGNIG NSFMIDPVLG SIKTAKELDR SNQAEYDLMV KATDKGSPPM

**1651** SEITSVRIFV TIADNASPKF TSKEYSVELS ETVSIGSFVG MVTAHSQSSV

**1701** VYEIKDGNTG DAFDINPHSG TIITQKALDF ETLPIYTLII QGTNMAGLST

**1751** NTTVLVHLQD ENDNAPVFMQ AEYTGLISES ASINSVVLTD RNVPLVIRAA

**1801** DADKDSNALL VYHIVEPSVH TYFAIDSSTG AIHTVLSLDY EETSIFHFTV

**1851** QVHDMGTPRL FAEYAANVTV HVIDINDCPP VFAKPLYEAS LLLPTYKGVK

**1901** VITVNATDAD SSAFSQLIYS ITEGNIGEKF SMDYKTGALT VQNTTQLRSR

**1951** YELTVRASDG RFAGLTSVKI NVKESKESHL K**FTQDVYSAV VK**ENSTEAET

**2001** LAVITAIGNP INEPLFYHIL NPDRRFKISR **TSGVLSTTGT PFDR**EQQEAF

**2051** DVVVEVTEEH KPSAVAHVVV KVIVEDQNDN APVFVNLPYY AVVKVDTEVG

**2101** HVIRYVTAVD RDSGRNGEVH YYLKEHHEHF QIGPLGEISL KKQFELDTLN

**2151** KEYLVTVVAK DGGNPAFSAE VIVPITVMNK AMPVFEKPFY SAEIAESIQV

**2201** HSPVVHVQAN SPEGLKVFYS ITDGDPFSQF TINFNTGVIN VIAPLDFEAH

**2251** PAYKLSIRAT DSLTGAHAEV FVDIIVDDIN DNPPVFAQQS YAVTLSEASV

**2301** IGTSVVQVRA TDSDSEPNRG ISYQMFGNHS KSHDHFHVDS STGLISLLRT

**2351** LDYEQSRQHT IFVRAVDGGM PTLSSDVIVT VDVTDLNDNP PLFEQQIYEA

**2401** RISEHAPHGH FVTCVKAYDA DSSDIDKLQY SILSGNDHKH FVIDSATGII

**2451** TLSNLHRHAL KPFYSLNLSV SDGVFRSSTQ VHVTVIGGNL HSPAFLQNEY

**2501** EVELAENAPL HTLVMEVKTT DGDSGIYGHV TYHIVNDFAK DRFYINERGQ

**2551** IFTLEKLDRE TPAEKVISVR LMAKDAGGKV AFCTVNVILT DDNDNAPQFR

**2601** ATKYEVNIGS SAAKGTSVVK VLASDADEGS NADITYAIEA DSESVKENLE

**2651** INKLSGVITT KESLIGLENE FFTFFVRAVD NGSPSKESVV LVYVKILPPE

**2701** MQLPKFSEPF YTFTVSEDVP IGTEIDLIRA EHSGTVLYSL VKGNTPESNR

**2751** DESFVIDRQS GRLKLEKSLD HETTKWYQFS ILARCTQDDH EMVASVDVSI

**2801** QVKDANDNSP VFESSPYEAF IVENLPGGSR VIQIRASDAD SGTNGQVMYS

**2851** LDQSQSVEVI ESFAINMETG WITTLKELDH EKRDNYQIKV VASDHGEKIQ

**2901** LSSTAIVDVT VTDVNDSPPR FTAEIYKGTV SEDDPQGGVI AILSTTDADS

**2951** EEINRQVTYF ITGGDPLGQF AVETIQNEWK VYVKKPLDRE KRDNYLLTIT

**3001** ATDGTFSSKA IVEVKVLDAN DNSPVCEKTL YSDTIPEDVL PGKLIMQISA

**3051** TDADIRSNAE ITYTLLGSGA EKFKLNPDTG ELKTSTPLDR EEQAVYHLLV

**3101** RATDGGGRFC QASIVLTLED VNDNAPEFSA DPYAITVFEN TEPGTLLTRV

**3151** QATDADAGLN RKILYSLIDS ADGQFSINEL SGIIQLEKPL DRELQAVYTL

**3201** SLKAVDQGLP RRLTATGTVI VSVLDINDNP PVFEYREYGA TVSEDILVGT

**3251** EVLQVYAASR DIEANAEITY SIISGNEHGK FSIDSKTGAV FIIENLDYES

**3301** SHEYYLTVEA TDGGTPSLSD VATVNVNVTD INDNTPVFSQ DTYTTVISED

**3351** AVLEQSVITV MADDADGPSN SHIHYSIIDG NQGSSFTIDP VRGEVKVTKL

**3401** LDRETISGYT LTVQASDNGS PPRVNTTTVN IDVSDVNDNA PVFSRGNYSV

**3451** IIQENKPVGF SVLQLVVTDE DSSHNGPPFF FTIVTGNDEK AFEVNPQGVL

**3501** LTSSAIKRKE KDHYLLQVKV ADNGKPQLSS LTYIDIRVIE ESIYPPAILP

**3551** LEIFITSSGE EYSGGVIGKI HATDQDVYDT LTYSLDPQMD NLFSVSSTGG

**3601** KLIAHKKLDI GQYLLNVSVT DGKFTTVADI TVHIRQVTQE MLNHTIAIRF

**3651** ANLTPEEFVG DYWRNFQRAL RNILGVRRND IQIVSLQSSE PHPHLDVLLF

**3701** VEKPGSAQIS TKQLLHKINS SVTDIEEIIG VRILNVFQKL CAGLDCPWKF

**3751** CDEKVSVDES VMSTHSTARL SFVTPRHHRA AVCLCKEGRC PPVHHGCEDD

**3801** PCPEGSECVS DPWEEKHTCV CPSGRFGQCP GSSSMTLTGN SYVKYRLTEN

**3851** ENKLEMKLTM RLRTYSTHAV VMYARGTDYS ILEIHHGRLQ YKFDCGSGPG

**3901** IVSVQSIQVN DGQWHAVALE VNGNYARLVL DQVHTASGTA PGTLKTLNLD

**3951** NYVFFGGHIR QQGTRHGRSP QVGNGFRGCM DSIYLNGQEL PLNSKPRSYA

**4001** HIEESVDVSP GCFLTATEDC ASNPCQNGGV CNPSPAGGYY CKCSALYIGT

**4051** HCEISVNPCS SKPCLYGGTC VVDNGGFVCQ CRGLYTGQRC QLSPYCKDEP

**4101** CKNGGTCFDS LDGAVCQCDS GFRGERCQSD IDECSGNPCL HGALCENTHG

**4151** SYHCNCSHEY RGRHCEDAAP NQYVSTPWNI GLAEGIGIVV FVAGIFLLVV

**4201** VFVLCRKMIS RKKKHQAEPK DKHLGPATAF LQRPYFDSKL NKNIYSDIPP

**4251** QVPVRPISYT PSIPSDSRNN LDRNSFEGSA IPEHPEFSTF NPESVHGHRK

**4301** AVAVCSVAPN LPPPPPSNSP SDSDSIQKPS WDFDYDTKVV DLDPCLSKKP

**4351** LEEKPSQPYS ARESLSEVQS LSSFQSESCD DNGYHWDTSD WMPSVPLPDI

**4401** QEFPNYEVID EQTPLYSADP NAIDTDYYPG GYDIESDFPP PPEDFPAADE

**4451** LPPLPPEFSN QFESIHPPRD MPAAGSLGSS SRNRQRFNLN QYLPNFYPLD

**4501** MSEPQTKGTG ENSTCREPHA PYPPGYQRHF EAPAVESMPM SVYASTASCS

**4551** DVSACCEVES EVMMSDYESG DDGHFEEVTI PPLDSQQHTE VJDGSQRLEQ

**4601** TPLQFTHLEY NVTVQENSAA KJGTVKEDAP VGSLVMTVSA HDEDAGRDGE

**4651** IRJGTVKEDA PVGSLVMTVS AHDEDARRDG EIRJLDREST SHYWLTVFAT

**4701** DQGVVPLSSF IEIYIEVEDV NDNAPQTSEP VYYPEIMENS PKDVSVVQIE

**4751** AFDPDSSSND KJLDRESTSH YWLTVFATDQ GVVPLSSFIE LYIEVEDVND

**4801** NAPQTSEPVY YPEIMENSPK DVSVVQIEAF DPDSSSNDKJ FYKIRLPERJ

**4851** REPLYHVIAT DKDEGPNAEI SYSIEDGNEH GKJREPLYRV IATDKDEGPN

**4901** AEISYSIEDG NEHGKJEPLY HVIATDKDEG LNAEISYSIE DGNEHGKFFI

**4951** EPKJEPLYHV IATDKDEGPN AEISYSIEDG NEHGKFFIEP KJALEKNTNL

**5001** EARTKJALEK NTNVEARTKJ ALEKNTNVEA RTKJALEKNT NVEVRTKJFS

**5051** AAGEYDILSI KAVDNGRPQK SSTTRJFSAA GEYDILSIKA VDSGRPQKSS

**5101** TTRJNFARIV VNVSDTNDHA PWFTASSYKG RJNFARIVVN VSDTNDHAPW

**5151** FTTSSYKGRJ GKNAEVLYSI ESGDFGNIGN SFMIDPVLGS IKTAKJGKNA

**5201** EVLYSIESGN FGNIGNSFMI DPVLGSIKTA KJGKNAEVLY SIESGTIGNI

**5251** GNSFMIDPVL GSIKTAKJGS PPMSEITSVR IFVTNADNAS PKFTSKJGSP

**5301** PMSEITSVRI FVTSADNASP KFTSKJGSPP MSEITSVRIF VTIADNASTK

**5351** FTSKJALDFE TLPIYTLIIQ GTNMAGLSTN TTVLVHLQDE NDNAPVFMQA

**5401** EYTGLISESA SINSVVLTDR NVPQVIRAAD ADKJALDFET LPIYTLIIQG

**5451** TNMAGLSTNT TVLVHLQDEN DNAPVFMQAE YTGLISESAS INSVVLTDRN

**5501** VPRVIRAADA DKJAADADKD SNVLLVYHIV EPSVHTYFAI DSSTGAIHTV

**5551** LSLDYEETSI FHFTVQVHDM GTPRLFAEYA ANVTVHVIDI NDCPPVFAKP

**5601** LYEASLLLPT YKJFTQDVYS AVVKTNSTEA ETLAVITAIG NPINEPLFYH

**5651** ILNPDRRJTS GVLSTTGTPF DREAQEAFDV VVEVTEEHKP SAVAHVVVKV

**5701** IVEDQNDNAP VFVNLPYYAV VKJTDMFAIH PTSGVIVLTG RLDFLETKLY

**5751** EMEILAADRJ TDMFAIHPTS GVIVLTGRLD YLETKLYEME ILAADRJNGE

**5801** VHYYLKEHHE HFQIGPLGEK SLKKJDGGNP AFSAEVIVPI TVMNKAMPVF

**5851** EKPFYSAEIA ESIQVHSHVV HVQANSPEGL KVFYSITDGD PFSQFTINFN

**5901** TGVINVIAPL DFEAHPAYKJ AMPVFEKPFY SAEIAESIQV HSPVVHVQAN

**5951** SPEGLKVFYS ITDGDPFSQF TINFNTGVIN VIAPLDFPAH PAYKLSIRJL

**6001** SIRATDSLTG AHAEVFVDDI VDDINDNPPV FAQQSYAVTL SEASVIGTSV

**6051** VQVRATDSDS EPNRJLSIRA TDSLTGAHAE VFVDEIVDDI NDNPPVFAQQ

**6101** SYAVTLSEAS VIGTSVVQVR ATDSDSEPNR JTLDYEQSRQ HTIAVRAVDG

**6151** GMPTLSSDVI VTVDVTDLND NPPLFEQQIY EARJLYGSSG ISSMAKLTVH

**6201** IEQANECAPV ITAVTLSPSE LDRDPAYAIV TVDDCDQGAN GDIASLSIVA

**6251** GDLLQQFRJI LPPEMQLPKF SEPFYTFTVS EIVPIGTEID LIRAEHSGTV

**6301** LYSLVKJILP PEMQLPKFSE PFYTFTVSEV VPIGTEIDLI RAEHSGTVLY

**6351** SLVKJCTQDD HEMVASVDVS IQVKDANDNS PVFESSPYEA FIVENLPGGS

**6401** RVIQIRJCTQ DDHEMVASVD VSIQVKDASD NSPVFESSPY EAFIVENLPG

**6451** GSRVIQIRJV IQIRASDADN GTNGQVMYSL DQSQSVEVIE SFAINMETGW

**6501** ITTLKELDHE KJFTAEIYKG TVSELDPQGG VIAILSTTDA DSEEINRQVT

**6551** YFITGGDPLG QFAVETIQNE WKJFTAEIYK GTVSEPDPQG GVIAILSTTD

**6601** ADSEEINRQV TYFITGGDPL GQFAVETIQN EWKJFTAEIY KGTVSEQDPQ

**6651** GGVIAILSTT DADSEEINRQ VTYFITGGDP LGQFAVETIQ NEWKJGTVSE

**6701** DDPQGGVIAI LSTTDADSEE INRQVTYGIT GGDPLGQFAV ETIQNEWKVY

**6751** VKJGTVSEDD PQGGVIAILS TTDADSEEIN RQVTYSITGG DPLGQFAVET

**6801** IQNEWKVYVK JGTVSEDDPQ GGVIAILSTT DADSEEINRQ VTYFITGGDP

**6851** LAQFAVETIQ NEWKVYVKJA IVEVKVLDAN DNSPVCEYTL YSDTIPEDVL

**6901** PGKLIMQISA TDADIRJVLD ANDNSPVCEK TLYIDTIPED VLPGKLIMQI

**6951** SATDADIRJV LDANDNSPVC EKTLYTDTIP EDVLPGKLIM QISATDADIR

**7001** JATDGGGRFC QASIVLDLED VNDNAPEFSA DPYAITVFEN TEPGTLLTRV

**7051** QATDADAGLN RJKILYSLID SADGQFSINE LIGIIQLEKP LDRELQAVYT

**7101** LSLKJFSIDS KTGAVFIIEN LDYESSHEYY LTVEATDGGT PSLSDVATVN

**7151** VNVTDINDAT PVFSQDTYTT VISEDAVLEQ SVITVMADDA DGPSNSHIHY

**7201** SIIDGNQGSS FTIDPVRGEV KJFSIDSKTG AVFIIENLDY ESSHEYYLTV

**7251** EATDGGTPSL SDVATVNVNV TDINDVTPVF SQDTYTTVIS EDAVLEQSVI

**7301** TVMADDADGP SNSHIHYSII DGNQGSSFTI DPVRGEVKJV NTTTVNIDVS

**7351** DVNDNAPVFS RGNYSVIIQE NKPVGFSVLQ LVVTDEDSSH NGPPFFFTIV

**7401** TENDEKAFEV NPQGVLLTSS AIKJVNTTTV NIDVSDVNDN APVFSRGNYS

**7451** VIIQENKPVG FSVLQLVVTD EDSSHNGPPF FFTIVTGNDE KAFEVNPQGV

**7501** LLTSSAIKJG NYSVIIQENK PVGFSVLQLV VTDEDSSHNG PPFFFTIVTG

**7551** NDEKAFEVNA QGVLLTSSAI KRJGNYSVII QENKPVGFSV LQLVVTDEDS

**7601** SHNGPPFFFT IVTGNDEKAF EVNVQGVLLT SSAIKRJVAD NGKPQLSSLT

**7651** YIDIRVIEES IYPPAILPLE IAITSSGEEY SGGVIGKIHA TDQDVYDTLT

**7701** YSLDPQMDNL FSVSSTGGKJ VADNGKPQLS SLTYIDIRVI EESIYPPAIL

**7751** PLEISITSSG EEYSGGVIGK IHATDQDVYD TLTYSLDPQM DNLFSVSSTG

**7801** GKJVIHVTSP QFKAGPVKFE KJQLLHKIMS SVTDIEEIIG VRILNVFQKJ

**7851** QLLHKIVSSV TDIEEIIGVR ILNVFQKJEG RCPPVHHGCE DHPCPEGSEC

**7901** VSDPWEEKHT CVCPSGRJEG RCPPVHHGCE DPPCPEGSEC VSDPWEEKHT

**7951** CVCPSGRJHT CVCPSGRFGG CPGSSSMTLT GNSYVKYRJH TCVCPSGRFG

**8001** RCPGSSSMTL TGNSYVKYRJ FGQCPGSSSM TLTGNSYVKT RLTENENKJG

**8051** CMDSIYLNGQ ELPLNSKPRS YAHIEESVPV SPGCFLTATE DCASNPCQNG

**8101** GVCNPSPAGG YYCKCSALYI GTHCEISVNP CSSKPCLYGG TCVVDNGGFV

**8151** CQCRJYVFKR TPGKAKJYVF KSTPGKAKJS YAHIEESVDV SPGCFLTATE

**8201** DCASNPCQNG GVCNPSPAGG YYCKCSALYI GTHCEISVNP KSSKPCLYGG

**8251** TCVVDNGGFV CQCRGLYTGQ RJSYAHIEES VDVSPGCFLT ATEDCASNPC

**8301** QNGGVCNPSP AGGYYCKCSA LYIGTHCEIS VNPNSSKPCL YGGTCVVDNG

**8351** GFVCQCRGLY TGQRJDEPCK NGGTCFDSAD GAVCQCDSGF RGERJGRHCA

**8401** DAAPNQYVST PWNIGLAEGI GIVVFVAGIF LLVVVFVLCR KJGRHCEDAA

**8451** PNQYVSTPWN IGLAEGIGIV VFVAGIFLLV VVFVLCRKJN IYSDIPPQVP

**8501** VRYISYTPSI PSDSRNNLDR JKAVAVCSVA PNLPPPPPSN SPSDSDSIQK

**8551** PSWDFDYDTK VVDLDPCLSK JKPLEEKPSQ PYSARESLSS VQSLSSFQSE

**8601** SCDDNGYHWD TSDWMPSVPL PDIQEFPNYE VIDEQTPLYS ADPNAIDTDY

**8651** YPGGYDIESD FPPPPEDFPA ADELPPLPPE FSNQFESIHP PRDMPAAGSL

**8701** GSSSRJKPLE EKPSQPYSAR ESLSEVQSLS SFQSESCDDN GYHWDTSDWM

**8751** PSVPLQDIQE FPNYEVIDEQ TPLYSADPNA IDTDYYPGGY DIESDFPPPP

**8801** EDFPAADELP PLPPEFSNQF ESIHPPRDMP AAGSLGSSSR JKPLEEKPSQ

**8851** PYSARESLSE VQSLSSFQSE SCDDNGYHWD TSDWMPSVPL PDIQEFPNYE

**8901** VIDPQTPLYS ADPNAIDTDY YPGGYDIESD FPPPPEDFPA ADELPPLPPE

**8951** FSNQFESIHP PRDMPAAGSL GSSSRJESLS EVQSLSSFQS ESCDDNGYHW

**9001** DTSDWMPSVP LPDIQEFPNY EVIDEQTPLY SADPNAIDTD YYPGGYDIES

**9051** DFPPPPEDFP AADELPPLPP EFSNQFESIH PPRDMPAAGS LGSSRRNRJE

**9101** SLSEVQSLSS FQSESCDDNG YHWDTSDWMP SVPLPDIQEF PNYEVIDEQT

**9151** PLYSADPNAI DTDYYPGGYD IESDFPPPPE DFPAADELPP LPPEFSNQFE

**9201** SIHPPRDMPA AGSLGSSWRN RJVLGANSNP PEFTQTAYKA AFDENVPIGT

**9251** TIMSLSAVDP DEGENGYVTY SIANLNHVPF AIDHFTGAVS TSENLDYELM

**9301** PRVYTLRJVL GANSNPPEFT QTAYKAAFDE NVPIGTTVMS LSAVDPDEGE

**9351** NGYVTYSIAN LNHVPFAIDH FTGAVSTSEN LDYELMPRVY TLRJVLGANS

**9401** NPPEFTQTAY KAAFDENVPI GTTVMSLSAV DPDEGENGYV TYSIANLNHV

**9451** PFAIDHFTGA VSTSENLDYE LMPRVYTLRJ INCEGTIPRD LGVGEQITTV

**9501** SAIDADELQL VQYQIEAGNE LDFFSLNPNS GVLSLKRJIN CEGTIPRDLG

**9551** VGEQITTVSA IDADELQLVQ YQIEAGNELD LFSLNPNSGV LSLKRJINCE

**9601** GTIPRDLGVG EQITTVSAID ADELQLVQYQ IEAGNELDFF SLNPNSGVLS

**9651** LKRJEVHSEI IQVEATDKDL GPNGHVTYSI LTDTDTFSID SVTGVVNIAR

**9701** PLDRELQHEH SLKJEVHSEI IQVEATDKDL GPNGHVTYSI VTDTDTFSID

**9751** SVTGVVNIAR PLDRELQHEH SLK

**Start - End Observed Mr(expt) Mr(calc) ppm Miss Sequence**

**453 - 469 918.9597 1835.9047 1835.9054 -0 0 K.VLGANSNPPEFTQTAYK.A**  ([Ions score 24](http://mbp-mascot4/mascot/cgi/peptide_view.pl?file=../data/20091021/F006306.dat&query=4213&hit=1&index=IPI00031411&px=1&section=5&ave_thresh=22))

**669 - 681 730.8729 1459.7312 1459.7341 -2 0 K.LVNLQCEETGVAK.M**  ([Ions score 40](http://mbp-mascot4/mascot/cgi/peptide_view.pl?file=../data/20091021/F006306.dat&query=3386&hit=1&index=IPI00031411&px=1&section=5&ave_thresh=22))

**1235 - 1248 567.9658 1700.8756 1700.8733 1 0 K.ILDENDNKPQFLQK.F**  ([Ions score 24](http://mbp-mascot4/mascot/cgi/peptide_view.pl?file=../data/20091021/F006306.dat&query=3933&hit=1&index=IPI00031411&px=1&section=5&ave_thresh=22))

**1982 - 1992 628.8293 1255.6440 1255.6449 -1 0 K.FTQDVYSAVVK.E**  ([Ions score 35](http://mbp-mascot4/mascot/cgi/peptide_view.pl?file=../data/20091021/F006306.dat&query=2645&hit=1&index=IPI00031411&px=1&section=5&ave_thresh=22))

**2031 - 2044 719.8616 1437.7087 1437.7100 -1 0 R.TSGVLSTTGTPFDR.E**  ([Ions score 34](http://mbp-mascot4/mascot/cgi/peptide_view.pl?file=../data/20091021/F006306.dat&query=3330&hit=1&index=IPI00031411&px=1&section=5&ave_thresh=22))

**A818 A2**

Match to: **IPI00031411** Score: **70**

**Gene_Symbol=FAT1 Protocadherin Fat 1 lng=4591 # SP[4593,D,22,D]SNP[4646,g,1064,R]SNP[4677,r,1064,R]SNP[4713,i,1125,I]SNP[4791,l,1125,I]SNP[4844,i,1252,I]SNP[4856,h,1273,H]SNP[4889,r,1273,H]SNP[4931,l,1283,P]SNP[4969,p,1283,P]SNP[5000,l,129,V]SNP[5**

Found in search of C:\mgf\Orbidata\081031_ISW1295_A818_A2.mgf

Nominal mass (M_r_): **1068827**; Calculated pI value: **4.55**

NCBI BLAST search of [IPI00031411](http://www.ncbi.nlm.nih.gov/blast/Blast.cgi?ALIGNMENTS=50&ALIGNMENT_VIEW=Pairwise&AUTO_FORMAT=Semiauto&CDD_SEARCH=on&CLIENT=web&COMPOSITION_BASED_STATISTICS=on&DATABASE=nr&DESCRIPTIONS=100&ENTREZ_QUERY=(none)&EXPECT=10&FILTER=L&FORMAT_BLOCK_ON_RESPAGE=None&FORMAT_OBJECT=Alignment&FORMAT_TYPE=HTML&GAPCOSTS=11+1&I_THRESH=0.001&LAYOUT=TwoWindows&MATRIX_NAME=BLOSUM62&NCBI_GI=on&PAGE=Proteins&PROGRAM=blastp&QUERY=IPI00031411&SERVICE=plain&SET_DEFAULTS.x=21&SET_DEFAULTS.y=7&SHOW_OVERVIEW=on&WORD_SIZE=3&END_OF_HTTPGET=Yes) against nr

Unformatted [sequence string](http://mbp-mascot4/mascot/cgi/getseq.pl?IPI_human+IPI00031411+seq) for pasting into other applications

Fixed modifications: Carbamidomethyl (C)

Variable modifications: Oxidation (M)

Cleavage by TrypsinMSIPI, a mixture of enzymes:

cuts C-term side of KR unless next residue is P

cuts C-term side of J

cuts N-term side of J

Sequence Coverage: **0%**

Matched peptides shown in **Bold Red**

**1** MGRHLALLLL LLLLFQHFGD SDGSQRLEQT PLQFTHLEYN VTVQENSAAK

**51** TYVGHPVKMG VYITHPAWEV RYKIVSGDSE NLFKAEEYIL GDFCFLRIRT

**101** KGGNTAILNR EVKDHYTLIV KALEKNTNVE ARTKVRVQVL DTNDLRPLFS

**151** PTSYSVSLPE NTAIRTSIAR VSATDADIGT NGEFYYSFKD RTDMFAIHPT

**201** SGVIVLTGRL DYLETKLYEM EILAADRGMK LYGSSGISSM AKLTVHIEQA

**251** NECAPVITAV TLSPSELDRD PAYAIVTVDD CDQGANGDIA SLSIVAGDLL

**301** QQFRTVRSFP GSKEYKVKAI GGIDWDSHPF GYNLTLQAKD KGTPPQFSSV

**351** KVIHVTSPQF KAGPVKFEKD VYRAEISEFA PPNTPVVMVK AIPAYSHLRY

**401** VFKSTPGKAK FSLNYNTGLI SILEPVKRQQ AAHFELEVTT SDRKASTKVL

**451** VKVLGANSNP PEFTQTAYKA AFDENVPIGT TVMSLSAVDP DEGENGYVTY

**501** SIANLNHVPF AIDHFTGAVS TSENLDYELM PRVYTLRIRA SDWGLPYRRE

**551** VEVLATITLN NLNDNTPLFE KINCEGTIPR DLGVGEQITT VSAIDADELQ

**601** LVQYQIEAGN ELDFFSLNPN SGVLSLKRSL MDGLGAKVSF HSLRITATDG

**651** ENFATPLYIN ITVAASHKLV NLQCEETGVA KMLAEKLLQA NKLHNQGEVE

**701** DIFFDSHSVN AHIPQFRSTL PTGIQVKENQ PVGSSVIFMN STDLDTGFNG

**751** KLVYAVSGGN EDSCFMIDME TGMLKILSPL DRETTDKYTL NITVYDLGIP

**801** QKAAWRLLHV VVVDANDNPP EFLQESYFVE VSEDKEVHSE IIQVEATDKD

**851** LGPNGHVTYS IVTDTDTFSI DSVTGVVNIA RPLDRELQHE HSLKIEARDQ

**901** AREEPQLFST VVVKVSLEDV NDNPPTFIPP NYRVKVREDL PEGTVIMWLE

**951** AHDPDLGQSG QVRYSLLDHG EGNFDVDKLS GAVRIVQQLD FEKKQVYNLT

**1001** VRAKDKGKPV SLSSTCYVEV EVVDVNENLH PPVFSSFVEK GTVKEDAPVG

**1051** SLVMTVSAHD EDARRDGEIR YSIRDGSGVG VFKIGEETGV IETSDRLDRE

**1101** STSHYWLTVF ATDQGVVPLS SFIEIYIEVE DVNDNAPQTS EPVYYPEIME

**1151** NSPKDVSVVQ IEAFDPDSSS NDKLMYKITS GNPQGFFSIH PKTGLITTTS

**1201** RKLDREQQDE HILEVTVTDN GSPPKSTIAR VIVKILDEND NKPQFLQKFY

**1251** KIRLPEREKP DRERNARREP LYHVIATDKD EGPNAEISYS IEDGNEHGKF

**1301** FIEPKTGVVS SKRFSAAGEY DILSIKAVDN GRPQKSSTTR LHIEWISKPK

**1351** PSLEPISFEE SFFTFTVMES DPVAHMIGVI SVEPPGIPLW FDITGGNYDS

**1401** HFDVDKGTGT IIVAKPLDAE QKSNYNLTVE ATDGTTTILT QVFIKVIDTN

**1451** DHRPQFSTSK YEVVIPEDTA PETEILQISA VDQDEKNKLI YTLQSSRDPL

**1501** SLKKFRLDPA TGSLYTSEKL DHEAVHQHTL TVMVRDQDVP VKRNFARIVV

**1551** NVSDTNDHAP WFTASSYKGR VYESAAVGSV VLQVTALDKD KGKNAEVLYS

**1601** IESGTFGNIG NSFMIDPVLG SIKTAKELDR SNQAEYDLMV KATDKGSPPM

**1651** SEITSVR**IFV TIADNASPK**F TSKEYSVELS ETVSIGSFVG MVTAHSQSSV

**1701** VYEIKDGNTG DAFDINPHSG TIITQKALDF ETLPIYTLII QGTNMAGLST

**1751** NTTVLVHLQD ENDNAPVFMQ AEYTGLISES ASINSVVLTD RNVPLVIRAA

**1801** DADKDSNALL VYHIVEPSVH TYFAIDSSTG AIHTVLSLDY EETSIFHFTV

**1851** QVHDMGTPRL FAEYAANVTV HVIDINDCPP VFAKPLYEAS LLLPTYKGVK

**1901** VITVNATDAD SSAFSQLIYS ITEGNIGEKF SMDYKTGALT VQNTTQLRSR

**1951** YELTVRASDG RFAGLTSVKI NVKESKESHL K**FTQDVYSAV VK**ENSTEAET

**2001** LAVITAIGNP INEPLFYHIL NPDRRFKISR **TSGVLSTTGT PFDR**EQQEAF

**2051** DVVVEVTEEH KPSAVAHVVV KVIVEDQNDN APVFVNLPYY AVVKVDTEVG

**2101** HVIRYVTAVD RDSGRNGEVH YYLKEHHEHF QIGPLGEISL KKQFELDTLN

**2151** KEYLVTVVAK DGGNPAFSAE VIVPITVMNK AMPVFEKPFY SAEIAESIQV

**2201** HSPVVHVQAN SPEGLKVFYS ITDGDPFSQF TINFNTGVIN VIAPLDFEAH

**2251** PAYKLSIRAT DSLTGAHAEV FVDIIVDDIN DNPPVFAQQS YAVTLSEASV

**2301** IGTSVVQVRA TDSDSEPNRG ISYQMFGNHS KSHDHFHVDS STGLISLLRT

**2351** LDYEQSRQHT IFVRAVDGGM PTLSSDVIVT VDVTDLNDNP PLFEQQIYEA

**2401** RISEHAPHGH FVTCVKAYDA DSSDIDKLQY SILSGNDHKH FVIDSATGII

**2451** TLSNLHRHAL KPFYSLNLSV SDGVFRSSTQ VHVTVIGGNL HSPAFLQNEY

**2501** EVELAENAPL HTLVMEVKTT DGDSGIYGHV TYHIVNDFAK DRFYINERGQ

**2551** IFTLEKLDRE TPAEKVISVR LMAKDAGGKV AFCTVNVILT DDNDNAPQFR

**2601** ATKYEVNIGS SAAKGTSVVK VLASDADEGS NADITYAIEA DSESVKENLE

**2651** INKLSGVITT KESLIGLENE FFTFFVRAVD NGSPSKESVV LVYVKILPPE

**2701** MQLPKFSEPF YTFTVSEDVP IGTEIDLIRA EHSGTVLYSL VKGNTPESNR

**2751** DESFVIDRQS GRLKLEKSLD HETTKWYQFS ILARCTQDDH EMVASVDVSI

**2801** QVKDANDNSP VFESSPYEAF IVENLPGGSR VIQIRASDAD SGTNGQVMYS

**2851** LDQSQSVEVI ESFAINMETG WITTLKELDH EKRDNYQIKV VASDHGEKIQ

**2901** LSSTAIVDVT VTDVNDSPPR FTAEIYKGTV SEDDPQGGVI AILSTTDADS

**2951** EEINRQVTYF ITGGDPLGQF AVETIQNEWK VYVKKPLDRE KRDNYLLTIT

**3001** ATDGTFSSKA IVEVKVLDAN DNSPVCEKTL YSDTIPEDVL PGKLIMQISA

**3051** TDADIRSNAE ITYTLLGSGA EKFKLNPDTG ELKTSTPLDR EEQAVYHLLV

**3101** RATDGGGRFC QASIVLTLED VNDNAPEFSA DPYAITVFEN TEPGTLLTRV

**3151** QATDADAGLN RKILYSLIDS ADGQFSINEL SGIIQLEKPL DRELQAVYTL

**3201** SLKAVDQGLP RRLTATGTVI VSVLDINDNP PVFEYREYGA TVSEDILVGT

**3251** EVLQVYAASR DIEANAEITY SIISGNEHGK FSIDSKTGAV FIIENLDYES

**3301** SHEYYLTVEA TDGGTPSLSD VATVNVNVTD INDNTPVFSQ DTYTTVISED

**3351** AVLEQSVITV MADDADGPSN SHIHYSIIDG NQGSSFTIDP VRGEVKVTKL

**3401** LDRETISGYT LTVQASDNGS PPRVNTTTVN IDVSDVNDNA PVFSRGNYSV

**3451** IIQENKPVGF SVLQLVVTDE DSSHNGPPFF FTIVTGNDEK AFEVNPQGVL

**3501** LTSSAIKRKE KDHYLLQVKV ADNGKPQLSS LTYIDIRVIE ESIYPPAILP

**3551** LEIFITSSGE EYSGGVIGKI HATDQDVYDT LTYSLDPQMD NLFSVSSTGG

**3601** KLIAHKKLDI GQYLLNVSVT DGKFTTVADI TVHIRQVTQE MLNHTIAIRF

**3651** ANLTPEEFVG DYWRNFQRAL RNILGVRRND IQIVSLQSSE PHPHLDVLLF

**3701** VEKPGSAQIS TKQLLHKINS SVTDIEEIIG VRILNVFQKL CAGLDCPWKF

**3751** CDEKVSVDES VMSTHSTARL SFVTPRHHRA AVCLCKEGRC PPVHHGCEDD

**3801** PCPEGSECVS DPWEEKHTCV CPSGRFGQCP GSSSMTLTGN SYVKYRLTEN

**3851** ENKLEMKLTM RLRTYSTHAV VMYARGTDYS ILEIHHGRLQ YKFDCGSGPG

**3901** IVSVQSIQVN DGQWHAVALE VNGNYARLVL DQVHTASGTA PGTLKTLNLD

**3951** NYVFFGGHIR QQGTRHGRSP QVGNGFRGCM DSIYLNGQEL PLNSKPRSYA

**4001** HIEESVDVSP GCFLTATEDC ASNPCQNGGV CNPSPAGGYY CKCSALYIGT

**4051** HCEISVNPCS SKPCLYGGTC VVDNGGFVCQ CRGLYTGQRC QLSPYCKDEP

**4101** CKNGGTCFDS LDGAVCQCDS GFRGERCQSD IDECSGNPCL HGALCENTHG

**4151** SYHCNCSHEY RGRHCEDAAP NQYVSTPWNI GLAEGIGIVV FVAGIFLLVV

**4201** VFVLCRKMIS RKKKHQAEPK DKHLGPATAF LQRPYFDSKL NKNIYSDIPP

**4251** QVPVRPISYT PSIPSDSRNN LDRNSFEGSA IPEHPEFSTF NPESVHGHRK

**4301** AVAVCSVAPN LPPPPPSNSP SDSDSIQKPS WDFDYDTKVV DLDPCLSKKP

**4351** LEEKPSQPYS ARESLSEVQS LSSFQSESCD DNGYHWDTSD WMPSVPLPDI

**4401** QEFPNYEVID EQTPLYSADP NAIDTDYYPG GYDIESDFPP PPEDFPAADE

**4451** LPPLPPEFSN QFESIHPPRD MPAAGSLGSS SRNRQRFNLN QYLPNFYPLD

**4501** MSEPQTKGTG ENSTCREPHA PYPPGYQRHF EAPAVESMPM SVYASTASCS

**4551** DVSACCEVES EVMMSDYESG DDGHFEEVTI PPLDSQQHTE VJDGSQRLEQ

**4601** TPLQFTHLEY NVTVQENSAA KJGTVKEDAP VGSLVMTVSA HDEDAGRDGE

**4651** IRJGTVKEDA PVGSLVMTVS AHDEDARRDG EIRJLDREST SHYWLTVFAT

**4701** DQGVVPLSSF IEIYIEVEDV NDNAPQTSEP VYYPEIMENS PKDVSVVQIE

**4751** AFDPDSSSND KJLDRESTSH YWLTVFATDQ GVVPLSSFIE LYIEVEDVND

**4801** NAPQTSEPVY YPEIMENSPK DVSVVQIEAF DPDSSSNDKJ FYKIRLPERJ

**4851** REPLYHVIAT DKDEGPNAEI SYSIEDGNEH GKJREPLYRV IATDKDEGPN

**4901** AEISYSIEDG NEHGKJEPLY HVIATDKDEG LNAEISYSIE DGNEHGKFFI

**4951** EPKJEPLYHV IATDKDEGPN AEISYSIEDG NEHGKFFIEP KJALEKNTNL

**5001** EARTKJALEK NTNVEARTKJ ALEKNTNVEA RTKJALEKNT NVEVRTKJFS

**5051** AAGEYDILSI KAVDNGRPQK SSTTRJFSAA GEYDILSIKA VDSGRPQKSS

**5101** TTRJNFARIV VNVSDTNDHA PWFTASSYKG RJNFARIVVN VSDTNDHAPW

**5151** FTTSSYKGRJ GKNAEVLYSI ESGDFGNIGN SFMIDPVLGS IKTAKJGKNA

**5201** EVLYSIESGN FGNIGNSFMI DPVLGSIKTA KJGKNAEVLY SIESGTIGNI

**5251** GNSFMIDPVL GSIKTAKJGS PPMSEITSVR IFVTNADNAS PKFTSKJGSP

**5301** PMSEITSVRI FVTSADNASP KFTSKJGSPP MSEITSVRIF VTIADNASTK

**5351** FTSKJALDFE TLPIYTLIIQ GTNMAGLSTN TTVLVHLQDE NDNAPVFMQA

**5401** EYTGLISESA SINSVVLTDR NVPQVIRAAD ADKJALDFET LPIYTLIIQG

**5451** TNMAGLSTNT TVLVHLQDEN DNAPVFMQAE YTGLISESAS INSVVLTDRN

**5501** VPRVIRAADA DKJAADADKD SNVLLVYHIV EPSVHTYFAI DSSTGAIHTV

**5551** LSLDYEETSI FHFTVQVHDM GTPRLFAEYA ANVTVHVIDI NDCPPVFAKP

**5601** LYEASLLLPT YKJFTQDVYS AVVKTNSTEA ETLAVITAIG NPINEPLFYH

**5651** ILNPDRRJTS GVLSTTGTPF DREAQEAFDV VVEVTEEHKP SAVAHVVVKV

**5701** IVEDQNDNAP VFVNLPYYAV VKJTDMFAIH PTSGVIVLTG RLDFLETKLY

**5751** EMEILAADRJ TDMFAIHPTS GVIVLTGRLD YLETKLYEME ILAADRJNGE

**5801** VHYYLKEHHE HFQIGPLGEK SLKKJDGGNP AFSAEVIVPI TVMNKAMPVF

**5851** EKPFYSAEIA ESIQVHSHVV HVQANSPEGL KVFYSITDGD PFSQFTINFN

**5901** TGVINVIAPL DFEAHPAYKJ AMPVFEKPFY SAEIAESIQV HSPVVHVQAN

**5951** SPEGLKVFYS ITDGDPFSQF TINFNTGVIN VIAPLDFPAH PAYKLSIRJL

**6001** SIRATDSLTG AHAEVFVDDI VDDINDNPPV FAQQSYAVTL SEASVIGTSV

**6051** VQVRATDSDS EPNRJLSIRA TDSLTGAHAE VFVDEIVDDI NDNPPVFAQQ

**6101** SYAVTLSEAS VIGTSVVQVR ATDSDSEPNR JTLDYEQSRQ HTIAVRAVDG

**6151** GMPTLSSDVI VTVDVTDLND NPPLFEQQIY EARJLYGSSG ISSMAKLTVH

**6201** IEQANECAPV ITAVTLSPSE LDRDPAYAIV TVDDCDQGAN GDIASLSIVA

**6251** GDLLQQFRJI LPPEMQLPKF SEPFYTFTVS EIVPIGTEID LIRAEHSGTV

**6301** LYSLVKJILP PEMQLPKFSE PFYTFTVSEV VPIGTEIDLI RAEHSGTVLY

**6351** SLVKJCTQDD HEMVASVDVS IQVKDANDNS PVFESSPYEA FIVENLPGGS

**6401** RVIQIRJCTQ DDHEMVASVD VSIQVKDASD NSPVFESSPY EAFIVENLPG

**6451** GSRVIQIRJV IQIRASDADN GTNGQVMYSL DQSQSVEVIE SFAINMETGW

**6501** ITTLKELDHE KJFTAEIYKG TVSELDPQGG VIAILSTTDA DSEEINRQVT

**6551** YFITGGDPLG QFAVETIQNE WKJFTAEIYK GTVSEPDPQG GVIAILSTTD

**6601** ADSEEINRQV TYFITGGDPL GQFAVETIQN EWKJFTAEIY KGTVSEQDPQ

**6651** GGVIAILSTT DADSEEINRQ VTYFITGGDP LGQFAVETIQ NEWKJGTVSE

**6701** DDPQGGVIAI LSTTDADSEE INRQVTYGIT GGDPLGQFAV ETIQNEWKVY

**6751** VKJGTVSEDD PQGGVIAILS TTDADSEEIN RQVTYSITGG DPLGQFAVET

**6801** IQNEWKVYVK JGTVSEDDPQ GGVIAILSTT DADSEEINRQ VTYFITGGDP

**6851** LAQFAVETIQ NEWKVYVKJA IVEVKVLDAN DNSPVCEYTL YSDTIPEDVL

**6901** PGKLIMQISA TDADIRJVLD ANDNSPVCEK TLYIDTIPED VLPGKLIMQI

**6951** SATDADIRJV LDANDNSPVC EKTLYTDTIP EDVLPGKLIM QISATDADIR

**7001** JATDGGGRFC QASIVLDLED VNDNAPEFSA DPYAITVFEN TEPGTLLTRV

**7051** QATDADAGLN RJKILYSLID SADGQFSINE LIGIIQLEKP LDRELQAVYT

**7101** LSLKJFSIDS KTGAVFIIEN LDYESSHEYY LTVEATDGGT PSLSDVATVN

**7151** VNVTDINDAT PVFSQDTYTT VISEDAVLEQ SVITVMADDA DGPSNSHIHY

**7201** SIIDGNQGSS FTIDPVRGEV KJFSIDSKTG AVFIIENLDY ESSHEYYLTV

**7251** EATDGGTPSL SDVATVNVNV TDINDVTPVF SQDTYTTVIS EDAVLEQSVI

**7301** TVMADDADGP SNSHIHYSII DGNQGSSFTI DPVRGEVKJV NTTTVNIDVS

**7351** DVNDNAPVFS RGNYSVIIQE NKPVGFSVLQ LVVTDEDSSH NGPPFFFTIV

**7401** TENDEKAFEV NPQGVLLTSS AIKJVNTTTV NIDVSDVNDN APVFSRGNYS

**7451** VIIQENKPVG FSVLQLVVTD EDSSHNGPPF FFTIVTGNDE KAFEVNPQGV

**7501** LLTSSAIKJG NYSVIIQENK PVGFSVLQLV VTDEDSSHNG PPFFFTIVTG

**7551** NDEKAFEVNA QGVLLTSSAI KRJGNYSVII QENKPVGFSV LQLVVTDEDS

**7601** SHNGPPFFFT IVTGNDEKAF EVNVQGVLLT SSAIKRJVAD NGKPQLSSLT

**7651** YIDIRVIEES IYPPAILPLE IAITSSGEEY SGGVIGKIHA TDQDVYDTLT

**7701** YSLDPQMDNL FSVSSTGGKJ VADNGKPQLS SLTYIDIRVI EESIYPPAIL

**7751** PLEISITSSG EEYSGGVIGK IHATDQDVYD TLTYSLDPQM DNLFSVSSTG

**7801** GKJVIHVTSP QFKAGPVKFE KJQLLHKIMS SVTDIEEIIG VRILNVFQKJ

**7851** QLLHKIVSSV TDIEEIIGVR ILNVFQKJEG RCPPVHHGCE DHPCPEGSEC

**7901** VSDPWEEKHT CVCPSGRJEG RCPPVHHGCE DPPCPEGSEC VSDPWEEKHT

**7951** CVCPSGRJHT CVCPSGRFGG CPGSSSMTLT GNSYVKYRJH TCVCPSGRFG

**8001** RCPGSSSMTL TGNSYVKYRJ FGQCPGSSSM TLTGNSYVKT RLTENENKJG

**8051** CMDSIYLNGQ ELPLNSKPRS YAHIEESVPV SPGCFLTATE DCASNPCQNG

**8101** GVCNPSPAGG YYCKCSALYI GTHCEISVNP CSSKPCLYGG TCVVDNGGFV

**8151** CQCRJYVFKR TPGKAKJYVF KSTPGKAKJS YAHIEESVDV SPGCFLTATE

**8201** DCASNPCQNG GVCNPSPAGG YYCKCSALYI GTHCEISVNP KSSKPCLYGG

**8251** TCVVDNGGFV CQCRGLYTGQ RJSYAHIEES VDVSPGCFLT ATEDCASNPC

**8301** QNGGVCNPSP AGGYYCKCSA LYIGTHCEIS VNPNSSKPCL YGGTCVVDNG

**8351** GFVCQCRGLY TGQRJDEPCK NGGTCFDSAD GAVCQCDSGF RGERJGRHCA

**8401** DAAPNQYVST PWNIGLAEGI GIVVFVAGIF LLVVVFVLCR KJGRHCEDAA

**8451** PNQYVSTPWN IGLAEGIGIV VFVAGIFLLV VVFVLCRKJN IYSDIPPQVP

**8501** VRYISYTPSI PSDSRNNLDR JKAVAVCSVA PNLPPPPPSN SPSDSDSIQK

**8551** PSWDFDYDTK VVDLDPCLSK JKPLEEKPSQ PYSARESLSS VQSLSSFQSE

**8601** SCDDNGYHWD TSDWMPSVPL PDIQEFPNYE VIDEQTPLYS ADPNAIDTDY

**8651** YPGGYDIESD FPPPPEDFPA ADELPPLPPE FSNQFESIHP PRDMPAAGSL

**8701** GSSSRJKPLE EKPSQPYSAR ESLSEVQSLS SFQSESCDDN GYHWDTSDWM

**8751** PSVPLQDIQE FPNYEVIDEQ TPLYSADPNA IDTDYYPGGY DIESDFPPPP

**8801** EDFPAADELP PLPPEFSNQF ESIHPPRDMP AAGSLGSSSR JKPLEEKPSQ

**8851** PYSARESLSE VQSLSSFQSE SCDDNGYHWD TSDWMPSVPL PDIQEFPNYE

**8901** VIDPQTPLYS ADPNAIDTDY YPGGYDIESD FPPPPEDFPA ADELPPLPPE

**8951** FSNQFESIHP PRDMPAAGSL GSSSRJESLS EVQSLSSFQS ESCDDNGYHW

**9001** DTSDWMPSVP LPDIQEFPNY EVIDEQTPLY SADPNAIDTD YYPGGYDIES

**9051** DFPPPPEDFP AADELPPLPP EFSNQFESIH PPRDMPAAGS LGSSRRNRJE

**9101** SLSEVQSLSS FQSESCDDNG YHWDTSDWMP SVPLPDIQEF PNYEVIDEQT

**9151** PLYSADPNAI DTDYYPGGYD IESDFPPPPE DFPAADELPP LPPEFSNQFE

**9201** SIHPPRDMPA AGSLGSSWRN RJVLGANSNP PEFTQTAYKA AFDENVPIGT

**9251** TIMSLSAVDP DEGENGYVTY SIANLNHVPF AIDHFTGAVS TSENLDYELM

**9301** PRVYTLRJVL GANSNPPEFT QTAYKAAFDE NVPIGTTVMS LSAVDPDEGE

**9351** NGYVTYSIAN LNHVPFAIDH FTGAVSTSEN LDYELMPRVY TLRJVLGANS

**9401** NPPEFTQTAY KAAFDENVPI GTTVMSLSAV DPDEGENGYV TYSIANLNHV

**9451** PFAIDHFTGA VSTSENLDYE LMPRVYTLRJ INCEGTIPRD LGVGEQITTV

**9501** SAIDADELQL VQYQIEAGNE LDFFSLNPNS GVLSLKRJIN CEGTIPRDLG

**9551** VGEQITTVSA IDADELQLVQ YQIEAGNELD LFSLNPNSGV LSLKRJINCE

**9601** GTIPRDLGVG EQITTVSAID ADELQLVQYQ IEAGNELDFF SLNPNSGVLS

**9651** LKRJEVHSEI IQVEATDKDL GPNGHVTYSI LTDTDTFSID SVTGVVNIAR

**9701** PLDRELQHEH SLKJEVHSEI IQVEATDKDL GPNGHVTYSI VTDTDTFSID

**9751** SVTGVVNIAR PLDRELQHEH SLK

**Start - End Observed Mr(expt) Mr(calc) ppm Miss Sequence**

**1658 - 1669 638.3507 1274.6869 1274.6870 -0 0 R.IFVTIADNASPK.F**  ([Ions score 51](http://mbp-mascot4/mascot/cgi/peptide_view.pl?file=../data/20091023/F006342.dat&query=1986&hit=1&index=IPI00031411&px=1&section=5&ave_thresh=22))

**1982 - 1992 628.8305 1255.6464 1255.6449 1 0 K.FTQDVYSAVVK.E**  ([Ions score 22](http://mbp-mascot4/mascot/cgi/peptide_view.pl?file=../data/20091023/F006342.dat&query=1915&hit=1&index=IPI00031411&px=1&section=5&ave_thresh=22))

**2031 - 2044 719.8631 1437.7116 1437.7100 1 0 R.TSGVLSTTGTPFDR.E**  ([Ions score 41](http://mbp-mascot4/mascot/cgi/peptide_view.pl?file=../data/20091023/F006342.dat&query=2409&hit=1&index=IPI00031411&px=1&section=5&ave_thresh=22))

**A818 A3**

Match to: **IPI00031411** Score: **1404**

**Gene_Symbol=FAT1 Protocadherin Fat 1 lng=4591 # SP[4593,D,22,D]SNP[4646,g,1064,R]SNP[4677,r,1064,R]SNP[4713,i,1125,I]SNP[4791,l,1125,I]SNP[4844,i,1252,I]SNP[4856,h,1273,H]SNP[4889,r,1273,H]SNP[4931,l,1283,P]SNP[4969,p,1283,P]SNP[5000,l,129,V]SNP[5**

Found in search of C:\mgf\Orbidata\081031_ISW1295_A818_A3.mgf

Nominal mass (M_r_): **1068827**; Calculated pI value: **4.55**

NCBI BLAST search of [IPI00031411](http://www.ncbi.nlm.nih.gov/blast/Blast.cgi?ALIGNMENTS=50&ALIGNMENT_VIEW=Pairwise&AUTO_FORMAT=Semiauto&CDD_SEARCH=on&CLIENT=web&COMPOSITION_BASED_STATISTICS=on&DATABASE=nr&DESCRIPTIONS=100&ENTREZ_QUERY=(none)&EXPECT=10&FILTER=L&FORMAT_BLOCK_ON_RESPAGE=None&FORMAT_OBJECT=Alignment&FORMAT_TYPE=HTML&GAPCOSTS=11+1&I_THRESH=0.001&LAYOUT=TwoWindows&MATRIX_NAME=BLOSUM62&NCBI_GI=on&PAGE=Proteins&PROGRAM=blastp&QUERY=IPI00031411&SERVICE=plain&SET_DEFAULTS.x=21&SET_DEFAULTS.y=7&SHOW_OVERVIEW=on&WORD_SIZE=3&END_OF_HTTPGET=Yes) against nr

Unformatted [sequence string](http://mbp-mascot4/mascot/cgi/getseq.pl?IPI_human+IPI00031411+seq) for pasting into other applications

Fixed modifications: Carbamidomethyl (C)

Variable modifications: Oxidation (M)

Cleavage by TrypsinMSIPI, a mixture of enzymes:

cuts C-term side of KR unless next residue is P

cuts C-term side of J

cuts N-term side of J

Sequence Coverage: **6%**

Matched peptides shown in **Bold Red**

**1** MGRHLALLLL LLLLFQHFGD SDGSQRLEQT PLQFTHLEYN VTVQENSAAK

**51** TYVGHPVK**MG VYITHPAWEV R**YKIVSGDSE NLFK**AEEYIL GDFCFLR**IRT

**101** KGGNTAILNR EVKDHYTLIV KALEKNTNVE ARTKVRVQVL DTNDLRPLFS

**151** PTSYSVSLPE NTAIRTSIAR VSATDADIGT NGEFYYSFKD RTDMFAIHPT

**201** SGVIVLTGRL DYLETK**LYEM EILAADR**GMK **LYGSSGISSM AK**LTVHIEQA

**251** NECAPVITAV TLSPSELDRD PAYAIVTVDD CDQGANGDIA SLSIVAGDLL

**301** QQFRTVRSFP GSKEYKVKAI GGIDWDSHPF GYNLTLQAKD KGTPPQFSSV

**351** KVIHVTSPQF KAGPVKFEKD VYR**AEISEFA PPNTPVVMVK** AIPAYSHLRY

**401** VFKSTPGKAK FSLNYNTGLI SILEPVKRQQ AAHFELEVTT SDRKASTKVL

**451** VK**VLGANSNP PEFTQTAYK**A AFDENVPIGT TVMSLSAVDP DEGENGYVTY

**501** SIANLNHVPF AIDHFTGAVS TSENLDYELM PRVYTLRIR**A SDWGLPYR**RE

**551** VEVLATITLN NLNDNTPLFE KINCEGTIPR DLGVGEQITT VSAIDADELQ

**601** LVQYQIEAGN ELDFFSLNPN SGVLSLKRSL MDGLGAKVSF HSLRITATDG

**651** ENFATPLYIN ITVAASHK**LV NLQCEETGVA K**MLAEKLLQA NK**LHNQGEVE**

**701 DIFFDSHSVN AHIPQFR**STL PTGIQVKENQ PVGSSVIFMN STDLDTGFNG

**751** KLVYAVSGGN EDSCFMIDME TGMLKILSPL DRETTDKYTL NITVYDLGIP

**801** QKAAWRLLHV VVVDANDNPP EFLQESYFVE VSEDKEVHSE IIQVEATDKD

**851** LGPNGHVTYS IVTDTDTFSI DSVTGVVNIA RPLDRELQHE HSLKIEARDQ

**901** AREEPQLFST VVVKVSLEDV NDNPPTFIPP NYRVK**VREDL PEGTVIMWLE**

**951 AHDPDLGQSG QVRYSLLDHG EGNFDVDK**LS GAVR**IVQQLD FEK**KQVYNLT

**1001** VRAKDKGKPV SLSSTCYVEV EVVDVNENLH PPVFSSFVEK GTVKEDAPVG

**1051** SLVMTVSAHD EDARRDGEIR YSIRDGSGVG VFK**IGEETGV IETSDRLDR**E

**1101** STSHYWLTVF ATDQGVVPLS SFIEIYIEVE DVNDNAPQTS EPVYYPEIME

**1151** NSPK**DVSVVQ IEAFDPDSSS NDK**LMYK**ITS GNPQGFFSIH PK**TGLITTTS

**1201** RKLDREQQDE HILEVTVTDN GSPPKSTIAR VIVK**ILDEND NKPQFLQK**FY

**1251** KIRLPEREKP DRERNARREP LYHVIATDKD EGPNAEISYS IEDGNEHGK**F**

**1301 FIEPK**TGVVS SKR**FSAAGEY DILSIK**AVDN GRPQKSSTTR LHIEWISKPK

**1351** PSLEPISFEE SFFTFTVMES DPVAHMIGVI SVEPPGIPLW FDITGGNYDS

**1401** HFDVDK**GTGT IIVAKPLDAE QK**SNYNLTVE ATDGTTTILT QVFIK**VIDTN**

**1451 DHRPQFSTSK** YEVVIPEDTA PETEILQISA VDQDEKNK**LI YTLQSSR**DPL

**1501** SLKKFR**LDPA TGSLYTSEKL DHEAVHQHTL TVMVR**DQDVP VKRNFARIVV

**1551** NVSDTNDHAP WFTASSYKGR VYESAAVGSV VLQVTALDKD KGKNAEVLYS

**1601** IESGTFGNIG NSFMIDPVLG SIKTAKELDR **SNQAEYDLMV K**ATDK**GSPPM**

**1651 SEITSVRIFV TIADNASPK**F TSKEYSVELS ETVSIGSFVG MVTAHSQSSV

**1701** VYEIK**DGNTG DAFDINPHSG TIITQK**ALDF ETLPIYTLII QGTNMAGLST

**1751** NTTVLVHLQD ENDNAPVFMQ AEYTGLISES ASINSVVLTD RNVPLVIRAA

**1801** DADKDSNALL VYHIVEPSVH TYFAIDSSTG AIHTVLSLDY EETSIFHFTV

**1851** QVHDMGTPRL FAEYAANVTV HVIDINDCPP VFAKPLYEAS LLLPTYKGVK

**1901** VITVNATDAD SSAFSQLIYS ITEGNIGEKF SMDYKTGALT VQNTTQLRSR

**1951** YELTVRASDG R**FAGLTSVK**I NVKESKESHL K**FTQDVYSAV VK**ENSTEAET

**2001** LAVITAIGNP INEPLFYHIL NPDRRFKISR **TSGVLSTTGT PFDR**EQQEAF

**2051** DVVVEVTEEH KPSAVAHVVV KVIVEDQNDN APVFVNLPYY AVVKVDTEVG

**2101** HVIRYVTAVD RDSGRNGEVH YYLKEHHEHF QIGPLGEISL KK**QFELDTLN**

**2151 K**EYLVTVVAK DGGNPAFSAE VIVPITVMNK AMPVFEKPFY SAEIAESIQV

**2201** HSPVVHVQAN SPEGLKVFYS ITDGDPFSQF TINFNTGVIN VIAPLDFEAH

**2251** PAYKLSIRAT DSLTGAHAEV FVDIIVDDIN DNPPVFAQQS YAVTLSEASV

**2301** IGTSVVQVRA TDSDSEPNRG ISYQMFGNHS KSHDHFHVDS STGLISLLRT

**2351** LDYEQSRQHT IFVRAVDGGM PTLSSDVIVT VDVTDLNDNP PLFEQQIYEA

**2401** RISEHAPHGH FVTCVKAYDA DSSDIDKLQY SILSGNDHK**H FVIDSATGII**

**2451 TLSNLHR**HAL KPFYSLNLSV SDGVFRSSTQ VHVTVIGGNL HSPAFLQNEY

**2501** EVELAENAPL HTLVMEVKTT DGDSGIYGHV TYHIVNDFAK DRFYINER**GQ**

**2551 IFTLEK**LDRE TPAEKVISVR LMAKDAGGKV AFCTVNVILT DDNDNAPQFR

**2601** ATK**YEVNIGS SAAK**GTSVVK VLASDADEGS NADITYAIEA DSESVKENLE

**2651** INKLSGVITT KESLIGLENE FFTFFVRAVD NGSPSK**ESVV LVYVK**ILPPE

**2701** MQLPKFSEPF YTFTVSEDVP IGTEIDLIR**A EHSGTVLYSL VK**GNTPESNR

**2751** DESFVIDRQS GRLKLEKSLD HETTKWYQFS ILARCTQDDH EMVASVDVSI

**2801** QVKDANDNSP VFESSPYEAF IVENLPGGSR VIQIRASDAD SGTNGQVMYS

**2851** LDQSQSVEVI ESFAINMETG WITTLKELDH EKRDNYQIKV VASDHGEKIQ

**2901** LSSTAIVDVT VTDVNDSPPR FTAEIYKGTV SEDDPQGGVI AILSTTDADS

**2951** EEINRQVTYF ITGGDPLGQF AVETIQNEWK VYVKKPLDRE KR**DNYLLTIT**

**3001 ATDGTFSSK**A IVEVKVLDAN DNSPVCEKTL YSDTIPEDVL PGKLIMQISA

**3051** TDADIR**SNAE ITYTLLGSGA EK**FKLNPDTG ELK**TSTPLDR EEQAVYHLLV**

**3101 R**ATDGGGRFC QASIVLTLED VNDNAPEFSA DPYAITVFEN TEPGTLLTRV

**3151** QATDADAGLN RKILYSLIDS ADGQFSINEL SGIIQLEKPL DR**ELQAVYTL**

**3201 SLK**AVDQGLP RRLTATGTVI VSVLDINDNP PVFEYREYGA TVSEDILVGT

**3251** EVLQVYAASR DIEANAEITY SIISGNEHGK FSIDSKTGAV FIIENLDYES

**3301** SHEYYLTVEA TDGGTPSLSD VATVNVNVTD INDNTPVFSQ DTYTTVISED

**3351** AVLEQSVITV MADDADGPSN SHIHYSIIDG NQGSSFTIDP VRGEVKVTKL

**3401** LDRETISGYT LTVQASDNGS PPRVNTTTVN IDVSDVNDNA PVFSRGNYSV

**3451** IIQENKPVGF SVLQLVVTDE DSSHNGPPFF FTIVTGNDEK **AFEVNPQGVL**

**3501 LTSSAIK**RKE KDHYLLQVKV ADNGKPQLSS LTYIDIRVIE ESIYPPAILP

**3551** LEIFITSSGE EYSGGVIGKI HATDQDVYDT LTYSLDPQMD NLFSVSSTGG

**3601** KLIAHKKLDI GQYLLNVSVT DGKFTTVADI TVHIRQVTQE MLNHTIAIRF

**3651** ANLTPEEFVG DYWRNFQRAL RNILGVRRND IQIVSLQSSE PHPHLDVLLF

**3701** VEKPGSAQIS TKQLLHKINS SVTDIEEIIG VR**ILNVFQKL CAGLDCPWK**F

**3751** CDEK**VSVDES VMSTHSTARL SFVTPR**HHRA AVCLCKEGRC PPVHHGCEDD

**3801** PCPEGSECVS DPWEEKHTCV CPSGRFGQCP GSSSMTLTGN SYVKYRLTEN

**3851** ENKLEMKLTM RLRTYSTHAV VMYAR**GTDYS ILEIHHGR**LQ YKFDCGSGPG

**3901** IVSVQSIQVN DGQWHAVALE VNGNYAR**LVL DQVHTASGTA PGTLK**TLNLD

**3951** NYVFFGGHIR QQGTRHGRSP QVGNGFRGCM DSIYLNGQEL PLNSKPRSYA

**4001** HIEESVDVSP GCFLTATEDC ASNPCQNGGV CNPSPAGGYY CKCSALYIGT

**4051** HCEISVNPCS SKPCLYGGTC VVDNGGFVCQ CRGLYTGQRC QLSPYCKDEP

**4101** CKNGGTCFDS LDGAVCQCDS GFRGERCQSD IDECSGNPCL HGALCENTHG

**4151** SYHCNCSHEY RGRHCEDAAP NQYVSTPWNI GLAEGIGIVV FVAGIFLLVV

**4201** VFVLCRKMIS RKKKHQAEPK DKHLGPATAF LQRPYFDSKL NKNIYSDIPP

**4251** QVPVRPISYT PSIPSDSRNN LDRNSFEGSA IPEHPEFSTF NPESVHGHRK

**4301** AVAVCSVAPN LPPPPPSNSP SDSDSIQKPS WDFDYDTKVV DLDPCLSKKP

**4351** LEEKPSQPYS ARESLSEVQS LSSFQSESCD DNGYHWDTSD WMPSVPLPDI

**4401** QEFPNYEVID EQTPLYSADP NAIDTDYYPG GYDIESDFPP PPEDFPAADE

**4451** LPPLPPEFSN QFESIHPPRD MPAAGSLGSS SRNRQRFNLN QYLPNFYPLD

**4501** MSEPQTKGTG ENSTCREPHA PYPPGYQRHF EAPAVESMPM SVYASTASCS

**4551** DVSACCEVES EVMMSDYESG DDGHFEEVTI PPLDSQQHTE VJDGSQRLEQ

**4601** TPLQFTHLEY NVTVQENSAA KJGTVKEDAP VGSLVMTVSA HDEDAGRDGE

**4651** IRJGTVKEDA PVGSLVMTVS AHDEDARRDG EIRJLDREST SHYWLTVFAT

**4701** DQGVVPLSSF IEIYIEVEDV NDNAPQTSEP VYYPEIMENS PKDVSVVQIE

**4751** AFDPDSSSND KJLDRESTSH YWLTVFATDQ GVVPLSSFIE LYIEVEDVND

**4801** NAPQTSEPVY YPEIMENSPK DVSVVQIEAF DPDSSSNDKJ FYKIRLPERJ

**4851** REPLYHVIAT DKDEGPNAEI SYSIEDGNEH GKJREPLYRV IATDKDEGPN

**4901** AEISYSIEDG NEHGKJEPLY HVIATDKDEG LNAEISYSIE DGNEHGKFFI

**4951** EPKJEPLYHV IATDKDEGPN AEISYSIEDG NEHGKFFIEP KJALEKNTNL

**5001** EARTKJALEK NTNVEARTKJ ALEKNTNVEA RTKJALEKNT NVEVRTKJFS

**5051** AAGEYDILSI KAVDNGRPQK SSTTRJFSAA GEYDILSIKA VDSGRPQKSS

**5101** TTRJNFARIV VNVSDTNDHA PWFTASSYKG RJNFARIVVN VSDTNDHAPW

**5151** FTTSSYKGRJ GKNAEVLYSI ESGDFGNIGN SFMIDPVLGS IKTAKJGKNA

**5201** EVLYSIESGN FGNIGNSFMI DPVLGSIKTA KJGKNAEVLY SIESGTIGNI

**5251** GNSFMIDPVL GSIKTAKJGS PPMSEITSVR IFVTNADNAS PKFTSKJGSP

**5301** PMSEITSVRI FVTSADNASP KFTSKJGSPP MSEITSVRIF VTIADNASTK

**5351** FTSKJALDFE TLPIYTLIIQ GTNMAGLSTN TTVLVHLQDE NDNAPVFMQA

**5401** EYTGLISESA SINSVVLTDR NVPQVIRAAD ADKJALDFET LPIYTLIIQG

**5451** TNMAGLSTNT TVLVHLQDEN DNAPVFMQAE YTGLISESAS INSVVLTDRN

**5501** VPRVIRAADA DKJAADADKD SNVLLVYHIV EPSVHTYFAI DSSTGAIHTV

**5551** LSLDYEETSI FHFTVQVHDM GTPRLFAEYA ANVTVHVIDI NDCPPVFAKP

**5601** LYEASLLLPT YKJFTQDVYS AVVKTNSTEA ETLAVITAIG NPINEPLFYH

**5651** ILNPDRRJTS GVLSTTGTPF DREAQEAFDV VVEVTEEHKP SAVAHVVVKV

**5701** IVEDQNDNAP VFVNLPYYAV VKJTDMFAIH PTSGVIVLTG RLDFLETKLY

**5751** EMEILAADRJ TDMFAIHPTS GVIVLTGRLD YLETKLYEME ILAADRJNGE

**5801** VHYYLKEHHE HFQIGPLGEK SLKKJDGGNP AFSAEVIVPI TVMNKAMPVF

**5851** EKPFYSAEIA ESIQVHSHVV HVQANSPEGL KVFYSITDGD PFSQFTINFN

**5901** TGVINVIAPL DFEAHPAYKJ AMPVFEKPFY SAEIAESIQV HSPVVHVQAN

**5951** SPEGLKVFYS ITDGDPFSQF TINFNTGVIN VIAPLDFPAH PAYKLSIRJL

**6001** SIRATDSLTG AHAEVFVDDI VDDINDNPPV FAQQSYAVTL SEASVIGTSV

**6051** VQVRATDSDS EPNRJLSIRA TDSLTGAHAE VFVDEIVDDI NDNPPVFAQQ

**6101** SYAVTLSEAS VIGTSVVQVR ATDSDSEPNR JTLDYEQSRQ HTIAVRAVDG

**6151** GMPTLSSDVI VTVDVTDLND NPPLFEQQIY EARJLYGSSG ISSMAKLTVH

**6201** IEQANECAPV ITAVTLSPSE LDRDPAYAIV TVDDCDQGAN GDIASLSIVA

**6251** GDLLQQFRJI LPPEMQLPKF SEPFYTFTVS EIVPIGTEID LIRAEHSGTV

**6301** LYSLVKJILP PEMQLPKFSE PFYTFTVSEV VPIGTEIDLI RAEHSGTVLY

**6351** SLVKJCTQDD HEMVASVDVS IQVKDANDNS PVFESSPYEA FIVENLPGGS

**6401** RVIQIRJCTQ DDHEMVASVD VSIQVKDASD NSPVFESSPY EAFIVENLPG

**6451** GSRVIQIRJV IQIRASDADN GTNGQVMYSL DQSQSVEVIE SFAINMETGW

**6501** ITTLKELDHE KJFTAEIYKG TVSELDPQGG VIAILSTTDA DSEEINRQVT

**6551** YFITGGDPLG QFAVETIQNE WKJFTAEIYK GTVSEPDPQG GVIAILSTTD

**6601** ADSEEINRQV TYFITGGDPL GQFAVETIQN EWKJFTAEIY KGTVSEQDPQ

**6651** GGVIAILSTT DADSEEINRQ VTYFITGGDP LGQFAVETIQ NEWKJGTVSE

**6701** DDPQGGVIAI LSTTDADSEE INRQVTYGIT GGDPLGQFAV ETIQNEWKVY

**6751** VKJGTVSEDD PQGGVIAILS TTDADSEEIN RQVTYSITGG DPLGQFAVET

**6801** IQNEWKVYVK JGTVSEDDPQ GGVIAILSTT DADSEEINRQ VTYFITGGDP

**6851** LAQFAVETIQ NEWKVYVKJA IVEVKVLDAN DNSPVCEYTL YSDTIPEDVL

**6901** PGKLIMQISA TDADIRJVLD ANDNSPVCEK TLYIDTIPED VLPGKLIMQI

**6951** SATDADIRJV LDANDNSPVC EKTLYTDTIP EDVLPGKLIM QISATDADIR

**7001** JATDGGGRFC QASIVLDLED VNDNAPEFSA DPYAITVFEN TEPGTLLTRV

**7051** QATDADAGLN RJKILYSLID SADGQFSINE LIGIIQLEKP LDRELQAVYT

**7101** LSLKJFSIDS KTGAVFIIEN LDYESSHEYY LTVEATDGGT PSLSDVATVN

**7151** VNVTDINDAT PVFSQDTYTT VISEDAVLEQ SVITVMADDA DGPSNSHIHY

**7201** SIIDGNQGSS FTIDPVRGEV KJFSIDSKTG AVFIIENLDY ESSHEYYLTV

**7251** EATDGGTPSL SDVATVNVNV TDINDVTPVF SQDTYTTVIS EDAVLEQSVI

**7301** TVMADDADGP SNSHIHYSII DGNQGSSFTI DPVRGEVKJV NTTTVNIDVS

**7351** DVNDNAPVFS RGNYSVIIQE NKPVGFSVLQ LVVTDEDSSH NGPPFFFTIV

**7401** TENDEKAFEV NPQGVLLTSS AIKJVNTTTV NIDVSDVNDN APVFSRGNYS

**7451** VIIQENKPVG FSVLQLVVTD EDSSHNGPPF FFTIVTGNDE KAFEVNPQGV

**7501** LLTSSAIKJG NYSVIIQENK PVGFSVLQLV VTDEDSSHNG PPFFFTIVTG

**7551** NDEKAFEVNA QGVLLTSSAI KRJGNYSVII QENKPVGFSV LQLVVTDEDS

**7601** SHNGPPFFFT IVTGNDEKAF EVNVQGVLLT SSAIKRJVAD NGKPQLSSLT

**7651** YIDIRVIEES IYPPAILPLE IAITSSGEEY SGGVIGKIHA TDQDVYDTLT

**7701** YSLDPQMDNL FSVSSTGGKJ VADNGKPQLS SLTYIDIRVI EESIYPPAIL

**7751** PLEISITSSG EEYSGGVIGK IHATDQDVYD TLTYSLDPQM DNLFSVSSTG

**7801** GKJVIHVTSP QFKAGPVKFE KJQLLHKIMS SVTDIEEIIG VRILNVFQKJ

**7851** QLLHKIVSSV TDIEEIIGVR ILNVFQKJEG RCPPVHHGCE DHPCPEGSEC

**7901** VSDPWEEKHT CVCPSGRJEG RCPPVHHGCE DPPCPEGSEC VSDPWEEKHT

**7951** CVCPSGRJHT CVCPSGRFGG CPGSSSMTLT GNSYVKYRJH TCVCPSGRFG

**8001** RCPGSSSMTL TGNSYVKYRJ FGQCPGSSSM TLTGNSYVKT RLTENENKJG

**8051** CMDSIYLNGQ ELPLNSKPRS YAHIEESVPV SPGCFLTATE DCASNPCQNG

**8101** GVCNPSPAGG YYCKCSALYI GTHCEISVNP CSSKPCLYGG TCVVDNGGFV

**8151** CQCRJYVFKR TPGKAKJYVF KSTPGKAKJS YAHIEESVDV SPGCFLTATE

**8201** DCASNPCQNG GVCNPSPAGG YYCKCSALYI GTHCEISVNP KSSKPCLYGG

**8251** TCVVDNGGFV CQCRGLYTGQ RJSYAHIEES VDVSPGCFLT ATEDCASNPC

**8301** QNGGVCNPSP AGGYYCKCSA LYIGTHCEIS VNPNSSKPCL YGGTCVVDNG

**8351** GFVCQCRGLY TGQRJDEPCK NGGTCFDSAD GAVCQCDSGF RGERJGRHCA

**8401** DAAPNQYVST PWNIGLAEGI GIVVFVAGIF LLVVVFVLCR KJGRHCEDAA

**8451** PNQYVSTPWN IGLAEGIGIV VFVAGIFLLV VVFVLCRKJN IYSDIPPQVP

**8501** VRYISYTPSI PSDSRNNLDR JKAVAVCSVA PNLPPPPPSN SPSDSDSIQK

**8551** PSWDFDYDTK VVDLDPCLSK JKPLEEKPSQ PYSARESLSS VQSLSSFQSE

**8601** SCDDNGYHWD TSDWMPSVPL PDIQEFPNYE VIDEQTPLYS ADPNAIDTDY

**8651** YPGGYDIESD FPPPPEDFPA ADELPPLPPE FSNQFESIHP PRDMPAAGSL

**8701** GSSSRJKPLE EKPSQPYSAR ESLSEVQSLS SFQSESCDDN GYHWDTSDWM

**8751** PSVPLQDIQE FPNYEVIDEQ TPLYSADPNA IDTDYYPGGY DIESDFPPPP

**8801** EDFPAADELP PLPPEFSNQF ESIHPPRDMP AAGSLGSSSR JKPLEEKPSQ

**8851** PYSARESLSE VQSLSSFQSE SCDDNGYHWD TSDWMPSVPL PDIQEFPNYE

**8901** VIDPQTPLYS ADPNAIDTDY YPGGYDIESD FPPPPEDFPA ADELPPLPPE

**8951** FSNQFESIHP PRDMPAAGSL GSSSRJESLS EVQSLSSFQS ESCDDNGYHW

**9001** DTSDWMPSVP LPDIQEFPNY EVIDEQTPLY SADPNAIDTD YYPGGYDIES

**9051** DFPPPPEDFP AADELPPLPP EFSNQFESIH PPRDMPAAGS LGSSRRNRJE

**9101** SLSEVQSLSS FQSESCDDNG YHWDTSDWMP SVPLPDIQEF PNYEVIDEQT

**9151** PLYSADPNAI DTDYYPGGYD IESDFPPPPE DFPAADELPP LPPEFSNQFE

**9201** SIHPPRDMPA AGSLGSSWRN RJVLGANSNP PEFTQTAYKA AFDENVPIGT

**9251** TIMSLSAVDP DEGENGYVTY SIANLNHVPF AIDHFTGAVS TSENLDYELM

**9301** PRVYTLRJVL GANSNPPEFT QTAYKAAFDE NVPIGTTVMS LSAVDPDEGE

**9351** NGYVTYSIAN LNHVPFAIDH FTGAVSTSEN LDYELMPRVY TLRJVLGANS

**9401** NPPEFTQTAY KAAFDENVPI GTTVMSLSAV DPDEGENGYV TYSIANLNHV

**9451** PFAIDHFTGA VSTSENLDYE LMPRVYTLRJ INCEGTIPRD LGVGEQITTV

**9501** SAIDADELQL VQYQIEAGNE LDFFSLNPNS GVLSLKRJIN CEGTIPRDLG

**9551** VGEQITTVSA IDADELQLVQ YQIEAGNELD LFSLNPNSGV LSLKRJINCE

**9601** GTIPRDLGVG EQITTVSAID ADELQLVQYQ IEAGNELDFF SLNPNSGVLS

**9651** LKRJEVHSEI IQVEATDKDL GPNGHVTYSI LTDTDTFSID SVTGVVNIAR

**9701** PLDRELQHEH SLKJEVHSEI IQVEATDKDL GPNGHVTYSI VTDTDTFSID

**9751** SVTGVVNIAR PLDRELQHEH SLK

**Start - End Observed Mr(expt) Mr(calc) ppm Miss Sequence**

**59 - 71 779.8946 1557.7746 1557.7762 -1 0 K.MGVYITHPAWEVR.Y**  ([Ions score 25](http://mbp-mascot4/mascot/cgi/peptide_view.pl?file=../data/20091023/F006344.dat&query=2728&hit=1&index=IPI00031411&px=1&section=5&ave_thresh=22))

**59 - 71 525.5979 1573.7719 1573.7711 0 0 K.MGVYITHPAWEVR.Y**  Oxidation (M) ([Ions score 23](http://mbp-mascot4/mascot/cgi/peptide_view.pl?file=../data/20091023/F006344.dat&query=2753&hit=1&index=IPI00031411&px=1&section=5&ave_thresh=22))

**85 - 97 816.8907 1631.7668 1631.7654 1 0 K.AEEYILGDFCFLR.I**  ([Ions score 69](http://mbp-mascot4/mascot/cgi/peptide_view.pl?file=../data/20091023/F006344.dat&query=2846&hit=1&index=IPI00031411&px=1&section=5&ave_thresh=22))

**217 - 227 662.3346 1322.6546 1322.6540 0 0 K.LYEMEILAADR.G**  ([Ions score 56](http://mbp-mascot4/mascot/cgi/peptide_view.pl?file=../data/20091023/F006344.dat&query=2275&hit=1&index=IPI00031411&px=1&section=5&ave_thresh=22))

**217 - 227 662.3347 1322.6547 1322.6540 1 0 K.LYEMEILAADR.G**  ([Ions score 50](http://mbp-mascot4/mascot/cgi/peptide_view.pl?file=../data/20091023/F006344.dat&query=2276&hit=1&index=IPI00031411&px=1&section=5&ave_thresh=22))

**231 - 242 600.7991 1199.5837 1199.5856 -2 0 K.LYGSSGISSMAK.L**  ([Ions score 48](http://mbp-mascot4/mascot/cgi/peptide_view.pl?file=../data/20091023/F006344.dat&query=1794&hit=1&index=IPI00031411&px=1&section=5&ave_thresh=22))

**374 - 390 914.9788 1827.9430 1827.9441 -1 0 R.AEISEFAPPNTPVVMVK.A**  ([Ions score 24](http://mbp-mascot4/mascot/cgi/peptide_view.pl?file=../data/20091023/F006344.dat&query=3093&hit=1&index=IPI00031411&px=1&section=5&ave_thresh=22))

**374 - 390 922.9757 1843.9369 1843.9390 -1 0 R.AEISEFAPPNTPVVMVK.A**  Oxidation (M) ([Ions score 26](http://mbp-mascot4/mascot/cgi/peptide_view.pl?file=../data/20091023/F006344.dat&query=3129&hit=1&index=IPI00031411&px=1&section=5&ave_thresh=22))

**453 - 469 918.9592 1835.9039 1835.9054 -1 0 K.VLGANSNPPEFTQTAYK.A**  ([Ions score 50](http://mbp-mascot4/mascot/cgi/peptide_view.pl?file=../data/20091023/F006344.dat&query=3114&hit=1&index=IPI00031411&px=1&section=5&ave_thresh=22))

**453 - 469 918.9598 1835.9050 1835.9054 -0 0 K.VLGANSNPPEFTQTAYK.A**  ([Ions score 50](http://mbp-mascot4/mascot/cgi/peptide_view.pl?file=../data/20091023/F006344.dat&query=3115&hit=1&index=IPI00031411&px=1&section=5&ave_thresh=22))

**540 - 548 532.7614 1063.5083 1063.5087 -0 0 R.ASDWGLPYR.R**  ([Ions score 28](http://mbp-mascot4/mascot/cgi/peptide_view.pl?file=../data/20091023/F006344.dat&query=1180&hit=1&index=IPI00031411&px=1&section=5&ave_thresh=22))

**540 - 548 532.7618 1063.5090 1063.5087 0 0 R.ASDWGLPYR.R**  ([Ions score 36](http://mbp-mascot4/mascot/cgi/peptide_view.pl?file=../data/20091023/F006344.dat&query=1181&hit=1&index=IPI00031411&px=1&section=5&ave_thresh=22))

**669 - 681 730.8752 1459.7358 1459.7341 1 0 K.LVNLQCEETGVAK.M**  ([Ions score 54](http://mbp-mascot4/mascot/cgi/peptide_view.pl?file=../data/20091023/F006344.dat&query=2586&hit=1&index=IPI00031411&px=1&section=5&ave_thresh=22))

**693 - 717 734.8579 2935.4025 2935.4002 1 0 K.LHNQGEVEDIFFDSHSVNAHIPQFR.S**  ([Ions score 26](http://mbp-mascot4/mascot/cgi/peptide_view.pl?file=../data/20091023/F006344.dat&query=4217&hit=1&index=IPI00031411&px=1&section=5&ave_thresh=22))

**936 - 963 787.6445 3146.5490 3146.5455 1 1 K.VREDLPEGTVIMWLEAHDPDLGQSGQVR.Y**  ([Ions score 52](http://mbp-mascot4/mascot/cgi/peptide_view.pl?file=../data/20091023/F006344.dat&query=4304&hit=1&index=IPI00031411&px=1&section=5&ave_thresh=22))

**964 - 978 854.8946 1707.7747 1707.7740 0 0 R.YSLLDHGEGNFDVDK.L**  ([Ions score 25](http://mbp-mascot4/mascot/cgi/peptide_view.pl?file=../data/20091023/F006344.dat&query=2937&hit=1&index=IPI00031411&px=1&section=5&ave_thresh=22))

**985 - 993 560.3066 1118.5987 1118.5972 1 0 R.IVQQLDFEK.K**  ([Ions score 57](http://mbp-mascot4/mascot/cgi/peptide_view.pl?file=../data/20091023/F006344.dat&query=1437&hit=1&index=IPI00031411&px=1&section=5&ave_thresh=22))

**1084 - 1099 597.3022 1788.8849 1788.8854 -0 1 K.IGEETGVIETSDRLDR.E**  ([Ions score 24](http://mbp-mascot4/mascot/cgi/peptide_view.pl?file=../data/20091023/F006344.dat&query=3017&hit=1&index=IPI00031411&px=1&section=5&ave_thresh=22))

**1155 - 1173 1026.4742 2050.9339 2050.9331 0 0 K.DVSVVQIEAFDPDSSSNDK.L**  ([Ions score 88](http://mbp-mascot4/mascot/cgi/peptide_view.pl?file=../data/20091023/F006344.dat&query=3356&hit=1&index=IPI00031411&px=1&section=5&ave_thresh=22))

**1178 - 1192 815.4235 1628.8325 1628.8311 1 0 K.ITSGNPQGFFSIHPK.T**  ([Ions score 45](http://mbp-mascot4/mascot/cgi/peptide_view.pl?file=../data/20091023/F006344.dat&query=2844&hit=1&index=IPI00031411&px=1&section=5&ave_thresh=22))

**1235 - 1248 567.9653 1700.8742 1700.8733 0 0 K.ILDENDNKPQFLQK.F**  ([Ions score 26](http://mbp-mascot4/mascot/cgi/peptide_view.pl?file=../data/20091023/F006344.dat&query=2916&hit=1&index=IPI00031411&px=1&section=5&ave_thresh=22))

**1300 - 1305 390.7185 779.4224 779.4218 1 0 K.FFIEPK.T**  ([Ions score 22](http://mbp-mascot4/mascot/cgi/peptide_view.pl?file=../data/20091023/F006344.dat&query=80&hit=1&index=IPI00031411&px=1&section=5&ave_thresh=22))

**1314 - 1326 707.3667 1412.7188 1412.7187 0 0 R.FSAAGEYDILSIK.A**  ([Ions score 59](http://mbp-mascot4/mascot/cgi/peptide_view.pl?file=../data/20091023/F006344.dat&query=2484&hit=1&index=IPI00031411&px=1&section=5&ave_thresh=22))

**1407 - 1422 547.6454 1639.9143 1639.9145 -0 0 K.GTGTIIVAKPLDAEQK.S**  ([Ions score 22](http://mbp-mascot4/mascot/cgi/peptide_view.pl?file=../data/20091023/F006344.dat&query=2856&hit=1&index=IPI00031411&px=1&section=5&ave_thresh=22))

**1446 - 1460 582.2944 1743.8613 1743.8540 4 0 K.VIDTNDHRPQFSTSK.Y**  ([Ions score 34](http://mbp-mascot4/mascot/cgi/peptide_view.pl?file=../data/20091023/F006344.dat&query=2965&hit=1&index=IPI00031411&px=1&section=5&ave_thresh=22))

**1489 - 1497 540.8057 1079.5969 1079.5975 -1 0 K.LIYTLQSSR.D**  ([Ions score 45](http://mbp-mascot4/mascot/cgi/peptide_view.pl?file=../data/20091023/F006344.dat&query=1247&hit=1&index=IPI00031411&px=1&section=5&ave_thresh=22))

**1489 - 1497 540.8063 1079.5980 1079.5975 0 0 K.LIYTLQSSR.D**  ([Ions score 50](http://mbp-mascot4/mascot/cgi/peptide_view.pl?file=../data/20091023/F006344.dat&query=1248&hit=1&index=IPI00031411&px=1&section=5&ave_thresh=22))

**1507 - 1519 691.3458 1380.6770 1380.6773 -0 0 R.LDPATGSLYTSEK.L**  ([Ions score 60](http://mbp-mascot4/mascot/cgi/peptide_view.pl?file=../data/20091023/F006344.dat&query=2401&hit=1&index=IPI00031411&px=1&section=5&ave_thresh=22))

**1520 - 1535 472.2484 1884.9644 1884.9629 1 0 K.LDHEAVHQHTLTVMVR.D**  ([Ions score 26](http://mbp-mascot4/mascot/cgi/peptide_view.pl?file=../data/20091023/F006344.dat&query=3193&hit=1&index=IPI00031411&px=1&section=5&ave_thresh=22))

**1631 - 1641 649.3085 1296.6024 1296.6020 0 0 R.SNQAEYDLMVK.A**  ([Ions score 63](http://mbp-mascot4/mascot/cgi/peptide_view.pl?file=../data/20091023/F006344.dat&query=2156&hit=1&index=IPI00031411&px=1&section=5&ave_thresh=22))

**1631 - 1641 649.3086 1296.6027 1296.6020 1 0 R.SNQAEYDLMVK.A**  ([Ions score 42](http://mbp-mascot4/mascot/cgi/peptide_view.pl?file=../data/20091023/F006344.dat&query=2157&hit=1&index=IPI00031411&px=1&section=5&ave_thresh=22))

**1646 - 1657 630.8171 1259.6196 1259.6180 1 0 K.GSPPMSEITSVR.I**  ([Ions score 35](http://mbp-mascot4/mascot/cgi/peptide_view.pl?file=../data/20091023/F006344.dat&query=2003&hit=1&index=IPI00031411&px=1&section=5&ave_thresh=22))

**1646 - 1657 630.8171 1259.6196 1259.6180 1 0 K.GSPPMSEITSVR.I**  ([Ions score 28](http://mbp-mascot4/mascot/cgi/peptide_view.pl?file=../data/20091023/F006344.dat&query=2004&hit=1&index=IPI00031411&px=1&section=5&ave_thresh=22))

**1658 - 1669 638.3510 1274.6875 1274.6870 0 0 R.IFVTIADNASPK.F**  ([Ions score 82](http://mbp-mascot4/mascot/cgi/peptide_view.pl?file=../data/20091023/F006344.dat&query=2060&hit=1&index=IPI00031411&px=1&section=5&ave_thresh=22))

**1658 - 1669 638.3511 1274.6877 1274.6870 1 0 R.IFVTIADNASPK.F**  ([Ions score 36](http://mbp-mascot4/mascot/cgi/peptide_view.pl?file=../data/20091023/F006344.dat&query=2061&hit=1&index=IPI00031411&px=1&section=5&ave_thresh=22))

**1706 - 1726 734.3549 2200.0429 2200.0397 1 0 K.DGNTGDAFDINPHSGTIITQK.A**  ([Ions score 54](http://mbp-mascot4/mascot/cgi/peptide_view.pl?file=../data/20091023/F006344.dat&query=3469&hit=1&index=IPI00031411&px=1&section=5&ave_thresh=22))

**1962 - 1969 411.7403 821.4660 821.4647 2 0 R.FAGLTSVK.I**  ([Ions score 23](http://mbp-mascot4/mascot/cgi/peptide_view.pl?file=../data/20091023/F006344.dat&query=167&hit=1&index=IPI00031411&px=1&section=5&ave_thresh=22))

**1982 - 1992 628.8296 1255.6447 1255.6449 -0 0 K.FTQDVYSAVVK.E**  ([Ions score 80](http://mbp-mascot4/mascot/cgi/peptide_view.pl?file=../data/20091023/F006344.dat&query=1992&hit=1&index=IPI00031411&px=1&section=5&ave_thresh=22))

**1982 - 1992 628.8300 1255.6453 1255.6449 0 0 K.FTQDVYSAVVK.E**  ([Ions score 74](http://mbp-mascot4/mascot/cgi/peptide_view.pl?file=../data/20091023/F006344.dat&query=1993&hit=1&index=IPI00031411&px=1&section=5&ave_thresh=22))

**2031 - 2044 719.8623 1437.7100 1437.7100 0 0 R.TSGVLSTTGTPFDR.E**  ([Ions score 42](http://mbp-mascot4/mascot/cgi/peptide_view.pl?file=../data/20091023/F006344.dat&query=2550&hit=1&index=IPI00031411&px=1&section=5&ave_thresh=22))

**2031 - 2044 719.8624 1437.7102 1437.7100 0 0 R.TSGVLSTTGTPFDR.E**  ([Ions score 45](http://mbp-mascot4/mascot/cgi/peptide_view.pl?file=../data/20091023/F006344.dat&query=2551&hit=1&index=IPI00031411&px=1&section=5&ave_thresh=22))

**2143 - 2151 554.2879 1106.5612 1106.5608 0 0 K.QFELDTLNK.E**  ([Ions score 46](http://mbp-mascot4/mascot/cgi/peptide_view.pl?file=../data/20091023/F006344.dat&query=1377&hit=1&index=IPI00031411&px=1&section=5&ave_thresh=22))

**2440 - 2457 665.3661 1993.0764 1993.0745 1 0 K.HFVIDSATGIITLSNLHR.H**  ([Ions score 49](http://mbp-mascot4/mascot/cgi/peptide_view.pl?file=../data/20091023/F006344.dat&query=3291&hit=1&index=IPI00031411&px=1&section=5&ave_thresh=22))

**2549 - 2556 468.2638 934.5130 934.5124 1 0 R.GQIFTLEK.L**  ([Ions score 30](http://mbp-mascot4/mascot/cgi/peptide_view.pl?file=../data/20091023/F006344.dat&query=757&hit=1&index=IPI00031411&px=1&section=5&ave_thresh=22))

**2549 - 2556 468.2639 934.5133 934.5124 1 0 R.GQIFTLEK.L**  ([Ions score 23](http://mbp-mascot4/mascot/cgi/peptide_view.pl?file=../data/20091023/F006344.dat&query=758&hit=1&index=IPI00031411&px=1&section=5&ave_thresh=22))

**2604 - 2614 569.7907 1137.5669 1137.5666 0 0 K.YEVNIGSSAAK.G**  ([Ions score 56](http://mbp-mascot4/mascot/cgi/peptide_view.pl?file=../data/20091023/F006344.dat&query=1518&hit=1&index=IPI00031411&px=1&section=5&ave_thresh=22))

**2604 - 2614 569.7909 1137.5673 1137.5666 1 0 K.YEVNIGSSAAK.G**  ([Ions score 22](http://mbp-mascot4/mascot/cgi/peptide_view.pl?file=../data/20091023/F006344.dat&query=1519&hit=1&index=IPI00031411&px=1&section=5&ave_thresh=22))

**2687 - 2695 518.3080 1034.6015 1034.6012 0 0 K.ESVVLVYVK.I**  ([Ions score 33](http://mbp-mascot4/mascot/cgi/peptide_view.pl?file=../data/20091023/F006344.dat&query=1066&hit=1&index=IPI00031411&px=1&section=5&ave_thresh=22))

**2687 - 2695 518.3083 1034.6021 1034.6012 1 0 K.ESVVLVYVK.I**  ([Ions score 24](http://mbp-mascot4/mascot/cgi/peptide_view.pl?file=../data/20091023/F006344.dat&query=1067&hit=1&index=IPI00031411&px=1&section=5&ave_thresh=22))

**2730 - 2742 702.3799 1402.7452 1402.7456 -0 0 R.AEHSGTVLYSLVK.G**  ([Ions score 51](http://mbp-mascot4/mascot/cgi/peptide_view.pl?file=../data/20091023/F006344.dat&query=2458&hit=1&index=IPI00031411&px=1&section=5&ave_thresh=22))

**2993 - 3009 923.9580 1845.9015 1845.8996 1 0 R.DNYLLTITATDGTFSSK.A**  ([Ions score 88](http://mbp-mascot4/mascot/cgi/peptide_view.pl?file=../data/20091023/F006344.dat&query=3132&hit=1&index=IPI00031411&px=1&section=5&ave_thresh=22))

**3057 - 3072 827.4203 1652.8261 1652.8257 0 0 R.SNAEITYTLLGSGAEK.F**  ([Ions score 95](http://mbp-mascot4/mascot/cgi/peptide_view.pl?file=../data/20091023/F006344.dat&query=2874&hit=1&index=IPI00031411&px=1&section=5&ave_thresh=22))

**3084 - 3101 709.7121 2126.1144 2126.1120 1 1 K.TSTPLDREEQAVYHLLVR.A**  ([Ions score 43](http://mbp-mascot4/mascot/cgi/peptide_view.pl?file=../data/20091023/F006344.dat&query=3406&hit=1&index=IPI00031411&px=1&section=5&ave_thresh=22))

**3084 - 3101 709.7126 2126.1159 2126.1120 2 1 K.TSTPLDREEQAVYHLLVR.A**  ([Ions score 35](http://mbp-mascot4/mascot/cgi/peptide_view.pl?file=../data/20091023/F006344.dat&query=3407&hit=1&index=IPI00031411&px=1&section=5&ave_thresh=22))

**3193 - 3203 632.8620 1263.7094 1263.7074 2 0 R.ELQAVYTLSLK.A**  ([Ions score 26](http://mbp-mascot4/mascot/cgi/peptide_view.pl?file=../data/20091023/F006344.dat&query=2020&hit=1&index=IPI00031411&px=1&section=5&ave_thresh=22))

**3491 - 3507 887.4906 1772.9666 1772.9672 -0 0 K.AFEVNPQGVLLTSSAIK.R**  ([Ions score 68](http://mbp-mascot4/mascot/cgi/peptide_view.pl?file=../data/20091023/F006344.dat&query=2996&hit=1&index=IPI00031411&px=1&section=5&ave_thresh=22))

**3491 - 3507 591.9972 1772.9697 1772.9672 1 0 K.AFEVNPQGVLLTSSAIK.R**  ([Ions score 27](http://mbp-mascot4/mascot/cgi/peptide_view.pl?file=../data/20091023/F006344.dat&query=2997&hit=1&index=IPI00031411&px=1&section=5&ave_thresh=22))

**3733 - 3739 431.2638 860.5130 860.5120 1 0 R.ILNVFQK.L**  ([Ions score 24](http://mbp-mascot4/mascot/cgi/peptide_view.pl?file=../data/20091023/F006344.dat&query=339&hit=1&index=IPI00031411&px=1&section=5&ave_thresh=22))

**3740 - 3749 610.2836 1218.5527 1218.5526 0 0 K.LCAGLDCPWK.F**  ([Ions score 23](http://mbp-mascot4/mascot/cgi/peptide_view.pl?file=../data/20091023/F006344.dat&query=1876&hit=1&index=IPI00031411&px=1&section=5&ave_thresh=22))

**3755 - 3769 535.9227 1604.7462 1604.7464 -0 0 K.VSVDESVMSTHSTAR.L**  ([Ions score 27](http://mbp-mascot4/mascot/cgi/peptide_view.pl?file=../data/20091023/F006344.dat&query=2810&hit=1&index=IPI00031411&px=1&section=5&ave_thresh=22))

**3770 - 3776 410.2406 818.4666 818.4650 2 0 R.LSFVTPR.H**  ([Ions score 36](http://mbp-mascot4/mascot/cgi/peptide_view.pl?file=../data/20091023/F006344.dat&query=164&hit=1&index=IPI00031411&px=1&section=5&ave_thresh=22))

**3876 - 3888 499.9198 1496.7375 1496.7372 0 0 R.GTDYSILEIHHGR.L**  ([Ions score 28](http://mbp-mascot4/mascot/cgi/peptide_view.pl?file=../data/20091023/F006344.dat&query=2639&hit=1&index=IPI00031411&px=1&section=5&ave_thresh=22))

**3876 - 3888 375.1922 1496.7395 1496.7372 2 0 R.GTDYSILEIHHGR.L**  ([Ions score 24](http://mbp-mascot4/mascot/cgi/peptide_view.pl?file=../data/20091023/F006344.dat&query=2640&hit=1&index=IPI00031411&px=1&section=5&ave_thresh=22))

**3928 - 3945 603.3362 1806.9869 1806.9840 2 0 R.LVLDQVHTASGTAPGTLK.T**  ([Ions score 39](http://mbp-mascot4/mascot/cgi/peptide_view.pl?file=../data/20091023/F006344.dat&query=3051&hit=1&index=IPI00031411&px=1&section=5&ave_thresh=22))

**A818 A4**

Match to: **IPI00031411** Score: **263**

**Gene_Symbol=FAT1 Protocadherin Fat 1 lng=4591 # SP[4593,D,22,D]SNP[4646,g,1064,R]SNP[4677,r,1064,R]SNP[4713,i,1125,I]SNP[4791,l,1125,I]SNP[4844,i,1252,I]SNP[4856,h,1273,H]SNP[4889,r,1273,H]SNP[4931,l,1283,P]SNP[4969,p,1283,P]SNP[5000,l,129,V]SNP[5**

Found in search of C:\mgf\Orbidata\081031_ISW1295_A818_A4.mgf

Nominal mass (M_r_): **1068827**; Calculated pI value: **4.55**

NCBI BLAST search of [IPI00031411](http://www.ncbi.nlm.nih.gov/blast/Blast.cgi?ALIGNMENTS=50&ALIGNMENT_VIEW=Pairwise&AUTO_FORMAT=Semiauto&CDD_SEARCH=on&CLIENT=web&COMPOSITION_BASED_STATISTICS=on&DATABASE=nr&DESCRIPTIONS=100&ENTREZ_QUERY=(none)&EXPECT=10&FILTER=L&FORMAT_BLOCK_ON_RESPAGE=None&FORMAT_OBJECT=Alignment&FORMAT_TYPE=HTML&GAPCOSTS=11+1&I_THRESH=0.001&LAYOUT=TwoWindows&MATRIX_NAME=BLOSUM62&NCBI_GI=on&PAGE=Proteins&PROGRAM=blastp&QUERY=IPI00031411&SERVICE=plain&SET_DEFAULTS.x=21&SET_DEFAULTS.y=7&SHOW_OVERVIEW=on&WORD_SIZE=3&END_OF_HTTPGET=Yes) against nr

Unformatted [sequence string](http://mbp-mascot4/mascot/cgi/getseq.pl?IPI_human+IPI00031411+seq) for pasting into other applications

Fixed modifications: Carbamidomethyl (C)

Variable modifications: Oxidation (M)

Cleavage by TrypsinMSIPI, a mixture of enzymes:

cuts C-term side of KR unless next residue is P

cuts C-term side of J

cuts N-term side of J

Sequence Coverage: **1%**

Matched peptides shown in **Bold Red**

**1** MGRHLALLLL LLLLFQHFGD SDGSQRLEQT PLQFTHLEYN VTVQENSAAK

**51** TYVGHPVKMG VYITHPAWEV RYKIVSGDSE NLFKAEEYIL GDFCFLRIRT

**101** KGGNTAILNR EVKDHYTLIV KALEKNTNVE ARTKVRVQVL DTNDLRPLFS

**151** PTSYSVSLPE NTAIRTSIAR VSATDADIGT NGEFYYSFKD RTDMFAIHPT

**201** SGVIVLTGRL DYLETKLYEM EILAADRGMK LYGSSGISSM AKLTVHIEQA

**251** NECAPVITAV TLSPSELDRD PAYAIVTVDD CDQGANGDIA SLSIVAGDLL

**301** QQFRTVRSFP GSKEYKVKAI GGIDWDSHPF GYNLTLQAKD KGTPPQFSSV

**351** KVIHVTSPQF KAGPVKFEKD VYRAEISEFA PPNTPVVMVK AIPAYSHLRY

**401** VFKSTPGKAK FSLNYNTGLI SILEPVKRQQ AAHFELEVTT SDRKASTKVL

**451** VK**VLGANSNP PEFTQTAYK**A AFDENVPIGT TVMSLSAVDP DEGENGYVTY

**501** SIANLNHVPF AIDHFTGAVS TSENLDYELM PRVYTLRIR**A SDWGLPYR**RE

**551** VEVLATITLN NLNDNTPLFE KINCEGTIPR DLGVGEQITT VSAIDADELQ

**601** LVQYQIEAGN ELDFFSLNPN SGVLSLKRSL MDGLGAKVSF HSLRITATDG

**651** ENFATPLYIN ITVAASHKLV NLQCEETGVA KMLAEKLLQA NKLHNQGEVE

**701** DIFFDSHSVN AHIPQFRSTL PTGIQVKENQ PVGSSVIFMN STDLDTGFNG

**751** KLVYAVSGGN EDSCFMIDME TGMLKILSPL DRETTDKYTL NITVYDLGIP

**801** QKAAWRLLHV VVVDANDNPP EFLQESYFVE VSEDKEVHSE IIQVEATDKD

**851** LGPNGHVTYS IVTDTDTFSI DSVTGVVNIA RPLDRELQHE HSLKIEARDQ

**901** AREEPQLFST VVVKVSLEDV NDNPPTFIPP NYRVKVREDL PEGTVIMWLE

**951** AHDPDLGQSG QVRYSLLDHG EGNFDVDKLS GAVRIVQQLD FEKKQVYNLT

**1001** VRAKDKGKPV SLSSTCYVEV EVVDVNENLH PPVFSSFVEK GTVKEDAPVG

**1051** SLVMTVSAHD EDARRDGEIR YSIRDGSGVG VFKIGEETGV IETSDRLDRE

**1101** STSHYWLTVF ATDQGVVPLS SFIEIYIEVE DVNDNAPQTS EPVYYPEIME

**1151** NSPKDVSVVQ IEAFDPDSSS NDKLMYKITS GNPQGFFSIH PKTGLITTTS

**1201** RKLDREQQDE HILEVTVTDN GSPPKSTIAR VIVKILDEND NKPQFLQKFY

**1251** KIRLPEREKP DRERNARREP LYHVIATDKD EGPNAEISYS IEDGNEHGK**F**

**1301 FIEPK**TGVVS SKRFSAAGEY DILSIKAVDN GRPQKSSTTR LHIEWISKPK

**1351** PSLEPISFEE SFFTFTVMES DPVAHMIGVI SVEPPGIPLW FDITGGNYDS

**1401** HFDVDK**GTGT IIVAKPLDAE QK**SNYNLTVE ATDGTTTILT QVFIKVIDTN

**1451** DHRPQFSTSK YEVVIPEDTA PETEILQISA VDQDEKNKLI YTLQSSRDPL

**1501** SLKKFRLDPA TGSLYTSEKL DHEAVHQHTL TVMVRDQDVP VKRNFARIVV

**1551** NVSDTNDHAP WFTASSYKGR VYESAAVGSV VLQVTALDKD KGKNAEVLYS

**1601** IESGTFGNIG NSFMIDPVLG SIKTAKELDR **SNQAEYDLMV K**ATDKGSPPM

**1651** SEITSVR**IFV TIADNASPK**F TSKEYSVELS ETVSIGSFVG MVTAHSQSSV

**1701** VYEIKDGNTG DAFDINPHSG TIITQKALDF ETLPIYTLII QGTNMAGLST

**1751** NTTVLVHLQD ENDNAPVFMQ AEYTGLISES ASINSVVLTD RNVPLVIRAA

**1801** DADKDSNALL VYHIVEPSVH TYFAIDSSTG AIHTVLSLDY EETSIFHFTV

**1851** QVHDMGTPRL FAEYAANVTV HVIDINDCPP VFAKPLYEAS LLLPTYKGVK

**1901** VITVNATDAD SSAFSQLIYS ITEGNIGEKF SMDYKTGALT VQNTTQLRSR

**1951** YELTVRASDG RFAGLTSVKI NVKESKESHL K**FTQDVYSAV VK**ENSTEAET

**2001** LAVITAIGNP INEPLFYHIL NPDRRFKISR **TSGVLSTTGT PFDR**EQQEAF

**2051** DVVVEVTEEH KPSAVAHVVV KVIVEDQNDN APVFVNLPYY AVVKVDTEVG

**2101** HVIRYVTAVD RDSGRNGEVH YYLKEHHEHF QIGPLGEISL KK**QFELDTLN**

**2151 K**EYLVTVVAK DGGNPAFSAE VIVPITVMNK AMPVFEKPFY SAEIAESIQV

**2201** HSPVVHVQAN SPEGLKVFYS ITDGDPFSQF TINFNTGVIN VIAPLDFEAH

**2251** PAYKLSIRAT DSLTGAHAEV FVDIIVDDIN DNPPVFAQQS YAVTLSEASV

**2301** IGTSVVQVRA TDSDSEPNRG ISYQMFGNHS KSHDHFHVDS STGLISLLRT

**2351** LDYEQSRQHT IFVRAVDGGM PTLSSDVIVT VDVTDLNDNP PLFEQQIYEA

**2401** RISEHAPHGH FVTCVKAYDA DSSDIDKLQY SILSGNDHK**H FVIDSATGII**

**2451 TLSNLHR**HAL KPFYSLNLSV SDGVFRSSTQ VHVTVIGGNL HSPAFLQNEY

**2501** EVELAENAPL HTLVMEVKTT DGDSGIYGHV TYHIVNDFAK DRFYINERGQ

**2551** IFTLEKLDRE TPAEKVISVR LMAKDAGGKV AFCTVNVILT DDNDNAPQFR

**2601** ATK**YEVNIGS SAAK**GTSVVK VLASDADEGS NADITYAIEA DSESVKENLE

**2651** INKLSGVITT KESLIGLENE FFTFFVRAVD NGSPSK**ESVV LVYVK**ILPPE

**2701** MQLPKFSEPF YTFTVSEDVP IGTEIDLIRA EHSGTVLYSL VKGNTPESNR

**2751** DESFVIDRQS GRLKLEKSLD HETTKWYQFS ILARCTQDDH EMVASVDVSI

**2801** QVKDANDNSP VFESSPYEAF IVENLPGGSR VIQIRASDAD SGTNGQVMYS

**2851** LDQSQSVEVI ESFAINMETG WITTLKELDH EKRDNYQIKV VASDHGEKIQ

**2901** LSSTAIVDVT VTDVNDSPPR FTAEIYKGTV SEDDPQGGVI AILSTTDADS

**2951** EEINRQVTYF ITGGDPLGQF AVETIQNEWK VYVKKPLDRE KRDNYLLTIT

**3001** ATDGTFSSKA IVEVKVLDAN DNSPVCEKTL YSDTIPEDVL PGKLIMQISA

**3051** TDADIR**SNAE ITYTLLGSGA EK**FKLNPDTG ELKTSTPLDR EEQAVYHLLV

**3101** RATDGGGRFC QASIVLTLED VNDNAPEFSA DPYAITVFEN TEPGTLLTRV

**3151** QATDADAGLN RKILYSLIDS ADGQFSINEL SGIIQLEKPL DRELQAVYTL

**3201** SLKAVDQGLP RRLTATGTVI VSVLDINDNP PVFEYREYGA TVSEDILVGT

**3251** EVLQVYAASR DIEANAEITY SIISGNEHGK FSIDSKTGAV FIIENLDYES

**3301** SHEYYLTVEA TDGGTPSLSD VATVNVNVTD INDNTPVFSQ DTYTTVISED

**3351** AVLEQSVITV MADDADGPSN SHIHYSIIDG NQGSSFTIDP VRGEVKVTKL

**3401** LDRETISGYT LTVQASDNGS PPRVNTTTVN IDVSDVNDNA PVFSRGNYSV

**3451** IIQENKPVGF SVLQLVVTDE DSSHNGPPFF FTIVTGNDEK AFEVNPQGVL

**3501** LTSSAIKRKE KDHYLLQVKV ADNGKPQLSS LTYIDIRVIE ESIYPPAILP

**3551** LEIFITSSGE EYSGGVIGKI HATDQDVYDT LTYSLDPQMD NLFSVSSTGG

**3601** KLIAHKKLDI GQYLLNVSVT DGKFTTVADI TVHIRQVTQE MLNHTIAIRF

**3651** ANLTPEEFVG DYWRNFQRAL RNILGVRRND IQIVSLQSSE PHPHLDVLLF

**3701** VEKPGSAQIS TKQLLHKINS SVTDIEEIIG VRILNVFQKL CAGLDCPWKF

**3751** CDEKVSVDES VMSTHSTARL SFVTPRHHRA AVCLCKEGRC PPVHHGCEDD

**3801** PCPEGSECVS DPWEEKHTCV CPSGRFGQCP GSSSMTLTGN SYVKYRLTEN

**3851** ENKLEMKLTM RLRTYSTHAV VMYARGTDYS ILEIHHGRLQ YKFDCGSGPG

**3901** IVSVQSIQVN DGQWHAVALE VNGNYARLVL DQVHTASGTA PGTLKTLNLD

**3951** NYVFFGGHIR QQGTRHGRSP QVGNGFRGCM DSIYLNGQEL PLNSKPRSYA

**4001** HIEESVDVSP GCFLTATEDC ASNPCQNGGV CNPSPAGGYY CKCSALYIGT

**4051** HCEISVNPCS SKPCLYGGTC VVDNGGFVCQ CRGLYTGQRC QLSPYCKDEP

**4101** CKNGGTCFDS LDGAVCQCDS GFRGERCQSD IDECSGNPCL HGALCENTHG

**4151** SYHCNCSHEY RGRHCEDAAP NQYVSTPWNI GLAEGIGIVV FVAGIFLLVV

**4201** VFVLCRKMIS RKKKHQAEPK DKHLGPATAF LQRPYFDSKL NKNIYSDIPP

**4251** QVPVRPISYT PSIPSDSRNN LDRNSFEGSA IPEHPEFSTF NPESVHGHRK

**4301** AVAVCSVAPN LPPPPPSNSP SDSDSIQKPS WDFDYDTKVV DLDPCLSKKP

**4351** LEEKPSQPYS ARESLSEVQS LSSFQSESCD DNGYHWDTSD WMPSVPLPDI

**4401** QEFPNYEVID EQTPLYSADP NAIDTDYYPG GYDIESDFPP PPEDFPAADE

**4451** LPPLPPEFSN QFESIHPPRD MPAAGSLGSS SRNRQRFNLN QYLPNFYPLD

**4501** MSEPQTKGTG ENSTCREPHA PYPPGYQRHF EAPAVESMPM SVYASTASCS

**4551** DVSACCEVES EVMMSDYESG DDGHFEEVTI PPLDSQQHTE VJDGSQRLEQ

**4601** TPLQFTHLEY NVTVQENSAA KJGTVKEDAP VGSLVMTVSA HDEDAGRDGE

**4651** IRJGTVKEDA PVGSLVMTVS AHDEDARRDG EIRJLDREST SHYWLTVFAT

**4701** DQGVVPLSSF IEIYIEVEDV NDNAPQTSEP VYYPEIMENS PKDVSVVQIE

**4751** AFDPDSSSND KJLDRESTSH YWLTVFATDQ GVVPLSSFIE LYIEVEDVND

**4801** NAPQTSEPVY YPEIMENSPK DVSVVQIEAF DPDSSSNDKJ FYKIRLPERJ

**4851** REPLYHVIAT DKDEGPNAEI SYSIEDGNEH GKJREPLYRV IATDKDEGPN

**4901** AEISYSIEDG NEHGKJEPLY HVIATDKDEG LNAEISYSIE DGNEHGKFFI

**4951** EPKJEPLYHV IATDKDEGPN AEISYSIEDG NEHGKFFIEP KJALEKNTNL

**5001** EARTKJALEK NTNVEARTKJ ALEKNTNVEA RTKJALEKNT NVEVRTKJFS

**5051** AAGEYDILSI KAVDNGRPQK SSTTRJFSAA GEYDILSIKA VDSGRPQKSS

**5101** TTRJNFARIV VNVSDTNDHA PWFTASSYKG RJNFARIVVN VSDTNDHAPW

**5151** FTTSSYKGRJ GKNAEVLYSI ESGDFGNIGN SFMIDPVLGS IKTAKJGKNA

**5201** EVLYSIESGN FGNIGNSFMI DPVLGSIKTA KJGKNAEVLY SIESGTIGNI

**5251** GNSFMIDPVL GSIKTAKJGS PPMSEITSVR IFVTNADNAS PKFTSKJGSP

**5301** PMSEITSVRI FVTSADNASP KFTSKJGSPP MSEITSVRIF VTIADNASTK

**5351** FTSKJALDFE TLPIYTLIIQ GTNMAGLSTN TTVLVHLQDE NDNAPVFMQA

**5401** EYTGLISESA SINSVVLTDR NVPQVIRAAD ADKJALDFET LPIYTLIIQG

**5451** TNMAGLSTNT TVLVHLQDEN DNAPVFMQAE YTGLISESAS INSVVLTDRN

**5501** VPRVIRAADA DKJAADADKD SNVLLVYHIV EPSVHTYFAI DSSTGAIHTV

**5551** LSLDYEETSI FHFTVQVHDM GTPRLFAEYA ANVTVHVIDI NDCPPVFAKP

**5601** LYEASLLLPT YKJFTQDVYS AVVKTNSTEA ETLAVITAIG NPINEPLFYH

**5651** ILNPDRRJTS GVLSTTGTPF DREAQEAFDV VVEVTEEHKP SAVAHVVVKV

**5701** IVEDQNDNAP VFVNLPYYAV VKJTDMFAIH PTSGVIVLTG RLDFLETKLY

**5751** EMEILAADRJ TDMFAIHPTS GVIVLTGRLD YLETKLYEME ILAADRJNGE

**5801** VHYYLKEHHE HFQIGPLGEK SLKKJDGGNP AFSAEVIVPI TVMNKAMPVF

**5851** EKPFYSAEIA ESIQVHSHVV HVQANSPEGL KVFYSITDGD PFSQFTINFN

**5901** TGVINVIAPL DFEAHPAYKJ AMPVFEKPFY SAEIAESIQV HSPVVHVQAN

**5951** SPEGLKVFYS ITDGDPFSQF TINFNTGVIN VIAPLDFPAH PAYKLSIRJL

**6001** SIRATDSLTG AHAEVFVDDI VDDINDNPPV FAQQSYAVTL SEASVIGTSV

**6051** VQVRATDSDS EPNRJLSIRA TDSLTGAHAE VFVDEIVDDI NDNPPVFAQQ

**6101** SYAVTLSEAS VIGTSVVQVR ATDSDSEPNR JTLDYEQSRQ HTIAVRAVDG

**6151** GMPTLSSDVI VTVDVTDLND NPPLFEQQIY EARJLYGSSG ISSMAKLTVH

**6201** IEQANECAPV ITAVTLSPSE LDRDPAYAIV TVDDCDQGAN GDIASLSIVA

**6251** GDLLQQFRJI LPPEMQLPKF SEPFYTFTVS EIVPIGTEID LIRAEHSGTV

**6301** LYSLVKJILP PEMQLPKFSE PFYTFTVSEV VPIGTEIDLI RAEHSGTVLY

**6351** SLVKJCTQDD HEMVASVDVS IQVKDANDNS PVFESSPYEA FIVENLPGGS

**6401** RVIQIRJCTQ DDHEMVASVD VSIQVKDASD NSPVFESSPY EAFIVENLPG

**6451** GSRVIQIRJV IQIRASDADN GTNGQVMYSL DQSQSVEVIE SFAINMETGW

**6501** ITTLKELDHE KJFTAEIYKG TVSELDPQGG VIAILSTTDA DSEEINRQVT

**6551** YFITGGDPLG QFAVETIQNE WKJFTAEIYK GTVSEPDPQG GVIAILSTTD

**6601** ADSEEINRQV TYFITGGDPL GQFAVETIQN EWKJFTAEIY KGTVSEQDPQ

**6651** GGVIAILSTT DADSEEINRQ VTYFITGGDP LGQFAVETIQ NEWKJGTVSE

**6701** DDPQGGVIAI LSTTDADSEE INRQVTYGIT GGDPLGQFAV ETIQNEWKVY

**6751** VKJGTVSEDD PQGGVIAILS TTDADSEEIN RQVTYSITGG DPLGQFAVET

**6801** IQNEWKVYVK JGTVSEDDPQ GGVIAILSTT DADSEEINRQ VTYFITGGDP

**6851** LAQFAVETIQ NEWKVYVKJA IVEVKVLDAN DNSPVCEYTL YSDTIPEDVL

**6901** PGKLIMQISA TDADIRJVLD ANDNSPVCEK TLYIDTIPED VLPGKLIMQI

**6951** SATDADIRJV LDANDNSPVC EKTLYTDTIP EDVLPGKLIM QISATDADIR

**7001** JATDGGGRFC QASIVLDLED VNDNAPEFSA DPYAITVFEN TEPGTLLTRV

**7051** QATDADAGLN RJKILYSLID SADGQFSINE LIGIIQLEKP LDRELQAVYT

**7101** LSLKJFSIDS KTGAVFIIEN LDYESSHEYY LTVEATDGGT PSLSDVATVN

**7151** VNVTDINDAT PVFSQDTYTT VISEDAVLEQ SVITVMADDA DGPSNSHIHY

**7201** SIIDGNQGSS FTIDPVRGEV KJFSIDSKTG AVFIIENLDY ESSHEYYLTV

**7251** EATDGGTPSL SDVATVNVNV TDINDVTPVF SQDTYTTVIS EDAVLEQSVI

**7301** TVMADDADGP SNSHIHYSII DGNQGSSFTI DPVRGEVKJV NTTTVNIDVS

**7351** DVNDNAPVFS RGNYSVIIQE NKPVGFSVLQ LVVTDEDSSH NGPPFFFTIV

**7401** TENDEKAFEV NPQGVLLTSS AIKJVNTTTV NIDVSDVNDN APVFSRGNYS

**7451** VIIQENKPVG FSVLQLVVTD EDSSHNGPPF FFTIVTGNDE KAFEVNPQGV

**7501** LLTSSAIKJG NYSVIIQENK PVGFSVLQLV VTDEDSSHNG PPFFFTIVTG

**7551** NDEKAFEVNA QGVLLTSSAI KRJGNYSVII QENKPVGFSV LQLVVTDEDS

**7601** SHNGPPFFFT IVTGNDEKAF EVNVQGVLLT SSAIKRJVAD NGKPQLSSLT

**7651** YIDIRVIEES IYPPAILPLE IAITSSGEEY SGGVIGKIHA TDQDVYDTLT

**7701** YSLDPQMDNL FSVSSTGGKJ VADNGKPQLS SLTYIDIRVI EESIYPPAIL

**7751** PLEISITSSG EEYSGGVIGK IHATDQDVYD TLTYSLDPQM DNLFSVSSTG

**7801** GKJVIHVTSP QFKAGPVKFE KJQLLHKIMS SVTDIEEIIG VRILNVFQKJ

**7851** QLLHKIVSSV TDIEEIIGVR ILNVFQKJEG RCPPVHHGCE DHPCPEGSEC

**7901** VSDPWEEKHT CVCPSGRJEG RCPPVHHGCE DPPCPEGSEC VSDPWEEKHT

**7951** CVCPSGRJHT CVCPSGRFGG CPGSSSMTLT GNSYVKYRJH TCVCPSGRFG

**8001** RCPGSSSMTL TGNSYVKYRJ FGQCPGSSSM TLTGNSYVKT RLTENENKJG

**8051** CMDSIYLNGQ ELPLNSKPRS YAHIEESVPV SPGCFLTATE DCASNPCQNG

**8101** GVCNPSPAGG YYCKCSALYI GTHCEISVNP CSSKPCLYGG TCVVDNGGFV

**8151** CQCRJYVFKR TPGKAKJYVF KSTPGKAKJS YAHIEESVDV SPGCFLTATE

**8201** DCASNPCQNG GVCNPSPAGG YYCKCSALYI GTHCEISVNP KSSKPCLYGG

**8251** TCVVDNGGFV CQCRGLYTGQ RJSYAHIEES VDVSPGCFLT ATEDCASNPC

**8301** QNGGVCNPSP AGGYYCKCSA LYIGTHCEIS VNPNSSKPCL YGGTCVVDNG

**8351** GFVCQCRGLY TGQRJDEPCK NGGTCFDSAD GAVCQCDSGF RGERJGRHCA

**8401** DAAPNQYVST PWNIGLAEGI GIVVFVAGIF LLVVVFVLCR KJGRHCEDAA

**8451** PNQYVSTPWN IGLAEGIGIV VFVAGIFLLV VVFVLCRKJN IYSDIPPQVP

**8501** VRYISYTPSI PSDSRNNLDR JKAVAVCSVA PNLPPPPPSN SPSDSDSIQK

**8551** PSWDFDYDTK VVDLDPCLSK JKPLEEKPSQ PYSARESLSS VQSLSSFQSE

**8601** SCDDNGYHWD TSDWMPSVPL PDIQEFPNYE VIDEQTPLYS ADPNAIDTDY

**8651** YPGGYDIESD FPPPPEDFPA ADELPPLPPE FSNQFESIHP PRDMPAAGSL

**8701** GSSSRJKPLE EKPSQPYSAR ESLSEVQSLS SFQSESCDDN GYHWDTSDWM

**8751** PSVPLQDIQE FPNYEVIDEQ TPLYSADPNA IDTDYYPGGY DIESDFPPPP

**8801** EDFPAADELP PLPPEFSNQF ESIHPPRDMP AAGSLGSSSR JKPLEEKPSQ

**8851** PYSARESLSE VQSLSSFQSE SCDDNGYHWD TSDWMPSVPL PDIQEFPNYE

**8901** VIDPQTPLYS ADPNAIDTDY YPGGYDIESD FPPPPEDFPA ADELPPLPPE

**8951** FSNQFESIHP PRDMPAAGSL GSSSRJESLS EVQSLSSFQS ESCDDNGYHW

**9001** DTSDWMPSVP LPDIQEFPNY EVIDEQTPLY SADPNAIDTD YYPGGYDIES

**9051** DFPPPPEDFP AADELPPLPP EFSNQFESIH PPRDMPAAGS LGSSRRNRJE

**9101** SLSEVQSLSS FQSESCDDNG YHWDTSDWMP SVPLPDIQEF PNYEVIDEQT

**9151** PLYSADPNAI DTDYYPGGYD IESDFPPPPE DFPAADELPP LPPEFSNQFE

**9201** SIHPPRDMPA AGSLGSSWRN RJVLGANSNP PEFTQTAYKA AFDENVPIGT

**9251** TIMSLSAVDP DEGENGYVTY SIANLNHVPF AIDHFTGAVS TSENLDYELM

**9301** PRVYTLRJVL GANSNPPEFT QTAYKAAFDE NVPIGTTVMS LSAVDPDEGE

**9351** NGYVTYSIAN LNHVPFAIDH FTGAVSTSEN LDYELMPRVY TLRJVLGANS

**9401** NPPEFTQTAY KAAFDENVPI GTTVMSLSAV DPDEGENGYV TYSIANLNHV

**9451** PFAIDHFTGA VSTSENLDYE LMPRVYTLRJ INCEGTIPRD LGVGEQITTV

**9501** SAIDADELQL VQYQIEAGNE LDFFSLNPNS GVLSLKRJIN CEGTIPRDLG

**9551** VGEQITTVSA IDADELQLVQ YQIEAGNELD LFSLNPNSGV LSLKRJINCE

**9601** GTIPRDLGVG EQITTVSAID ADELQLVQYQ IEAGNELDFF SLNPNSGVLS

**9651** LKRJEVHSEI IQVEATDKDL GPNGHVTYSI LTDTDTFSID SVTGVVNIAR

**9701** PLDRELQHEH SLKJEVHSEI IQVEATDKDL GPNGHVTYSI VTDTDTFSID

**9751** SVTGVVNIAR PLDRELQHEH SLK

**Start - End Observed Mr(expt) Mr(calc) ppm Miss Sequence**

**453 - 469 918.9597 1835.9049 1835.9054 -0 0 K.VLGANSNPPEFTQTAYK.A**  ([Ions score 29](http://mbp-mascot4/mascot/cgi/peptide_view.pl?file=../data/20091023/F006340.dat&query=3315&hit=1&index=IPI00031411&px=1&section=5&ave_thresh=22))

**540 - 548 532.7609 1063.5073 1063.5087 -1 0 R.ASDWGLPYR.R**  ([Ions score 36](http://mbp-mascot4/mascot/cgi/peptide_view.pl?file=../data/20091023/F006340.dat&query=1249&hit=1&index=IPI00031411&px=1&section=5&ave_thresh=22))

**1300 - 1305 390.7188 779.4230 779.4218 2 0 K.FFIEPK.T**  ([Ions score 26](http://mbp-mascot4/mascot/cgi/peptide_view.pl?file=../data/20091023/F006340.dat&query=90&hit=1&index=IPI00031411&px=1&section=5&ave_thresh=22))

**1407 - 1422 547.6462 1639.9167 1639.9145 1 0 K.GTGTIIVAKPLDAEQK.S**  ([Ions score 24](http://mbp-mascot4/mascot/cgi/peptide_view.pl?file=../data/20091023/F006340.dat&query=3055&hit=1&index=IPI00031411&px=1&section=5&ave_thresh=22))

**1631 - 1641 649.3079 1296.6012 1296.6020 -1 0 R.SNQAEYDLMVK.A**  ([Ions score 45](http://mbp-mascot4/mascot/cgi/peptide_view.pl?file=../data/20091023/F006340.dat&query=2224&hit=1&index=IPI00031411&px=1&section=5&ave_thresh=22))

**1658 - 1669 638.3503 1274.6861 1274.6870 -1 0 R.IFVTIADNASPK.F**  ([Ions score 35](http://mbp-mascot4/mascot/cgi/peptide_view.pl?file=../data/20091023/F006340.dat&query=2133&hit=1&index=IPI00031411&px=1&section=5&ave_thresh=22))

**1658 - 1669 638.3511 1274.6877 1274.6870 1 0 R.IFVTIADNASPK.F**  ([Ions score 44](http://mbp-mascot4/mascot/cgi/peptide_view.pl?file=../data/20091023/F006340.dat&query=2134&hit=1&index=IPI00031411&px=1&section=5&ave_thresh=22))

**1982 - 1992 628.8296 1255.6446 1255.6449 -0 0 K.FTQDVYSAVVK.E**  ([Ions score 58](http://mbp-mascot4/mascot/cgi/peptide_view.pl?file=../data/20091023/F006340.dat&query=2044&hit=1&index=IPI00031411&px=1&section=5&ave_thresh=22))

**2031 - 2044 719.8624 1437.7102 1437.7100 0 0 R.TSGVLSTTGTPFDR.E**  ([Ions score 47](http://mbp-mascot4/mascot/cgi/peptide_view.pl?file=../data/20091023/F006340.dat&query=2679&hit=1&index=IPI00031411&px=1&section=5&ave_thresh=22))

**2143 - 2151 554.2880 1106.5614 1106.5608 1 0 K.QFELDTLNK.E**  ([Ions score 24](http://mbp-mascot4/mascot/cgi/peptide_view.pl?file=../data/20091023/F006340.dat&query=1458&hit=1&index=IPI00031411&px=1&section=5&ave_thresh=22))

**2440 - 2457 665.3666 1993.0779 1993.0745 2 0 K.HFVIDSATGIITLSNLHR.H**  ([Ions score 52](http://mbp-mascot4/mascot/cgi/peptide_view.pl?file=../data/20091023/F006340.dat&query=3477&hit=1&index=IPI00031411&px=1&section=5&ave_thresh=22))

**2604 - 2614 569.7904 1137.5662 1137.5666 -0 0 K.YEVNIGSSAAK.G**  ([Ions score 31](http://mbp-mascot4/mascot/cgi/peptide_view.pl?file=../data/20091023/F006340.dat&query=1578&hit=1&index=IPI00031411&px=1&section=5&ave_thresh=22))

**2687 - 2695 518.3082 1034.6019 1034.6012 1 0 K.ESVVLVYVK.I**  ([Ions score 26](http://mbp-mascot4/mascot/cgi/peptide_view.pl?file=../data/20091023/F006340.dat&query=1138&hit=1&index=IPI00031411&px=1&section=5&ave_thresh=22))

**3057 - 3072 827.4200 1652.8255 1652.8257 -0 0 R.SNAEITYTLLGSGAEK.F**  ([Ions score 54](http://mbp-mascot4/mascot/cgi/peptide_view.pl?file=../data/20091023/F006340.dat&query=3072&hit=1&index=IPI00031411&px=1&section=5&ave_thresh=22))

**BxPc3 A1:**

Match to: **IPI00031411** Score: **262**

**Gene_Symbol=FAT1 Protocadherin Fat 1 lng=4591 # SP[4593,D,22,D]SNP[4646,g,1064,R]SNP[4677,r,1064,R]SNP[4713,i,1125,I]SNP[4791,l,1125,I]SNP[4844,i,1252,I]SNP[4856,h,1273,H]SNP[4889,r,1273,H]SNP[4931,l,1283,P]SNP[4969,p,1283,P]SNP[5000,l,129,V]SNP[5**

Found in search of C:\mgf\Orbidata\081107_ISW1295_BXPC3_A1.mgf

Nominal mass (M_r_): **1068827**; Calculated pI value: **4.55**

NCBI BLAST search of [IPI00031411](http://www.ncbi.nlm.nih.gov/blast/Blast.cgi?ALIGNMENTS=50&ALIGNMENT_VIEW=Pairwise&AUTO_FORMAT=Semiauto&CDD_SEARCH=on&CLIENT=web&COMPOSITION_BASED_STATISTICS=on&DATABASE=nr&DESCRIPTIONS=100&ENTREZ_QUERY=(none)&EXPECT=10&FILTER=L&FORMAT_BLOCK_ON_RESPAGE=None&FORMAT_OBJECT=Alignment&FORMAT_TYPE=HTML&GAPCOSTS=11+1&I_THRESH=0.001&LAYOUT=TwoWindows&MATRIX_NAME=BLOSUM62&NCBI_GI=on&PAGE=Proteins&PROGRAM=blastp&QUERY=IPI00031411&SERVICE=plain&SET_DEFAULTS.x=21&SET_DEFAULTS.y=7&SHOW_OVERVIEW=on&WORD_SIZE=3&END_OF_HTTPGET=Yes) against nr

Unformatted [sequence string](http://mbp-mascot4/mascot/cgi/getseq.pl?IPI_human+IPI00031411+seq) for pasting into other applications

Fixed modifications: Carbamidomethyl (C)

Variable modifications: Oxidation (M)

Cleavage by TrypsinMSIPI, a mixture of enzymes:

cuts C-term side of KR unless next residue is P

cuts C-term side of J

cuts N-term side of J

Sequence Coverage: **1%**

Matched peptides shown in **Bold Red**

**1** MGRHLALLLL LLLLFQHFGD SDGSQRLEQT PLQFTHLEYN VTVQENSAAK

**51** TYVGHPVKMG VYITHPAWEV RYKIVSGDSE NLFKAEEYIL GDFCFLRIRT

**101** KGGNTAILNR EVKDHYTLIV KALEKNTNVE ARTKVRVQVL DTNDLRPLFS

**151** PTSYSVSLPE NTAIRTSIAR VSATDADIGT NGEFYYSFKD R**TDMFAIHPT**

**201 SGVIVLTGR**L DYLETKLYEM EILAADRGMK LYGSSGISSM AKLTVHIEQA

**251** NECAPVITAV TLSPSELDRD PAYAIVTVDD CDQGANGDIA SLSIVAGDLL

**301** QQFRTVRSFP GSKEYKVKAI GGIDWDSHPF GYNLTLQAKD KGTPPQFSSV

**351** KVIHVTSPQF KAGPVKFEKD VYRAEISEFA PPNTPVVMVK AIPAYSHLRY

**401** VFKSTPGKAK FSLNYNTGLI SILEPVKRQQ AAHFELEVTT SDRKASTKVL

**451** VK**VLGANSNP PEFTQTAYK**A AFDENVPIGT TVMSLSAVDP DEGENGYVTY

**501** SIANLNHVPF AIDHFTGAVS TSENLDYELM PRVYTLRIRA SDWGLPYRRE

**551** VEVLATITLN NLNDNTPLFE KINCEGTIPR DLGVGEQITT VSAIDADELQ

**601** LVQYQIEAGN ELDFFSLNPN SGVLSLKRSL MDGLGAKVSF HSLRITATDG

**651** ENFATPLYIN ITVAASHKLV NLQCEETGVA KMLAEKLLQA NKLHNQGEVE

**701** DIFFDSHSVN AHIPQFRSTL PTGIQVKENQ PVGSSVIFMN STDLDTGFNG

**751** KLVYAVSGGN EDSCFMIDME TGMLKILSPL DRETTDKYTL NITVYDLGIP

**801** QKAAWRLLHV VVVDANDNPP EFLQESYFVE VSEDKEVHSE IIQVEATDKD

**851** LGPNGHVTYS IVTDTDTFSI DSVTGVVNIA RPLDRELQHE HSLKIEARDQ

**901** AR**EEPQLFST VVVK**VSLEDV NDNPPTFIPP NYRVKVREDL PEGTVIMWLE

**951** AHDPDLGQSG QVRYSLLDHG EGNFDVDKLS GAVR**IVQQLD FEK**KQVYNLT

**1001** VRAKDKGKPV SLSSTCYVEV EVVDVNENLH PPVFSSFVEK GTVKEDAPVG

**1051** SLVMTVSAHD EDARRDGEIR YSIRDGSGVG VFKIGEETGV IETSDRLDRE

**1101** STSHYWLTVF ATDQGVVPLS SFIEIYIEVE DVNDNAPQTS EPVYYPEIME

**1151** NSPKDVSVVQ IEAFDPDSSS NDKLMYKITS GNPQGFFSIH PKTGLITTTS

**1201** RKLDREQQDE HILEVTVTDN GSPPKSTIAR VIVKILDEND NKPQFLQKFY

**1251** KIRLPEREKP DRERNARREP LYHVIATDKD EGPNAEISYS IEDGNEHGKF

**1301** FIEPKTGVVS SKRFSAAGEY DILSIKAVDN GRPQKSSTTR LHIEWISKPK

**1351** PSLEPISFEE SFFTFTVMES DPVAHMIGVI SVEPPGIPLW FDITGGNYDS

**1401** HFDVDKGTGT IIVAKPLDAE QKSNYNLTVE ATDGTTTILT QVFIKVIDTN

**1451** DHRPQFSTSK YEVVIPEDTA PETEILQISA VDQDEKNKLI YTLQSSRDPL

**1501** SLKKFRLDPA TGSLYTSEKL DHEAVHQHTL TVMVRDQDVP VKRNFARIVV

**1551** NVSDTNDHAP WFTASSYKGR VYESAAVGSV VLQVTALDKD KGKNAEVLYS

**1601** IESGTFGNIG NSFMIDPVLG SIKTAKELDR SNQAEYDLMV KATDKGSPPM

**1651** SEITSVR**IFV TIADNASPK**F TSKEYSVELS ETVSIGSFVG MVTAHSQSSV

**1701** VYEIKDGNTG DAFDINPHSG TIITQKALDF ETLPIYTLII QGTNMAGLST

**1751** NTTVLVHLQD ENDNAPVFMQ AEYTGLISES ASINSVVLTD RNVPLVIRAA

**1801** DADKDSNALL VYHIVEPSVH TYFAIDSSTG AIHTVLSLDY EETSIFHFTV

**1851** QVHDMGTPRL FAEYAANVTV HVIDINDCPP VFAKPLYEAS LLLPTYKGVK

**1901** VITVNATDAD SSAFSQLIYS ITEGNIGEKF SMDYKTGALT VQNTTQLRSR

**1951** YELTVRASDG RFAGLTSVKI NVKESKESHL K**FTQDVYSAV VK**ENSTEAET

**2001** LAVITAIGNP INEPLFYHIL NPDRRFKISR TSGVLSTTGT PFDREQQEAF

**2051** DVVVEVTEEH KPSAVAHVVV KVIVEDQNDN APVFVNLPYY AVVKVDTEVG

**2101** HVIRYVTAVD RDSGRNGEVH YYLKEHHEHF QIGPLGEISL KKQFELDTLN

**2151** KEYLVTVVAK DGGNPAFSAE VIVPITVMNK AMPVFEKPFY SAEIAESIQV

**2201** HSPVVHVQAN SPEGLKVFYS ITDGDPFSQF TINFNTGVIN VIAPLDFEAH

**2251** PAYKLSIRAT DSLTGAHAEV FVDIIVDDIN DNPPVFAQQS YAVTLSEASV

**2301** IGTSVVQVRA TDSDSEPNRG ISYQMFGNHS KSHDHFHVDS STGLISLLRT

**2351** LDYEQSRQHT IFVRAVDGGM PTLSSDVIVT VDVTDLNDNP PLFEQQIYEA

**2401** RISEHAPHGH FVTCVKAYDA DSSDIDKLQY SILSGNDHK**H FVIDSATGII**

**2451 TLSNLHR**HAL KPFYSLNLSV SDGVFRSSTQ VHVTVIGGNL HSPAFLQNEY

**2501** EVELAENAPL HTLVMEVKTT DGDSGIYGHV TYHIVNDFAK DRFYINERGQ

**2551** IFTLEKLDRE TPAEKVISVR LMAKDAGGKV AFCTVNVILT DDNDNAPQFR

**2601** ATK**YEVNIGS SAAK**GTSVVK VLASDADEGS NADITYAIEA DSESVKENLE

**2651** INKLSGVITT KESLIGLENE FFTFFVRAVD NGSPSKESVV LVYVKILPPE

**2701** MQLPKFSEPF YTFTVSEDVP IGTEIDLIRA EHSGTVLYSL VKGNTPESNR

**2751** DESFVIDRQS GRLKLEKSLD HETTKWYQFS ILARCTQDDH EMVASVDVSI

**2801** QVKDANDNSP VFESSPYEAF IVENLPGGSR VIQIRASDAD SGTNGQVMYS

**2851** LDQSQSVEVI ESFAINMETG WITTLKELDH EKRDNYQIKV VASDHGEKIQ

**2901** LSSTAIVDVT VTDVNDSPPR FTAEIYKGTV SEDDPQGGVI AILSTTDADS

**2951** EEINRQVTYF ITGGDPLGQF AVETIQNEWK VYVKKPLDRE KRDNYLLTIT

**3001** ATDGTFSSKA IVEVKVLDAN DNSPVCEK**TL YSDTIPEDVL PGKLIMQISA**

**3051 TDADIR**SNAE ITYTLLGSGA EKFKLNPDTG ELKTSTPLDR EEQAVYHLLV

**3101** RATDGGGRFC QASIVLTLED VNDNAPEFSA DPYAITVFEN TEPGTLLTRV

**3151** QATDADAGLN RKILYSLIDS ADGQFSINEL SGIIQLEKPL DRELQAVYTL

**3201** SLKAVDQGLP RRLTATGTVI VSVLDINDNP PVFEYREYGA TVSEDILVGT

**3251** EVLQVYAASR DIEANAEITY SIISGNEHGK FSIDSKTGAV FIIENLDYES

**3301** SHEYYLTVEA TDGGTPSLSD VATVNVNVTD INDNTPVFSQ DTYTTVISED

**3351** AVLEQSVITV MADDADGPSN SHIHYSIIDG NQGSSFTIDP VRGEVKVTKL

**3401** LDRETISGYT LTVQASDNGS PPRVNTTTVN IDVSDVNDNA PVFSRGNYSV

**3451** IIQENKPVGF SVLQLVVTDE DSSHNGPPFF FTIVTGNDEK **AFEVNPQGVL**

**3501 LTSSAIK**RKE KDHYLLQVKV ADNGKPQLSS LTYIDIRVIE ESIYPPAILP

**3551** LEIFITSSGE EYSGGVIGKI HATDQDVYDT LTYSLDPQMD NLFSVSSTGG

**3601** KLIAHKKLDI GQYLLNVSVT DGKFTTVADI TVHIRQVTQE MLNHTIAIRF

**3651** ANLTPEEFVG DYWRNFQRAL RNILGVRRND IQIVSLQSSE PHPHLDVLLF

**3701** VEKPGSAQIS TKQLLHKINS SVTDIEEIIG VRILNVFQKL CAGLDCPWKF

**3751** CDEKVSVDES VMSTHSTARL SFVTPRHHRA AVCLCKEGRC PPVHHGCEDD

**3801** PCPEGSECVS DPWEEKHTCV CPSGRFGQCP GSSSMTLTGN SYVKYRLTEN

**3851** ENKLEMKLTM RLRTYSTHAV VMYARGTDYS ILEIHHGRLQ YKFDCGSGPG

**3901** IVSVQSIQVN DGQWHAVALE VNGNYARLVL DQVHTASGTA PGTLKTLNLD

**3951** NYVFFGGHIR QQGTRHGRSP QVGNGFRGCM DSIYLNGQEL PLNSKPRSYA

**4001** HIEESVDVSP GCFLTATEDC ASNPCQNGGV CNPSPAGGYY CKCSALYIGT

**4051** HCEISVNPCS SKPCLYGGTC VVDNGGFVCQ CRGLYTGQRC QLSPYCKDEP

**4101** CKNGGTCFDS LDGAVCQCDS GFRGERCQSD IDECSGNPCL HGALCENTHG

**4151** SYHCNCSHEY RGRHCEDAAP NQYVSTPWNI GLAEGIGIVV FVAGIFLLVV

**4201** VFVLCRKMIS RKKKHQAEPK DKHLGPATAF LQRPYFDSKL NKNIYSDIPP

**4251** QVPVRPISYT PSIPSDSRNN LDRNSFEGSA IPEHPEFSTF NPESVHGHRK

**4301** AVAVCSVAPN LPPPPPSNSP SDSDSIQKPS WDFDYDTKVV DLDPCLSKKP

**4351** LEEKPSQPYS ARESLSEVQS LSSFQSESCD DNGYHWDTSD WMPSVPLPDI

**4401** QEFPNYEVID EQTPLYSADP NAIDTDYYPG GYDIESDFPP PPEDFPAADE

**4451** LPPLPPEFSN QFESIHPPRD MPAAGSLGSS SRNRQRFNLN QYLPNFYPLD

**4501** MSEPQTKGTG ENSTCREPHA PYPPGYQRHF EAPAVESMPM SVYASTASCS

**4551** DVSACCEVES EVMMSDYESG DDGHFEEVTI PPLDSQQHTE VJDGSQRLEQ

**4601** TPLQFTHLEY NVTVQENSAA KJGTVKEDAP VGSLVMTVSA HDEDAGRDGE

**4651** IRJGTVKEDA PVGSLVMTVS AHDEDARRDG EIRJLDREST SHYWLTVFAT

**4701** DQGVVPLSSF IEIYIEVEDV NDNAPQTSEP VYYPEIMENS PKDVSVVQIE

**4751** AFDPDSSSND KJLDRESTSH YWLTVFATDQ GVVPLSSFIE LYIEVEDVND

**4801** NAPQTSEPVY YPEIMENSPK DVSVVQIEAF DPDSSSNDKJ FYKIRLPERJ

**4851** REPLYHVIAT DKDEGPNAEI SYSIEDGNEH GKJREPLYRV IATDKDEGPN

**4901** AEISYSIEDG NEHGKJEPLY HVIATDKDEG LNAEISYSIE DGNEHGKFFI

**4951** EPKJEPLYHV IATDKDEGPN AEISYSIEDG NEHGKFFIEP KJALEKNTNL

**5001** EARTKJALEK NTNVEARTKJ ALEKNTNVEA RTKJALEKNT NVEVRTKJFS

**5051** AAGEYDILSI KAVDNGRPQK SSTTRJFSAA GEYDILSIKA VDSGRPQKSS

**5101** TTRJNFARIV VNVSDTNDHA PWFTASSYKG RJNFARIVVN VSDTNDHAPW

**5151** FTTSSYKGRJ GKNAEVLYSI ESGDFGNIGN SFMIDPVLGS IKTAKJGKNA

**5201** EVLYSIESGN FGNIGNSFMI DPVLGSIKTA KJGKNAEVLY SIESGTIGNI

**5251** GNSFMIDPVL GSIKTAKJGS PPMSEITSVR IFVTNADNAS PKFTSKJGSP

**5301** PMSEITSVRI FVTSADNASP KFTSKJGSPP MSEITSVRIF VTIADNASTK

**5351** FTSKJALDFE TLPIYTLIIQ GTNMAGLSTN TTVLVHLQDE NDNAPVFMQA

**5401** EYTGLISESA SINSVVLTDR NVPQVIRAAD ADKJALDFET LPIYTLIIQG

**5451** TNMAGLSTNT TVLVHLQDEN DNAPVFMQAE YTGLISESAS INSVVLTDRN

**5501** VPRVIRAADA DKJAADADKD SNVLLVYHIV EPSVHTYFAI DSSTGAIHTV

**5551** LSLDYEETSI FHFTVQVHDM GTPRLFAEYA ANVTVHVIDI NDCPPVFAKP

**5601** LYEASLLLPT YKJFTQDVYS AVVKTNSTEA ETLAVITAIG NPINEPLFYH

**5651** ILNPDRRJTS GVLSTTGTPF DREAQEAFDV VVEVTEEHKP SAVAHVVVKV

**5701** IVEDQNDNAP VFVNLPYYAV VKJTDMFAIH PTSGVIVLTG RLDFLETKLY

**5751** EMEILAADRJ TDMFAIHPTS GVIVLTGRLD YLETKLYEME ILAADRJNGE

**5801** VHYYLKEHHE HFQIGPLGEK SLKKJDGGNP AFSAEVIVPI TVMNKAMPVF

**5851** EKPFYSAEIA ESIQVHSHVV HVQANSPEGL KVFYSITDGD PFSQFTINFN

**5901** TGVINVIAPL DFEAHPAYKJ AMPVFEKPFY SAEIAESIQV HSPVVHVQAN

**5951** SPEGLKVFYS ITDGDPFSQF TINFNTGVIN VIAPLDFPAH PAYKLSIRJL

**6001** SIRATDSLTG AHAEVFVDDI VDDINDNPPV FAQQSYAVTL SEASVIGTSV

**6051** VQVRATDSDS EPNRJLSIRA TDSLTGAHAE VFVDEIVDDI NDNPPVFAQQ

**6101** SYAVTLSEAS VIGTSVVQVR ATDSDSEPNR JTLDYEQSRQ HTIAVRAVDG

**6151** GMPTLSSDVI VTVDVTDLND NPPLFEQQIY EARJLYGSSG ISSMAKLTVH

**6201** IEQANECAPV ITAVTLSPSE LDRDPAYAIV TVDDCDQGAN GDIASLSIVA

**6251** GDLLQQFRJI LPPEMQLPKF SEPFYTFTVS EIVPIGTEID LIRAEHSGTV

**6301** LYSLVKJILP PEMQLPKFSE PFYTFTVSEV VPIGTEIDLI RAEHSGTVLY

**6351** SLVKJCTQDD HEMVASVDVS IQVKDANDNS PVFESSPYEA FIVENLPGGS

**6401** RVIQIRJCTQ DDHEMVASVD VSIQVKDASD NSPVFESSPY EAFIVENLPG

**6451** GSRVIQIRJV IQIRASDADN GTNGQVMYSL DQSQSVEVIE SFAINMETGW

**6501** ITTLKELDHE KJFTAEIYKG TVSELDPQGG VIAILSTTDA DSEEINRQVT

**6551** YFITGGDPLG QFAVETIQNE WKJFTAEIYK GTVSEPDPQG GVIAILSTTD

**6601** ADSEEINRQV TYFITGGDPL GQFAVETIQN EWKJFTAEIY KGTVSEQDPQ

**6651** GGVIAILSTT DADSEEINRQ VTYFITGGDP LGQFAVETIQ NEWKJGTVSE

**6701** DDPQGGVIAI LSTTDADSEE INRQVTYGIT GGDPLGQFAV ETIQNEWKVY

**6751** VKJGTVSEDD PQGGVIAILS TTDADSEEIN RQVTYSITGG DPLGQFAVET

**6801** IQNEWKVYVK JGTVSEDDPQ GGVIAILSTT DADSEEINRQ VTYFITGGDP

**6851** LAQFAVETIQ NEWKVYVKJA IVEVKVLDAN DNSPVCEYTL YSDTIPEDVL

**6901** PGKLIMQISA TDADIRJVLD ANDNSPVCEK TLYIDTIPED VLPGKLIMQI

**6951** SATDADIRJV LDANDNSPVC EKTLYTDTIP EDVLPGKLIM QISATDADIR

**7001** JATDGGGRFC QASIVLDLED VNDNAPEFSA DPYAITVFEN TEPGTLLTRV

**7051** QATDADAGLN RJKILYSLID SADGQFSINE LIGIIQLEKP LDRELQAVYT

**7101** LSLKJFSIDS KTGAVFIIEN LDYESSHEYY LTVEATDGGT PSLSDVATVN

**7151** VNVTDINDAT PVFSQDTYTT VISEDAVLEQ SVITVMADDA DGPSNSHIHY

**7201** SIIDGNQGSS FTIDPVRGEV KJFSIDSKTG AVFIIENLDY ESSHEYYLTV

**7251** EATDGGTPSL SDVATVNVNV TDINDVTPVF SQDTYTTVIS EDAVLEQSVI

**7301** TVMADDADGP SNSHIHYSII DGNQGSSFTI DPVRGEVKJV NTTTVNIDVS

**7351** DVNDNAPVFS RGNYSVIIQE NKPVGFSVLQ LVVTDEDSSH NGPPFFFTIV

**7401** TENDEKAFEV NPQGVLLTSS AIKJVNTTTV NIDVSDVNDN APVFSRGNYS

**7451** VIIQENKPVG FSVLQLVVTD EDSSHNGPPF FFTIVTGNDE KAFEVNPQGV

**7501** LLTSSAIKJG NYSVIIQENK PVGFSVLQLV VTDEDSSHNG PPFFFTIVTG

**7551** NDEKAFEVNA QGVLLTSSAI KRJGNYSVII QENKPVGFSV LQLVVTDEDS

**7601** SHNGPPFFFT IVTGNDEKAF EVNVQGVLLT SSAIKRJVAD NGKPQLSSLT

**7651** YIDIRVIEES IYPPAILPLE IAITSSGEEY SGGVIGKIHA TDQDVYDTLT

**7701** YSLDPQMDNL FSVSSTGGKJ VADNGKPQLS SLTYIDIRVI EESIYPPAIL

**7751** PLEISITSSG EEYSGGVIGK IHATDQDVYD TLTYSLDPQM DNLFSVSSTG

**7801** GKJVIHVTSP QFKAGPVKFE KJQLLHKIMS SVTDIEEIIG VRILNVFQKJ

**7851** QLLHKIVSSV TDIEEIIGVR ILNVFQKJEG RCPPVHHGCE DHPCPEGSEC

**7901** VSDPWEEKHT CVCPSGRJEG RCPPVHHGCE DPPCPEGSEC VSDPWEEKHT

**7951** CVCPSGRJHT CVCPSGRFGG CPGSSSMTLT GNSYVKYRJH TCVCPSGRFG

**8001** RCPGSSSMTL TGNSYVKYRJ FGQCPGSSSM TLTGNSYVKT RLTENENKJG

**8051** CMDSIYLNGQ ELPLNSKPRS YAHIEESVPV SPGCFLTATE DCASNPCQNG

**8101** GVCNPSPAGG YYCKCSALYI GTHCEISVNP CSSKPCLYGG TCVVDNGGFV

**8151** CQCRJYVFKR TPGKAKJYVF KSTPGKAKJS YAHIEESVDV SPGCFLTATE

**8201** DCASNPCQNG GVCNPSPAGG YYCKCSALYI GTHCEISVNP KSSKPCLYGG

**8251** TCVVDNGGFV CQCRGLYTGQ RJSYAHIEES VDVSPGCFLT ATEDCASNPC

**8301** QNGGVCNPSP AGGYYCKCSA LYIGTHCEIS VNPNSSKPCL YGGTCVVDNG

**8351** GFVCQCRGLY TGQRJDEPCK NGGTCFDSAD GAVCQCDSGF RGERJGRHCA

**8401** DAAPNQYVST PWNIGLAEGI GIVVFVAGIF LLVVVFVLCR KJGRHCEDAA

**8451** PNQYVSTPWN IGLAEGIGIV VFVAGIFLLV VVFVLCRKJN IYSDIPPQVP

**8501** VRYISYTPSI PSDSRNNLDR JKAVAVCSVA PNLPPPPPSN SPSDSDSIQK

**8551** PSWDFDYDTK VVDLDPCLSK JKPLEEKPSQ PYSARESLSS VQSLSSFQSE

**8601** SCDDNGYHWD TSDWMPSVPL PDIQEFPNYE VIDEQTPLYS ADPNAIDTDY

**8651** YPGGYDIESD FPPPPEDFPA ADELPPLPPE FSNQFESIHP PRDMPAAGSL

**8701** GSSSRJKPLE EKPSQPYSAR ESLSEVQSLS SFQSESCDDN GYHWDTSDWM

**8751** PSVPLQDIQE FPNYEVIDEQ TPLYSADPNA IDTDYYPGGY DIESDFPPPP

**8801** EDFPAADELP PLPPEFSNQF ESIHPPRDMP AAGSLGSSSR JKPLEEKPSQ

**8851** PYSARESLSE VQSLSSFQSE SCDDNGYHWD TSDWMPSVPL PDIQEFPNYE

**8901** VIDPQTPLYS ADPNAIDTDY YPGGYDIESD FPPPPEDFPA ADELPPLPPE

**8951** FSNQFESIHP PRDMPAAGSL GSSSRJESLS EVQSLSSFQS ESCDDNGYHW

**9001** DTSDWMPSVP LPDIQEFPNY EVIDEQTPLY SADPNAIDTD YYPGGYDIES

**9051** DFPPPPEDFP AADELPPLPP EFSNQFESIH PPRDMPAAGS LGSSRRNRJE

**9101** SLSEVQSLSS FQSESCDDNG YHWDTSDWMP SVPLPDIQEF PNYEVIDEQT

**9151** PLYSADPNAI DTDYYPGGYD IESDFPPPPE DFPAADELPP LPPEFSNQFE

**9201** SIHPPRDMPA AGSLGSSWRN RJVLGANSNP PEFTQTAYKA AFDENVPIGT

**9251** TIMSLSAVDP DEGENGYVTY SIANLNHVPF AIDHFTGAVS TSENLDYELM

**9301** PRVYTLRJVL GANSNPPEFT QTAYKAAFDE NVPIGTTVMS LSAVDPDEGE

**9351** NGYVTYSIAN LNHVPFAIDH FTGAVSTSEN LDYELMPRVY TLRJVLGANS

**9401** NPPEFTQTAY KAAFDENVPI GTTVMSLSAV DPDEGENGYV TYSIANLNHV

**9451** PFAIDHFTGA VSTSENLDYE LMPRVYTLRJ INCEGTIPRD LGVGEQITTV

**9501** SAIDADELQL VQYQIEAGNE LDFFSLNPNS GVLSLKRJIN CEGTIPRDLG

**9551** VGEQITTVSA IDADELQLVQ YQIEAGNELD LFSLNPNSGV LSLKRJINCE

**9601** GTIPRDLGVG EQITTVSAID ADELQLVQYQ IEAGNELDFF SLNPNSGVLS

**9651** LKRJEVHSEI IQVEATDKDL GPNGHVTYSI LTDTDTFSID SVTGVVNIAR

**9701** PLDRELQHEH SLKJEVHSEI IQVEATDKDL GPNGHVTYSI VTDTDTFSID

**9751** SVTGVVNIAR PLDRELQHEH SLK

**Start - End Observed Mr(expt) Mr(calc) ppm Miss Sequence**

**192 - 209 644.3412 1930.0019 1929.9983 2 0 R.TDMFAIHPTSGVIVLTGR.L**  Oxidation (M) ([Ions score 29](http://mbp-mascot4/mascot/cgi/peptide_view.pl?file=../data/20091021/F006273.dat&query=2269&hit=1&index=IPI00031411&px=1&section=5&ave_thresh=22))

**453 - 469 918.9605 1835.9065 1835.9054 1 0 K.VLGANSNPPEFTQTAYK.A**  ([Ions score 45](http://mbp-mascot4/mascot/cgi/peptide_view.pl?file=../data/20091021/F006273.dat&query=2156&hit=1&index=IPI00031411&px=1&section=5&ave_thresh=22))

**903 - 914 688.3770 1374.7395 1374.7395 -0 0 R.EEPQLFSTVVVK.V**  ([Ions score 39](http://mbp-mascot4/mascot/cgi/peptide_view.pl?file=../data/20091021/F006273.dat&query=1472&hit=1&index=IPI00031411&px=1&section=5&ave_thresh=22))

**985 - 993 560.3060 1118.5975 1118.5972 0 0 R.IVQQLDFEK.K**  ([Ions score 33](http://mbp-mascot4/mascot/cgi/peptide_view.pl?file=../data/20091021/F006273.dat&query=817&hit=1&index=IPI00031411&px=1&section=5&ave_thresh=22))

**1658 - 1669 638.3510 1274.6875 1274.6870 0 0 R.IFVTIADNASPK.F**  ([Ions score 46](http://mbp-mascot4/mascot/cgi/peptide_view.pl?file=../data/20091021/F006273.dat&query=1307&hit=1&index=IPI00031411&px=1&section=5&ave_thresh=22))

**1658 - 1669 638.3519 1274.6892 1274.6870 2 0 R.IFVTIADNASPK.F**  ([Ions score 38](http://mbp-mascot4/mascot/cgi/peptide_view.pl?file=../data/20091021/F006273.dat&query=1308&hit=1&index=IPI00031411&px=1&section=5&ave_thresh=22))

**1982 - 1992 628.8306 1255.6466 1255.6449 1 0 K.FTQDVYSAVVK.E**  ([Ions score 51](http://mbp-mascot4/mascot/cgi/peptide_view.pl?file=../data/20091021/F006273.dat&query=1261&hit=1&index=IPI00031411&px=1&section=5&ave_thresh=22))

**2440 - 2457 665.3661 1993.0766 1993.0745 1 0 K.HFVIDSATGIITLSNLHR.H**  ([Ions score 45](http://mbp-mascot4/mascot/cgi/peptide_view.pl?file=../data/20091021/F006273.dat&query=2309&hit=1&index=IPI00031411&px=1&section=5&ave_thresh=22))

**2604 - 2614 569.7908 1137.5671 1137.5666 0 0 K.YEVNIGSSAAK.G**  ([Ions score 31](http://mbp-mascot4/mascot/cgi/peptide_view.pl?file=../data/20091021/F006273.dat&query=841&hit=1&index=IPI00031411&px=1&section=5&ave_thresh=22))

**3029 - 3043 824.4277 1646.8409 1646.8403 0 0 K.TLYSDTIPEDVLPGK.L**  ([Ions score 44](http://mbp-mascot4/mascot/cgi/peptide_view.pl?file=../data/20091021/F006273.dat&query=1841&hit=1&index=IPI00031411&px=1&section=5&ave_thresh=22))

**3044 - 3056 731.8821 1461.7496 1461.7497 -0 0 K.LIMQISATDADIR.S**  Oxidation (M) ([Ions score 45](http://mbp-mascot4/mascot/cgi/peptide_view.pl?file=../data/20091021/F006273.dat&query=1626&hit=1&index=IPI00031411&px=1&section=5&ave_thresh=22))

**3491 - 3507 887.4911 1772.9676 1772.9672 0 0 K.AFEVNPQGVLLTSSAIK.R**  ([Ions score 73](http://mbp-mascot4/mascot/cgi/peptide_view.pl?file=../data/20091021/F006273.dat&query=2055&hit=1&index=IPI00031411&px=1&section=5&ave_thresh=22))

**BxPc3 A2:**

Match to: **IPI00031411** Score: **395**

**Gene_Symbol=FAT1 Protocadherin Fat 1 lng=4591 # SP[4593,D,22,D]SNP[4646,g,1064,R]SNP[4677,r,1064,R]SNP[4713,i,1125,I]SNP[4791,l,1125,I]SNP[4844,i,1252,I]SNP[4856,h,1273,H]SNP[4889,r,1273,H]SNP[4931,l,1283,P]SNP[4969,p,1283,P]SNP[5000,l,129,V]SNP[5**

Found in search of C:\mgf\Orbidata\081107_ISW1295_BXPC3_A2.mgf

Nominal mass (M_r_): **1068827**; Calculated pI value: **4.55**

NCBI BLAST search of [IPI00031411](http://www.ncbi.nlm.nih.gov/blast/Blast.cgi?ALIGNMENTS=50&ALIGNMENT_VIEW=Pairwise&AUTO_FORMAT=Semiauto&CDD_SEARCH=on&CLIENT=web&COMPOSITION_BASED_STATISTICS=on&DATABASE=nr&DESCRIPTIONS=100&ENTREZ_QUERY=(none)&EXPECT=10&FILTER=L&FORMAT_BLOCK_ON_RESPAGE=None&FORMAT_OBJECT=Alignment&FORMAT_TYPE=HTML&GAPCOSTS=11+1&I_THRESH=0.001&LAYOUT=TwoWindows&MATRIX_NAME=BLOSUM62&NCBI_GI=on&PAGE=Proteins&PROGRAM=blastp&QUERY=IPI00031411&SERVICE=plain&SET_DEFAULTS.x=21&SET_DEFAULTS.y=7&SHOW_OVERVIEW=on&WORD_SIZE=3&END_OF_HTTPGET=Yes) against nr

Unformatted [sequence string](http://mbp-mascot4/mascot/cgi/getseq.pl?IPI_human+IPI00031411+seq) for pasting into other applications

Fixed modifications: Carbamidomethyl (C)

Variable modifications: Oxidation (M)

Cleavage by TrypsinMSIPI, a mixture of enzymes:

cuts C-term side of KR unless next residue is P

cuts C-term side of J

cuts N-term side of J

Sequence Coverage: **2%**

Matched peptides shown in **Bold Red**

**1** MGRHLALLLL LLLLFQHFGD SDGSQRLEQT PLQFTHLEYN VTVQENSAAK

**51** TYVGHPVKMG VYITHPAWEV RYKIVSGDSE NLFKAEEYIL GDFCFLRIRT

**101** KGGNTAILNR EVKDHYTLIV KALEKNTNVE ARTKVRVQVL DTNDLRPLFS

**151** PTSYSVSLPE NTAIRTSIAR VSATDADIGT NGEFYYSFKD RTDMFAIHPT

**201** SGVIVLTGRL DYLETKLYEM EILAADRGMK LYGSSGISSM AKLTVHIEQA

**251** NECAPVITAV TLSPSELDRD PAYAIVTVDD CDQGANGDIA SLSIVAGDLL

**301** QQFRTVRSFP GSKEYKVKAI GGIDWDSHPF GYNLTLQAKD KGTPPQFSSV

**351** KVIHVTSPQF KAGPVKFEKD VYRAEISEFA PPNTPVVMVK AIPAYSHLRY

**401** VFKSTPGKAK FSLNYNTGLI SILEPVKRQQ AAHFELEVTT SDRKASTKVL

**451** VK**VLGANSNP PEFTQTAYK**A AFDENVPIGT TVMSLSAVDP DEGENGYVTY

**501** SIANLNHVPF AIDHFTGAVS TSENLDYELM PRVYTLRIRA SDWGLPYRRE

**551** VEVLATITLN NLNDNTPLFE KINCEGTIPR DLGVGEQITT VSAIDADELQ

**601** LVQYQIEAGN ELDFFSLNPN SGVLSLKRSL MDGLGAKVSF HSLRITATDG

**651** ENFATPLYIN ITVAASHKLV NLQCEETGVA KMLAEKLLQA NKLHNQGEVE

**701** DIFFDSHSVN AHIPQFRSTL PTGIQVKENQ PVGSSVIFMN STDLDTGFNG

**751** KLVYAVSGGN EDSCFMIDME TGMLKILSPL DRETTDKYTL NITVYDLGIP

**801** QKAAWRLLHV VVVDANDNPP EFLQESYFVE VSEDKEVHSE IIQVEATDKD

**851** LGPNGHVTYS IVTDTDTFSI DSVTGVVNIA RPLDRELQHE HSLKIEARDQ

**901** AREEPQLFST VVVKVSLEDV NDNPPTFIPP NYRVKVREDL PEGTVIMWLE

**951** AHDPDLGQSG QVR**YSLLDHG EGNFDVDK**LS GAVR**IVQQLD FEK**KQVYNLT

**1001** VRAKDKGKPV SLSSTCYVEV EVVDVNENLH PPVFSSFVEK GTVKEDAPVG

**1051** SLVMTVSAHD EDARRDGEIR YSIRDGSGVG VFK**IGEETGV IETSDRLDR**E

**1101** STSHYWLTVF ATDQGVVPLS SFIEIYIEVE DVNDNAPQTS EPVYYPEIME

**1151** NSPKDVSVVQ IEAFDPDSSS NDKLMYK**ITS GNPQGFFSIH PK**TGLITTTS

**1201** RKLDREQQDE HILEVTVTDN GSPPKSTIAR VIVK**ILDEND NKPQFLQK**FY

**1251** KIRLPEREKP DRERNARREP LYHVIATDKD EGPNAEISYS IEDGNEHGKF

**1301** FIEPKTGVVS SKR**FSAAGEY DILSIK**AVDN GRPQKSSTTR LHIEWISKPK

**1351** PSLEPISFEE SFFTFTVMES DPVAHMIGVI SVEPPGIPLW FDITGGNYDS

**1401** HFDVDKGTGT IIVAKPLDAE QKSNYNLTVE ATDGTTTILT QVFIKVIDTN

**1451** DHRPQFSTSK YEVVIPEDTA PETEILQISA VDQDEKNK**LI YTLQSSR**DPL

**1501** SLKKFRLDPA TGSLYTSEKL DHEAVHQHTL TVMVRDQDVP VKRNFARIVV

**1551** NVSDTNDHAP WFTASSYKGR VYESAAVGSV VLQVTALDKD KGKNAEVLYS

**1601** IESGTFGNIG NSFMIDPVLG SIKTAKELDR **SNQAEYDLMV K**ATDK**GSPPM**

**1651 SEITSVRIFV TIADNASPK**F TSKEYSVELS ETVSIGSFVG MVTAHSQSSV

**1701** VYEIKDGNTG DAFDINPHSG TIITQKALDF ETLPIYTLII QGTNMAGLST

**1751** NTTVLVHLQD ENDNAPVFMQ AEYTGLISES ASINSVVLTD RNVPLVIRAA

**1801** DADKDSNALL VYHIVEPSVH TYFAIDSSTG AIHTVLSLDY EETSIFHFTV

**1851** QVHDMGTPRL FAEYAANVTV HVIDINDCPP VFAKPLYEAS LLLPTYKGVK

**1901** VITVNATDAD SSAFSQLIYS ITEGNIGEKF SMDYKTGALT VQNTTQLRSR

**1951** YELTVRASDG RFAGLTSVKI NVKESKESHL K**FTQDVYSAV VK**ENSTEAET

**2001** LAVITAIGNP INEPLFYHIL NPDRRFKISR TSGVLSTTGT PFDREQQEAF

**2051** DVVVEVTEEH KPSAVAHVVV KVIVEDQNDN APVFVNLPYY AVVKVDTEVG

**2101** HVIRYVTAVD RDSGRNGEVH YYLKEHHEHF QIGPLGEISL KK**QFELDTLN**

**2151 K**EYLVTVVAK DGGNPAFSAE VIVPITVMNK AMPVFEKPFY SAEIAESIQV

**2201** HSPVVHVQAN SPEGLKVFYS ITDGDPFSQF TINFNTGVIN VIAPLDFEAH

**2251** PAYKLSIRAT DSLTGAHAEV FVDIIVDDIN DNPPVFAQQS YAVTLSEASV

**2301** IGTSVVQVRA TDSDSEPNRG ISYQMFGNHS KSHDHFHVDS STGLISLLRT

**2351** LDYEQSRQHT IFVRAVDGGM PTLSSDVIVT VDVTDLNDNP PLFEQQIYEA

**2401** RISEHAPHGH FVTCVKAYDA DSSDIDKLQY SILSGNDHK**H FVIDSATGII**

**2451 TLSNLHR**HAL KPFYSLNLSV SDGVFRSSTQ VHVTVIGGNL HSPAFLQNEY

**2501** EVELAENAPL HTLVMEVKTT DGDSGIYGHV TYHIVNDFAK DRFYINERGQ

**2551** IFTLEKLDRE TPAEKVISVR LMAKDAGGKV AFCTVNVILT DDNDNAPQFR

**2601** ATK**YEVNIGS SAAK**GTSVVK VLASDADEGS NADITYAIEA DSESVKENLE

**2651** INKLSGVITT KESLIGLENE FFTFFVRAVD NGSPSK**ESVV LVYVK**ILPPE

**2701** MQLPKFSEPF YTFTVSEDVP IGTEIDLIRA EHSGTVLYSL VKGNTPESNR

**2751** DESFVIDRQS GRLKLEKSLD HETTKWYQFS ILARCTQDDH EMVASVDVSI

**2801** QVKDANDNSP VFESSPYEAF IVENLPGGSR VIQIRASDAD SGTNGQVMYS

**2851** LDQSQSVEVI ESFAINMETG WITTLKELDH EKRDNYQIKV VASDHGEKIQ

**2901** LSSTAIVDVT VTDVNDSPPR FTAEIYKGTV SEDDPQGGVI AILSTTDADS

**2951** EEINRQVTYF ITGGDPLGQF AVETIQNEWK VYVKKPLDRE KRDNYLLTIT

**3001** ATDGTFSSKA IVEVKVLDAN DNSPVCEK**TL YSDTIPEDVL PGK**LIMQISA

**3051** TDADIRSNAE ITYTLLGSGA EKFKLNPDTG ELKTSTPLDR EEQAVYHLLV

**3101** RATDGGGRFC QASIVLTLED VNDNAPEFSA DPYAITVFEN TEPGTLLTRV

**3151** QATDADAGLN RKILYSLIDS ADGQFSINEL SGIIQLEKPL DRELQAVYTL

**3201** SLKAVDQGLP RRLTATGTVI VSVLDINDNP PVFEYREYGA TVSEDILVGT

**3251** EVLQVYAASR DIEANAEITY SIISGNEHGK FSIDSKTGAV FIIENLDYES

**3301** SHEYYLTVEA TDGGTPSLSD VATVNVNVTD INDNTPVFSQ DTYTTVISED

**3351** AVLEQSVITV MADDADGPSN SHIHYSIIDG NQGSSFTIDP VRGEVKVTKL

**3401** LDRETISGYT LTVQASDNGS PPRVNTTTVN IDVSDVNDNA PVFSRGNYSV

**3451** IIQENKPVGF SVLQLVVTDE DSSHNGPPFF FTIVTGNDEK **AFEVNPQGVL**

**3501 LTSSAIK**RKE KDHYLLQVKV ADNGKPQLSS LTYIDIRVIE ESIYPPAILP

**3551** LEIFITSSGE EYSGGVIGKI HATDQDVYDT LTYSLDPQMD NLFSVSSTGG

**3601** KLIAHKKLDI GQYLLNVSVT DGKFTTVADI TVHIRQVTQE MLNHTIAIRF

**3651** ANLTPEEFVG DYWRNFQRAL RNILGVRRND IQIVSLQSSE PHPHLDVLLF

**3701** VEKPGSAQIS TKQLLHKINS SVTDIEEIIG VRILNVFQKL CAGLDCPWKF

**3751** CDEKVSVDES VMSTHSTARL SFVTPRHHRA AVCLCKEGRC PPVHHGCEDD

**3801** PCPEGSECVS DPWEEKHTCV CPSGRFGQCP GSSSMTLTGN SYVKYRLTEN

**3851** ENKLEMKLTM RLRTYSTHAV VMYARGTDYS ILEIHHGRLQ YKFDCGSGPG

**3901** IVSVQSIQVN DGQWHAVALE VNGNYARLVL DQVHTASGTA PGTLKTLNLD

**3951** NYVFFGGHIR QQGTRHGRSP QVGNGFRGCM DSIYLNGQEL PLNSKPRSYA

**4001** HIEESVDVSP GCFLTATEDC ASNPCQNGGV CNPSPAGGYY CKCSALYIGT

**4051** HCEISVNPCS SKPCLYGGTC VVDNGGFVCQ CRGLYTGQRC QLSPYCKDEP

**4101** CKNGGTCFDS LDGAVCQCDS GFRGERCQSD IDECSGNPCL HGALCENTHG

**4151** SYHCNCSHEY RGRHCEDAAP NQYVSTPWNI GLAEGIGIVV FVAGIFLLVV

**4201** VFVLCRKMIS RKKKHQAEPK DKHLGPATAF LQRPYFDSKL NKNIYSDIPP

**4251** QVPVRPISYT PSIPSDSRNN LDRNSFEGSA IPEHPEFSTF NPESVHGHRK

**4301** AVAVCSVAPN LPPPPPSNSP SDSDSIQKPS WDFDYDTKVV DLDPCLSKKP

**4351** LEEKPSQPYS ARESLSEVQS LSSFQSESCD DNGYHWDTSD WMPSVPLPDI

**4401** QEFPNYEVID EQTPLYSADP NAIDTDYYPG GYDIESDFPP PPEDFPAADE

**4451** LPPLPPEFSN QFESIHPPRD MPAAGSLGSS SRNRQRFNLN QYLPNFYPLD

**4501** MSEPQTKGTG ENSTCREPHA PYPPGYQRHF EAPAVESMPM SVYASTASCS

**4551** DVSACCEVES EVMMSDYESG DDGHFEEVTI PPLDSQQHTE VJDGSQRLEQ

**4601** TPLQFTHLEY NVTVQENSAA KJGTVKEDAP VGSLVMTVSA HDEDAGRDGE

**4651** IRJGTVKEDA PVGSLVMTVS AHDEDARRDG EIRJLDREST SHYWLTVFAT

**4701** DQGVVPLSSF IEIYIEVEDV NDNAPQTSEP VYYPEIMENS PKDVSVVQIE

**4751** AFDPDSSSND KJLDRESTSH YWLTVFATDQ GVVPLSSFIE LYIEVEDVND

**4801** NAPQTSEPVY YPEIMENSPK DVSVVQIEAF DPDSSSNDKJ FYKIRLPERJ

**4851** REPLYHVIAT DKDEGPNAEI SYSIEDGNEH GKJREPLYRV IATDKDEGPN

**4901** AEISYSIEDG NEHGKJEPLY HVIATDKDEG LNAEISYSIE DGNEHGKFFI

**4951** EPKJEPLYHV IATDKDEGPN AEISYSIEDG NEHGKFFIEP KJALEKNTNL

**5001** EARTKJALEK NTNVEARTKJ ALEKNTNVEA RTKJALEKNT NVEVRTKJFS

**5051** AAGEYDILSI KAVDNGRPQK SSTTRJFSAA GEYDILSIKA VDSGRPQKSS

**5101** TTRJNFARIV VNVSDTNDHA PWFTASSYKG RJNFARIVVN VSDTNDHAPW

**5151** FTTSSYKGRJ GKNAEVLYSI ESGDFGNIGN SFMIDPVLGS IKTAKJGKNA

**5201** EVLYSIESGN FGNIGNSFMI DPVLGSIKTA KJGKNAEVLY SIESGTIGNI

**5251** GNSFMIDPVL GSIKTAKJGS PPMSEITSVR IFVTNADNAS PKFTSKJGSP

**5301** PMSEITSVRI FVTSADNASP KFTSKJGSPP MSEITSVRIF VTIADNASTK

**5351** FTSKJALDFE TLPIYTLIIQ GTNMAGLSTN TTVLVHLQDE NDNAPVFMQA

**5401** EYTGLISESA SINSVVLTDR NVPQVIRAAD ADKJALDFET LPIYTLIIQG

**5451** TNMAGLSTNT TVLVHLQDEN DNAPVFMQAE YTGLISESAS INSVVLTDRN

**5501** VPRVIRAADA DKJAADADKD SNVLLVYHIV EPSVHTYFAI DSSTGAIHTV

**5551** LSLDYEETSI FHFTVQVHDM GTPRLFAEYA ANVTVHVIDI NDCPPVFAKP

**5601** LYEASLLLPT YKJFTQDVYS AVVKTNSTEA ETLAVITAIG NPINEPLFYH

**5651** ILNPDRRJTS GVLSTTGTPF DREAQEAFDV VVEVTEEHKP SAVAHVVVKV

**5701** IVEDQNDNAP VFVNLPYYAV VKJTDMFAIH PTSGVIVLTG RLDFLETKLY

**5751** EMEILAADRJ TDMFAIHPTS GVIVLTGRLD YLETKLYEME ILAADRJNGE

**5801** VHYYLKEHHE HFQIGPLGEK SLKKJDGGNP AFSAEVIVPI TVMNKAMPVF

**5851** EKPFYSAEIA ESIQVHSHVV HVQANSPEGL KVFYSITDGD PFSQFTINFN

**5901** TGVINVIAPL DFEAHPAYKJ AMPVFEKPFY SAEIAESIQV HSPVVHVQAN

**5951** SPEGLKVFYS ITDGDPFSQF TINFNTGVIN VIAPLDFPAH PAYKLSIRJL

**6001** SIRATDSLTG AHAEVFVDDI VDDINDNPPV FAQQSYAVTL SEASVIGTSV

**6051** VQVRATDSDS EPNRJLSIRA TDSLTGAHAE VFVDEIVDDI NDNPPVFAQQ

**6101** SYAVTLSEAS VIGTSVVQVR ATDSDSEPNR JTLDYEQSRQ HTIAVRAVDG

**6151** GMPTLSSDVI VTVDVTDLND NPPLFEQQIY EARJLYGSSG ISSMAKLTVH

**6201** IEQANECAPV ITAVTLSPSE LDRDPAYAIV TVDDCDQGAN GDIASLSIVA

**6251** GDLLQQFRJI LPPEMQLPKF SEPFYTFTVS EIVPIGTEID LIRAEHSGTV

**6301** LYSLVKJILP PEMQLPKFSE PFYTFTVSEV VPIGTEIDLI RAEHSGTVLY

**6351** SLVKJCTQDD HEMVASVDVS IQVKDANDNS PVFESSPYEA FIVENLPGGS

**6401** RVIQIRJCTQ DDHEMVASVD VSIQVKDASD NSPVFESSPY EAFIVENLPG

**6451** GSRVIQIRJV IQIRASDADN GTNGQVMYSL DQSQSVEVIE SFAINMETGW

**6501** ITTLKELDHE KJFTAEIYKG TVSELDPQGG VIAILSTTDA DSEEINRQVT

**6551** YFITGGDPLG QFAVETIQNE WKJFTAEIYK GTVSEPDPQG GVIAILSTTD

**6601** ADSEEINRQV TYFITGGDPL GQFAVETIQN EWKJFTAEIY KGTVSEQDPQ

**6651** GGVIAILSTT DADSEEINRQ VTYFITGGDP LGQFAVETIQ NEWKJGTVSE

**6701** DDPQGGVIAI LSTTDADSEE INRQVTYGIT GGDPLGQFAV ETIQNEWKVY

**6751** VKJGTVSEDD PQGGVIAILS TTDADSEEIN RQVTYSITGG DPLGQFAVET

**6801** IQNEWKVYVK JGTVSEDDPQ GGVIAILSTT DADSEEINRQ VTYFITGGDP

**6851** LAQFAVETIQ NEWKVYVKJA IVEVKVLDAN DNSPVCEYTL YSDTIPEDVL

**6901** PGKLIMQISA TDADIRJVLD ANDNSPVCEK TLYIDTIPED VLPGKLIMQI

**6951** SATDADIRJV LDANDNSPVC EKTLYTDTIP EDVLPGKLIM QISATDADIR

**7001** JATDGGGRFC QASIVLDLED VNDNAPEFSA DPYAITVFEN TEPGTLLTRV

**7051** QATDADAGLN RJKILYSLID SADGQFSINE LIGIIQLEKP LDRELQAVYT

**7101** LSLKJFSIDS KTGAVFIIEN LDYESSHEYY LTVEATDGGT PSLSDVATVN

**7151** VNVTDINDAT PVFSQDTYTT VISEDAVLEQ SVITVMADDA DGPSNSHIHY

**7201** SIIDGNQGSS FTIDPVRGEV KJFSIDSKTG AVFIIENLDY ESSHEYYLTV

**7251** EATDGGTPSL SDVATVNVNV TDINDVTPVF SQDTYTTVIS EDAVLEQSVI

**7301** TVMADDADGP SNSHIHYSII DGNQGSSFTI DPVRGEVKJV NTTTVNIDVS

**7351** DVNDNAPVFS RGNYSVIIQE NKPVGFSVLQ LVVTDEDSSH NGPPFFFTIV

**7401** TENDEKAFEV NPQGVLLTSS AIKJVNTTTV NIDVSDVNDN APVFSRGNYS

**7451** VIIQENKPVG FSVLQLVVTD EDSSHNGPPF FFTIVTGNDE KAFEVNPQGV

**7501** LLTSSAIKJG NYSVIIQENK PVGFSVLQLV VTDEDSSHNG PPFFFTIVTG

**7551** NDEKAFEVNA QGVLLTSSAI KRJGNYSVII QENKPVGFSV LQLVVTDEDS

**7601** SHNGPPFFFT IVTGNDEKAF EVNVQGVLLT SSAIKRJVAD NGKPQLSSLT

**7651** YIDIRVIEES IYPPAILPLE IAITSSGEEY SGGVIGKIHA TDQDVYDTLT

**7701** YSLDPQMDNL FSVSSTGGKJ VADNGKPQLS SLTYIDIRVI EESIYPPAIL

**7751** PLEISITSSG EEYSGGVIGK IHATDQDVYD TLTYSLDPQM DNLFSVSSTG

**7801** GKJVIHVTSP QFKAGPVKFE KJQLLHKIMS SVTDIEEIIG VRILNVFQKJ

**7851** QLLHKIVSSV TDIEEIIGVR ILNVFQKJEG RCPPVHHGCE DHPCPEGSEC

**7901** VSDPWEEKHT CVCPSGRJEG RCPPVHHGCE DPPCPEGSEC VSDPWEEKHT

**7951** CVCPSGRJHT CVCPSGRFGG CPGSSSMTLT GNSYVKYRJH TCVCPSGRFG

**8001** RCPGSSSMTL TGNSYVKYRJ FGQCPGSSSM TLTGNSYVKT RLTENENKJG

**8051** CMDSIYLNGQ ELPLNSKPRS YAHIEESVPV SPGCFLTATE DCASNPCQNG

**8101** GVCNPSPAGG YYCKCSALYI GTHCEISVNP CSSKPCLYGG TCVVDNGGFV

**8151** CQCRJYVFKR TPGKAKJYVF KSTPGKAKJS YAHIEESVDV SPGCFLTATE

**8201** DCASNPCQNG GVCNPSPAGG YYCKCSALYI GTHCEISVNP KSSKPCLYGG

**8251** TCVVDNGGFV CQCRGLYTGQ RJSYAHIEES VDVSPGCFLT ATEDCASNPC

**8301** QNGGVCNPSP AGGYYCKCSA LYIGTHCEIS VNPNSSKPCL YGGTCVVDNG

**8351** GFVCQCRGLY TGQRJDEPCK NGGTCFDSAD GAVCQCDSGF RGERJGRHCA

**8401** DAAPNQYVST PWNIGLAEGI GIVVFVAGIF LLVVVFVLCR KJGRHCEDAA

**8451** PNQYVSTPWN IGLAEGIGIV VFVAGIFLLV VVFVLCRKJN IYSDIPPQVP

**8501** VRYISYTPSI PSDSRNNLDR JKAVAVCSVA PNLPPPPPSN SPSDSDSIQK

**8551** PSWDFDYDTK VVDLDPCLSK JKPLEEKPSQ PYSARESLSS VQSLSSFQSE

**8601** SCDDNGYHWD TSDWMPSVPL PDIQEFPNYE VIDEQTPLYS ADPNAIDTDY

**8651** YPGGYDIESD FPPPPEDFPA ADELPPLPPE FSNQFESIHP PRDMPAAGSL

**8701** GSSSRJKPLE EKPSQPYSAR ESLSEVQSLS SFQSESCDDN GYHWDTSDWM

**8751** PSVPLQDIQE FPNYEVIDEQ TPLYSADPNA IDTDYYPGGY DIESDFPPPP

**8801** EDFPAADELP PLPPEFSNQF ESIHPPRDMP AAGSLGSSSR JKPLEEKPSQ

**8851** PYSARESLSE VQSLSSFQSE SCDDNGYHWD TSDWMPSVPL PDIQEFPNYE

**8901** VIDPQTPLYS ADPNAIDTDY YPGGYDIESD FPPPPEDFPA ADELPPLPPE

**8951** FSNQFESIHP PRDMPAAGSL GSSSRJESLS EVQSLSSFQS ESCDDNGYHW

**9001** DTSDWMPSVP LPDIQEFPNY EVIDEQTPLY SADPNAIDTD YYPGGYDIES

**9051** DFPPPPEDFP AADELPPLPP EFSNQFESIH PPRDMPAAGS LGSSRRNRJE

**9101** SLSEVQSLSS FQSESCDDNG YHWDTSDWMP SVPLPDIQEF PNYEVIDEQT

**9151** PLYSADPNAI DTDYYPGGYD IESDFPPPPE DFPAADELPP LPPEFSNQFE

**9201** SIHPPRDMPA AGSLGSSWRN RJVLGANSNP PEFTQTAYKA AFDENVPIGT

**9251** TIMSLSAVDP DEGENGYVTY SIANLNHVPF AIDHFTGAVS TSENLDYELM

**9301** PRVYTLRJVL GANSNPPEFT QTAYKAAFDE NVPIGTTVMS LSAVDPDEGE

**9351** NGYVTYSIAN LNHVPFAIDH FTGAVSTSEN LDYELMPRVY TLRJVLGANS

**9401** NPPEFTQTAY KAAFDENVPI GTTVMSLSAV DPDEGENGYV TYSIANLNHV

**9451** PFAIDHFTGA VSTSENLDYE LMPRVYTLRJ INCEGTIPRD LGVGEQITTV

**9501** SAIDADELQL VQYQIEAGNE LDFFSLNPNS GVLSLKRJIN CEGTIPRDLG

**9551** VGEQITTVSA IDADELQLVQ YQIEAGNELD LFSLNPNSGV LSLKRJINCE

**9601** GTIPRDLGVG EQITTVSAID ADELQLVQYQ IEAGNELDFF SLNPNSGVLS

**9651** LKRJEVHSEI IQVEATDKDL GPNGHVTYSI LTDTDTFSID SVTGVVNIAR

**9701** PLDRELQHEH SLKJEVHSEI IQVEATDKDL GPNGHVTYSI VTDTDTFSID

**9751** SVTGVVNIAR PLDRELQHEH SLK

**Start - End Observed Mr(expt) Mr(calc) ppm Miss Sequence**

**453 - 469 918.9595 1835.9044 1835.9054 -1 0 K.VLGANSNPPEFTQTAYK.A**  ([Ions score 35](http://mbp-mascot4/mascot/cgi/peptide_view.pl?file=../data/20091021/F006274.dat&query=2122&hit=1&index=IPI00031411&px=1&section=5&ave_thresh=22))

**964 - 978 570.2670 1707.7791 1707.7740 3 0 R.YSLLDHGEGNFDVDK.L**  ([Ions score 37](http://mbp-mascot4/mascot/cgi/peptide_view.pl?file=../data/20091021/F006274.dat&query=1946&hit=1&index=IPI00031411&px=1&section=5&ave_thresh=22))

**985 - 993 560.3068 1118.5990 1118.5972 2 0 R.IVQQLDFEK.K**  ([Ions score 41](http://mbp-mascot4/mascot/cgi/peptide_view.pl?file=../data/20091021/F006274.dat&query=895&hit=1&index=IPI00031411&px=1&section=5&ave_thresh=22))

**1084 - 1099 597.3018 1788.8834 1788.8854 -1 1 K.IGEETGVIETSDRLDR.E**  ([Ions score 24](http://mbp-mascot4/mascot/cgi/peptide_view.pl?file=../data/20091021/F006274.dat&query=2050&hit=1&index=IPI00031411&px=1&section=5&ave_thresh=22))

**1178 - 1192 543.9520 1628.8341 1628.8311 2 0 K.ITSGNPQGFFSIHPK.T**  ([Ions score 23](http://mbp-mascot4/mascot/cgi/peptide_view.pl?file=../data/20091021/F006274.dat&query=1808&hit=1&index=IPI00031411&px=1&section=5&ave_thresh=22))

**1235 - 1248 567.9655 1700.8747 1700.8733 1 0 K.ILDENDNKPQFLQK.F**  ([Ions score 22](http://mbp-mascot4/mascot/cgi/peptide_view.pl?file=../data/20091021/F006274.dat&query=1931&hit=1&index=IPI00031411&px=1&section=5&ave_thresh=22))

**1314 - 1326 707.3662 1412.7179 1412.7187 -1 0 R.FSAAGEYDILSIK.A**  ([Ions score 74](http://mbp-mascot4/mascot/cgi/peptide_view.pl?file=../data/20091021/F006274.dat&query=1600&hit=1&index=IPI00031411&px=1&section=5&ave_thresh=22))

**1489 - 1497 540.8060 1079.5975 1079.5975 0 0 K.LIYTLQSSR.D**  ([Ions score 44](http://mbp-mascot4/mascot/cgi/peptide_view.pl?file=../data/20091021/F006274.dat&query=787&hit=1&index=IPI00031411&px=1&section=5&ave_thresh=22))

**1489 - 1497 540.8063 1079.5980 1079.5975 0 0 K.LIYTLQSSR.D**  ([Ions score 43](http://mbp-mascot4/mascot/cgi/peptide_view.pl?file=../data/20091021/F006274.dat&query=788&hit=1&index=IPI00031411&px=1&section=5&ave_thresh=22))

**1631 - 1641 657.3080 1312.6015 1312.5969 4 0 R.SNQAEYDLMVK.A**  Oxidation (M) ([Ions score 21](http://mbp-mascot4/mascot/cgi/peptide_view.pl?file=../data/20091021/F006274.dat&query=1433&hit=1&index=IPI00031411&px=1&section=5&ave_thresh=22))

**1646 - 1657 638.8141 1275.6136 1275.6129 1 0 K.GSPPMSEITSVR.I**  Oxidation (M) ([Ions score 22](http://mbp-mascot4/mascot/cgi/peptide_view.pl?file=../data/20091021/F006274.dat&query=1363&hit=1&index=IPI00031411&px=1&section=5&ave_thresh=22))

**1658 - 1669 638.3509 1274.6872 1274.6870 0 0 R.IFVTIADNASPK.F**  ([Ions score 31](http://mbp-mascot4/mascot/cgi/peptide_view.pl?file=../data/20091021/F006274.dat&query=1359&hit=1&index=IPI00031411&px=1&section=5&ave_thresh=22))

**1658 - 1669 638.3510 1274.6873 1274.6870 0 0 R.IFVTIADNASPK.F**  ([Ions score 59](http://mbp-mascot4/mascot/cgi/peptide_view.pl?file=../data/20091021/F006274.dat&query=1360&hit=1&index=IPI00031411&px=1&section=5&ave_thresh=22))

**1982 - 1992 628.8300 1255.6453 1255.6449 0 0 K.FTQDVYSAVVK.E**  ([Ions score 40](http://mbp-mascot4/mascot/cgi/peptide_view.pl?file=../data/20091021/F006274.dat&query=1318&hit=1&index=IPI00031411&px=1&section=5&ave_thresh=22))

**2143 - 2151 554.2887 1106.5628 1106.5608 2 0 K.QFELDTLNK.E**  ([Ions score 21](http://mbp-mascot4/mascot/cgi/peptide_view.pl?file=../data/20091021/F006274.dat&query=878&hit=1&index=IPI00031411&px=1&section=5&ave_thresh=22))

**2440 - 2457 665.3654 1993.0744 1993.0745 -0 0 K.HFVIDSATGIITLSNLHR.H**  ([Ions score 53](http://mbp-mascot4/mascot/cgi/peptide_view.pl?file=../data/20091021/F006274.dat&query=2246&hit=1&index=IPI00031411&px=1&section=5&ave_thresh=22))

**2604 - 2614 569.7906 1137.5667 1137.5666 0 0 K.YEVNIGSSAAK.G**  ([Ions score 30](http://mbp-mascot4/mascot/cgi/peptide_view.pl?file=../data/20091021/F006274.dat&query=919&hit=1&index=IPI00031411&px=1&section=5&ave_thresh=22))

**2687 - 2695 518.3080 1034.6015 1034.6012 0 0 K.ESVVLVYVK.I**  ([Ions score 46](http://mbp-mascot4/mascot/cgi/peptide_view.pl?file=../data/20091021/F006274.dat&query=709&hit=1&index=IPI00031411&px=1&section=5&ave_thresh=22))

**3029 - 3043 824.4280 1646.8414 1646.8403 1 0 K.TLYSDTIPEDVLPGK.L**  ([Ions score 44](http://mbp-mascot4/mascot/cgi/peptide_view.pl?file=../data/20091021/F006274.dat&query=1852&hit=1&index=IPI00031411&px=1&section=5&ave_thresh=22))

**3491 - 3507 887.4908 1772.9671 1772.9672 -0 0 K.AFEVNPQGVLLTSSAIK.R**  ([Ions score 68](http://mbp-mascot4/mascot/cgi/peptide_view.pl?file=../data/20091021/F006274.dat&query=2030&hit=1&index=IPI00031411&px=1&section=5&ave_thresh=22))

**HPDE A2:**

Match to: **IPI00031411** Score: **185**

**Gene_Symbol=FAT1 Protocadherin Fat 1 lng=4591 # SP[4593,D,22,D]SNP[4646,g,1064,R]SNP[4677,r,1064,R]SNP[4713,i,1125,I]SNP[4791,l,1125,I]SNP[4844,i,1252,I]SNP[4856,h,1273,H]SNP[4889,r,1273,H]SNP[4931,l,1283,P]SNP[4969,p,1283,P]SNP[5000,l,129,V]SNP[5**

Found in search of C:\mgf\Orbidata\080723_ISW1295_HPDE_A2.mgf

Nominal mass (M_r_): **1068827**; Calculated pI value: **4.55**

NCBI BLAST search of [IPI00031411](http://www.ncbi.nlm.nih.gov/blast/Blast.cgi?ALIGNMENTS=50&ALIGNMENT_VIEW=Pairwise&AUTO_FORMAT=Semiauto&CDD_SEARCH=on&CLIENT=web&COMPOSITION_BASED_STATISTICS=on&DATABASE=nr&DESCRIPTIONS=100&ENTREZ_QUERY=(none)&EXPECT=10&FILTER=L&FORMAT_BLOCK_ON_RESPAGE=None&FORMAT_OBJECT=Alignment&FORMAT_TYPE=HTML&GAPCOSTS=11+1&I_THRESH=0.001&LAYOUT=TwoWindows&MATRIX_NAME=BLOSUM62&NCBI_GI=on&PAGE=Proteins&PROGRAM=blastp&QUERY=IPI00031411&SERVICE=plain&SET_DEFAULTS.x=21&SET_DEFAULTS.y=7&SHOW_OVERVIEW=on&WORD_SIZE=3&END_OF_HTTPGET=Yes) against nr

Unformatted [sequence string](http://mbp-mascot4/mascot/cgi/getseq.pl?IPI_human+IPI00031411+seq) for pasting into other applications

Fixed modifications: Carbamidomethyl (C)

Variable modifications: Oxidation (M)

Cleavage by TrypsinMSIPI, a mixture of enzymes:

cuts C-term side of KR unless next residue is P

cuts C-term side of J

cuts N-term side of J

Sequence Coverage: **0%**

Matched peptides shown in **Bold Red**

**1** MGRHLALLLL LLLLFQHFGD SDGSQRLEQT PLQFTHLEYN VTVQENSAAK

**51** TYVGHPVKMG VYITHPAWEV RYKIVSGDSE NLFK**AEEYIL GDFCFLR**IRT

**101** KGGNTAILNR EVKDHYTLIV KALEKNTNVE ARTKVRVQVL DTNDLRPLFS

**151** PTSYSVSLPE NTAIRTSIAR VSATDADIGT NGEFYYSFKD RTDMFAIHPT

**201** SGVIVLTGRL DYLETKLYEM EILAADRGMK LYGSSGISSM AKLTVHIEQA

**251** NECAPVITAV TLSPSELDRD PAYAIVTVDD CDQGANGDIA SLSIVAGDLL

**301** QQFRTVRSFP GSKEYKVKAI GGIDWDSHPF GYNLTLQAKD KGTPPQFSSV

**351** KVIHVTSPQF KAGPVKFEKD VYRAEISEFA PPNTPVVMVK AIPAYSHLRY

**401** VFKSTPGKAK FSLNYNTGLI SILEPVKRQQ AAHFELEVTT SDRKASTKVL

**451** VK**VLGANSNP PEFTQTAYK**A AFDENVPIGT TVMSLSAVDP DEGENGYVTY

**501** SIANLNHVPF AIDHFTGAVS TSENLDYELM PRVYTLRIRA SDWGLPYRRE

**551** VEVLATITLN NLNDNTPLFE KINCEGTIPR DLGVGEQITT VSAIDADELQ

**601** LVQYQIEAGN ELDFFSLNPN SGVLSLKRSL MDGLGAKVSF HSLRITATDG

**651** ENFATPLYIN ITVAASHKLV NLQCEETGVA KMLAEKLLQA NK**LHNQGEVE**

**701 DIFFDSHSVN AHIPQFR**STL PTGIQVKENQ PVGSSVIFMN STDLDTGFNG

**751** KLVYAVSGGN EDSCFMIDME TGMLKILSPL DRETTDKYTL NITVYDLGIP

**801** QKAAWRLLHV VVVDANDNPP EFLQESYFVE VSEDKEVHSE IIQVEATDKD

**851** LGPNGHVTYS IVTDTDTFSI DSVTGVVNIA RPLDRELQHE HSLKIEARDQ

**901** AREEPQLFST VVVKVSLEDV NDNPPTFIPP NYRVKVREDL PEGTVIMWLE

**951** AHDPDLGQSG QVRYSLLDHG EGNFDVDKLS GAVRIVQQLD FEKKQVYNLT

**1001** VRAKDKGKPV SLSSTCYVEV EVVDVNENLH PPVFSSFVEK GTVKEDAPVG

**1051** SLVMTVSAHD EDARRDGEIR YSIRDGSGVG VFKIGEETGV IETSDRLDRE

**1101** STSHYWLTVF ATDQGVVPLS SFIEIYIEVE DVNDNAPQTS EPVYYPEIME

**1151** NSPKDVSVVQ IEAFDPDSSS NDKLMYKITS GNPQGFFSIH PKTGLITTTS

**1201** RKLDREQQDE HILEVTVTDN GSPPKSTIAR VIVKILDEND NKPQFLQKFY

**1251** KIRLPEREKP DRERNARREP LYHVIATDKD EGPNAEISYS IEDGNEHGKF

**1301** FIEPKTGVVS SKRFSAAGEY DILSIKAVDN GRPQKSSTTR LHIEWISKPK

**1351** PSLEPISFEE SFFTFTVMES DPVAHMIGVI SVEPPGIPLW FDITGGNYDS

**1401** HFDVDKGTGT IIVAKPLDAE QKSNYNLTVE ATDGTTTILT QVFIKVIDTN

**1451** DHRPQFSTSK YEVVIPEDTA PETEILQISA VDQDEKNKLI YTLQSSRDPL

**1501** SLKKFRLDPA TGSLYTSEKL DHEAVHQHTL TVMVRDQDVP VKRNFARIVV

**1551** NVSDTNDHAP WFTASSYKGR VYESAAVGSV VLQVTALDKD KGKNAEVLYS

**1601** IESGTFGNIG NSFMIDPVLG SIKTAKELDR SNQAEYDLMV KATDKGSPPM

**1651** SEITSVRIFV TIADNASPKF TSKEYSVELS ETVSIGSFVG MVTAHSQSSV

**1701** VYEIKDGNTG DAFDINPHSG TIITQKALDF ETLPIYTLII QGTNMAGLST

**1751** NTTVLVHLQD ENDNAPVFMQ AEYTGLISES ASINSVVLTD RNVPLVIRAA

**1801** DADKDSNALL VYHIVEPSVH TYFAIDSSTG AIHTVLSLDY EETSIFHFTV

**1851** QVHDMGTPRL FAEYAANVTV HVIDINDCPP VFAKPLYEAS LLLPTYKGVK

**1901** VITVNATDAD SSAFSQLIYS ITEGNIGEKF SMDYKTGALT VQNTTQLRSR

**1951** YELTVRASDG RFAGLTSVKI NVKESKESHL KFTQDVYSAV VKENSTEAET

**2001** LAVITAIGNP INEPLFYHIL NPDRRFKISR TSGVLSTTGT PFDREQQEAF

**2051** DVVVEVTEEH KPSAVAHVVV KVIVEDQNDN APVFVNLPYY AVVKVDTEVG

**2101** HVIRYVTAVD RDSGRNGEVH YYLKEHHEHF QIGPLGEISL KKQFELDTLN

**2151** KEYLVTVVAK DGGNPAFSAE VIVPITVMNK AMPVFEKPFY SAEIAESIQV

**2201** HSPVVHVQAN SPEGLKVFYS ITDGDPFSQF TINFNTGVIN VIAPLDFEAH

**2251** PAYKLSIRAT DSLTGAHAEV FVDIIVDDIN DNPPVFAQQS YAVTLSEASV

**2301** IGTSVVQVRA TDSDSEPNRG ISYQMFGNHS KSHDHFHVDS STGLISLLRT

**2351** LDYEQSRQHT IFVRAVDGGM PTLSSDVIVT VDVTDLNDNP PLFEQQIYEA

**2401** RISEHAPHGH FVTCVKAYDA DSSDIDKLQY SILSGNDHK**H FVIDSATGII**

**2451 TLSNLHR**HAL KPFYSLNLSV SDGVFRSSTQ VHVTVIGGNL HSPAFLQNEY

**2501** EVELAENAPL HTLVMEVKTT DGDSGIYGHV TYHIVNDFAK DRFYINERGQ

**2551** IFTLEKLDRE TPAEKVISVR LMAKDAGGKV AFCTVNVILT DDNDNAPQFR

**2601** ATKYEVNIGS SAAKGTSVVK VLASDADEGS NADITYAIEA DSESVKENLE

**2651** INKLSGVITT KESLIGLENE FFTFFVRAVD NGSPSKESVV LVYVKILPPE

**2701** MQLPKFSEPF YTFTVSEDVP IGTEIDLIRA EHSGTVLYSL VKGNTPESNR

**2751** DESFVIDRQS GRLKLEKSLD HETTKWYQFS ILARCTQDDH EMVASVDVSI

**2801** QVKDANDNSP VFESSPYEAF IVENLPGGSR VIQIRASDAD SGTNGQVMYS

**2851** LDQSQSVEVI ESFAINMETG WITTLKELDH EKRDNYQIKV VASDHGEKIQ

**2901** LSSTAIVDVT VTDVNDSPPR FTAEIYKGTV SEDDPQGGVI AILSTTDADS

**2951** EEINRQVTYF ITGGDPLGQF AVETIQNEWK VYVKKPLDRE KRDNYLLTIT

**3001** ATDGTFSSKA IVEVKVLDAN DNSPVCEKTL YSDTIPEDVL PGKLIMQISA

**3051** TDADIRSNAE ITYTLLGSGA EKFKLNPDTG ELKTSTPLDR EEQAVYHLLV

**3101** RATDGGGRFC QASIVLTLED VNDNAPEFSA DPYAITVFEN TEPGTLLTRV

**3151** QATDADAGLN RKILYSLIDS ADGQFSINEL SGIIQLEKPL DRELQAVYTL

**3201** SLKAVDQGLP RRLTATGTVI VSVLDINDNP PVFEYREYGA TVSEDILVGT

**3251** EVLQVYAASR DIEANAEITY SIISGNEHGK FSIDSKTGAV FIIENLDYES

**3301** SHEYYLTVEA TDGGTPSLSD VATVNVNVTD INDNTPVFSQ DTYTTVISED

**3351** AVLEQSVITV MADDADGPSN SHIHYSIIDG NQGSSFTIDP VRGEVKVTKL

**3401** LDRETISGYT LTVQASDNGS PPRVNTTTVN IDVSDVNDNA PVFSRGNYSV

**3451** IIQENKPVGF SVLQLVVTDE DSSHNGPPFF FTIVTGNDEK **AFEVNPQGVL**

**3501 LTSSAIK**RKE KDHYLLQVKV ADNGKPQLSS LTYIDIRVIE ESIYPPAILP

**3551** LEIFITSSGE EYSGGVIGKI HATDQDVYDT LTYSLDPQMD NLFSVSSTGG

**3601** KLIAHKKLDI GQYLLNVSVT DGKFTTVADI TVHIRQVTQE MLNHTIAIRF

**3651** ANLTPEEFVG DYWRNFQRAL RNILGVRRND IQIVSLQSSE PHPHLDVLLF

**3701** VEKPGSAQIS TKQLLHKINS SVTDIEEIIG VRILNVFQKL CAGLDCPWKF

**3751** CDEKVSVDES VMSTHSTARL SFVTPRHHRA AVCLCKEGRC PPVHHGCEDD

**3801** PCPEGSECVS DPWEEKHTCV CPSGRFGQCP GSSSMTLTGN SYVKYRLTEN

**3851** ENKLEMKLTM RLRTYSTHAV VMYARGTDYS ILEIHHGRLQ YKFDCGSGPG

**3901** IVSVQSIQVN DGQWHAVALE VNGNYARLVL DQVHTASGTA PGTLKTLNLD

**3951** NYVFFGGHIR QQGTRHGRSP QVGNGFRGCM DSIYLNGQEL PLNSKPRSYA

**4001** HIEESVDVSP GCFLTATEDC ASNPCQNGGV CNPSPAGGYY CKCSALYIGT

**4051** HCEISVNPCS SKPCLYGGTC VVDNGGFVCQ CRGLYTGQRC QLSPYCKDEP

**4101** CKNGGTCFDS LDGAVCQCDS GFRGERCQSD IDECSGNPCL HGALCENTHG

**4151** SYHCNCSHEY RGRHCEDAAP NQYVSTPWNI GLAEGIGIVV FVAGIFLLVV

**4201** VFVLCRKMIS RKKKHQAEPK DKHLGPATAF LQRPYFDSKL NKNIYSDIPP

**4251** QVPVRPISYT PSIPSDSRNN LDRNSFEGSA IPEHPEFSTF NPESVHGHRK

**4301** AVAVCSVAPN LPPPPPSNSP SDSDSIQKPS WDFDYDTKVV DLDPCLSKKP

**4351** LEEKPSQPYS ARESLSEVQS LSSFQSESCD DNGYHWDTSD WMPSVPLPDI

**4401** QEFPNYEVID EQTPLYSADP NAIDTDYYPG GYDIESDFPP PPEDFPAADE

**4451** LPPLPPEFSN QFESIHPPRD MPAAGSLGSS SRNRQRFNLN QYLPNFYPLD

**4501** MSEPQTKGTG ENSTCREPHA PYPPGYQRHF EAPAVESMPM SVYASTASCS

**4551** DVSACCEVES EVMMSDYESG DDGHFEEVTI PPLDSQQHTE VJDGSQRLEQ

**4601** TPLQFTHLEY NVTVQENSAA KJGTVKEDAP VGSLVMTVSA HDEDAGRDGE

**4651** IRJGTVKEDA PVGSLVMTVS AHDEDARRDG EIRJLDREST SHYWLTVFAT

**4701** DQGVVPLSSF IEIYIEVEDV NDNAPQTSEP VYYPEIMENS PKDVSVVQIE

**4751** AFDPDSSSND KJLDRESTSH YWLTVFATDQ GVVPLSSFIE LYIEVEDVND

**4801** NAPQTSEPVY YPEIMENSPK DVSVVQIEAF DPDSSSNDKJ FYKIRLPERJ

**4851** REPLYHVIAT DKDEGPNAEI SYSIEDGNEH GKJREPLYRV IATDKDEGPN

**4901** AEISYSIEDG NEHGKJEPLY HVIATDKDEG LNAEISYSIE DGNEHGKFFI

**4951** EPKJEPLYHV IATDKDEGPN AEISYSIEDG NEHGKFFIEP KJALEKNTNL

**5001** EARTKJALEK NTNVEARTKJ ALEKNTNVEA RTKJALEKNT NVEVRTKJFS

**5051** AAGEYDILSI KAVDNGRPQK SSTTRJFSAA GEYDILSIKA VDSGRPQKSS

**5101** TTRJNFARIV VNVSDTNDHA PWFTASSYKG RJNFARIVVN VSDTNDHAPW

**5151** FTTSSYKGRJ GKNAEVLYSI ESGDFGNIGN SFMIDPVLGS IKTAKJGKNA

**5201** EVLYSIESGN FGNIGNSFMI DPVLGSIKTA KJGKNAEVLY SIESGTIGNI

**5251** GNSFMIDPVL GSIKTAKJGS PPMSEITSVR IFVTNADNAS PKFTSKJGSP

**5301** PMSEITSVRI FVTSADNASP KFTSKJGSPP MSEITSVRIF VTIADNASTK

**5351** FTSKJALDFE TLPIYTLIIQ GTNMAGLSTN TTVLVHLQDE NDNAPVFMQA

**5401** EYTGLISESA SINSVVLTDR NVPQVIRAAD ADKJALDFET LPIYTLIIQG

**5451** TNMAGLSTNT TVLVHLQDEN DNAPVFMQAE YTGLISESAS INSVVLTDRN

**5501** VPRVIRAADA DKJAADADKD SNVLLVYHIV EPSVHTYFAI DSSTGAIHTV

**5551** LSLDYEETSI FHFTVQVHDM GTPRLFAEYA ANVTVHVIDI NDCPPVFAKP

**5601** LYEASLLLPT YKJFTQDVYS AVVKTNSTEA ETLAVITAIG NPINEPLFYH

**5651** ILNPDRRJTS GVLSTTGTPF DREAQEAFDV VVEVTEEHKP SAVAHVVVKV

**5701** IVEDQNDNAP VFVNLPYYAV VKJTDMFAIH PTSGVIVLTG RLDFLETKLY

**5751** EMEILAADRJ TDMFAIHPTS GVIVLTGRLD YLETKLYEME ILAADRJNGE

**5801** VHYYLKEHHE HFQIGPLGEK SLKKJDGGNP AFSAEVIVPI TVMNKAMPVF

**5851** EKPFYSAEIA ESIQVHSHVV HVQANSPEGL KVFYSITDGD PFSQFTINFN

**5901** TGVINVIAPL DFEAHPAYKJ AMPVFEKPFY SAEIAESIQV HSPVVHVQAN

**5951** SPEGLKVFYS ITDGDPFSQF TINFNTGVIN VIAPLDFPAH PAYKLSIRJL

**6001** SIRATDSLTG AHAEVFVDDI VDDINDNPPV FAQQSYAVTL SEASVIGTSV

**6051** VQVRATDSDS EPNRJLSIRA TDSLTGAHAE VFVDEIVDDI NDNPPVFAQQ

**6101** SYAVTLSEAS VIGTSVVQVR ATDSDSEPNR JTLDYEQSRQ HTIAVRAVDG

**6151** GMPTLSSDVI VTVDVTDLND NPPLFEQQIY EARJLYGSSG ISSMAKLTVH

**6201** IEQANECAPV ITAVTLSPSE LDRDPAYAIV TVDDCDQGAN GDIASLSIVA

**6251** GDLLQQFRJI LPPEMQLPKF SEPFYTFTVS EIVPIGTEID LIRAEHSGTV

**6301** LYSLVKJILP PEMQLPKFSE PFYTFTVSEV VPIGTEIDLI RAEHSGTVLY

**6351** SLVKJCTQDD HEMVASVDVS IQVKDANDNS PVFESSPYEA FIVENLPGGS

**6401** RVIQIRJCTQ DDHEMVASVD VSIQVKDASD NSPVFESSPY EAFIVENLPG

**6451** GSRVIQIRJV IQIRASDADN GTNGQVMYSL DQSQSVEVIE SFAINMETGW

**6501** ITTLKELDHE KJFTAEIYKG TVSELDPQGG VIAILSTTDA DSEEINRQVT

**6551** YFITGGDPLG QFAVETIQNE WKJFTAEIYK GTVSEPDPQG GVIAILSTTD

**6601** ADSEEINRQV TYFITGGDPL GQFAVETIQN EWKJFTAEIY KGTVSEQDPQ

**6651** GGVIAILSTT DADSEEINRQ VTYFITGGDP LGQFAVETIQ NEWKJGTVSE

**6701** DDPQGGVIAI LSTTDADSEE INRQVTYGIT GGDPLGQFAV ETIQNEWKVY

**6751** VKJGTVSEDD PQGGVIAILS TTDADSEEIN RQVTYSITGG DPLGQFAVET

**6801** IQNEWKVYVK JGTVSEDDPQ GGVIAILSTT DADSEEINRQ VTYFITGGDP

**6851** LAQFAVETIQ NEWKVYVKJA IVEVKVLDAN DNSPVCEYTL YSDTIPEDVL

**6901** PGKLIMQISA TDADIRJVLD ANDNSPVCEK TLYIDTIPED VLPGKLIMQI

**6951** SATDADIRJV LDANDNSPVC EKTLYTDTIP EDVLPGKLIM QISATDADIR

**7001** JATDGGGRFC QASIVLDLED VNDNAPEFSA DPYAITVFEN TEPGTLLTRV

**7051** QATDADAGLN RJKILYSLID SADGQFSINE LIGIIQLEKP LDRELQAVYT

**7101** LSLKJFSIDS KTGAVFIIEN LDYESSHEYY LTVEATDGGT PSLSDVATVN

**7151** VNVTDINDAT PVFSQDTYTT VISEDAVLEQ SVITVMADDA DGPSNSHIHY

**7201** SIIDGNQGSS FTIDPVRGEV KJFSIDSKTG AVFIIENLDY ESSHEYYLTV

**7251** EATDGGTPSL SDVATVNVNV TDINDVTPVF SQDTYTTVIS EDAVLEQSVI

**7301** TVMADDADGP SNSHIHYSII DGNQGSSFTI DPVRGEVKJV NTTTVNIDVS

**7351** DVNDNAPVFS RGNYSVIIQE NKPVGFSVLQ LVVTDEDSSH NGPPFFFTIV

**7401** TENDEKAFEV NPQGVLLTSS AIKJVNTTTV NIDVSDVNDN APVFSRGNYS

**7451** VIIQENKPVG FSVLQLVVTD EDSSHNGPPF FFTIVTGNDE KAFEVNPQGV

**7501** LLTSSAIKJG NYSVIIQENK PVGFSVLQLV VTDEDSSHNG PPFFFTIVTG

**7551** NDEKAFEVNA QGVLLTSSAI KRJGNYSVII QENKPVGFSV LQLVVTDEDS

**7601** SHNGPPFFFT IVTGNDEKAF EVNVQGVLLT SSAIKRJVAD NGKPQLSSLT

**7651** YIDIRVIEES IYPPAILPLE IAITSSGEEY SGGVIGKIHA TDQDVYDTLT

**7701** YSLDPQMDNL FSVSSTGGKJ VADNGKPQLS SLTYIDIRVI EESIYPPAIL

**7751** PLEISITSSG EEYSGGVIGK IHATDQDVYD TLTYSLDPQM DNLFSVSSTG

**7801** GKJVIHVTSP QFKAGPVKFE KJQLLHKIMS SVTDIEEIIG VRILNVFQKJ

**7851** QLLHKIVSSV TDIEEIIGVR ILNVFQKJEG RCPPVHHGCE DHPCPEGSEC

**7901** VSDPWEEKHT CVCPSGRJEG RCPPVHHGCE DPPCPEGSEC VSDPWEEKHT

**7951** CVCPSGRJHT CVCPSGRFGG CPGSSSMTLT GNSYVKYRJH TCVCPSGRFG

**8001** RCPGSSSMTL TGNSYVKYRJ FGQCPGSSSM TLTGNSYVKT RLTENENKJG

**8051** CMDSIYLNGQ ELPLNSKPRS YAHIEESVPV SPGCFLTATE DCASNPCQNG

**8101** GVCNPSPAGG YYCKCSALYI GTHCEISVNP CSSKPCLYGG TCVVDNGGFV

**8151** CQCRJYVFKR TPGKAKJYVF KSTPGKAKJS YAHIEESVDV SPGCFLTATE

**8201** DCASNPCQNG GVCNPSPAGG YYCKCSALYI GTHCEISVNP KSSKPCLYGG

**8251** TCVVDNGGFV CQCRGLYTGQ RJSYAHIEES VDVSPGCFLT ATEDCASNPC

**8301** QNGGVCNPSP AGGYYCKCSA LYIGTHCEIS VNPNSSKPCL YGGTCVVDNG

**8351** GFVCQCRGLY TGQRJDEPCK NGGTCFDSAD GAVCQCDSGF RGERJGRHCA

**8401** DAAPNQYVST PWNIGLAEGI GIVVFVAGIF LLVVVFVLCR KJGRHCEDAA

**8451** PNQYVSTPWN IGLAEGIGIV VFVAGIFLLV VVFVLCRKJN IYSDIPPQVP

**8501** VRYISYTPSI PSDSRNNLDR JKAVAVCSVA PNLPPPPPSN SPSDSDSIQK

**8551** PSWDFDYDTK VVDLDPCLSK JKPLEEKPSQ PYSARESLSS VQSLSSFQSE

**8601** SCDDNGYHWD TSDWMPSVPL PDIQEFPNYE VIDEQTPLYS ADPNAIDTDY

**8651** YPGGYDIESD FPPPPEDFPA ADELPPLPPE FSNQFESIHP PRDMPAAGSL

**8701** GSSSRJKPLE EKPSQPYSAR ESLSEVQSLS SFQSESCDDN GYHWDTSDWM

**8751** PSVPLQDIQE FPNYEVIDEQ TPLYSADPNA IDTDYYPGGY DIESDFPPPP

**8801** EDFPAADELP PLPPEFSNQF ESIHPPRDMP AAGSLGSSSR JKPLEEKPSQ

**8851** PYSARESLSE VQSLSSFQSE SCDDNGYHWD TSDWMPSVPL PDIQEFPNYE

**8901** VIDPQTPLYS ADPNAIDTDY YPGGYDIESD FPPPPEDFPA ADELPPLPPE

**8951** FSNQFESIHP PRDMPAAGSL GSSSRJESLS EVQSLSSFQS ESCDDNGYHW

**9001** DTSDWMPSVP LPDIQEFPNY EVIDEQTPLY SADPNAIDTD YYPGGYDIES

**9051** DFPPPPEDFP AADELPPLPP EFSNQFESIH PPRDMPAAGS LGSSRRNRJE

**9101** SLSEVQSLSS FQSESCDDNG YHWDTSDWMP SVPLPDIQEF PNYEVIDEQT

**9151** PLYSADPNAI DTDYYPGGYD IESDFPPPPE DFPAADELPP LPPEFSNQFE

**9201** SIHPPRDMPA AGSLGSSWRN RJVLGANSNP PEFTQTAYKA AFDENVPIGT

**9251** TIMSLSAVDP DEGENGYVTY SIANLNHVPF AIDHFTGAVS TSENLDYELM

**9301** PRVYTLRJVL GANSNPPEFT QTAYKAAFDE NVPIGTTVMS LSAVDPDEGE

**9351** NGYVTYSIAN LNHVPFAIDH FTGAVSTSEN LDYELMPRVY TLRJVLGANS

**9401** NPPEFTQTAY KAAFDENVPI GTTVMSLSAV DPDEGENGYV TYSIANLNHV

**9451** PFAIDHFTGA VSTSENLDYE LMPRVYTLRJ INCEGTIPRD LGVGEQITTV

**9501** SAIDADELQL VQYQIEAGNE LDFFSLNPNS GVLSLKRJIN CEGTIPRDLG

**9551** VGEQITTVSA IDADELQLVQ YQIEAGNELD LFSLNPNSGV LSLKRJINCE

**9601** GTIPRDLGVG EQITTVSAID ADELQLVQYQ IEAGNELDFF SLNPNSGVLS

**9651** LKRJEVHSEI IQVEATDKDL GPNGHVTYSI LTDTDTFSID SVTGVVNIAR

**9701** PLDRELQHEH SLKJEVHSEI IQVEATDKDL GPNGHVTYSI VTDTDTFSID

**9751** SVTGVVNIAR PLDRELQHEH SLK

**Start - End Observed Mr(expt) Mr(calc) ppm Miss Sequence**

**85 - 97 816.8916 1631.7686 1631.7654 2 0 K.AEEYILGDFCFLR.I**  ([Ions score 62](http://mbp-mascot4/mascot/cgi/peptide_view.pl?file=../data/20091021/F006293.dat&query=3884&hit=1&index=IPI00031411&px=1&section=5&ave_thresh=22))

**453 - 469 918.9631 1835.9117 1835.9054 3 0 K.VLGANSNPPEFTQTAYK.A**  ([Ions score 35](http://mbp-mascot4/mascot/cgi/peptide_view.pl?file=../data/20091021/F006293.dat&query=4600&hit=1&index=IPI00031411&px=1&section=5&ave_thresh=22))

**453 - 469 918.9631 1835.9117 1835.9054 3 0 K.VLGANSNPPEFTQTAYK.A**  ([Ions score 33](http://mbp-mascot4/mascot/cgi/peptide_view.pl?file=../data/20091021/F006293.dat&query=4601&hit=1&index=IPI00031411&px=1&section=5&ave_thresh=22))

**693 - 717 734.8584 2935.4045 2935.4002 1 0 K.LHNQGEVEDIFFDSHSVNAHIPQFR.S**  ([Ions score 32](http://mbp-mascot4/mascot/cgi/peptide_view.pl?file=../data/20091021/F006293.dat&query=6714&hit=1&index=IPI00031411&px=1&section=5&ave_thresh=22))

**2440 - 2457 665.3679 1993.0819 1993.0745 4 0 K.HFVIDSATGIITLSNLHR.H**  ([Ions score 40](http://mbp-mascot4/mascot/cgi/peptide_view.pl?file=../data/20091021/F006293.dat&query=5053&hit=1&index=IPI00031411&px=1&section=5&ave_thresh=22))

**2440 - 2457 665.3680 1993.0823 1993.0745 4 0 K.HFVIDSATGIITLSNLHR.H**  ([Ions score 32](http://mbp-mascot4/mascot/cgi/peptide_view.pl?file=../data/20091021/F006293.dat&query=5054&hit=1&index=IPI00031411&px=1&section=5&ave_thresh=22))

**3491 - 3507 887.4932 1772.9719 1772.9672 3 0 K.AFEVNPQGVLLTSSAIK.R**  ([Ions score 77](http://mbp-mascot4/mascot/cgi/peptide_view.pl?file=../data/20091021/F006293.dat&query=4398&hit=1&index=IPI00031411&px=1&section=5&ave_thresh=22))

**HPDE A3:**

Match to: **IPI00031411** Score: **395**

**Gene_Symbol=FAT1 Protocadherin Fat 1 lng=4591 # SP[4593,D,22,D]SNP[4646,g,1064,R]SNP[4677,r,1064,R]SNP[4713,i,1125,I]SNP[4791,l,1125,I]SNP[4844,i,1252,I]SNP[4856,h,1273,H]SNP[4889,r,1273,H]SNP[4931,l,1283,P]SNP[4969,p,1283,P]SNP[5000,l,129,V]SNP[5**

Found in search of C:\mgf\Orbidata\080723_ISW1295_HPDE_A3.mgf

Nominal mass (M_r_): **1068827**; Calculated pI value: **4.55**

NCBI BLAST search of [IPI00031411](http://www.ncbi.nlm.nih.gov/blast/Blast.cgi?ALIGNMENTS=50&ALIGNMENT_VIEW=Pairwise&AUTO_FORMAT=Semiauto&CDD_SEARCH=on&CLIENT=web&COMPOSITION_BASED_STATISTICS=on&DATABASE=nr&DESCRIPTIONS=100&ENTREZ_QUERY=(none)&EXPECT=10&FILTER=L&FORMAT_BLOCK_ON_RESPAGE=None&FORMAT_OBJECT=Alignment&FORMAT_TYPE=HTML&GAPCOSTS=11+1&I_THRESH=0.001&LAYOUT=TwoWindows&MATRIX_NAME=BLOSUM62&NCBI_GI=on&PAGE=Proteins&PROGRAM=blastp&QUERY=IPI00031411&SERVICE=plain&SET_DEFAULTS.x=21&SET_DEFAULTS.y=7&SHOW_OVERVIEW=on&WORD_SIZE=3&END_OF_HTTPGET=Yes) against nr

Unformatted [sequence string](http://mbp-mascot4/mascot/cgi/getseq.pl?IPI_human+IPI00031411+seq) for pasting into other applications

Fixed modifications: Carbamidomethyl (C)

Variable modifications: Oxidation (M)

Cleavage by TrypsinMSIPI, a mixture of enzymes:

cuts C-term side of KR unless next residue is P

cuts C-term side of J

cuts N-term side of J

Sequence Coverage: **1%**

Matched peptides shown in **Bold Red**

**1** MGRHLALLLL LLLLFQHFGD SDGSQRLEQT PLQFTHLEYN VTVQENSAAK

**51** TYVGHPVKMG VYITHPAWEV RYKIVSGDSE NLFKAEEYIL GDFCFLRIRT

**101** KGGNTAILNR EVKDHYTLIV KALEKNTNVE ARTKVRVQVL DTNDLRPLFS

**151** PTSYSVSLPE NTAIRTSIAR VSATDADIGT NGEFYYSFKD RTDMFAIHPT

**201** SGVIVLTGRL DYLETKLYEM EILAADRGMK LYGSSGISSM AKLTVHIEQA

**251** NECAPVITAV TLSPSELDRD PAYAIVTVDD CDQGANGDIA SLSIVAGDLL

**301** QQFRTVRSFP GSKEYKVKAI GGIDWDSHPF GYNLTLQAKD KGTPPQFSSV

**351** KVIHVTSPQF KAGPVKFEKD VYR**AEISEFA PPNTPVVMVK** AIPAYSHLRY

**401** VFKSTPGKAK FSLNYNTGLI SILEPVKRQQ AAHFELEVTT SDRKASTKVL

**451** VKVLGANSNP PEFTQTAYKA AFDENVPIGT TVMSLSAVDP DEGENGYVTY

**501** SIANLNHVPF AIDHFTGAVS TSENLDYELM PRVYTLRIRA SDWGLPYRRE

**551** VEVLATITLN NLNDNTPLFE KINCEGTIPR DLGVGEQITT VSAIDADELQ

**601** LVQYQIEAGN ELDFFSLNPN SGVLSLKRSL MDGLGAKVSF HSLRITATDG

**651** ENFATPLYIN ITVAASHKLV NLQCEETGVA KMLAEKLLQA NKLHNQGEVE

**701** DIFFDSHSVN AHIPQFRSTL PTGIQVKENQ PVGSSVIFMN STDLDTGFNG

**751** KLVYAVSGGN EDSCFMIDME TGMLKILSPL DRETTDKYTL NITVYDLGIP

**801** QKAAWRLLHV VVVDANDNPP EFLQESYFVE VSEDKEVHSE IIQVEATDKD

**851** LGPNGHVTYS IVTDTDTFSI DSVTGVVNIA RPLDRELQHE HSLKIEARDQ

**901** AR**EEPQLFST VVVK**VSLEDV NDNPPTFIPP NYRVKVREDL PEGTVIMWLE

**951** AHDPDLGQSG QVRYSLLDHG EGNFDVDKLS GAVRIVQQLD FEKKQVYNLT

**1001** VRAKDKGKPV SLSSTCYVEV EVVDVNENLH PPVFSSFVEK GTVKEDAPVG

**1051** SLVMTVSAHD EDARRDGEIR YSIRDGSGVG VFKIGEETGV IETSDRLDRE

**1101** STSHYWLTVF ATDQGVVPLS SFIEIYIEVE DVNDNAPQTS EPVYYPEIME

**1151** NSPKDVSVVQ IEAFDPDSSS NDKLMYKITS GNPQGFFSIH PKTGLITTTS

**1201** RKLDREQQDE HILEVTVTDN GSPPKSTIAR VIVKILDEND NKPQFLQKFY

**1251** KIRLPEREKP DRERNARREP LYHVIATDKD EGPNAEISYS IEDGNEHGKF

**1301** FIEPKTGVVS SKRFSAAGEY DILSIKAVDN GRPQKSSTTR LHIEWISKPK

**1351** PSLEPISFEE SFFTFTVMES DPVAHMIGVI SVEPPGIPLW FDITGGNYDS

**1401** HFDVDKGTGT IIVAKPLDAE QKSNYNLTVE ATDGTTTILT QVFIKVIDTN

**1451** DHRPQFSTSK YEVVIPEDTA PETEILQISA VDQDEKNKLI YTLQSSRDPL

**1501** SLKKFRLDPA TGSLYTSEKL DHEAVHQHTL TVMVRDQDVP VKRNFARIVV

**1551** NVSDTNDHAP WFTASSYKGR VYESAAVGSV VLQVTALDKD KGKNAEVLYS

**1601** IESGTFGNIG NSFMIDPVLG SIKTAKELDR SNQAEYDLMV KATDKGSPPM

**1651** SEITSVRIFV TIADNASPKF TSKEYSVELS ETVSIGSFVG MVTAHSQSSV

**1701** VYEIK**DGNTG DAFDINPHSG TIITQK**ALDF ETLPIYTLII QGTNMAGLST

**1751** NTTVLVHLQD ENDNAPVFMQ AEYTGLISES ASINSVVLTD RNVPLVIRAA

**1801** DADKDSNALL VYHIVEPSVH TYFAIDSSTG AIHTVLSLDY EETSIFHFTV

**1851** QVHDMGTPRL FAEYAANVTV HVIDINDCPP VFAKPLYEAS LLLPTYKGVK

**1901** VITVNATDAD SSAFSQLIYS ITEGNIGEKF SMDYKTGALT VQNTTQLRSR

**1951** YELTVRASDG RFAGLTSVKI NVKESKESHL KFTQDVYSAV VKENSTEAET

**2001** LAVITAIGNP INEPLFYHIL NPDRRFKISR TSGVLSTTGT PFDREQQEAF

**2051** DVVVEVTEEH KPSAVAHVVV K**VIVEDQNDN APVFVNLPYY AVVK**VDTEVG

**2101** HVIRYVTAVD RDSGRNGEVH YYLK**EHHEHF QIGPLGEISL K**KQFELDTLN

**2151** KEYLVTVVAK DGGNPAFSAE VIVPITVMNK AMPVFEKPFY SAEIAESIQV

**2201** HSPVVHVQAN SPEGLKVFYS ITDGDPFSQF TINFNTGVIN VIAPLDFEAH

**2251** PAYKLSIRAT DSLTGAHAEV FVDIIVDDIN DNPPVFAQQS YAVTLSEASV

**2301** IGTSVVQVRA TDSDSEPNRG ISYQMFGNHS KSHDHFHVDS STGLISLLRT

**2351** LDYEQSRQHT IFVRAVDGGM PTLSSDVIVT VDVTDLNDNP PLFEQQIYEA

**2401** RISEHAPHGH FVTCVKAYDA DSSDIDKLQY SILSGNDHKH FVIDSATGII

**2451** TLSNLHRHAL KPFYSLNLSV SDGVFRSSTQ VHVTVIGGNL HSPAFLQNEY

**2501** EVELAENAPL HTLVMEVK**TT DGDSGIYGHV TYHIVNDFAK** DRFYINERGQ

**2551** IFTLEKLDRE TPAEKVISVR LMAKDAGGKV AFCTVNVILT DDNDNAPQFR

**2601** ATKYEVNIGS SAAKGTSVVK VLASDADEGS NADITYAIEA DSESVKENLE

**2651** INKLSGVITT KESLIGLENE FFTFFVRAVD NGSPSKESVV LVYVKILPPE

**2701** MQLPKFSEPF YTFTVSEDVP IGTEIDLIRA EHSGTVLYSL VKGNTPESNR

**2751** DESFVIDRQS GRLKLEKSLD HETTKWYQFS ILARCTQDDH EMVASVDVSI

**2801** QVKDANDNSP VFESSPYEAF IVENLPGGSR VIQIRASDAD SGTNGQVMYS

**2851** LDQSQSVEVI ESFAINMETG WITTLKELDH EKRDNYQIKV VASDHGEKIQ

**2901** LSSTAIVDVT VTDVNDSPPR FTAEIYKGTV SEDDPQGGVI AILSTTDADS

**2951** EEINRQVTYF ITGGDPLGQF AVETIQNEWK VYVKKPLDRE KRDNYLLTIT

**3001** ATDGTFSSKA IVEVKVLDAN DNSPVCEK**TL YSDTIPEDVL PGK**LIMQISA

**3051** TDADIRSNAE ITYTLLGSGA EKFKLNPDTG ELK**TSTPLDR EEQAVYHLLV**

**3101 R**ATDGGGRFC QASIVLTLED VNDNAPEFSA DPYAITVFEN TEPGTLLTRV

**3151** QATDADAGLN RKILYSLIDS ADGQFSINEL SGIIQLEKPL DRELQAVYTL

**3201** SLKAVDQGLP RRLTATGTVI VSVLDINDNP PVFEYREYGA TVSEDILVGT

**3251** EVLQVYAASR DIEANAEITY SIISGNEHGK FSIDSKTGAV FIIENLDYES

**3301** SHEYYLTVEA TDGGTPSLSD VATVNVNVTD INDNTPVFSQ DTYTTVISED

**3351** AVLEQSVITV MADDADGPSN SHIHYSIIDG NQGSSFTIDP VRGEVKVTKL

**3401** LDRETISGYT LTVQASDNGS PPRVNTTTVN IDVSDVNDNA PVFSRGNYSV

**3451** IIQENKPVGF SVLQLVVTDE DSSHNGPPFF FTIVTGNDEK AFEVNPQGVL

**3501** LTSSAIKRKE KDHYLLQVKV ADNGKPQLSS LTYIDIRVIE ESIYPPAILP

**3551** LEIFITSSGE EYSGGVIGK**I HATDQDVYDT LTYSLDPQMD NLFSVSSTGG**

**3601 K**LIAHKKLDI GQYLLNVSVT DGKFTTVADI TVHIRQVTQE MLNHTIAIR**F**

**3651 ANLTPEEFVG DYWR**NFQRAL RNILGVRRND IQIVSLQSSE PHPHLDVLLF

**3701** VEKPGSAQIS TKQLLHKINS SVTDIEEIIG VRILNVFQKL CAGLDCPWKF

**3751** CDEKVSVDES VMSTHSTARL SFVTPRHHRA AVCLCKEGRC PPVHHGCEDD

**3801** PCPEGSECVS DPWEEKHTCV CPSGRFGQCP GSSSMTLTGN SYVKYRLTEN

**3851** ENKLEMKLTM RLRTYSTHAV VMYARGTDYS ILEIHHGRLQ YKFDCGSGPG

**3901** IVSVQSIQVN DGQWHAVALE VNGNYARLVL DQVHTASGTA PGTLKTLNLD

**3951** NYVFFGGHIR QQGTRHGRSP QVGNGFRGCM DSIYLNGQEL PLNSKPRSYA

**4001** HIEESVDVSP GCFLTATEDC ASNPCQNGGV CNPSPAGGYY CKCSALYIGT

**4051** HCEISVNPCS SKPCLYGGTC VVDNGGFVCQ CRGLYTGQRC QLSPYCKDEP

**4101** CKNGGTCFDS LDGAVCQCDS GFRGERCQSD IDECSGNPCL HGALCENTHG

**4151** SYHCNCSHEY RGRHCEDAAP NQYVSTPWNI GLAEGIGIVV FVAGIFLLVV

**4201** VFVLCRKMIS RKKKHQAEPK DKHLGPATAF LQRPYFDSKL NKNIYSDIPP

**4251** QVPVRPISYT PSIPSDSRNN LDRNSFEGSA IPEHPEFSTF NPESVHGHRK

**4301** AVAVCSVAPN LPPPPPSNSP SDSDSIQKPS WDFDYDTKVV DLDPCLSKKP

**4351** LEEKPSQPYS ARESLSEVQS LSSFQSESCD DNGYHWDTSD WMPSVPLPDI

**4401** QEFPNYEVID EQTPLYSADP NAIDTDYYPG GYDIESDFPP PPEDFPAADE

**4451** LPPLPPEFSN QFESIHPPRD MPAAGSLGSS SRNRQRFNLN QYLPNFYPLD

**4501** MSEPQTKGTG ENSTCREPHA PYPPGYQRHF EAPAVESMPM SVYASTASCS

**4551** DVSACCEVES EVMMSDYESG DDGHFEEVTI PPLDSQQHTE VJDGSQRLEQ

**4601** TPLQFTHLEY NVTVQENSAA KJGTVKEDAP VGSLVMTVSA HDEDAGRDGE

**4651** IRJGTVKEDA PVGSLVMTVS AHDEDARRDG EIRJLDREST SHYWLTVFAT

**4701** DQGVVPLSSF IEIYIEVEDV NDNAPQTSEP VYYPEIMENS PKDVSVVQIE

**4751** AFDPDSSSND KJLDRESTSH YWLTVFATDQ GVVPLSSFIE LYIEVEDVND

**4801** NAPQTSEPVY YPEIMENSPK DVSVVQIEAF DPDSSSNDKJ FYKIRLPERJ

**4851** REPLYHVIAT DKDEGPNAEI SYSIEDGNEH GKJREPLYRV IATDKDEGPN

**4901** AEISYSIEDG NEHGKJEPLY HVIATDKDEG LNAEISYSIE DGNEHGKFFI

**4951** EPKJEPLYHV IATDKDEGPN AEISYSIEDG NEHGKFFIEP KJALEKNTNL

**5001** EARTKJALEK NTNVEARTKJ ALEKNTNVEA RTKJALEKNT NVEVRTKJFS

**5051** AAGEYDILSI KAVDNGRPQK SSTTRJFSAA GEYDILSIKA VDSGRPQKSS

**5101** TTRJNFARIV VNVSDTNDHA PWFTASSYKG RJNFARIVVN VSDTNDHAPW

**5151** FTTSSYKGRJ GKNAEVLYSI ESGDFGNIGN SFMIDPVLGS IKTAKJGKNA

**5201** EVLYSIESGN FGNIGNSFMI DPVLGSIKTA KJGKNAEVLY SIESGTIGNI

**5251** GNSFMIDPVL GSIKTAKJGS PPMSEITSVR IFVTNADNAS PKFTSKJGSP

**5301** PMSEITSVRI FVTSADNASP KFTSKJGSPP MSEITSVRIF VTIADNASTK

**5351** FTSKJALDFE TLPIYTLIIQ GTNMAGLSTN TTVLVHLQDE NDNAPVFMQA

**5401** EYTGLISESA SINSVVLTDR NVPQVIRAAD ADKJALDFET LPIYTLIIQG

**5451** TNMAGLSTNT TVLVHLQDEN DNAPVFMQAE YTGLISESAS INSVVLTDRN

**5501** VPRVIRAADA DKJAADADKD SNVLLVYHIV EPSVHTYFAI DSSTGAIHTV

**5551** LSLDYEETSI FHFTVQVHDM GTPRLFAEYA ANVTVHVIDI NDCPPVFAKP

**5601** LYEASLLLPT YKJFTQDVYS AVVKTNSTEA ETLAVITAIG NPINEPLFYH

**5651** ILNPDRRJTS GVLSTTGTPF DREAQEAFDV VVEVTEEHKP SAVAHVVVKV

**5701** IVEDQNDNAP VFVNLPYYAV VKJTDMFAIH PTSGVIVLTG RLDFLETKLY

**5751** EMEILAADRJ TDMFAIHPTS GVIVLTGRLD YLETKLYEME ILAADRJNGE

**5801** VHYYLKEHHE HFQIGPLGEK SLKKJDGGNP AFSAEVIVPI TVMNKAMPVF

**5851** EKPFYSAEIA ESIQVHSHVV HVQANSPEGL KVFYSITDGD PFSQFTINFN

**5901** TGVINVIAPL DFEAHPAYKJ AMPVFEKPFY SAEIAESIQV HSPVVHVQAN

**5951** SPEGLKVFYS ITDGDPFSQF TINFNTGVIN VIAPLDFPAH PAYKLSIRJL

**6001** SIRATDSLTG AHAEVFVDDI VDDINDNPPV FAQQSYAVTL SEASVIGTSV

**6051** VQVRATDSDS EPNRJLSIRA TDSLTGAHAE VFVDEIVDDI NDNPPVFAQQ

**6101** SYAVTLSEAS VIGTSVVQVR ATDSDSEPNR JTLDYEQSRQ HTIAVRAVDG

**6151** GMPTLSSDVI VTVDVTDLND NPPLFEQQIY EARJLYGSSG ISSMAKLTVH

**6201** IEQANECAPV ITAVTLSPSE LDRDPAYAIV TVDDCDQGAN GDIASLSIVA

**6251** GDLLQQFRJI LPPEMQLPKF SEPFYTFTVS EIVPIGTEID LIRAEHSGTV

**6301** LYSLVKJILP PEMQLPKFSE PFYTFTVSEV VPIGTEIDLI RAEHSGTVLY

**6351** SLVKJCTQDD HEMVASVDVS IQVKDANDNS PVFESSPYEA FIVENLPGGS

**6401** RVIQIRJCTQ DDHEMVASVD VSIQVKDASD NSPVFESSPY EAFIVENLPG

**6451** GSRVIQIRJV IQIRASDADN GTNGQVMYSL DQSQSVEVIE SFAINMETGW

**6501** ITTLKELDHE KJFTAEIYKG TVSELDPQGG VIAILSTTDA DSEEINRQVT

**6551** YFITGGDPLG QFAVETIQNE WKJFTAEIYK GTVSEPDPQG GVIAILSTTD

**6601** ADSEEINRQV TYFITGGDPL GQFAVETIQN EWKJFTAEIY KGTVSEQDPQ

**6651** GGVIAILSTT DADSEEINRQ VTYFITGGDP LGQFAVETIQ NEWKJGTVSE

**6701** DDPQGGVIAI LSTTDADSEE INRQVTYGIT GGDPLGQFAV ETIQNEWKVY

**6751** VKJGTVSEDD PQGGVIAILS TTDADSEEIN RQVTYSITGG DPLGQFAVET

**6801** IQNEWKVYVK JGTVSEDDPQ GGVIAILSTT DADSEEINRQ VTYFITGGDP

**6851** LAQFAVETIQ NEWKVYVKJA IVEVKVLDAN DNSPVCEYTL YSDTIPEDVL

**6901** PGKLIMQISA TDADIRJVLD ANDNSPVCEK TLYIDTIPED VLPGKLIMQI

**6951** SATDADIRJV LDANDNSPVC EKTLYTDTIP EDVLPGKLIM QISATDADIR

**7001** JATDGGGRFC QASIVLDLED VNDNAPEFSA DPYAITVFEN TEPGTLLTRV

**7051** QATDADAGLN RJKILYSLID SADGQFSINE LIGIIQLEKP LDRELQAVYT

**7101** LSLKJFSIDS KTGAVFIIEN LDYESSHEYY LTVEATDGGT PSLSDVATVN

**7151** VNVTDINDAT PVFSQDTYTT VISEDAVLEQ SVITVMADDA DGPSNSHIHY

**7201** SIIDGNQGSS FTIDPVRGEV KJFSIDSKTG AVFIIENLDY ESSHEYYLTV

**7251** EATDGGTPSL SDVATVNVNV TDINDVTPVF SQDTYTTVIS EDAVLEQSVI

**7301** TVMADDADGP SNSHIHYSII DGNQGSSFTI DPVRGEVKJV NTTTVNIDVS

**7351** DVNDNAPVFS RGNYSVIIQE NKPVGFSVLQ LVVTDEDSSH NGPPFFFTIV

**7401** TENDEKAFEV NPQGVLLTSS AIKJVNTTTV NIDVSDVNDN APVFSRGNYS

**7451** VIIQENKPVG FSVLQLVVTD EDSSHNGPPF FFTIVTGNDE KAFEVNPQGV

**7501** LLTSSAIKJG NYSVIIQENK PVGFSVLQLV VTDEDSSHNG PPFFFTIVTG

**7551** NDEKAFEVNA QGVLLTSSAI KRJGNYSVII QENKPVGFSV LQLVVTDEDS

**7601** SHNGPPFFFT IVTGNDEKAF EVNVQGVLLT SSAIKRJVAD NGKPQLSSLT

**7651** YIDIRVIEES IYPPAILPLE IAITSSGEEY SGGVIGKIHA TDQDVYDTLT

**7701** YSLDPQMDNL FSVSSTGGKJ VADNGKPQLS SLTYIDIRVI EESIYPPAIL

**7751** PLEISITSSG EEYSGGVIGK IHATDQDVYD TLTYSLDPQM DNLFSVSSTG

**7801** GKJVIHVTSP QFKAGPVKFE KJQLLHKIMS SVTDIEEIIG VRILNVFQKJ

**7851** QLLHKIVSSV TDIEEIIGVR ILNVFQKJEG RCPPVHHGCE DHPCPEGSEC

**7901** VSDPWEEKHT CVCPSGRJEG RCPPVHHGCE DPPCPEGSEC VSDPWEEKHT

**7951** CVCPSGRJHT CVCPSGRFGG CPGSSSMTLT GNSYVKYRJH TCVCPSGRFG

**8001** RCPGSSSMTL TGNSYVKYRJ FGQCPGSSSM TLTGNSYVKT RLTENENKJG

**8051** CMDSIYLNGQ ELPLNSKPRS YAHIEESVPV SPGCFLTATE DCASNPCQNG

**8101** GVCNPSPAGG YYCKCSALYI GTHCEISVNP CSSKPCLYGG TCVVDNGGFV

**8151** CQCRJYVFKR TPGKAKJYVF KSTPGKAKJS YAHIEESVDV SPGCFLTATE

**8201** DCASNPCQNG GVCNPSPAGG YYCKCSALYI GTHCEISVNP KSSKPCLYGG

**8251** TCVVDNGGFV CQCRGLYTGQ RJSYAHIEES VDVSPGCFLT ATEDCASNPC

**8301** QNGGVCNPSP AGGYYCKCSA LYIGTHCEIS VNPNSSKPCL YGGTCVVDNG

**8351** GFVCQCRGLY TGQRJDEPCK NGGTCFDSAD GAVCQCDSGF RGERJGRHCA

**8401** DAAPNQYVST PWNIGLAEGI GIVVFVAGIF LLVVVFVLCR KJGRHCEDAA

**8451** PNQYVSTPWN IGLAEGIGIV VFVAGIFLLV VVFVLCRKJN IYSDIPPQVP

**8501** VRYISYTPSI PSDSRNNLDR JKAVAVCSVA PNLPPPPPSN SPSDSDSIQK

**8551** PSWDFDYDTK VVDLDPCLSK JKPLEEKPSQ PYSARESLSS VQSLSSFQSE

**8601** SCDDNGYHWD TSDWMPSVPL PDIQEFPNYE VIDEQTPLYS ADPNAIDTDY

**8651** YPGGYDIESD FPPPPEDFPA ADELPPLPPE FSNQFESIHP PRDMPAAGSL

**8701** GSSSRJKPLE EKPSQPYSAR ESLSEVQSLS SFQSESCDDN GYHWDTSDWM

**8751** PSVPLQDIQE FPNYEVIDEQ TPLYSADPNA IDTDYYPGGY DIESDFPPPP

**8801** EDFPAADELP PLPPEFSNQF ESIHPPRDMP AAGSLGSSSR JKPLEEKPSQ

**8851** PYSARESLSE VQSLSSFQSE SCDDNGYHWD TSDWMPSVPL PDIQEFPNYE

**8901** VIDPQTPLYS ADPNAIDTDY YPGGYDIESD FPPPPEDFPA ADELPPLPPE

**8951** FSNQFESIHP PRDMPAAGSL GSSSRJESLS EVQSLSSFQS ESCDDNGYHW

**9001** DTSDWMPSVP LPDIQEFPNY EVIDEQTPLY SADPNAIDTD YYPGGYDIES

**9051** DFPPPPEDFP AADELPPLPP EFSNQFESIH PPRDMPAAGS LGSSRRNRJE

**9101** SLSEVQSLSS FQSESCDDNG YHWDTSDWMP SVPLPDIQEF PNYEVIDEQT

**9151** PLYSADPNAI DTDYYPGGYD IESDFPPPPE DFPAADELPP LPPEFSNQFE

**9201** SIHPPRDMPA AGSLGSSWRN RJVLGANSNP PEFTQTAYKA AFDENVPIGT

**9251** TIMSLSAVDP DEGENGYVTY SIANLNHVPF AIDHFTGAVS TSENLDYELM

**9301** PRVYTLRJVL GANSNPPEFT QTAYKAAFDE NVPIGTTVMS LSAVDPDEGE

**9351** NGYVTYSIAN LNHVPFAIDH FTGAVSTSEN LDYELMPRVY TLRJVLGANS

**9401** NPPEFTQTAY KAAFDENVPI GTTVMSLSAV DPDEGENGYV TYSIANLNHV

**9451** PFAIDHFTGA VSTSENLDYE LMPRVYTLRJ INCEGTIPRD LGVGEQITTV

**9501** SAIDADELQL VQYQIEAGNE LDFFSLNPNS GVLSLKRJIN CEGTIPRDLG

**9551** VGEQITTVSA IDADELQLVQ YQIEAGNELD LFSLNPNSGV LSLKRJINCE

**9601** GTIPRDLGVG EQITTVSAID ADELQLVQYQ IEAGNELDFF SLNPNSGVLS

**9651** LKRJEVHSEI IQVEATDKDL GPNGHVTYSI LTDTDTFSID SVTGVVNIAR

**9701** PLDRELQHEH SLKJEVHSEI IQVEATDKDL GPNGHVTYSI VTDTDTFSID

**9751** SVTGVVNIAR PLDRELQHEH SLK

**Start - End Observed Mr(expt) Mr(calc) ppm Miss Sequence**

**374 - 390 914.9833 1827.9520 1827.9441 4 0 R.AEISEFAPPNTPVVMVK.A**  ([Ions score 39](http://mbp-mascot4/mascot/cgi/peptide_view.pl?file=../data/20091021/F006294.dat&query=4293&hit=1&index=IPI00031411&px=1&section=5&ave_thresh=21))

**903 - 914 688.3772 1374.7398 1374.7395 0 0 R.EEPQLFSTVVVK.V**  ([Ions score 24](http://mbp-mascot4/mascot/cgi/peptide_view.pl?file=../data/20091021/F006294.dat&query=2441&hit=1&index=IPI00031411&px=1&section=5&ave_thresh=21))

**903 - 914 688.3776 1374.7406 1374.7395 1 0 R.EEPQLFSTVVVK.V**  ([Ions score 21](http://mbp-mascot4/mascot/cgi/peptide_view.pl?file=../data/20091021/F006294.dat&query=2442&hit=1&index=IPI00031411&px=1&section=5&ave_thresh=21))

**1706 - 1726 734.3543 2200.0411 2200.0397 1 0 K.DGNTGDAFDINPHSGTIITQK.A**  ([Ions score 55](http://mbp-mascot4/mascot/cgi/peptide_view.pl?file=../data/20091021/F006294.dat&query=5198&hit=1&index=IPI00031411&px=1&section=5&ave_thresh=21))

**1706 - 1726 734.3545 2200.0418 2200.0397 1 0 K.DGNTGDAFDINPHSGTIITQK.A**  ([Ions score 57](http://mbp-mascot4/mascot/cgi/peptide_view.pl?file=../data/20091021/F006294.dat&query=5199&hit=1&index=IPI00031411&px=1&section=5&ave_thresh=21))

**2072 - 2094 869.4542 2605.3408 2605.3428 -1 0 K.VIVEDQNDNAPVFVNLPYYAVVK.V**  ([Ions score 45](http://mbp-mascot4/mascot/cgi/peptide_view.pl?file=../data/20091021/F006294.dat&query=5988&hit=1&index=IPI00031411&px=1&section=5&ave_thresh=21))

**2072 - 2094 869.4561 2605.3465 2605.3428 1 0 K.VIVEDQNDNAPVFVNLPYYAVVK.V**  ([Ions score 52](http://mbp-mascot4/mascot/cgi/peptide_view.pl?file=../data/20091021/F006294.dat&query=5989&hit=1&index=IPI00031411&px=1&section=5&ave_thresh=21))

**2125 - 2141 493.5072 1969.9997 1970.0010 -1 0 K.EHHEHFQIGPLGEISLK.K**  ([Ions score 43](http://mbp-mascot4/mascot/cgi/peptide_view.pl?file=../data/20091021/F006294.dat&query=4754&hit=1&index=IPI00031411&px=1&section=5&ave_thresh=21))

**2125 - 2141 493.5078 1970.0020 1970.0010 1 0 K.EHHEHFQIGPLGEISLK.K**  ([Ions score 22](http://mbp-mascot4/mascot/cgi/peptide_view.pl?file=../data/20091021/F006294.dat&query=4756&hit=1&index=IPI00031411&px=1&section=5&ave_thresh=21))

**2519 - 2540 804.0488 2409.1245 2409.1237 0 0 K.TTDGDSGIYGHVTYHIVNDFAK.D**  ([Ions score 23](http://mbp-mascot4/mascot/cgi/peptide_view.pl?file=../data/20091021/F006294.dat&query=5730&hit=1&index=IPI00031411&px=1&section=5&ave_thresh=21))

**3029 - 3043 824.4282 1646.8419 1646.8403 1 0 K.TLYSDTIPEDVLPGK.L**  ([Ions score 65](http://mbp-mascot4/mascot/cgi/peptide_view.pl?file=../data/20091021/F006294.dat&query=3581&hit=1&index=IPI00031411&px=1&section=5&ave_thresh=21))

**3029 - 3043 824.4291 1646.8437 1646.8403 2 0 K.TLYSDTIPEDVLPGK.L**  ([Ions score 61](http://mbp-mascot4/mascot/cgi/peptide_view.pl?file=../data/20091021/F006294.dat&query=3582&hit=1&index=IPI00031411&px=1&section=5&ave_thresh=21))

**3084 - 3101 709.7119 2126.1139 2126.1120 1 1 K.TSTPLDREEQAVYHLLVR.A**  ([Ions score 21](http://mbp-mascot4/mascot/cgi/peptide_view.pl?file=../data/20091021/F006294.dat&query=5027&hit=1&index=IPI00031411&px=1&section=5&ave_thresh=21))

**3084 - 3101 709.7125 2126.1155 2126.1120 2 1 K.TSTPLDREEQAVYHLLVR.A**  ([Ions score 39](http://mbp-mascot4/mascot/cgi/peptide_view.pl?file=../data/20091021/F006294.dat&query=5029&hit=1&index=IPI00031411&px=1&section=5&ave_thresh=21))

**3570 - 3601 1173.5469 3517.6188 3517.6195 -0 0 K.IHATDQDVYDTLTYSLDPQMDNLFSVSSTGGK.L**  ([Ions score 79](http://mbp-mascot4/mascot/cgi/peptide_view.pl?file=../data/20091021/F006294.dat&query=6886&hit=1&index=IPI00031411&px=1&section=5&ave_thresh=21))

**3570 - 3601 1173.5470 3517.6191 3517.6195 -0 0 K.IHATDQDVYDTLTYSLDPQMDNLFSVSSTGGK.L**  ([Ions score 80](http://mbp-mascot4/mascot/cgi/peptide_view.pl?file=../data/20091021/F006294.dat&query=6887&hit=1&index=IPI00031411&px=1&section=5&ave_thresh=21))

**3650 - 3664 922.4376 1842.8606 1842.8577 2 0 R.FANLTPEEFVGDYWR.N**  ([Ions score 24](http://mbp-mascot4/mascot/cgi/peptide_view.pl?file=../data/20091021/F006294.dat&query=4360&hit=1&index=IPI00031411&px=1&section=5&ave_thresh=21))

**HPDE A4:**

Match to: **IPI00031411** Score: **98**

**Gene_Symbol=FAT1 Protocadherin Fat 1 lng=4591 # SP[4593,D,22,D]SNP[4646,g,1064,R]SNP[4677,r,1064,R]SNP[4713,i,1125,I]SNP[4791,l,1125,I]SNP[4844,i,1252,I]SNP[4856,h,1273,H]SNP[4889,r,1273,H]SNP[4931,l,1283,P]SNP[4969,p,1283,P]SNP[5000,l,129,V]SNP[5**

Found in search of C:\mgf\Orbidata\080723_ISW1295_HPDE_A4.mgf

Nominal mass (M_r_): **1068827**; Calculated pI value: **4.55**

NCBI BLAST search of [IPI00031411](http://www.ncbi.nlm.nih.gov/blast/Blast.cgi?ALIGNMENTS=50&ALIGNMENT_VIEW=Pairwise&AUTO_FORMAT=Semiauto&CDD_SEARCH=on&CLIENT=web&COMPOSITION_BASED_STATISTICS=on&DATABASE=nr&DESCRIPTIONS=100&ENTREZ_QUERY=(none)&EXPECT=10&FILTER=L&FORMAT_BLOCK_ON_RESPAGE=None&FORMAT_OBJECT=Alignment&FORMAT_TYPE=HTML&GAPCOSTS=11+1&I_THRESH=0.001&LAYOUT=TwoWindows&MATRIX_NAME=BLOSUM62&NCBI_GI=on&PAGE=Proteins&PROGRAM=blastp&QUERY=IPI00031411&SERVICE=plain&SET_DEFAULTS.x=21&SET_DEFAULTS.y=7&SHOW_OVERVIEW=on&WORD_SIZE=3&END_OF_HTTPGET=Yes) against nr

Unformatted [sequence string](http://mbp-mascot4/mascot/cgi/getseq.pl?IPI_human+IPI00031411+seq) for pasting into other applications

Fixed modifications: Carbamidomethyl (C)

Variable modifications: Oxidation (M)

Cleavage by TrypsinMSIPI, a mixture of enzymes:

cuts C-term side of KR unless next residue is P

cuts C-term side of J

cuts N-term side of J

Sequence Coverage: **0%**

Matched peptides shown in **Bold Red**

**1** MGRHLALLLL LLLLFQHFGD SDGSQRLEQT PLQFTHLEYN VTVQENSAAK

**51** TYVGHPVKMG VYITHPAWEV RYKIVSGDSE NLFKAEEYIL GDFCFLRIRT

**101** KGGNTAILNR EVKDHYTLIV KALEKNTNVE ARTKVRVQVL DTNDLRPLFS

**151** PTSYSVSLPE NTAIRTSIAR VSATDADIGT NGEFYYSFKD RTDMFAIHPT

**201** SGVIVLTGRL DYLETKLYEM EILAADRGMK LYGSSGISSM AKLTVHIEQA

**251** NECAPVITAV TLSPSELDRD PAYAIVTVDD CDQGANGDIA SLSIVAGDLL

**301** QQFRTVRSFP GSKEYKVKAI GGIDWDSHPF GYNLTLQAKD KGTPPQFSSV

**351** KVIHVTSPQF KAGPVKFEKD VYRAEISEFA PPNTPVVMVK AIPAYSHLRY

**401** VFKSTPGKAK FSLNYNTGLI SILEPVKRQQ AAHFELEVTT SDRKASTKVL

**451** VKVLGANSNP PEFTQTAYKA AFDENVPIGT TVMSLSAVDP DEGENGYVTY

**501** SIANLNHVPF AIDHFTGAVS TSENLDYELM PRVYTLRIRA SDWGLPYRRE

**551** VEVLATITLN NLNDNTPLFE KINCEGTIPR DLGVGEQITT VSAIDADELQ

**601** LVQYQIEAGN ELDFFSLNPN SGVLSLKRSL MDGLGAKVSF HSLRITATDG

**651** ENFATPLYIN ITVAASHKLV NLQCEETGVA KMLAEKLLQA NKLHNQGEVE

**701** DIFFDSHSVN AHIPQFRSTL PTGIQVKENQ PVGSSVIFMN STDLDTGFNG

**751** KLVYAVSGGN EDSCFMIDME TGMLKILSPL DRETTDKYTL NITVYDLGIP

**801** QKAAWRLLHV VVVDANDNPP EFLQESYFVE VSEDKEVHSE IIQVEATDKD

**851** LGPNGHVTYS IVTDTDTFSI DSVTGVVNIA RPLDRELQHE HSLKIEARDQ

**901** AREEPQLFST VVVKVSLEDV NDNPPTFIPP NYRVKVREDL PEGTVIMWLE

**951** AHDPDLGQSG QVRYSLLDHG EGNFDVDKLS GAVRIVQQLD FEKKQVYNLT

**1001** VRAKDKGKPV SLSSTCYVEV EVVDVNENLH PPVFSSFVEK GTVKEDAPVG

**1051** SLVMTVSAHD EDARRDGEIR YSIRDGSGVG VFKIGEETGV IETSDRLDRE

**1101** STSHYWLTVF ATDQGVVPLS SFIEIYIEVE DVNDNAPQTS EPVYYPEIME

**1151** NSPKDVSVVQ IEAFDPDSSS NDKLMYKITS GNPQGFFSIH PKTGLITTTS

**1201** RKLDREQQDE HILEVTVTDN GSPPKSTIAR VIVKILDEND NKPQFLQKFY

**1251** KIRLPEREKP DRERNARREP LYHVIATDKD EGPNAEISYS IEDGNEHGKF

**1301** FIEPKTGVVS SKRFSAAGEY DILSIKAVDN GRPQKSSTTR LHIEWISKPK

**1351** PSLEPISFEE SFFTFTVMES DPVAHMIGVI SVEPPGIPLW FDITGGNYDS

**1401** HFDVDKGTGT IIVAKPLDAE QKSNYNLTVE ATDGTTTILT QVFIKVIDTN

**1451** DHRPQFSTSK YEVVIPEDTA PETEILQISA VDQDEKNKLI YTLQSSRDPL

**1501** SLKKFRLDPA TGSLYTSEKL DHEAVHQHTL TVMVRDQDVP VKRNFARIVV

**1551** NVSDTNDHAP WFTASSYKGR VYESAAVGSV VLQVTALDKD KGKNAEVLYS

**1601** IESGTFGNIG NSFMIDPVLG SIKTAKELDR SNQAEYDLMV KATDKGSPPM

**1651** SEITSVRIFV TIADNASPKF TSKEYSVELS ETVSIGSFVG MVTAHSQSSV

**1701** VYEIK**DGNTG DAFDINPHSG TIITQK**ALDF ETLPIYTLII QGTNMAGLST

**1751** NTTVLVHLQD ENDNAPVFMQ AEYTGLISES ASINSVVLTD RNVPLVIRAA

**1801** DADKDSNALL VYHIVEPSVH TYFAIDSSTG AIHTVLSLDY EETSIFHFTV

**1851** QVHDMGTPRL FAEYAANVTV HVIDINDCPP VFAKPLYEAS LLLPTYKGVK

**1901** VITVNATDAD SSAFSQLIYS ITEGNIGEKF SMDYKTGALT VQNTTQLRSR

**1951** YELTVRASDG RFAGLTSVKI NVKESKESHL KFTQDVYSAV VKENSTEAET

**2001** LAVITAIGNP INEPLFYHIL NPDRRFKISR **TSGVLSTTGT PFDR**EQQEAF

**2051** DVVVEVTEEH KPSAVAHVVV K**VIVEDQNDN APVFVNLPYY AVVK**VDTEVG

**2101** HVIRYVTAVD RDSGRNGEVH YYLKEHHEHF QIGPLGEISL KKQFELDTLN

**2151** KEYLVTVVAK DGGNPAFSAE VIVPITVMNK AMPVFEKPFY SAEIAESIQV

**2201** HSPVVHVQAN SPEGLKVFYS ITDGDPFSQF TINFNTGVIN VIAPLDFEAH

**2251** PAYKLSIRAT DSLTGAHAEV FVDIIVDDIN DNPPVFAQQS YAVTLSEASV

**2301** IGTSVVQVRA TDSDSEPNRG ISYQMFGNHS KSHDHFHVDS STGLISLLRT

**2351** LDYEQSRQHT IFVRAVDGGM PTLSSDVIVT VDVTDLNDNP PLFEQQIYEA

**2401** RISEHAPHGH FVTCVKAYDA DSSDIDKLQY SILSGNDHKH FVIDSATGII

**2451** TLSNLHRHAL KPFYSLNLSV SDGVFRSSTQ VHVTVIGGNL HSPAFLQNEY

**2501** EVELAENAPL HTLVMEVKTT DGDSGIYGHV TYHIVNDFAK DRFYINERGQ

**2551** IFTLEKLDRE TPAEKVISVR LMAKDAGGKV AFCTVNVILT DDNDNAPQFR

**2601** ATKYEVNIGS SAAKGTSVVK VLASDADEGS NADITYAIEA DSESVKENLE

**2651** INKLSGVITT KESLIGLENE FFTFFVRAVD NGSPSKESVV LVYVKILPPE

**2701** MQLPKFSEPF YTFTVSEDVP IGTEIDLIRA EHSGTVLYSL VKGNTPESNR

**2751** DESFVIDRQS GRLKLEKSLD HETTKWYQFS ILARCTQDDH EMVASVDVSI

**2801** QVKDANDNSP VFESSPYEAF IVENLPGGSR VIQIRASDAD SGTNGQVMYS

**2851** LDQSQSVEVI ESFAINMETG WITTLKELDH EKRDNYQIKV VASDHGEKIQ

**2901** LSSTAIVDVT VTDVNDSPPR FTAEIYKGTV SEDDPQGGVI AILSTTDADS

**2951** EEINRQVTYF ITGGDPLGQF AVETIQNEWK VYVKKPLDRE KRDNYLLTIT

**3001** ATDGTFSSKA IVEVKVLDAN DNSPVCEKTL YSDTIPEDVL PGKLIMQISA

**3051** TDADIRSNAE ITYTLLGSGA EKFKLNPDTG ELKTSTPLDR EEQAVYHLLV

**3101** RATDGGGRFC QASIVLTLED VNDNAPEFSA DPYAITVFEN TEPGTLLTRV

**3151** QATDADAGLN RKILYSLIDS ADGQFSINEL SGIIQLEKPL DRELQAVYTL

**3201** SLKAVDQGLP RRLTATGTVI VSVLDINDNP PVFEYREYGA TVSEDILVGT

**3251** EVLQVYAASR DIEANAEITY SIISGNEHGK FSIDSKTGAV FIIENLDYES

**3301** SHEYYLTVEA TDGGTPSLSD VATVNVNVTD INDNTPVFSQ DTYTTVISED

**3351** AVLEQSVITV MADDADGPSN SHIHYSIIDG NQGSSFTIDP VRGEVKVTKL

**3401** LDRETISGYT LTVQASDNGS PPRVNTTTVN IDVSDVNDNA PVFSRGNYSV

**3451** IIQENKPVGF SVLQLVVTDE DSSHNGPPFF FTIVTGNDEK AFEVNPQGVL

**3501** LTSSAIKRKE KDHYLLQVKV ADNGKPQLSS LTYIDIRVIE ESIYPPAILP

**3551** LEIFITSSGE EYSGGVIGKI HATDQDVYDT LTYSLDPQMD NLFSVSSTGG

**3601** KLIAHKKLDI GQYLLNVSVT DGKFTTVADI TVHIRQVTQE MLNHTIAIRF

**3651** ANLTPEEFVG DYWRNFQRAL RNILGVRRND IQIVSLQSSE PHPHLDVLLF

**3701** VEKPGSAQIS TKQLLHKINS SVTDIEEIIG VRILNVFQKL CAGLDCPWKF

**3751** CDEKVSVDES VMSTHSTARL SFVTPRHHRA AVCLCKEGRC PPVHHGCEDD

**3801** PCPEGSECVS DPWEEKHTCV CPSGRFGQCP GSSSMTLTGN SYVKYRLTEN

**3851** ENKLEMKLTM RLRTYSTHAV VMYARGTDYS ILEIHHGRLQ YKFDCGSGPG

**3901** IVSVQSIQVN DGQWHAVALE VNGNYARLVL DQVHTASGTA PGTLKTLNLD

**3951** NYVFFGGHIR QQGTRHGRSP QVGNGFRGCM DSIYLNGQEL PLNSKPRSYA

**4001** HIEESVDVSP GCFLTATEDC ASNPCQNGGV CNPSPAGGYY CKCSALYIGT

**4051** HCEISVNPCS SKPCLYGGTC VVDNGGFVCQ CRGLYTGQRC QLSPYCKDEP

**4101** CKNGGTCFDS LDGAVCQCDS GFRGERCQSD IDECSGNPCL HGALCENTHG

**4151** SYHCNCSHEY RGRHCEDAAP NQYVSTPWNI GLAEGIGIVV FVAGIFLLVV

**4201** VFVLCRKMIS RKKKHQAEPK DKHLGPATAF LQRPYFDSKL NKNIYSDIPP

**4251** QVPVRPISYT PSIPSDSRNN LDRNSFEGSA IPEHPEFSTF NPESVHGHRK

**4301** AVAVCSVAPN LPPPPPSNSP SDSDSIQKPS WDFDYDTKVV DLDPCLSKKP

**4351** LEEKPSQPYS ARESLSEVQS LSSFQSESCD DNGYHWDTSD WMPSVPLPDI

**4401** QEFPNYEVID EQTPLYSADP NAIDTDYYPG GYDIESDFPP PPEDFPAADE

**4451** LPPLPPEFSN QFESIHPPRD MPAAGSLGSS SRNRQRFNLN QYLPNFYPLD

**4501** MSEPQTKGTG ENSTCREPHA PYPPGYQRHF EAPAVESMPM SVYASTASCS

**4551** DVSACCEVES EVMMSDYESG DDGHFEEVTI PPLDSQQHTE VJDGSQRLEQ

**4601** TPLQFTHLEY NVTVQENSAA KJGTVKEDAP VGSLVMTVSA HDEDAGRDGE

**4651** IRJGTVKEDA PVGSLVMTVS AHDEDARRDG EIRJLDREST SHYWLTVFAT

**4701** DQGVVPLSSF IEIYIEVEDV NDNAPQTSEP VYYPEIMENS PKDVSVVQIE

**4751** AFDPDSSSND KJLDRESTSH YWLTVFATDQ GVVPLSSFIE LYIEVEDVND

**4801** NAPQTSEPVY YPEIMENSPK DVSVVQIEAF DPDSSSNDKJ FYKIRLPERJ

**4851** REPLYHVIAT DKDEGPNAEI SYSIEDGNEH GKJREPLYRV IATDKDEGPN

**4901** AEISYSIEDG NEHGKJEPLY HVIATDKDEG LNAEISYSIE DGNEHGKFFI

**4951** EPKJEPLYHV IATDKDEGPN AEISYSIEDG NEHGKFFIEP KJALEKNTNL

**5001** EARTKJALEK NTNVEARTKJ ALEKNTNVEA RTKJALEKNT NVEVRTKJFS

**5051** AAGEYDILSI KAVDNGRPQK SSTTRJFSAA GEYDILSIKA VDSGRPQKSS

**5101** TTRJNFARIV VNVSDTNDHA PWFTASSYKG RJNFARIVVN VSDTNDHAPW

**5151** FTTSSYKGRJ GKNAEVLYSI ESGDFGNIGN SFMIDPVLGS IKTAKJGKNA

**5201** EVLYSIESGN FGNIGNSFMI DPVLGSIKTA KJGKNAEVLY SIESGTIGNI

**5251** GNSFMIDPVL GSIKTAKJGS PPMSEITSVR IFVTNADNAS PKFTSKJGSP

**5301** PMSEITSVRI FVTSADNASP KFTSKJGSPP MSEITSVRIF VTIADNASTK

**5351** FTSKJALDFE TLPIYTLIIQ GTNMAGLSTN TTVLVHLQDE NDNAPVFMQA

**5401** EYTGLISESA SINSVVLTDR NVPQVIRAAD ADKJALDFET LPIYTLIIQG

**5451** TNMAGLSTNT TVLVHLQDEN DNAPVFMQAE YTGLISESAS INSVVLTDRN

**5501** VPRVIRAADA DKJAADADKD SNVLLVYHIV EPSVHTYFAI DSSTGAIHTV

**5551** LSLDYEETSI FHFTVQVHDM GTPRLFAEYA ANVTVHVIDI NDCPPVFAKP

**5601** LYEASLLLPT YKJFTQDVYS AVVKTNSTEA ETLAVITAIG NPINEPLFYH

**5651** ILNPDRRJTS GVLSTTGTPF DREAQEAFDV VVEVTEEHKP SAVAHVVVKV

**5701** IVEDQNDNAP VFVNLPYYAV VKJTDMFAIH PTSGVIVLTG RLDFLETKLY

**5751** EMEILAADRJ TDMFAIHPTS GVIVLTGRLD YLETKLYEME ILAADRJNGE

**5801** VHYYLKEHHE HFQIGPLGEK SLKKJDGGNP AFSAEVIVPI TVMNKAMPVF

**5851** EKPFYSAEIA ESIQVHSHVV HVQANSPEGL KVFYSITDGD PFSQFTINFN

**5901** TGVINVIAPL DFEAHPAYKJ AMPVFEKPFY SAEIAESIQV HSPVVHVQAN

**5951** SPEGLKVFYS ITDGDPFSQF TINFNTGVIN VIAPLDFPAH PAYKLSIRJL

**6001** SIRATDSLTG AHAEVFVDDI VDDINDNPPV FAQQSYAVTL SEASVIGTSV

**6051** VQVRATDSDS EPNRJLSIRA TDSLTGAHAE VFVDEIVDDI NDNPPVFAQQ

**6101** SYAVTLSEAS VIGTSVVQVR ATDSDSEPNR JTLDYEQSRQ HTIAVRAVDG

**6151** GMPTLSSDVI VTVDVTDLND NPPLFEQQIY EARJLYGSSG ISSMAKLTVH

**6201** IEQANECAPV ITAVTLSPSE LDRDPAYAIV TVDDCDQGAN GDIASLSIVA

**6251** GDLLQQFRJI LPPEMQLPKF SEPFYTFTVS EIVPIGTEID LIRAEHSGTV

**6301** LYSLVKJILP PEMQLPKFSE PFYTFTVSEV VPIGTEIDLI RAEHSGTVLY

**6351** SLVKJCTQDD HEMVASVDVS IQVKDANDNS PVFESSPYEA FIVENLPGGS

**6401** RVIQIRJCTQ DDHEMVASVD VSIQVKDASD NSPVFESSPY EAFIVENLPG

**6451** GSRVIQIRJV IQIRASDADN GTNGQVMYSL DQSQSVEVIE SFAINMETGW

**6501** ITTLKELDHE KJFTAEIYKG TVSELDPQGG VIAILSTTDA DSEEINRQVT

**6551** YFITGGDPLG QFAVETIQNE WKJFTAEIYK GTVSEPDPQG GVIAILSTTD

**6601** ADSEEINRQV TYFITGGDPL GQFAVETIQN EWKJFTAEIY KGTVSEQDPQ

**6651** GGVIAILSTT DADSEEINRQ VTYFITGGDP LGQFAVETIQ NEWKJGTVSE

**6701** DDPQGGVIAI LSTTDADSEE INRQVTYGIT GGDPLGQFAV ETIQNEWKVY

**6751** VKJGTVSEDD PQGGVIAILS TTDADSEEIN RQVTYSITGG DPLGQFAVET

**6801** IQNEWKVYVK JGTVSEDDPQ GGVIAILSTT DADSEEINRQ VTYFITGGDP

**6851** LAQFAVETIQ NEWKVYVKJA IVEVKVLDAN DNSPVCEYTL YSDTIPEDVL

**6901** PGKLIMQISA TDADIRJVLD ANDNSPVCEK TLYIDTIPED VLPGKLIMQI

**6951** SATDADIRJV LDANDNSPVC EKTLYTDTIP EDVLPGKLIM QISATDADIR

**7001** JATDGGGRFC QASIVLDLED VNDNAPEFSA DPYAITVFEN TEPGTLLTRV

**7051** QATDADAGLN RJKILYSLID SADGQFSINE LIGIIQLEKP LDRELQAVYT

**7101** LSLKJFSIDS KTGAVFIIEN LDYESSHEYY LTVEATDGGT PSLSDVATVN

**7151** VNVTDINDAT PVFSQDTYTT VISEDAVLEQ SVITVMADDA DGPSNSHIHY

**7201** SIIDGNQGSS FTIDPVRGEV KJFSIDSKTG AVFIIENLDY ESSHEYYLTV

**7251** EATDGGTPSL SDVATVNVNV TDINDVTPVF SQDTYTTVIS EDAVLEQSVI

**7301** TVMADDADGP SNSHIHYSII DGNQGSSFTI DPVRGEVKJV NTTTVNIDVS

**7351** DVNDNAPVFS RGNYSVIIQE NKPVGFSVLQ LVVTDEDSSH NGPPFFFTIV

**7401** TENDEKAFEV NPQGVLLTSS AIKJVNTTTV NIDVSDVNDN APVFSRGNYS

**7451** VIIQENKPVG FSVLQLVVTD EDSSHNGPPF FFTIVTGNDE KAFEVNPQGV

**7501** LLTSSAIKJG NYSVIIQENK PVGFSVLQLV VTDEDSSHNG PPFFFTIVTG

**7551** NDEKAFEVNA QGVLLTSSAI KRJGNYSVII QENKPVGFSV LQLVVTDEDS

**7601** SHNGPPFFFT IVTGNDEKAF EVNVQGVLLT SSAIKRJVAD NGKPQLSSLT

**7651** YIDIRVIEES IYPPAILPLE IAITSSGEEY SGGVIGKIHA TDQDVYDTLT

**7701** YSLDPQMDNL FSVSSTGGKJ VADNGKPQLS SLTYIDIRVI EESIYPPAIL

**7751** PLEISITSSG EEYSGGVIGK IHATDQDVYD TLTYSLDPQM DNLFSVSSTG

**7801** GKJVIHVTSP QFKAGPVKFE KJQLLHKIMS SVTDIEEIIG VRILNVFQKJ

**7851** QLLHKIVSSV TDIEEIIGVR ILNVFQKJEG RCPPVHHGCE DHPCPEGSEC

**7901** VSDPWEEKHT CVCPSGRJEG RCPPVHHGCE DPPCPEGSEC VSDPWEEKHT

**7951** CVCPSGRJHT CVCPSGRFGG CPGSSSMTLT GNSYVKYRJH TCVCPSGRFG

**8001** RCPGSSSMTL TGNSYVKYRJ FGQCPGSSSM TLTGNSYVKT RLTENENKJG

**8051** CMDSIYLNGQ ELPLNSKPRS YAHIEESVPV SPGCFLTATE DCASNPCQNG

**8101** GVCNPSPAGG YYCKCSALYI GTHCEISVNP CSSKPCLYGG TCVVDNGGFV

**8151** CQCRJYVFKR TPGKAKJYVF KSTPGKAKJS YAHIEESVDV SPGCFLTATE

**8201** DCASNPCQNG GVCNPSPAGG YYCKCSALYI GTHCEISVNP KSSKPCLYGG

**8251** TCVVDNGGFV CQCRGLYTGQ RJSYAHIEES VDVSPGCFLT ATEDCASNPC

**8301** QNGGVCNPSP AGGYYCKCSA LYIGTHCEIS VNPNSSKPCL YGGTCVVDNG

**8351** GFVCQCRGLY TGQRJDEPCK NGGTCFDSAD GAVCQCDSGF RGERJGRHCA

**8401** DAAPNQYVST PWNIGLAEGI GIVVFVAGIF LLVVVFVLCR KJGRHCEDAA

**8451** PNQYVSTPWN IGLAEGIGIV VFVAGIFLLV VVFVLCRKJN IYSDIPPQVP

**8501** VRYISYTPSI PSDSRNNLDR JKAVAVCSVA PNLPPPPPSN SPSDSDSIQK

**8551** PSWDFDYDTK VVDLDPCLSK JKPLEEKPSQ PYSARESLSS VQSLSSFQSE

**8601** SCDDNGYHWD TSDWMPSVPL PDIQEFPNYE VIDEQTPLYS ADPNAIDTDY

**8651** YPGGYDIESD FPPPPEDFPA ADELPPLPPE FSNQFESIHP PRDMPAAGSL

**8701** GSSSRJKPLE EKPSQPYSAR ESLSEVQSLS SFQSESCDDN GYHWDTSDWM

**8751** PSVPLQDIQE FPNYEVIDEQ TPLYSADPNA IDTDYYPGGY DIESDFPPPP

**8801** EDFPAADELP PLPPEFSNQF ESIHPPRDMP AAGSLGSSSR JKPLEEKPSQ

**8851** PYSARESLSE VQSLSSFQSE SCDDNGYHWD TSDWMPSVPL PDIQEFPNYE

**8901** VIDPQTPLYS ADPNAIDTDY YPGGYDIESD FPPPPEDFPA ADELPPLPPE

**8951** FSNQFESIHP PRDMPAAGSL GSSSRJESLS EVQSLSSFQS ESCDDNGYHW

**9001** DTSDWMPSVP LPDIQEFPNY EVIDEQTPLY SADPNAIDTD YYPGGYDIES

**9051** DFPPPPEDFP AADELPPLPP EFSNQFESIH PPRDMPAAGS LGSSRRNRJE

**9101** SLSEVQSLSS FQSESCDDNG YHWDTSDWMP SVPLPDIQEF PNYEVIDEQT

**9151** PLYSADPNAI DTDYYPGGYD IESDFPPPPE DFPAADELPP LPPEFSNQFE

**9201** SIHPPRDMPA AGSLGSSWRN RJVLGANSNP PEFTQTAYKA AFDENVPIGT

**9251** TIMSLSAVDP DEGENGYVTY SIANLNHVPF AIDHFTGAVS TSENLDYELM

**9301** PRVYTLRJVL GANSNPPEFT QTAYKAAFDE NVPIGTTVMS LSAVDPDEGE

**9351** NGYVTYSIAN LNHVPFAIDH FTGAVSTSEN LDYELMPRVY TLRJVLGANS

**9401** NPPEFTQTAY KAAFDENVPI GTTVMSLSAV DPDEGENGYV TYSIANLNHV

**9451** PFAIDHFTGA VSTSENLDYE LMPRVYTLRJ INCEGTIPRD LGVGEQITTV

**9501** SAIDADELQL VQYQIEAGNE LDFFSLNPNS GVLSLKRJIN CEGTIPRDLG

**9551** VGEQITTVSA IDADELQLVQ YQIEAGNELD LFSLNPNSGV LSLKRJINCE

**9601** GTIPRDLGVG EQITTVSAID ADELQLVQYQ IEAGNELDFF SLNPNSGVLS

**9651** LKRJEVHSEI IQVEATDKDL GPNGHVTYSI LTDTDTFSID SVTGVVNIAR

**9701** PLDRELQHEH SLKJEVHSEI IQVEATDKDL GPNGHVTYSI VTDTDTFSID

**9751** SVTGVVNIAR PLDRELQHEH SLK

**Start - End Observed Mr(expt) Mr(calc) ppm Miss Sequence**

**1706 - 1726 734.3555 2200.0447 2200.0397 2 0 K.DGNTGDAFDINPHSGTIITQK.A**  ([Ions score 51](http://mbp-mascot4/mascot/cgi/peptide_view.pl?file=../data/20091021/F006295.dat&query=5076&hit=1&index=IPI00031411&px=1&section=5&ave_thresh=22))

**1706 - 1726 734.3559 2200.0458 2200.0397 3 0 K.DGNTGDAFDINPHSGTIITQK.A**  ([Ions score 62](http://mbp-mascot4/mascot/cgi/peptide_view.pl?file=../data/20091021/F006295.dat&query=5077&hit=1&index=IPI00031411&px=1&section=5&ave_thresh=22))

**2031 - 2044 719.8613 1437.7080 1437.7100 -1 0 R.TSGVLSTTGTPFDR.E**  ([Ions score 21](http://mbp-mascot4/mascot/cgi/peptide_view.pl?file=../data/20091021/F006295.dat&query=2595&hit=1&index=IPI00031411&px=1&section=5&ave_thresh=22))

**2072 - 2094 869.4578 2605.3516 2605.3428 3 0 K.VIVEDQNDNAPVFVNLPYYAVVK.V**  ([Ions score 26](http://mbp-mascot4/mascot/cgi/peptide_view.pl?file=../data/20091021/F006295.dat&query=5825&hit=1&index=IPI00031411&px=1&section=5&ave_thresh=22))

**HPDE 1:**

Match to: **IPI00031411** Score: **44**

**Gene_Symbol=FAT1 Protocadherin Fat 1 lng=4591 # SP[4593,D,22,D]SNP[4646,g,1064,R]SNP[4677,r,1064,R]SNP[4713,i,1125,I]SNP[4791,l,1125,I]SNP[4844,i,1252,I]SNP[4856,h,1273,H]SNP[4889,r,1273,H]SNP[4931,l,1283,P]SNP[4969,p,1283,P]SNP[5000,l,129,V]SNP[5**

Found in search of C:\mgf\Orbidata\090430_ISW1295_HPDE_1.mgf

Nominal mass (M_r_): **1068827**; Calculated pI value: **4.55**

NCBI BLAST search of [IPI00031411](http://www.ncbi.nlm.nih.gov/blast/Blast.cgi?ALIGNMENTS=50&ALIGNMENT_VIEW=Pairwise&AUTO_FORMAT=Semiauto&CDD_SEARCH=on&CLIENT=web&COMPOSITION_BASED_STATISTICS=on&DATABASE=nr&DESCRIPTIONS=100&ENTREZ_QUERY=(none)&EXPECT=10&FILTER=L&FORMAT_BLOCK_ON_RESPAGE=None&FORMAT_OBJECT=Alignment&FORMAT_TYPE=HTML&GAPCOSTS=11+1&I_THRESH=0.001&LAYOUT=TwoWindows&MATRIX_NAME=BLOSUM62&NCBI_GI=on&PAGE=Proteins&PROGRAM=blastp&QUERY=IPI00031411&SERVICE=plain&SET_DEFAULTS.x=21&SET_DEFAULTS.y=7&SHOW_OVERVIEW=on&WORD_SIZE=3&END_OF_HTTPGET=Yes) against nr

Unformatted [sequence string](http://mbp-mascot4/mascot/cgi/getseq.pl?IPI_human+IPI00031411+seq) for pasting into other applications

Fixed modifications: Carbamidomethyl (C)

Variable modifications: Oxidation (M)

Cleavage by TrypsinMSIPI, a mixture of enzymes:

cuts C-term side of KR unless next residue is P

cuts C-term side of J

cuts N-term side of J

Sequence Coverage: **0%**

Matched peptides shown in **Bold Red**

**1** MGRHLALLLL LLLLFQHFGD SDGSQRLEQT PLQFTHLEYN VTVQENSAAK

**51** TYVGHPVKMG VYITHPAWEV RYKIVSGDSE NLFKAEEYIL GDFCFLRIRT

**101** KGGNTAILNR EVKDHYTLIV KALEKNTNVE ARTKVRVQVL DTNDLRPLFS

**151** PTSYSVSLPE NTAIRTSIAR VSATDADIGT NGEFYYSFKD RTDMFAIHPT

**201** SGVIVLTGRL DYLETKLYEM EILAADRGMK LYGSSGISSM AKLTVHIEQA

**251** NECAPVITAV TLSPSELDRD PAYAIVTVDD CDQGANGDIA SLSIVAGDLL

**301** QQFRTVRSFP GSKEYKVKAI GGIDWDSHPF GYNLTLQAKD KGTPPQFSSV

**351** KVIHVTSPQF KAGPVKFEKD VYRAEISEFA PPNTPVVMVK AIPAYSHLRY

**401** VFKSTPGKAK FSLNYNTGLI SILEPVKRQQ AAHFELEVTT SDRKASTKVL

**451** VKVLGANSNP PEFTQTAYKA AFDENVPIGT TVMSLSAVDP DEGENGYVTY

**501** SIANLNHVPF AIDHFTGAVS TSENLDYELM PRVYTLRIRA SDWGLPYRRE

**551** VEVLATITLN NLNDNTPLFE KINCEGTIPR DLGVGEQITT VSAIDADELQ

**601** LVQYQIEAGN ELDFFSLNPN SGVLSLKRSL MDGLGAKVSF HSLRITATDG

**651** ENFATPLYIN ITVAASHKLV NLQCEETGVA KMLAEKLLQA NKLHNQGEVE

**701** DIFFDSHSVN AHIPQFRSTL PTGIQVKENQ PVGSSVIFMN STDLDTGFNG

**751** KLVYAVSGGN EDSCFMIDME TGMLKILSPL DRETTDKYTL NITVYDLGIP

**801** QKAAWRLLHV VVVDANDNPP EFLQESYFVE VSEDKEVHSE IIQVEATDKD

**851** LGPNGHVTYS IVTDTDTFSI DSVTGVVNIA RPLDRELQHE HSLKIEARDQ

**901** AREEPQLFST VVVKVSLEDV NDNPPTFIPP NYRVKVREDL PEGTVIMWLE

**951** AHDPDLGQSG QVRYSLLDHG EGNFDVDKLS GAVRIVQQLD FEKKQVYNLT

**1001** VRAKDKGKPV SLSSTCYVEV EVVDVNENLH PPVFSSFVEK GTVKEDAPVG

**1051** SLVMTVSAHD EDARRDGEIR YSIRDGSGVG VFKIGEETGV IETSDRLDRE

**1101** STSHYWLTVF ATDQGVVPLS SFIEIYIEVE DVNDNAPQTS EPVYYPEIME

**1151** NSPKDVSVVQ IEAFDPDSSS NDKLMYKITS GNPQGFFSIH PKTGLITTTS

**1201** RKLDREQQDE HILEVTVTDN GSPPKSTIAR VIVKILDEND NKPQFLQKFY

**1251** KIRLPEREKP DRERNARREP LYHVIATDKD EGPNAEISYS IEDGNEHGKF

**1301** FIEPKTGVVS SKRFSAAGEY DILSIKAVDN GRPQKSSTTR LHIEWISKPK

**1351** PSLEPISFEE SFFTFTVMES DPVAHMIGVI SVEPPGIPLW FDITGGNYDS

**1401** HFDVDKGTGT IIVAKPLDAE QKSNYNLTVE ATDGTTTILT QVFIKVIDTN

**1451** DHRPQFSTSK YEVVIPEDTA PETEILQISA VDQDEKNKLI YTLQSSRDPL

**1501** SLKKFRLDPA TGSLYTSEKL DHEAVHQHTL TVMVRDQDVP VKRNFARIVV

**1551** NVSDTNDHAP WFTASSYKGR VYESAAVGSV VLQVTALDKD KGKNAEVLYS

**1601** IESGTFGNIG NSFMIDPVLG SIKTAKELDR SNQAEYDLMV KATDKGSPPM

**1651** SEITSVR**IFV TIADNASPK**F TSKEYSVELS ETVSIGSFVG MVTAHSQSSV

**1701** VYEIKDGNTG DAFDINPHSG TIITQKALDF ETLPIYTLII QGTNMAGLST

**1751** NTTVLVHLQD ENDNAPVFMQ AEYTGLISES ASINSVVLTD RNVPLVIRAA

**1801** DADKDSNALL VYHIVEPSVH TYFAIDSSTG AIHTVLSLDY EETSIFHFTV

**1851** QVHDMGTPRL FAEYAANVTV HVIDINDCPP VFAKPLYEAS LLLPTYKGVK

**1901** VITVNATDAD SSAFSQLIYS ITEGNIGEKF SMDYKTGALT VQNTTQLRSR

**1951** YELTVRASDG RFAGLTSVKI NVKESKESHL KFTQDVYSAV VKENSTEAET

**2001** LAVITAIGNP INEPLFYHIL NPDRRFKISR TSGVLSTTGT PFDREQQEAF

**2051** DVVVEVTEEH KPSAVAHVVV KVIVEDQNDN APVFVNLPYY AVVKVDTEVG

**2101** HVIRYVTAVD RDSGRNGEVH YYLKEHHEHF QIGPLGEISL KKQFELDTLN

**2151** KEYLVTVVAK DGGNPAFSAE VIVPITVMNK AMPVFEKPFY SAEIAESIQV

**2201** HSPVVHVQAN SPEGLKVFYS ITDGDPFSQF TINFNTGVIN VIAPLDFEAH

**2251** PAYKLSIRAT DSLTGAHAEV FVDIIVDDIN DNPPVFAQQS YAVTLSEASV

**2301** IGTSVVQVRA TDSDSEPNRG ISYQMFGNHS KSHDHFHVDS STGLISLLRT

**2351** LDYEQSRQHT IFVRAVDGGM PTLSSDVIVT VDVTDLNDNP PLFEQQIYEA

**2401** RISEHAPHGH FVTCVKAYDA DSSDIDKLQY SILSGNDHKH FVIDSATGII

**2451** TLSNLHRHAL KPFYSLNLSV SDGVFRSSTQ VHVTVIGGNL HSPAFLQNEY

**2501** EVELAENAPL HTLVMEVKTT DGDSGIYGHV TYHIVNDFAK DRFYINERGQ

**2551** IFTLEKLDRE TPAEKVISVR LMAKDAGGKV AFCTVNVILT DDNDNAPQFR

**2601** ATKYEVNIGS SAAKGTSVVK VLASDADEGS NADITYAIEA DSESVKENLE

**2651** INKLSGVITT KESLIGLENE FFTFFVRAVD NGSPSKESVV LVYVKILPPE

**2701** MQLPKFSEPF YTFTVSEDVP IGTEIDLIRA EHSGTVLYSL VKGNTPESNR

**2751** DESFVIDRQS GRLKLEKSLD HETTKWYQFS ILARCTQDDH EMVASVDVSI

**2801** QVKDANDNSP VFESSPYEAF IVENLPGGSR VIQIRASDAD SGTNGQVMYS

**2851** LDQSQSVEVI ESFAINMETG WITTLKELDH EKRDNYQIKV VASDHGEKIQ

**2901** LSSTAIVDVT VTDVNDSPPR FTAEIYKGTV SEDDPQGGVI AILSTTDADS

**2951** EEINRQVTYF ITGGDPLGQF AVETIQNEWK VYVKKPLDRE KRDNYLLTIT

**3001** ATDGTFSSKA IVEVKVLDAN DNSPVCEKTL YSDTIPEDVL PGKLIMQISA

**3051** TDADIRSNAE ITYTLLGSGA EKFKLNPDTG ELKTSTPLDR EEQAVYHLLV

**3101** RATDGGGRFC QASIVLTLED VNDNAPEFSA DPYAITVFEN TEPGTLLTRV

**3151** QATDADAGLN RKILYSLIDS ADGQFSINEL SGIIQLEKPL DRELQAVYTL

**3201** SLKAVDQGLP RRLTATGTVI VSVLDINDNP PVFEYREYGA TVSEDILVGT

**3251** EVLQVYAASR DIEANAEITY SIISGNEHGK FSIDSKTGAV FIIENLDYES

**3301** SHEYYLTVEA TDGGTPSLSD VATVNVNVTD INDNTPVFSQ DTYTTVISED

**3351** AVLEQSVITV MADDADGPSN SHIHYSIIDG NQGSSFTIDP VRGEVKVTKL

**3401** LDRETISGYT LTVQASDNGS PPRVNTTTVN IDVSDVNDNA PVFSRGNYSV

**3451** IIQENKPVGF SVLQLVVTDE DSSHNGPPFF FTIVTGNDEK AFEVNPQGVL

**3501** LTSSAIKRKE KDHYLLQVKV ADNGKPQLSS LTYIDIRVIE ESIYPPAILP

**3551** LEIFITSSGE EYSGGVIGKI HATDQDVYDT LTYSLDPQMD NLFSVSSTGG

**3601** KLIAHKKLDI GQYLLNVSVT DGKFTTVADI TVHIRQVTQE MLNHTIAIRF

**3651** ANLTPEEFVG DYWRNFQRAL RNILGVRRND IQIVSLQSSE PHPHLDVLLF

**3701** VEKPGSAQIS TKQLLHKINS SVTDIEEIIG VRILNVFQKL CAGLDCPWKF

**3751** CDEKVSVDES VMSTHSTARL SFVTPRHHRA AVCLCKEGRC PPVHHGCEDD

**3801** PCPEGSECVS DPWEEKHTCV CPSGRFGQCP GSSSMTLTGN SYVKYRLTEN

**3851** ENKLEMKLTM RLRTYSTHAV VMYARGTDYS ILEIHHGRLQ YKFDCGSGPG

**3901** IVSVQSIQVN DGQWHAVALE VNGNYARLVL DQVHTASGTA PGTLKTLNLD

**3951** NYVFFGGHIR QQGTRHGRSP QVGNGFRGCM DSIYLNGQEL PLNSKPRSYA

**4001** HIEESVDVSP GCFLTATEDC ASNPCQNGGV CNPSPAGGYY CKCSALYIGT

**4051** HCEISVNPCS SKPCLYGGTC VVDNGGFVCQ CRGLYTGQRC QLSPYCKDEP

**4101** CKNGGTCFDS LDGAVCQCDS GFRGERCQSD IDECSGNPCL HGALCENTHG

**4151** SYHCNCSHEY RGRHCEDAAP NQYVSTPWNI GLAEGIGIVV FVAGIFLLVV

**4201** VFVLCRKMIS RKKKHQAEPK DKHLGPATAF LQRPYFDSKL NKNIYSDIPP

**4251** QVPVRPISYT PSIPSDSRNN LDRNSFEGSA IPEHPEFSTF NPESVHGHRK

**4301** AVAVCSVAPN LPPPPPSNSP SDSDSIQKPS WDFDYDTKVV DLDPCLSKKP

**4351** LEEKPSQPYS ARESLSEVQS LSSFQSESCD DNGYHWDTSD WMPSVPLPDI

**4401** QEFPNYEVID EQTPLYSADP NAIDTDYYPG GYDIESDFPP PPEDFPAADE

**4451** LPPLPPEFSN QFESIHPPRD MPAAGSLGSS SRNRQRFNLN QYLPNFYPLD

**4501** MSEPQTKGTG ENSTCREPHA PYPPGYQRHF EAPAVESMPM SVYASTASCS

**4551** DVSACCEVES EVMMSDYESG DDGHFEEVTI PPLDSQQHTE VJDGSQRLEQ

**4601** TPLQFTHLEY NVTVQENSAA KJGTVKEDAP VGSLVMTVSA HDEDAGRDGE

**4651** IRJGTVKEDA PVGSLVMTVS AHDEDARRDG EIRJLDREST SHYWLTVFAT

**4701** DQGVVPLSSF IEIYIEVEDV NDNAPQTSEP VYYPEIMENS PKDVSVVQIE

**4751** AFDPDSSSND KJLDRESTSH YWLTVFATDQ GVVPLSSFIE LYIEVEDVND

**4801** NAPQTSEPVY YPEIMENSPK DVSVVQIEAF DPDSSSNDKJ FYKIRLPERJ

**4851** REPLYHVIAT DKDEGPNAEI SYSIEDGNEH GKJREPLYRV IATDKDEGPN

**4901** AEISYSIEDG NEHGKJEPLY HVIATDKDEG LNAEISYSIE DGNEHGKFFI

**4951** EPKJEPLYHV IATDKDEGPN AEISYSIEDG NEHGKFFIEP KJALEKNTNL

**5001** EARTKJALEK NTNVEARTKJ ALEKNTNVEA RTKJALEKNT NVEVRTKJFS

**5051** AAGEYDILSI KAVDNGRPQK SSTTRJFSAA GEYDILSIKA VDSGRPQKSS

**5101** TTRJNFARIV VNVSDTNDHA PWFTASSYKG RJNFARIVVN VSDTNDHAPW

**5151** FTTSSYKGRJ GKNAEVLYSI ESGDFGNIGN SFMIDPVLGS IKTAKJGKNA

**5201** EVLYSIESGN FGNIGNSFMI DPVLGSIKTA KJGKNAEVLY SIESGTIGNI

**5251** GNSFMIDPVL GSIKTAKJGS PPMSEITSVR IFVTNADNAS PKFTSKJGSP

**5301** PMSEITSVRI FVTSADNASP KFTSKJGSPP MSEITSVRIF VTIADNASTK

**5351** FTSKJALDFE TLPIYTLIIQ GTNMAGLSTN TTVLVHLQDE NDNAPVFMQA

**5401** EYTGLISESA SINSVVLTDR NVPQVIRAAD ADKJALDFET LPIYTLIIQG

**5451** TNMAGLSTNT TVLVHLQDEN DNAPVFMQAE YTGLISESAS INSVVLTDRN

**5501** VPRVIRAADA DKJAADADKD SNVLLVYHIV EPSVHTYFAI DSSTGAIHTV

**5551** LSLDYEETSI FHFTVQVHDM GTPRLFAEYA ANVTVHVIDI NDCPPVFAKP

**5601** LYEASLLLPT YKJFTQDVYS AVVKTNSTEA ETLAVITAIG NPINEPLFYH

**5651** ILNPDRRJTS GVLSTTGTPF DREAQEAFDV VVEVTEEHKP SAVAHVVVKV

**5701** IVEDQNDNAP VFVNLPYYAV VKJTDMFAIH PTSGVIVLTG RLDFLETKLY

**5751** EMEILAADRJ TDMFAIHPTS GVIVLTGRLD YLETKLYEME ILAADRJNGE

**5801** VHYYLKEHHE HFQIGPLGEK SLKKJDGGNP AFSAEVIVPI TVMNKAMPVF

**5851** EKPFYSAEIA ESIQVHSHVV HVQANSPEGL KVFYSITDGD PFSQFTINFN

**5901** TGVINVIAPL DFEAHPAYKJ AMPVFEKPFY SAEIAESIQV HSPVVHVQAN

**5951** SPEGLKVFYS ITDGDPFSQF TINFNTGVIN VIAPLDFPAH PAYKLSIRJL

**6001** SIRATDSLTG AHAEVFVDDI VDDINDNPPV FAQQSYAVTL SEASVIGTSV

**6051** VQVRATDSDS EPNRJLSIRA TDSLTGAHAE VFVDEIVDDI NDNPPVFAQQ

**6101** SYAVTLSEAS VIGTSVVQVR ATDSDSEPNR JTLDYEQSRQ HTIAVRAVDG

**6151** GMPTLSSDVI VTVDVTDLND NPPLFEQQIY EARJLYGSSG ISSMAKLTVH

**6201** IEQANECAPV ITAVTLSPSE LDRDPAYAIV TVDDCDQGAN GDIASLSIVA

**6251** GDLLQQFRJI LPPEMQLPKF SEPFYTFTVS EIVPIGTEID LIRAEHSGTV

**6301** LYSLVKJILP PEMQLPKFSE PFYTFTVSEV VPIGTEIDLI RAEHSGTVLY

**6351** SLVKJCTQDD HEMVASVDVS IQVKDANDNS PVFESSPYEA FIVENLPGGS

**6401** RVIQIRJCTQ DDHEMVASVD VSIQVKDASD NSPVFESSPY EAFIVENLPG

**6451** GSRVIQIRJV IQIRASDADN GTNGQVMYSL DQSQSVEVIE SFAINMETGW

**6501** ITTLKELDHE KJFTAEIYKG TVSELDPQGG VIAILSTTDA DSEEINRQVT

**6551** YFITGGDPLG QFAVETIQNE WKJFTAEIYK GTVSEPDPQG GVIAILSTTD

**6601** ADSEEINRQV TYFITGGDPL GQFAVETIQN EWKJFTAEIY KGTVSEQDPQ

**6651** GGVIAILSTT DADSEEINRQ VTYFITGGDP LGQFAVETIQ NEWKJGTVSE

**6701** DDPQGGVIAI LSTTDADSEE INRQVTYGIT GGDPLGQFAV ETIQNEWKVY

**6751** VKJGTVSEDD PQGGVIAILS TTDADSEEIN RQVTYSITGG DPLGQFAVET

**6801** IQNEWKVYVK JGTVSEDDPQ GGVIAILSTT DADSEEINRQ VTYFITGGDP

**6851** LAQFAVETIQ NEWKVYVKJA IVEVKVLDAN DNSPVCEYTL YSDTIPEDVL

**6901** PGKLIMQISA TDADIRJVLD ANDNSPVCEK TLYIDTIPED VLPGKLIMQI

**6951** SATDADIRJV LDANDNSPVC EKTLYTDTIP EDVLPGKLIM QISATDADIR

**7001** JATDGGGRFC QASIVLDLED VNDNAPEFSA DPYAITVFEN TEPGTLLTRV

**7051** QATDADAGLN RJKILYSLID SADGQFSINE LIGIIQLEKP LDRELQAVYT

**7101** LSLKJFSIDS KTGAVFIIEN LDYESSHEYY LTVEATDGGT PSLSDVATVN

**7151** VNVTDINDAT PVFSQDTYTT VISEDAVLEQ SVITVMADDA DGPSNSHIHY

**7201** SIIDGNQGSS FTIDPVRGEV KJFSIDSKTG AVFIIENLDY ESSHEYYLTV

**7251** EATDGGTPSL SDVATVNVNV TDINDVTPVF SQDTYTTVIS EDAVLEQSVI

**7301** TVMADDADGP SNSHIHYSII DGNQGSSFTI DPVRGEVKJV NTTTVNIDVS

**7351** DVNDNAPVFS RGNYSVIIQE NKPVGFSVLQ LVVTDEDSSH NGPPFFFTIV

**7401** TENDEKAFEV NPQGVLLTSS AIKJVNTTTV NIDVSDVNDN APVFSRGNYS

**7451** VIIQENKPVG FSVLQLVVTD EDSSHNGPPF FFTIVTGNDE KAFEVNPQGV

**7501** LLTSSAIKJG NYSVIIQENK PVGFSVLQLV VTDEDSSHNG PPFFFTIVTG

**7551** NDEKAFEVNA QGVLLTSSAI KRJGNYSVII QENKPVGFSV LQLVVTDEDS

**7601** SHNGPPFFFT IVTGNDEKAF EVNVQGVLLT SSAIKRJVAD NGKPQLSSLT

**7651** YIDIRVIEES IYPPAILPLE IAITSSGEEY SGGVIGKIHA TDQDVYDTLT

**7701** YSLDPQMDNL FSVSSTGGKJ VADNGKPQLS SLTYIDIRVI EESIYPPAIL

**7751** PLEISITSSG EEYSGGVIGK IHATDQDVYD TLTYSLDPQM DNLFSVSSTG

**7801** GKJVIHVTSP QFKAGPVKFE KJQLLHKIMS SVTDIEEIIG VRILNVFQKJ

**7851** QLLHKIVSSV TDIEEIIGVR ILNVFQKJEG RCPPVHHGCE DHPCPEGSEC

**7901** VSDPWEEKHT CVCPSGRJEG RCPPVHHGCE DPPCPEGSEC VSDPWEEKHT

**7951** CVCPSGRJHT CVCPSGRFGG CPGSSSMTLT GNSYVKYRJH TCVCPSGRFG

**8001** RCPGSSSMTL TGNSYVKYRJ FGQCPGSSSM TLTGNSYVKT RLTENENKJG

**8051** CMDSIYLNGQ ELPLNSKPRS YAHIEESVPV SPGCFLTATE DCASNPCQNG

**8101** GVCNPSPAGG YYCKCSALYI GTHCEISVNP CSSKPCLYGG TCVVDNGGFV

**8151** CQCRJYVFKR TPGKAKJYVF KSTPGKAKJS YAHIEESVDV SPGCFLTATE

**8201** DCASNPCQNG GVCNPSPAGG YYCKCSALYI GTHCEISVNP KSSKPCLYGG

**8251** TCVVDNGGFV CQCRGLYTGQ RJSYAHIEES VDVSPGCFLT ATEDCASNPC

**8301** QNGGVCNPSP AGGYYCKCSA LYIGTHCEIS VNPNSSKPCL YGGTCVVDNG

**8351** GFVCQCRGLY TGQRJDEPCK NGGTCFDSAD GAVCQCDSGF RGERJGRHCA

**8401** DAAPNQYVST PWNIGLAEGI GIVVFVAGIF LLVVVFVLCR KJGRHCEDAA

**8451** PNQYVSTPWN IGLAEGIGIV VFVAGIFLLV VVFVLCRKJN IYSDIPPQVP

**8501** VRYISYTPSI PSDSRNNLDR JKAVAVCSVA PNLPPPPPSN SPSDSDSIQK

**8551** PSWDFDYDTK VVDLDPCLSK JKPLEEKPSQ PYSARESLSS VQSLSSFQSE

**8601** SCDDNGYHWD TSDWMPSVPL PDIQEFPNYE VIDEQTPLYS ADPNAIDTDY

**8651** YPGGYDIESD FPPPPEDFPA ADELPPLPPE FSNQFESIHP PRDMPAAGSL

**8701** GSSSRJKPLE EKPSQPYSAR ESLSEVQSLS SFQSESCDDN GYHWDTSDWM

**8751** PSVPLQDIQE FPNYEVIDEQ TPLYSADPNA IDTDYYPGGY DIESDFPPPP

**8801** EDFPAADELP PLPPEFSNQF ESIHPPRDMP AAGSLGSSSR JKPLEEKPSQ

**8851** PYSARESLSE VQSLSSFQSE SCDDNGYHWD TSDWMPSVPL PDIQEFPNYE

**8901** VIDPQTPLYS ADPNAIDTDY YPGGYDIESD FPPPPEDFPA ADELPPLPPE

**8951** FSNQFESIHP PRDMPAAGSL GSSSRJESLS EVQSLSSFQS ESCDDNGYHW

**9001** DTSDWMPSVP LPDIQEFPNY EVIDEQTPLY SADPNAIDTD YYPGGYDIES

**9051** DFPPPPEDFP AADELPPLPP EFSNQFESIH PPRDMPAAGS LGSSRRNRJE

**9101** SLSEVQSLSS FQSESCDDNG YHWDTSDWMP SVPLPDIQEF PNYEVIDEQT

**9151** PLYSADPNAI DTDYYPGGYD IESDFPPPPE DFPAADELPP LPPEFSNQFE

**9201** SIHPPRDMPA AGSLGSSWRN RJVLGANSNP PEFTQTAYKA AFDENVPIGT

**9251** TIMSLSAVDP DEGENGYVTY SIANLNHVPF AIDHFTGAVS TSENLDYELM

**9301** PRVYTLRJVL GANSNPPEFT QTAYKAAFDE NVPIGTTVMS LSAVDPDEGE

**9351** NGYVTYSIAN LNHVPFAIDH FTGAVSTSEN LDYELMPRVY TLRJVLGANS

**9401** NPPEFTQTAY KAAFDENVPI GTTVMSLSAV DPDEGENGYV TYSIANLNHV

**9451** PFAIDHFTGA VSTSENLDYE LMPRVYTLRJ INCEGTIPRD LGVGEQITTV

**9501** SAIDADELQL VQYQIEAGNE LDFFSLNPNS GVLSLKRJIN CEGTIPRDLG

**9551** VGEQITTVSA IDADELQLVQ YQIEAGNELD LFSLNPNSGV LSLKRJINCE

**9601** GTIPRDLGVG EQITTVSAID ADELQLVQYQ IEAGNELDFF SLNPNSGVLS

**9651** LKRJEVHSEI IQVEATDKDL GPNGHVTYSI LTDTDTFSID SVTGVVNIAR

**9701** PLDRELQHEH SLKJEVHSEI IQVEATDKDL GPNGHVTYSI VTDTDTFSID

**9751** SVTGVVNIAR PLDRELQHEH SLK

**Start - End Observed Mr(expt) Mr(calc) ppm Miss Sequence**

**1658 - 1669 638.3514 1274.6883 1274.6870 1 0 R.IFVTIADNASPK.F**  ([Ions score 44](http://mbp-mascot4/mascot/cgi/peptide_view.pl?file=../data/20091021/F006288.dat&query=1806&hit=1&index=IPI00031411&px=1&section=5&ave_thresh=22))

**HPDE 3:**

Match to: **IPI00031411** Score: **148**

**Gene_Symbol=FAT1 Protocadherin Fat 1 lng=4591 # SP[4593,D,22,D]SNP[4646,g,1064,R]SNP[4677,r,1064,R]SNP[4713,i,1125,I]SNP[4791,l,1125,I]SNP[4844,i,1252,I]SNP[4856,h,1273,H]SNP[4889,r,1273,H]SNP[4931,l,1283,P]SNP[4969,p,1283,P]SNP[5000,l,129,V]SNP[5**

Found in search of C:\mgf\Orbidata\090430_ISW1295_HPDE_3.mgf

Nominal mass (M_r_): **1068827**; Calculated pI value: **4.55**

NCBI BLAST search of [IPI00031411](http://www.ncbi.nlm.nih.gov/blast/Blast.cgi?ALIGNMENTS=50&ALIGNMENT_VIEW=Pairwise&AUTO_FORMAT=Semiauto&CDD_SEARCH=on&CLIENT=web&COMPOSITION_BASED_STATISTICS=on&DATABASE=nr&DESCRIPTIONS=100&ENTREZ_QUERY=(none)&EXPECT=10&FILTER=L&FORMAT_BLOCK_ON_RESPAGE=None&FORMAT_OBJECT=Alignment&FORMAT_TYPE=HTML&GAPCOSTS=11+1&I_THRESH=0.001&LAYOUT=TwoWindows&MATRIX_NAME=BLOSUM62&NCBI_GI=on&PAGE=Proteins&PROGRAM=blastp&QUERY=IPI00031411&SERVICE=plain&SET_DEFAULTS.x=21&SET_DEFAULTS.y=7&SHOW_OVERVIEW=on&WORD_SIZE=3&END_OF_HTTPGET=Yes) against nr

Unformatted [sequence string](http://mbp-mascot4/mascot/cgi/getseq.pl?IPI_human+IPI00031411+seq) for pasting into other applications

Fixed modifications: Carbamidomethyl (C)

Variable modifications: Oxidation (M)

Cleavage by TrypsinMSIPI, a mixture of enzymes:

cuts C-term side of KR unless next residue is P

cuts C-term side of J

cuts N-term side of J

Sequence Coverage: **0%**

Matched peptides shown in **Bold Red**

**1** MGRHLALLLL LLLLFQHFGD SDGSQRLEQT PLQFTHLEYN VTVQENSAAK

**51** TYVGHPVKMG VYITHPAWEV RYK**IVSGDSE NLFK**AEEYIL GDFCFLRIRT

**101** KGGNTAILNR EVKDHYTLIV KALEKNTNVE ARTKVRVQVL DTNDLRPLFS

**151** PTSYSVSLPE NTAIRTSIAR VSATDADIGT NGEFYYSFKD RTDMFAIHPT

**201** SGVIVLTGRL DYLETK**LYEM EILAADR**GMK LYGSSGISSM AKLTVHIEQA

**251** NECAPVITAV TLSPSELDRD PAYAIVTVDD CDQGANGDIA SLSIVAGDLL

**301** QQFRTVRSFP GSKEYKVKAI GGIDWDSHPF GYNLTLQAKD KGTPPQFSSV

**351** KVIHVTSPQF KAGPVKFEKD VYRAEISEFA PPNTPVVMVK AIPAYSHLRY

**401** VFKSTPGKAK FSLNYNTGLI SILEPVKRQQ AAHFELEVTT SDRKASTKVL

**451** VKVLGANSNP PEFTQTAYKA AFDENVPIGT TVMSLSAVDP DEGENGYVTY

**501** SIANLNHVPF AIDHFTGAVS TSENLDYELM PRVYTLRIRA SDWGLPYRRE

**551** VEVLATITLN NLNDNTPLFE KINCEGTIPR DLGVGEQITT VSAIDADELQ

**601** LVQYQIEAGN ELDFFSLNPN SGVLSLKRSL MDGLGAKVSF HSLRITATDG

**651** ENFATPLYIN ITVAASHKLV NLQCEETGVA KMLAEKLLQA NKLHNQGEVE

**701** DIFFDSHSVN AHIPQFRSTL PTGIQVKENQ PVGSSVIFMN STDLDTGFNG

**751** KLVYAVSGGN EDSCFMIDME TGMLKILSPL DRETTDKYTL NITVYDLGIP

**801** QKAAWRLLHV VVVDANDNPP EFLQESYFVE VSEDKEVHSE IIQVEATDKD

**851** LGPNGHVTYS IVTDTDTFSI DSVTGVVNIA RPLDRELQHE HSLKIEARDQ

**901** AREEPQLFST VVVKVSLEDV NDNPPTFIPP NYRVKVREDL PEGTVIMWLE

**951** AHDPDLGQSG QVR**YSLLDHG EGNFDVDK**LS GAVR**IVQQLD FEK**KQVYNLT

**1001** VRAKDKGKPV SLSSTCYVEV EVVDVNENLH PPVFSSFVEK GTVKEDAPVG

**1051** SLVMTVSAHD EDARRDGEIR YSIRDGSGVG VFKIGEETGV IETSDRLDRE

**1101** STSHYWLTVF ATDQGVVPLS SFIEIYIEVE DVNDNAPQTS EPVYYPEIME

**1151** NSPKDVSVVQ IEAFDPDSSS NDKLMYKITS GNPQGFFSIH PKTGLITTTS

**1201** RKLDREQQDE HILEVTVTDN GSPPKSTIAR VIVKILDEND NKPQFLQKFY

**1251** KIRLPEREKP DRERNARREP LYHVIATDKD EGPNAEISYS IEDGNEHGKF

**1301** FIEPKTGVVS SKR**FSAAGEY DILSIK**AVDN GRPQKSSTTR LHIEWISKPK

**1351** PSLEPISFEE SFFTFTVMES DPVAHMIGVI SVEPPGIPLW FDITGGNYDS

**1401** HFDVDKGTGT IIVAKPLDAE QKSNYNLTVE ATDGTTTILT QVFIKVIDTN

**1451** DHRPQFSTSK YEVVIPEDTA PETEILQISA VDQDEKNKLI YTLQSSRDPL

**1501** SLKKFRLDPA TGSLYTSEKL DHEAVHQHTL TVMVRDQDVP VKRNFARIVV

**1551** NVSDTNDHAP WFTASSYKGR VYESAAVGSV VLQVTALDKD KGKNAEVLYS

**1601** IESGTFGNIG NSFMIDPVLG SIKTAKELDR SNQAEYDLMV KATDKGSPPM

**1651** SEITSVR**IFV TIADNASPK**F TSKEYSVELS ETVSIGSFVG MVTAHSQSSV

**1701** VYEIKDGNTG DAFDINPHSG TIITQKALDF ETLPIYTLII QGTNMAGLST

**1751** NTTVLVHLQD ENDNAPVFMQ AEYTGLISES ASINSVVLTD RNVPLVIRAA

**1801** DADKDSNALL VYHIVEPSVH TYFAIDSSTG AIHTVLSLDY EETSIFHFTV

**1851** QVHDMGTPRL FAEYAANVTV HVIDINDCPP VFAKPLYEAS LLLPTYKGVK

**1901** VITVNATDAD SSAFSQLIYS ITEGNIGEKF SMDYKTGALT VQNTTQLRSR

**1951** YELTVRASDG RFAGLTSVKI NVKESKESHL KFTQDVYSAV VKENSTEAET

**2001** LAVITAIGNP INEPLFYHIL NPDRRFKISR TSGVLSTTGT PFDREQQEAF

**2051** DVVVEVTEEH KPSAVAHVVV KVIVEDQNDN APVFVNLPYY AVVKVDTEVG

**2101** HVIRYVTAVD RDSGRNGEVH YYLKEHHEHF QIGPLGEISL KKQFELDTLN

**2151** KEYLVTVVAK DGGNPAFSAE VIVPITVMNK AMPVFEKPFY SAEIAESIQV

**2201** HSPVVHVQAN SPEGLKVFYS ITDGDPFSQF TINFNTGVIN VIAPLDFEAH

**2251** PAYKLSIRAT DSLTGAHAEV FVDIIVDDIN DNPPVFAQQS YAVTLSEASV

**2301** IGTSVVQVRA TDSDSEPNRG ISYQMFGNHS KSHDHFHVDS STGLISLLRT

**2351** LDYEQSRQHT IFVRAVDGGM PTLSSDVIVT VDVTDLNDNP PLFEQQIYEA

**2401** RISEHAPHGH FVTCVKAYDA DSSDIDKLQY SILSGNDHKH FVIDSATGII

**2451** TLSNLHRHAL KPFYSLNLSV SDGVFRSSTQ VHVTVIGGNL HSPAFLQNEY

**2501** EVELAENAPL HTLVMEVKTT DGDSGIYGHV TYHIVNDFAK DRFYINERGQ

**2551** IFTLEKLDRE TPAEKVISVR LMAKDAGGKV AFCTVNVILT DDNDNAPQFR

**2601** ATKYEVNIGS SAAKGTSVVK VLASDADEGS NADITYAIEA DSESVKENLE

**2651** INKLSGVITT KESLIGLENE FFTFFVRAVD NGSPSKESVV LVYVKILPPE

**2701** MQLPKFSEPF YTFTVSEDVP IGTEIDLIRA EHSGTVLYSL VKGNTPESNR

**2751** DESFVIDRQS GRLKLEKSLD HETTKWYQFS ILARCTQDDH EMVASVDVSI

**2801** QVKDANDNSP VFESSPYEAF IVENLPGGSR VIQIRASDAD SGTNGQVMYS

**2851** LDQSQSVEVI ESFAINMETG WITTLKELDH EKRDNYQIKV VASDHGEKIQ

**2901** LSSTAIVDVT VTDVNDSPPR FTAEIYKGTV SEDDPQGGVI AILSTTDADS

**2951** EEINRQVTYF ITGGDPLGQF AVETIQNEWK VYVKKPLDRE KRDNYLLTIT

**3001** ATDGTFSSKA IVEVKVLDAN DNSPVCEKTL YSDTIPEDVL PGK**LIMQISA**

**3051 TDADIR**SNAE ITYTLLGSGA EKFKLNPDTG ELKTSTPLDR EEQAVYHLLV

**3101** RATDGGGRFC QASIVLTLED VNDNAPEFSA DPYAITVFEN TEPGTLLTRV

**3151** QATDADAGLN RKILYSLIDS ADGQFSINEL SGIIQLEKPL DRELQAVYTL

**3201** SLKAVDQGLP RRLTATGTVI VSVLDINDNP PVFEYREYGA TVSEDILVGT

**3251** EVLQVYAASR DIEANAEITY SIISGNEHGK FSIDSKTGAV FIIENLDYES

**3301** SHEYYLTVEA TDGGTPSLSD VATVNVNVTD INDNTPVFSQ DTYTTVISED

**3351** AVLEQSVITV MADDADGPSN SHIHYSIIDG NQGSSFTIDP VRGEVKVTKL

**3401** LDRETISGYT LTVQASDNGS PPRVNTTTVN IDVSDVNDNA PVFSRGNYSV

**3451** IIQENKPVGF SVLQLVVTDE DSSHNGPPFF FTIVTGNDEK AFEVNPQGVL

**3501** LTSSAIKRKE KDHYLLQVKV ADNGKPQLSS LTYIDIRVIE ESIYPPAILP

**3551** LEIFITSSGE EYSGGVIGKI HATDQDVYDT LTYSLDPQMD NLFSVSSTGG

**3601** KLIAHKKLDI GQYLLNVSVT DGKFTTVADI TVHIRQVTQE MLNHTIAIRF

**3651** ANLTPEEFVG DYWRNFQRAL RNILGVRRND IQIVSLQSSE PHPHLDVLLF

**3701** VEKPGSAQIS TKQLLHKINS SVTDIEEIIG VRILNVFQKL CAGLDCPWKF

**3751** CDEKVSVDES VMSTHSTARL SFVTPRHHRA AVCLCKEGRC PPVHHGCEDD

**3801** PCPEGSECVS DPWEEKHTCV CPSGRFGQCP GSSSMTLTGN SYVKYRLTEN

**3851** ENKLEMKLTM RLRTYSTHAV VMYARGTDYS ILEIHHGRLQ YKFDCGSGPG

**3901** IVSVQSIQVN DGQWHAVALE VNGNYARLVL DQVHTASGTA PGTLKTLNLD

**3951** NYVFFGGHIR QQGTRHGRSP QVGNGFRGCM DSIYLNGQEL PLNSKPRSYA

**4001** HIEESVDVSP GCFLTATEDC ASNPCQNGGV CNPSPAGGYY CKCSALYIGT

**4051** HCEISVNPCS SKPCLYGGTC VVDNGGFVCQ CRGLYTGQRC QLSPYCKDEP

**4101** CKNGGTCFDS LDGAVCQCDS GFRGERCQSD IDECSGNPCL HGALCENTHG

**4151** SYHCNCSHEY RGRHCEDAAP NQYVSTPWNI GLAEGIGIVV FVAGIFLLVV

**4201** VFVLCRKMIS RKKKHQAEPK DKHLGPATAF LQRPYFDSKL NKNIYSDIPP

**4251** QVPVRPISYT PSIPSDSRNN LDRNSFEGSA IPEHPEFSTF NPESVHGHRK

**4301** AVAVCSVAPN LPPPPPSNSP SDSDSIQKPS WDFDYDTKVV DLDPCLSKKP

**4351** LEEKPSQPYS ARESLSEVQS LSSFQSESCD DNGYHWDTSD WMPSVPLPDI

**4401** QEFPNYEVID EQTPLYSADP NAIDTDYYPG GYDIESDFPP PPEDFPAADE

**4451** LPPLPPEFSN QFESIHPPRD MPAAGSLGSS SRNRQRFNLN QYLPNFYPLD

**4501** MSEPQTKGTG ENSTCREPHA PYPPGYQRHF EAPAVESMPM SVYASTASCS

**4551** DVSACCEVES EVMMSDYESG DDGHFEEVTI PPLDSQQHTE VJDGSQRLEQ

**4601** TPLQFTHLEY NVTVQENSAA KJGTVKEDAP VGSLVMTVSA HDEDAGRDGE

**4651** IRJGTVKEDA PVGSLVMTVS AHDEDARRDG EIRJLDREST SHYWLTVFAT

**4701** DQGVVPLSSF IEIYIEVEDV NDNAPQTSEP VYYPEIMENS PKDVSVVQIE

**4751** AFDPDSSSND KJLDRESTSH YWLTVFATDQ GVVPLSSFIE LYIEVEDVND

**4801** NAPQTSEPVY YPEIMENSPK DVSVVQIEAF DPDSSSNDKJ FYKIRLPERJ

**4851** REPLYHVIAT DKDEGPNAEI SYSIEDGNEH GKJREPLYRV IATDKDEGPN

**4901** AEISYSIEDG NEHGKJEPLY HVIATDKDEG LNAEISYSIE DGNEHGKFFI

**4951** EPKJEPLYHV IATDKDEGPN AEISYSIEDG NEHGKFFIEP KJALEKNTNL

**5001** EARTKJALEK NTNVEARTKJ ALEKNTNVEA RTKJALEKNT NVEVRTKJFS

**5051** AAGEYDILSI KAVDNGRPQK SSTTRJFSAA GEYDILSIKA VDSGRPQKSS

**5101** TTRJNFARIV VNVSDTNDHA PWFTASSYKG RJNFARIVVN VSDTNDHAPW

**5151** FTTSSYKGRJ GKNAEVLYSI ESGDFGNIGN SFMIDPVLGS IKTAKJGKNA

**5201** EVLYSIESGN FGNIGNSFMI DPVLGSIKTA KJGKNAEVLY SIESGTIGNI

**5251** GNSFMIDPVL GSIKTAKJGS PPMSEITSVR IFVTNADNAS PKFTSKJGSP

**5301** PMSEITSVRI FVTSADNASP KFTSKJGSPP MSEITSVRIF VTIADNASTK

**5351** FTSKJALDFE TLPIYTLIIQ GTNMAGLSTN TTVLVHLQDE NDNAPVFMQA

**5401** EYTGLISESA SINSVVLTDR NVPQVIRAAD ADKJALDFET LPIYTLIIQG

**5451** TNMAGLSTNT TVLVHLQDEN DNAPVFMQAE YTGLISESAS INSVVLTDRN

**5501** VPRVIRAADA DKJAADADKD SNVLLVYHIV EPSVHTYFAI DSSTGAIHTV

**5551** LSLDYEETSI FHFTVQVHDM GTPRLFAEYA ANVTVHVIDI NDCPPVFAKP

**5601** LYEASLLLPT YKJFTQDVYS AVVKTNSTEA ETLAVITAIG NPINEPLFYH

**5651** ILNPDRRJTS GVLSTTGTPF DREAQEAFDV VVEVTEEHKP SAVAHVVVKV

**5701** IVEDQNDNAP VFVNLPYYAV VKJTDMFAIH PTSGVIVLTG RLDFLETKLY

**5751** EMEILAADRJ TDMFAIHPTS GVIVLTGRLD YLETKLYEME ILAADRJNGE

**5801** VHYYLKEHHE HFQIGPLGEK SLKKJDGGNP AFSAEVIVPI TVMNKAMPVF

**5851** EKPFYSAEIA ESIQVHSHVV HVQANSPEGL KVFYSITDGD PFSQFTINFN

**5901** TGVINVIAPL DFEAHPAYKJ AMPVFEKPFY SAEIAESIQV HSPVVHVQAN

**5951** SPEGLKVFYS ITDGDPFSQF TINFNTGVIN VIAPLDFPAH PAYKLSIRJL

**6001** SIRATDSLTG AHAEVFVDDI VDDINDNPPV FAQQSYAVTL SEASVIGTSV

**6051** VQVRATDSDS EPNRJLSIRA TDSLTGAHAE VFVDEIVDDI NDNPPVFAQQ

**6101** SYAVTLSEAS VIGTSVVQVR ATDSDSEPNR JTLDYEQSRQ HTIAVRAVDG

**6151** GMPTLSSDVI VTVDVTDLND NPPLFEQQIY EARJLYGSSG ISSMAKLTVH

**6201** IEQANECAPV ITAVTLSPSE LDRDPAYAIV TVDDCDQGAN GDIASLSIVA

**6251** GDLLQQFRJI LPPEMQLPKF SEPFYTFTVS EIVPIGTEID LIRAEHSGTV

**6301** LYSLVKJILP PEMQLPKFSE PFYTFTVSEV VPIGTEIDLI RAEHSGTVLY

**6351** SLVKJCTQDD HEMVASVDVS IQVKDANDNS PVFESSPYEA FIVENLPGGS

**6401** RVIQIRJCTQ DDHEMVASVD VSIQVKDASD NSPVFESSPY EAFIVENLPG

**6451** GSRVIQIRJV IQIRASDADN GTNGQVMYSL DQSQSVEVIE SFAINMETGW

**6501** ITTLKELDHE KJFTAEIYKG TVSELDPQGG VIAILSTTDA DSEEINRQVT

**6551** YFITGGDPLG QFAVETIQNE WKJFTAEIYK GTVSEPDPQG GVIAILSTTD

**6601** ADSEEINRQV TYFITGGDPL GQFAVETIQN EWKJFTAEIY KGTVSEQDPQ

**6651** GGVIAILSTT DADSEEINRQ VTYFITGGDP LGQFAVETIQ NEWKJGTVSE

**6701** DDPQGGVIAI LSTTDADSEE INRQVTYGIT GGDPLGQFAV ETIQNEWKVY

**6751** VKJGTVSEDD PQGGVIAILS TTDADSEEIN RQVTYSITGG DPLGQFAVET

**6801** IQNEWKVYVK JGTVSEDDPQ GGVIAILSTT DADSEEINRQ VTYFITGGDP

**6851** LAQFAVETIQ NEWKVYVKJA IVEVKVLDAN DNSPVCEYTL YSDTIPEDVL

**6901** PGKLIMQISA TDADIRJVLD ANDNSPVCEK TLYIDTIPED VLPGKLIMQI

**6951** SATDADIRJV LDANDNSPVC EKTLYTDTIP EDVLPGKLIM QISATDADIR

**7001** JATDGGGRFC QASIVLDLED VNDNAPEFSA DPYAITVFEN TEPGTLLTRV

**7051** QATDADAGLN RJKILYSLID SADGQFSINE LIGIIQLEKP LDRELQAVYT

**7101** LSLKJFSIDS KTGAVFIIEN LDYESSHEYY LTVEATDGGT PSLSDVATVN

**7151** VNVTDINDAT PVFSQDTYTT VISEDAVLEQ SVITVMADDA DGPSNSHIHY

**7201** SIIDGNQGSS FTIDPVRGEV KJFSIDSKTG AVFIIENLDY ESSHEYYLTV

**7251** EATDGGTPSL SDVATVNVNV TDINDVTPVF SQDTYTTVIS EDAVLEQSVI

**7301** TVMADDADGP SNSHIHYSII DGNQGSSFTI DPVRGEVKJV NTTTVNIDVS

**7351** DVNDNAPVFS RGNYSVIIQE NKPVGFSVLQ LVVTDEDSSH NGPPFFFTIV

**7401** TENDEKAFEV NPQGVLLTSS AIKJVNTTTV NIDVSDVNDN APVFSRGNYS

**7451** VIIQENKPVG FSVLQLVVTD EDSSHNGPPF FFTIVTGNDE KAFEVNPQGV

**7501** LLTSSAIKJG NYSVIIQENK PVGFSVLQLV VTDEDSSHNG PPFFFTIVTG

**7551** NDEKAFEVNA QGVLLTSSAI KRJGNYSVII QENKPVGFSV LQLVVTDEDS

**7601** SHNGPPFFFT IVTGNDEKAF EVNVQGVLLT SSAIKRJVAD NGKPQLSSLT

**7651** YIDIRVIEES IYPPAILPLE IAITSSGEEY SGGVIGKIHA TDQDVYDTLT

**7701** YSLDPQMDNL FSVSSTGGKJ VADNGKPQLS SLTYIDIRVI EESIYPPAIL

**7751** PLEISITSSG EEYSGGVIGK IHATDQDVYD TLTYSLDPQM DNLFSVSSTG

**7801** GKJVIHVTSP QFKAGPVKFE KJQLLHKIMS SVTDIEEIIG VRILNVFQKJ

**7851** QLLHKIVSSV TDIEEIIGVR ILNVFQKJEG RCPPVHHGCE DHPCPEGSEC

**7901** VSDPWEEKHT CVCPSGRJEG RCPPVHHGCE DPPCPEGSEC VSDPWEEKHT

**7951** CVCPSGRJHT CVCPSGRFGG CPGSSSMTLT GNSYVKYRJH TCVCPSGRFG

**8001** RCPGSSSMTL TGNSYVKYRJ FGQCPGSSSM TLTGNSYVKT RLTENENKJG

**8051** CMDSIYLNGQ ELPLNSKPRS YAHIEESVPV SPGCFLTATE DCASNPCQNG

**8101** GVCNPSPAGG YYCKCSALYI GTHCEISVNP CSSKPCLYGG TCVVDNGGFV

**8151** CQCRJYVFKR TPGKAKJYVF KSTPGKAKJS YAHIEESVDV SPGCFLTATE

**8201** DCASNPCQNG GVCNPSPAGG YYCKCSALYI GTHCEISVNP KSSKPCLYGG

**8251** TCVVDNGGFV CQCRGLYTGQ RJSYAHIEES VDVSPGCFLT ATEDCASNPC

**8301** QNGGVCNPSP AGGYYCKCSA LYIGTHCEIS VNPNSSKPCL YGGTCVVDNG

**8351** GFVCQCRGLY TGQRJDEPCK NGGTCFDSAD GAVCQCDSGF RGERJGRHCA

**8401** DAAPNQYVST PWNIGLAEGI GIVVFVAGIF LLVVVFVLCR KJGRHCEDAA

**8451** PNQYVSTPWN IGLAEGIGIV VFVAGIFLLV VVFVLCRKJN IYSDIPPQVP

**8501** VRYISYTPSI PSDSRNNLDR JKAVAVCSVA PNLPPPPPSN SPSDSDSIQK

**8551** PSWDFDYDTK VVDLDPCLSK JKPLEEKPSQ PYSARESLSS VQSLSSFQSE

**8601** SCDDNGYHWD TSDWMPSVPL PDIQEFPNYE VIDEQTPLYS ADPNAIDTDY

**8651** YPGGYDIESD FPPPPEDFPA ADELPPLPPE FSNQFESIHP PRDMPAAGSL

**8701** GSSSRJKPLE EKPSQPYSAR ESLSEVQSLS SFQSESCDDN GYHWDTSDWM

**8751** PSVPLQDIQE FPNYEVIDEQ TPLYSADPNA IDTDYYPGGY DIESDFPPPP

**8801** EDFPAADELP PLPPEFSNQF ESIHPPRDMP AAGSLGSSSR JKPLEEKPSQ

**8851** PYSARESLSE VQSLSSFQSE SCDDNGYHWD TSDWMPSVPL PDIQEFPNYE

**8901** VIDPQTPLYS ADPNAIDTDY YPGGYDIESD FPPPPEDFPA ADELPPLPPE

**8951** FSNQFESIHP PRDMPAAGSL GSSSRJESLS EVQSLSSFQS ESCDDNGYHW

**9001** DTSDWMPSVP LPDIQEFPNY EVIDEQTPLY SADPNAIDTD YYPGGYDIES

**9051** DFPPPPEDFP AADELPPLPP EFSNQFESIH PPRDMPAAGS LGSSRRNRJE

**9101** SLSEVQSLSS FQSESCDDNG YHWDTSDWMP SVPLPDIQEF PNYEVIDEQT

**9151** PLYSADPNAI DTDYYPGGYD IESDFPPPPE DFPAADELPP LPPEFSNQFE

**9201** SIHPPRDMPA AGSLGSSWRN RJVLGANSNP PEFTQTAYKA AFDENVPIGT

**9251** TIMSLSAVDP DEGENGYVTY SIANLNHVPF AIDHFTGAVS TSENLDYELM

**9301** PRVYTLRJVL GANSNPPEFT QTAYKAAFDE NVPIGTTVMS LSAVDPDEGE

**9351** NGYVTYSIAN LNHVPFAIDH FTGAVSTSEN LDYELMPRVY TLRJVLGANS

**9401** NPPEFTQTAY KAAFDENVPI GTTVMSLSAV DPDEGENGYV TYSIANLNHV

**9451** PFAIDHFTGA VSTSENLDYE LMPRVYTLRJ INCEGTIPRD LGVGEQITTV

**9501** SAIDADELQL VQYQIEAGNE LDFFSLNPNS GVLSLKRJIN CEGTIPRDLG

**9551** VGEQITTVSA IDADELQLVQ YQIEAGNELD LFSLNPNSGV LSLKRJINCE

**9601** GTIPRDLGVG EQITTVSAID ADELQLVQYQ IEAGNELDFF SLNPNSGVLS

**9651** LKRJEVHSEI IQVEATDKDL GPNGHVTYSI LTDTDTFSID SVTGVVNIAR

**9701** PLDRELQHEH SLKJEVHSEI IQVEATDKDL GPNGHVTYSI VTDTDTFSID

**9751** SVTGVVNIAR PLDRELQHEH SLK

**Start - End Observed Mr(expt) Mr(calc) ppm Miss Sequence**

**74 - 84 604.8118 1207.6090 1207.6085 0 0 K.IVSGDSENLFK.A**  ([Ions score 24](http://mbp-mascot4/mascot/cgi/peptide_view.pl?file=../data/20091021/F006290.dat&query=1398&hit=1&index=IPI00031411&px=1&section=5&ave_thresh=22))

**217 - 227 662.3353 1322.6561 1322.6540 2 0 K.LYEMEILAADR.G**  ([Ions score 47](http://mbp-mascot4/mascot/cgi/peptide_view.pl?file=../data/20091021/F006290.dat&query=1989&hit=1&index=IPI00031411&px=1&section=5&ave_thresh=22))

**964 - 978 570.2658 1707.7756 1707.7740 1 0 R.YSLLDHGEGNFDVDK.L**  ([Ions score 21](http://mbp-mascot4/mascot/cgi/peptide_view.pl?file=../data/20091021/F006290.dat&query=3112&hit=1&index=IPI00031411&px=1&section=5&ave_thresh=22))

**985 - 993 560.3062 1118.5979 1118.5972 1 0 R.IVQQLDFEK.K**  ([Ions score 23](http://mbp-mascot4/mascot/cgi/peptide_view.pl?file=../data/20091021/F006290.dat&query=997&hit=1&index=IPI00031411&px=1&section=5&ave_thresh=22))

**1314 - 1326 707.3679 1412.7212 1412.7187 2 0 R.FSAAGEYDILSIK.A**  ([Ions score 54](http://mbp-mascot4/mascot/cgi/peptide_view.pl?file=../data/20091021/F006290.dat&query=2272&hit=1&index=IPI00031411&px=1&section=5&ave_thresh=22))

**1658 - 1669 638.3511 1274.6877 1274.6870 1 0 R.IFVTIADNASPK.F**  ([Ions score 46](http://mbp-mascot4/mascot/cgi/peptide_view.pl?file=../data/20091021/F006290.dat&query=1734&hit=1&index=IPI00031411&px=1&section=5&ave_thresh=22))

**3044 - 3056 723.8849 1445.7553 1445.7548 0 0 K.LIMQISATDADIR.S**  ([Ions score 65](http://mbp-mascot4/mascot/cgi/peptide_view.pl?file=../data/20091021/F006290.dat&query=2390&hit=1&index=IPI00031411&px=1&section=5&ave_thresh=22))

**MiaPaCa A2:**

Match to: **IPI00031411** Score: **662**

**Gene_Symbol=FAT1 Protocadherin Fat 1 lng=4591 # SP[4593,D,22,D]SNP[4646,g,1064,R]SNP[4677,r,1064,R]SNP[4713,i,1125,I]SNP[4791,l,1125,I]SNP[4844,i,1252,I]SNP[4856,h,1273,H]SNP[4889,r,1273,H]SNP[4931,l,1283,P]SNP[4969,p,1283,P]SNP[5000,l,129,V]SNP[5**

Found in search of C:\mgf\Orbidata\081126_ISW1295_MiaPaca2_A2.mgf

Nominal mass (M_r_): **1068827**; Calculated pI value: **4.55**

NCBI BLAST search of [IPI00031411](http://www.ncbi.nlm.nih.gov/blast/Blast.cgi?ALIGNMENTS=50&ALIGNMENT_VIEW=Pairwise&AUTO_FORMAT=Semiauto&CDD_SEARCH=on&CLIENT=web&COMPOSITION_BASED_STATISTICS=on&DATABASE=nr&DESCRIPTIONS=100&ENTREZ_QUERY=(none)&EXPECT=10&FILTER=L&FORMAT_BLOCK_ON_RESPAGE=None&FORMAT_OBJECT=Alignment&FORMAT_TYPE=HTML&GAPCOSTS=11+1&I_THRESH=0.001&LAYOUT=TwoWindows&MATRIX_NAME=BLOSUM62&NCBI_GI=on&PAGE=Proteins&PROGRAM=blastp&QUERY=IPI00031411&SERVICE=plain&SET_DEFAULTS.x=21&SET_DEFAULTS.y=7&SHOW_OVERVIEW=on&WORD_SIZE=3&END_OF_HTTPGET=Yes) against nr

Unformatted [sequence string](http://mbp-mascot4/mascot/cgi/getseq.pl?IPI_human+IPI00031411+seq) for pasting into other applications

Fixed modifications: Carbamidomethyl (C)

Variable modifications: Oxidation (M)

Cleavage by TrypsinMSIPI, a mixture of enzymes:

cuts C-term side of KR unless next residue is P

cuts C-term side of J

cuts N-term side of J

Sequence Coverage: **5%**

Matched peptides shown in **Bold Red**

**1** MGRHLALLLL LLLLFQHFGD SDGSQRLEQT PLQFTHLEYN VTVQENSAAK

**51** TYVGHPVKMG VYITHPAWEV RYK**IVSGDSE NLFKAEEYIL GDFCFLR**IRT

**101** KGGNTAILNR EVKDHYTLIV KALEKNTNVE ARTKVRVQVL DTNDLRPLFS

**151** PTSYSVSLPE NTAIRTSIAR VSATDADIGT NGEFYYSFKD RTDMFAIHPT

**201** SGVIVLTGRL DYLETK**LYEM EILAADR**GMK LYGSSGISSM AKLTVHIEQA

**251** NECAPVITAV TLSPSELDRD PAYAIVTVDD CDQGANGDIA SLSIVAGDLL

**301** QQFRTVRSFP GSKEYKVKAI GGIDWDSHPF GYNLTLQAKD KGTPPQFSSV

**351** KVIHVTSPQF KAGPVKFEKD VYR**AEISEFA PPNTPVVMVK** AIPAYSHLRY

**401** VFKSTPGKAK FSLNYNTGLI SILEPVKRQQ AAHFELEVTT SDRKASTKVL

**451** VK**VLGANSNP PEFTQTAYK**A AFDENVPIGT TVMSLSAVDP DEGENGYVTY

**501** SIANLNHVPF AIDHFTGAVS TSENLDYELM PRVYTLRIRA SDWGLPYRRE

**551** VEVLATITLN NLNDNTPLFE KINCEGTIPR DLGVGEQITT VSAIDADELQ

**601** LVQYQIEAGN ELDFFSLNPN SGVLSLKRSL MDGLGAKVSF HSLRITATDG

**651** ENFATPLYIN ITVAASHK**LV NLQCEETGVA K**MLAEKLLQA NKLHNQGEVE

**701** DIFFDSHSVN AHIPQFRSTL PTGIQVKENQ PVGSSVIFMN STDLDTGFNG

**751** KLVYAVSGGN EDSCFMIDME TGMLKILSPL DRETTDKYTL NITVYDLGIP

**801** QKAAWRLLHV VVVDANDNPP EFLQESYFVE VSEDKEVHSE IIQVEATDKD

**851** LGPNGHVTYS IVTDTDTFSI DSVTGVVNIA RPLDRELQHE HSLKIEARDQ

**901** AR**EEPQLFST VVVK**VSLEDV NDNPPTFIPP NYRVKVREDL PEGTVIMWLE

**951** AHDPDLGQSG QVRYSLLDHG EGNFDVDKLS GAVR**IVQQLD FEK**KQVYNLT

**1001** VRAKDKGKPV SLSSTCYVEV EVVDVNENLH PPVFSSFVEK GTVKEDAPVG

**1051** SLVMTVSAHD EDARRDGEIR YSIRDGSGVG VFK**IGEETGV IETSDRLDR**E

**1101** STSHYWLTVF ATDQGVVPLS SFIEIYIEVE DVNDNAPQTS EPVYYPEIME

**1151** NSPKDVSVVQ IEAFDPDSSS NDKLMYKITS GNPQGFFSIH PKTGLITTTS

**1201** RKLDREQQDE HILEVTVTDN GSPPKSTIAR VIVKILDEND NKPQFLQKFY

**1251** KIRLPEREKP DRERNARREP LYHVIATDKD EGPNAEISYS IEDGNEHGK**F**

**1301 FIEPK**TGVVS SKR**FSAAGEY DILSIK**AVDN GRPQKSSTTR LHIEWISKPK

**1351** PSLEPISFEE SFFTFTVMES DPVAHMIGVI SVEPPGIPLW FDITGGNYDS

**1401** HFDVDK**GTGT IIVAKPLDAE QK**SNYNLTVE ATDGTTTILT QVFIKVIDTN

**1451** DHRPQFSTSK YEVVIPEDTA PETEILQISA VDQDEKNK**LI YTLQSSR**DPL

**1501** SLKKFR**LDPA TGSLYTSEK**L DHEAVHQHTL TVMVRDQDVP VKRNFARIVV

**1551** NVSDTNDHAP WFTASSYKGR VYESAAVGSV VLQVTALDKD KGKNAEVLYS

**1601** IESGTFGNIG NSFMIDPVLG SIKTAKELDR SNQAEYDLMV KATDK**GSPPM**

**1651 SEITSVRIFV TIADNASPK**F TSKEYSVELS ETVSIGSFVG MVTAHSQSSV

**1701** VYEIK**DGNTG DAFDINPHSG TIITQK**ALDF ETLPIYTLII QGTNMAGLST

**1751** NTTVLVHLQD ENDNAPVFMQ AEYTGLISES ASINSVVLTD RNVPLVIRAA

**1801** DADKDSNALL VYHIVEPSVH TYFAIDSSTG AIHTVLSLDY EETSIFHFTV

**1851** QVHDMGTPRL FAEYAANVTV HVIDINDCPP VFAKPLYEAS LLLPTYKGVK

**1901** VITVNATDAD SSAFSQLIYS ITEGNIGEKF SMDYKTGALT VQNTTQLRSR

**1951** YELTVRASDG RFAGLTSVKI NVKESKESHL K**FTQDVYSAV VK**ENSTEAET

**2001** LAVITAIGNP INEPLFYHIL NPDRRFKISR **TSGVLSTTGT PFDR**EQQEAF

**2051** DVVVEVTEEH KPSAVAHVVV K**VIVEDQNDN APVFVNLPYY AVVK**VDTEVG

**2101** HVIRYVTAVD RDSGRNGEVH YYLK**EHHEHF QIGPLGEISL K**KQFELDTLN

**2151** KEYLVTVVAK DGGNPAFSAE VIVPITVMNK AMPVFEKPFY SAEIAESIQV

**2201** HSPVVHVQAN SPEGLKVFYS ITDGDPFSQF TINFNTGVIN VIAPLDFEAH

**2251** PAYKLSIRAT DSLTGAHAEV FVDIIVDDIN DNPPVFAQQS YAVTLSEASV

**2301** IGTSVVQVRA TDSDSEPNRG ISYQMFGNHS K**SHDHFHVDS STGLISLLR**T

**2351** LDYEQSRQHT IFVRAVDGGM PTLSSDVIVT VDVTDLNDNP PLFEQQIYEA

**2401** RISEHAPHGH FVTCVKAYDA DSSDIDKLQY SILSGNDHK**H FVIDSATGII**

**2451 TLSNLHR**HAL KPFYSLNLSV SDGVFRSSTQ VHVTVIGGNL HSPAFLQNEY

**2501** EVELAENAPL HTLVMEVKTT DGDSGIYGHV TYHIVNDFAK DRFYINERGQ

**2551** IFTLEKLDRE TPAEKVISVR LMAKDAGGKV AFCTVNVILT DDNDNAPQFR

**2601** ATK**YEVNIGS SAAK**GTSVVK VLASDADEGS NADITYAIEA DSESVKENLE

**2651** INKLSGVITT KESLIGLENE FFTFFVRAVD NGSPSKESVV LVYVKILPPE

**2701** MQLPKFSEPF YTFTVSEDVP IGTEIDLIRA EHSGTVLYSL VK**GNTPESNR**

**2751 DESFVIDR**QS GRLKLEKSLD HETTK**WYQFS ILAR**CTQDDH EMVASVDVSI

**2801** QVKDANDNSP VFESSPYEAF IVENLPGGSR VIQIRASDAD SGTNGQVMYS

**2851** LDQSQSVEVI ESFAINMETG WITTLKELDH EKRDNYQIKV VASDHGEKIQ

**2901** LSSTAIVDVT VTDVNDSPPR FTAEIYKGTV SEDDPQGGVI AILSTTDADS

**2951** EEINRQVTYF ITGGDPLGQF AVETIQNEWK VYVKKPLDRE KRDNYLLTIT

**3001** ATDGTFSSKA IVEVKVLDAN DNSPVCEK**TL YSDTIPEDVL PGKLIMQISA**

**3051 TDADIRSNAE ITYTLLGSGA EK**FKLNPDTG ELK**TSTPLDR EEQAVYHLLV**

**3101 R**ATDGGGRFC QASIVLTLED VNDNAPEFSA DPYAITVFEN TEPGTLLTRV

**3151** QATDADAGLN RKILYSLIDS ADGQFSINEL SGIIQLEKPL DRELQAVYTL

**3201** SLKAVDQGLP RRLTATGTVI VSVLDINDNP PVFEYREYGA TVSEDILVGT

**3251** EVLQVYAASR DIEANAEITY SIISGNEHGK FSIDSKTGAV FIIENLDYES

**3301** SHEYYLTVEA TDGGTPSLSD VATVNVNVTD INDNTPVFSQ DTYTTVISED

**3351** AVLEQSVITV MADDADGPSN SHIHYSIIDG NQGSSFTIDP VRGEVKVTKL

**3401** LDRETISGYT LTVQASDNGS PPRVNTTTVN IDVSDVNDNA PVFSRGNYSV

**3451** IIQENKPVGF SVLQLVVTDE DSSHNGPPFF FTIVTGNDEK **AFEVNPQGVL**

**3501 LTSSAIK**RKE KDHYLLQVK**V ADNGKPQLSS LTYIDIR**VIE ESIYPPAILP

**3551** LEIFITSSGE EYSGGVIGKI HATDQDVYDT LTYSLDPQMD NLFSVSSTGG

**3601** KLIAHKKLDI GQYLLNVSVT DGKFTTVADI TVHIRQVTQE MLNHTIAIR**F**

**3651 ANLTPEEFVG DYWR**NFQRAL RNILGVRRND IQIVSLQSSE PHPHLDVLLF

**3701** VEKPGSAQIS TKQLLHKINS SVTDIEEIIG VRILNVFQKL CAGLDCPWKF

**3751** CDEKVSVDES VMSTHSTARL SFVTPRHHRA AVCLCKEGRC PPVHHGCEDD

**3801** PCPEGSECVS DPWEEKHTCV CPSGRFGQCP GSSSMTLTGN SYVKYRLTEN

**3851** ENKLEMKLTM RLRTYSTHAV VMYAR**GTDYS ILEIHHGR**LQ YKFDCGSGPG

**3901** IVSVQSIQVN DGQWHAVALE VNGNYAR**LVL DQVHTASGTA PGTLK**TLNLD

**3951** NYVFFGGHIR QQGTRHGRSP QVGNGFRGCM DSIYLNGQEL PLNSKPRSYA

**4001** HIEESVDVSP GCFLTATEDC ASNPCQNGGV CNPSPAGGYY CKCSALYIGT

**4051** HCEISVNPCS SKPCLYGGTC VVDNGGFVCQ CRGLYTGQRC QLSPYCKDEP

**4101** CKNGGTCFDS LDGAVCQCDS GFRGERCQSD IDECSGNPCL HGALCENTHG

**4151** SYHCNCSHEY RGRHCEDAAP NQYVSTPWNI GLAEGIGIVV FVAGIFLLVV

**4201** VFVLCRKMIS RKKKHQAEPK DKHLGPATAF LQRPYFDSKL NKNIYSDIPP

**4251** QVPVRPISYT PSIPSDSRNN LDRNSFEGSA IPEHPEFSTF NPESVHGHRK

**4301** AVAVCSVAPN LPPPPPSNSP SDSDSIQKPS WDFDYDTKVV DLDPCLSKKP

**4351** LEEKPSQPYS ARESLSEVQS LSSFQSESCD DNGYHWDTSD WMPSVPLPDI

**4401** QEFPNYEVID EQTPLYSADP NAIDTDYYPG GYDIESDFPP PPEDFPAADE

**4451** LPPLPPEFSN QFESIHPPRD MPAAGSLGSS SRNRQRFNLN QYLPNFYPLD

**4501** MSEPQTKGTG ENSTCREPHA PYPPGYQRHF EAPAVESMPM SVYASTASCS

**4551** DVSACCEVES EVMMSDYESG DDGHFEEVTI PPLDSQQHTE VJDGSQRLEQ

**4601** TPLQFTHLEY NVTVQENSAA KJGTVKEDAP VGSLVMTVSA HDEDAGRDGE

**4651** IRJGTVKEDA PVGSLVMTVS AHDEDARRDG EIRJLDREST SHYWLTVFAT

**4701** DQGVVPLSSF IEIYIEVEDV NDNAPQTSEP VYYPEIMENS PKDVSVVQIE

**4751** AFDPDSSSND KJLDRESTSH YWLTVFATDQ GVVPLSSFIE LYIEVEDVND

**4801** NAPQTSEPVY YPEIMENSPK DVSVVQIEAF DPDSSSNDKJ FYKIRLPERJ

**4851** REPLYHVIAT DKDEGPNAEI SYSIEDGNEH GKJREPLYRV IATDKDEGPN

**4901** AEISYSIEDG NEHGKJEPLY HVIATDKDEG LNAEISYSIE DGNEHGKFFI

**4951** EPKJEPLYHV IATDKDEGPN AEISYSIEDG NEHGKFFIEP KJALEKNTNL

**5001** EARTKJALEK NTNVEARTKJ ALEKNTNVEA RTKJALEKNT NVEVRTKJFS

**5051** AAGEYDILSI KAVDNGRPQK SSTTRJFSAA GEYDILSIKA VDSGRPQKSS

**5101** TTRJNFARIV VNVSDTNDHA PWFTASSYKG RJNFARIVVN VSDTNDHAPW

**5151** FTTSSYKGRJ GKNAEVLYSI ESGDFGNIGN SFMIDPVLGS IKTAKJGKNA

**5201** EVLYSIESGN FGNIGNSFMI DPVLGSIKTA KJGKNAEVLY SIESGTIGNI

**5251** GNSFMIDPVL GSIKTAKJGS PPMSEITSVR IFVTNADNAS PKFTSKJGSP

**5301** PMSEITSVRI FVTSADNASP KFTSKJGSPP MSEITSVRIF VTIADNASTK

**5351** FTSKJALDFE TLPIYTLIIQ GTNMAGLSTN TTVLVHLQDE NDNAPVFMQA

**5401** EYTGLISESA SINSVVLTDR NVPQVIRAAD ADKJALDFET LPIYTLIIQG

**5451** TNMAGLSTNT TVLVHLQDEN DNAPVFMQAE YTGLISESAS INSVVLTDRN

**5501** VPRVIRAADA DKJAADADKD SNVLLVYHIV EPSVHTYFAI DSSTGAIHTV

**5551** LSLDYEETSI FHFTVQVHDM GTPRLFAEYA ANVTVHVIDI NDCPPVFAKP

**5601** LYEASLLLPT YKJFTQDVYS AVVKTNSTEA ETLAVITAIG NPINEPLFYH

**5651** ILNPDRRJTS GVLSTTGTPF DREAQEAFDV VVEVTEEHKP SAVAHVVVKV

**5701** IVEDQNDNAP VFVNLPYYAV VKJTDMFAIH PTSGVIVLTG RLDFLETKLY

**5751** EMEILAADRJ TDMFAIHPTS GVIVLTGRLD YLETKLYEME ILAADRJNGE

**5801** VHYYLKEHHE HFQIGPLGEK SLKKJDGGNP AFSAEVIVPI TVMNKAMPVF

**5851** EKPFYSAEIA ESIQVHSHVV HVQANSPEGL KVFYSITDGD PFSQFTINFN

**5901** TGVINVIAPL DFEAHPAYKJ AMPVFEKPFY SAEIAESIQV HSPVVHVQAN

**5951** SPEGLKVFYS ITDGDPFSQF TINFNTGVIN VIAPLDFPAH PAYKLSIRJL

**6001** SIRATDSLTG AHAEVFVDDI VDDINDNPPV FAQQSYAVTL SEASVIGTSV

**6051** VQVRATDSDS EPNRJLSIRA TDSLTGAHAE VFVDEIVDDI NDNPPVFAQQ

**6101** SYAVTLSEAS VIGTSVVQVR ATDSDSEPNR JTLDYEQSRQ HTIAVRAVDG

**6151** GMPTLSSDVI VTVDVTDLND NPPLFEQQIY EARJLYGSSG ISSMAKLTVH

**6201** IEQANECAPV ITAVTLSPSE LDRDPAYAIV TVDDCDQGAN GDIASLSIVA

**6251** GDLLQQFRJI LPPEMQLPKF SEPFYTFTVS EIVPIGTEID LIRAEHSGTV

**6301** LYSLVKJILP PEMQLPKFSE PFYTFTVSEV VPIGTEIDLI RAEHSGTVLY

**6351** SLVKJCTQDD HEMVASVDVS IQVKDANDNS PVFESSPYEA FIVENLPGGS

**6401** RVIQIRJCTQ DDHEMVASVD VSIQVKDASD NSPVFESSPY EAFIVENLPG

**6451** GSRVIQIRJV IQIRASDADN GTNGQVMYSL DQSQSVEVIE SFAINMETGW

**6501** ITTLKELDHE KJFTAEIYKG TVSELDPQGG VIAILSTTDA DSEEINRQVT

**6551** YFITGGDPLG QFAVETIQNE WKJFTAEIYK GTVSEPDPQG GVIAILSTTD

**6601** ADSEEINRQV TYFITGGDPL GQFAVETIQN EWKJFTAEIY KGTVSEQDPQ

**6651** GGVIAILSTT DADSEEINRQ VTYFITGGDP LGQFAVETIQ NEWKJGTVSE

**6701** DDPQGGVIAI LSTTDADSEE INRQVTYGIT GGDPLGQFAV ETIQNEWKVY

**6751** VKJGTVSEDD PQGGVIAILS TTDADSEEIN RQVTYSITGG DPLGQFAVET

**6801** IQNEWKVYVK JGTVSEDDPQ GGVIAILSTT DADSEEINRQ VTYFITGGDP

**6851** LAQFAVETIQ NEWKVYVKJA IVEVKVLDAN DNSPVCEYTL YSDTIPEDVL

**6901** PGKLIMQISA TDADIRJVLD ANDNSPVCEK TLYIDTIPED VLPGKLIMQI

**6951** SATDADIRJV LDANDNSPVC EKTLYTDTIP EDVLPGKLIM QISATDADIR

**7001** JATDGGGRFC QASIVLDLED VNDNAPEFSA DPYAITVFEN TEPGTLLTRV

**7051** QATDADAGLN RJKILYSLID SADGQFSINE LIGIIQLEKP LDRELQAVYT

**7101** LSLKJFSIDS KTGAVFIIEN LDYESSHEYY LTVEATDGGT PSLSDVATVN

**7151** VNVTDINDAT PVFSQDTYTT VISEDAVLEQ SVITVMADDA DGPSNSHIHY

**7201** SIIDGNQGSS FTIDPVRGEV KJFSIDSKTG AVFIIENLDY ESSHEYYLTV

**7251** EATDGGTPSL SDVATVNVNV TDINDVTPVF SQDTYTTVIS EDAVLEQSVI

**7301** TVMADDADGP SNSHIHYSII DGNQGSSFTI DPVRGEVKJV NTTTVNIDVS

**7351** DVNDNAPVFS RGNYSVIIQE NKPVGFSVLQ LVVTDEDSSH NGPPFFFTIV

**7401** TENDEKAFEV NPQGVLLTSS AIKJVNTTTV NIDVSDVNDN APVFSRGNYS

**7451** VIIQENKPVG FSVLQLVVTD EDSSHNGPPF FFTIVTGNDE KAFEVNPQGV

**7501** LLTSSAIKJG NYSVIIQENK PVGFSVLQLV VTDEDSSHNG PPFFFTIVTG

**7551** NDEKAFEVNA QGVLLTSSAI KRJGNYSVII QENKPVGFSV LQLVVTDEDS

**7601** SHNGPPFFFT IVTGNDEKAF EVNVQGVLLT SSAIKRJVAD NGKPQLSSLT

**7651** YIDIRVIEES IYPPAILPLE IAITSSGEEY SGGVIGKIHA TDQDVYDTLT

**7701** YSLDPQMDNL FSVSSTGGKJ VADNGKPQLS SLTYIDIRVI EESIYPPAIL

**7751** PLEISITSSG EEYSGGVIGK IHATDQDVYD TLTYSLDPQM DNLFSVSSTG

**7801** GKJVIHVTSP QFKAGPVKFE KJQLLHKIMS SVTDIEEIIG VRILNVFQKJ

**7851** QLLHKIVSSV TDIEEIIGVR ILNVFQKJEG RCPPVHHGCE DHPCPEGSEC

**7901** VSDPWEEKHT CVCPSGRJEG RCPPVHHGCE DPPCPEGSEC VSDPWEEKHT

**7951** CVCPSGRJHT CVCPSGRFGG CPGSSSMTLT GNSYVKYRJH TCVCPSGRFG

**8001** RCPGSSSMTL TGNSYVKYRJ FGQCPGSSSM TLTGNSYVKT RLTENENKJG

**8051** CMDSIYLNGQ ELPLNSKPRS YAHIEESVPV SPGCFLTATE DCASNPCQNG

**8101** GVCNPSPAGG YYCKCSALYI GTHCEISVNP CSSKPCLYGG TCVVDNGGFV

**8151** CQCRJYVFKR TPGKAKJYVF KSTPGKAKJS YAHIEESVDV SPGCFLTATE

**8201** DCASNPCQNG GVCNPSPAGG YYCKCSALYI GTHCEISVNP KSSKPCLYGG

**8251** TCVVDNGGFV CQCRGLYTGQ RJSYAHIEES VDVSPGCFLT ATEDCASNPC

**8301** QNGGVCNPSP AGGYYCKCSA LYIGTHCEIS VNPNSSKPCL YGGTCVVDNG

**8351** GFVCQCRGLY TGQRJDEPCK NGGTCFDSAD GAVCQCDSGF RGERJGRHCA

**8401** DAAPNQYVST PWNIGLAEGI GIVVFVAGIF LLVVVFVLCR KJGRHCEDAA

**8451** PNQYVSTPWN IGLAEGIGIV VFVAGIFLLV VVFVLCRKJN IYSDIPPQVP

**8501** VRYISYTPSI PSDSRNNLDR JKAVAVCSVA PNLPPPPPSN SPSDSDSIQK

**8551** PSWDFDYDTK VVDLDPCLSK JKPLEEKPSQ PYSARESLSS VQSLSSFQSE

**8601** SCDDNGYHWD TSDWMPSVPL PDIQEFPNYE VIDEQTPLYS ADPNAIDTDY

**8651** YPGGYDIESD FPPPPEDFPA ADELPPLPPE FSNQFESIHP PRDMPAAGSL

**8701** GSSSRJKPLE EKPSQPYSAR ESLSEVQSLS SFQSESCDDN GYHWDTSDWM

**8751** PSVPLQDIQE FPNYEVIDEQ TPLYSADPNA IDTDYYPGGY DIESDFPPPP

**8801** EDFPAADELP PLPPEFSNQF ESIHPPRDMP AAGSLGSSSR JKPLEEKPSQ

**8851** PYSARESLSE VQSLSSFQSE SCDDNGYHWD TSDWMPSVPL PDIQEFPNYE

**8901** VIDPQTPLYS ADPNAIDTDY YPGGYDIESD FPPPPEDFPA ADELPPLPPE

**8951** FSNQFESIHP PRDMPAAGSL GSSSRJESLS EVQSLSSFQS ESCDDNGYHW

**9001** DTSDWMPSVP LPDIQEFPNY EVIDEQTPLY SADPNAIDTD YYPGGYDIES

**9051** DFPPPPEDFP AADELPPLPP EFSNQFESIH PPRDMPAAGS LGSSRRNRJE

**9101** SLSEVQSLSS FQSESCDDNG YHWDTSDWMP SVPLPDIQEF PNYEVIDEQT

**9151** PLYSADPNAI DTDYYPGGYD IESDFPPPPE DFPAADELPP LPPEFSNQFE

**9201** SIHPPRDMPA AGSLGSSWRN RJVLGANSNP PEFTQTAYKA AFDENVPIGT

**9251** TIMSLSAVDP DEGENGYVTY SIANLNHVPF AIDHFTGAVS TSENLDYELM

**9301** PRVYTLRJVL GANSNPPEFT QTAYKAAFDE NVPIGTTVMS LSAVDPDEGE

**9351** NGYVTYSIAN LNHVPFAIDH FTGAVSTSEN LDYELMPRVY TLRJVLGANS

**9401** NPPEFTQTAY KAAFDENVPI GTTVMSLSAV DPDEGENGYV TYSIANLNHV

**9451** PFAIDHFTGA VSTSENLDYE LMPRVYTLRJ INCEGTIPRD LGVGEQITTV

**9501** SAIDADELQL VQYQIEAGNE LDFFSLNPNS GVLSLKRJIN CEGTIPRDLG

**9551** VGEQITTVSA IDADELQLVQ YQIEAGNELD LFSLNPNSGV LSLKRJINCE

**9601** GTIPRDLGVG EQITTVSAID ADELQLVQYQ IEAGNELDFF SLNPNSGVLS

**9651** LKRJEVHSEI IQVEATDKDL GPNGHVTYSI LTDTDTFSID SVTGVVNIAR

**9701** PLDRELQHEH SLKJEVHSEI IQVEATDKDL GPNGHVTYSI VTDTDTFSID

**9751** SVTGVVNIAR PLDRELQHEH SLK

**Start - End Observed Mr(expt) Mr(calc) ppm Miss Sequence**

**74 - 84 604.8118 1207.6090 1207.6085 0 0 K.IVSGDSENLFK.A**  ([Ions score 26](http://mbp-mascot4/mascot/cgi/peptide_view.pl?file=../data/20091021/F006277.dat&query=962&hit=1&index=IPI00031411&px=1&section=5&ave_thresh=22))

**85 - 97 816.8891 1631.7636 1631.7654 -1 0 K.AEEYILGDFCFLR.I**  ([Ions score 40](http://mbp-mascot4/mascot/cgi/peptide_view.pl?file=../data/20091021/F006277.dat&query=1619&hit=1&index=IPI00031411&px=1&section=5&ave_thresh=22))

**217 - 227 670.3318 1338.6490 1338.6489 0 0 K.LYEMEILAADR.G**  Oxidation (M) ([Ions score 69](http://mbp-mascot4/mascot/cgi/peptide_view.pl?file=../data/20091021/F006277.dat&query=1280&hit=1&index=IPI00031411&px=1&section=5&ave_thresh=22))

**217 - 227 670.3320 1338.6494 1338.6489 0 0 K.LYEMEILAADR.G**  Oxidation (M) ([Ions score 31](http://mbp-mascot4/mascot/cgi/peptide_view.pl?file=../data/20091021/F006277.dat&query=1281&hit=1&index=IPI00031411&px=1&section=5&ave_thresh=22))

**374 - 390 922.9763 1843.9381 1843.9390 -0 0 R.AEISEFAPPNTPVVMVK.A**  Oxidation (M) ([Ions score 25](http://mbp-mascot4/mascot/cgi/peptide_view.pl?file=../data/20091021/F006277.dat&query=1892&hit=1&index=IPI00031411&px=1&section=5&ave_thresh=22))

**453 - 469 918.9600 1835.9055 1835.9054 0 0 K.VLGANSNPPEFTQTAYK.A**  ([Ions score 25](http://mbp-mascot4/mascot/cgi/peptide_view.pl?file=../data/20091021/F006277.dat&query=1876&hit=1&index=IPI00031411&px=1&section=5&ave_thresh=22))

**669 - 681 730.8744 1459.7342 1459.7341 0 0 K.LVNLQCEETGVAK.M**  ([Ions score 53](http://mbp-mascot4/mascot/cgi/peptide_view.pl?file=../data/20091021/F006277.dat&query=1449&hit=1&index=IPI00031411&px=1&section=5&ave_thresh=22))

**903 - 914 688.3772 1374.7398 1374.7395 0 0 R.EEPQLFSTVVVK.V**  ([Ions score 21](http://mbp-mascot4/mascot/cgi/peptide_view.pl?file=../data/20091021/F006277.dat&query=1338&hit=1&index=IPI00031411&px=1&section=5&ave_thresh=22))

**985 - 993 560.3061 1118.5976 1118.5972 0 0 R.IVQQLDFEK.K**  ([Ions score 34](http://mbp-mascot4/mascot/cgi/peptide_view.pl?file=../data/20091021/F006277.dat&query=754&hit=1&index=IPI00031411&px=1&section=5&ave_thresh=22))

**1084 - 1096 703.3442 1404.6739 1404.6732 0 0 K.IGEETGVIETSDR.L**  ([Ions score 55](http://mbp-mascot4/mascot/cgi/peptide_view.pl?file=../data/20091021/F006277.dat&query=1393&hit=1&index=IPI00031411&px=1&section=5&ave_thresh=22))

**1084 - 1099 597.3026 1788.8860 1788.8854 0 1 K.IGEETGVIETSDRLDR.E**  ([Ions score 23](http://mbp-mascot4/mascot/cgi/peptide_view.pl?file=../data/20091021/F006277.dat&query=1809&hit=1&index=IPI00031411&px=1&section=5&ave_thresh=22))

**1300 - 1305 390.7182 779.4219 779.4218 0 0 K.FFIEPK.T**  ([Ions score 23](http://mbp-mascot4/mascot/cgi/peptide_view.pl?file=../data/20091021/F006277.dat&query=66&hit=1&index=IPI00031411&px=1&section=5&ave_thresh=22))

**1314 - 1326 707.3668 1412.7190 1412.7187 0 0 R.FSAAGEYDILSIK.A**  ([Ions score 60](http://mbp-mascot4/mascot/cgi/peptide_view.pl?file=../data/20091021/F006277.dat&query=1398&hit=1&index=IPI00031411&px=1&section=5&ave_thresh=22))

**1407 - 1422 547.6451 1639.9136 1639.9145 -1 0 K.GTGTIIVAKPLDAEQK.S**  ([Ions score 20](http://mbp-mascot4/mascot/cgi/peptide_view.pl?file=../data/20091021/F006277.dat&query=1627&hit=1&index=IPI00031411&px=1&section=5&ave_thresh=22))

**1489 - 1497 540.8063 1079.5981 1079.5975 1 0 K.LIYTLQSSR.D**  ([Ions score 49](http://mbp-mascot4/mascot/cgi/peptide_view.pl?file=../data/20091021/F006277.dat&query=649&hit=1&index=IPI00031411&px=1&section=5&ave_thresh=22))

**1489 - 1497 540.8066 1079.5987 1079.5975 1 0 K.LIYTLQSSR.D**  ([Ions score 33](http://mbp-mascot4/mascot/cgi/peptide_view.pl?file=../data/20091021/F006277.dat&query=650&hit=1&index=IPI00031411&px=1&section=5&ave_thresh=22))

**1507 - 1519 691.3455 1380.6765 1380.6773 -1 0 R.LDPATGSLYTSEK.L**  ([Ions score 24](http://mbp-mascot4/mascot/cgi/peptide_view.pl?file=../data/20091021/F006277.dat&query=1349&hit=1&index=IPI00031411&px=1&section=5&ave_thresh=22))

**1646 - 1657 638.8138 1275.6131 1275.6129 0 0 K.GSPPMSEITSVR.I**  Oxidation (M) ([Ions score 23](http://mbp-mascot4/mascot/cgi/peptide_view.pl?file=../data/20091021/F006277.dat&query=1176&hit=1&index=IPI00031411&px=1&section=5&ave_thresh=22))

**1646 - 1657 638.8140 1275.6135 1275.6129 0 0 K.GSPPMSEITSVR.I**  Oxidation (M) ([Ions score 31](http://mbp-mascot4/mascot/cgi/peptide_view.pl?file=../data/20091021/F006277.dat&query=1177&hit=1&index=IPI00031411&px=1&section=5&ave_thresh=22))

**1658 - 1669 638.3510 1274.6875 1274.6870 0 0 R.IFVTIADNASPK.F**  ([Ions score 58](http://mbp-mascot4/mascot/cgi/peptide_view.pl?file=../data/20091021/F006277.dat&query=1174&hit=1&index=IPI00031411&px=1&section=5&ave_thresh=22))

**1706 - 1726 734.3538 2200.0396 2200.0397 -0 0 K.DGNTGDAFDINPHSGTIITQK.A**  ([Ions score 38](http://mbp-mascot4/mascot/cgi/peptide_view.pl?file=../data/20091021/F006277.dat&query=2177&hit=1&index=IPI00031411&px=1&section=5&ave_thresh=22))

**1982 - 1992 628.8298 1255.6450 1255.6449 0 0 K.FTQDVYSAVVK.E**  ([Ions score 30](http://mbp-mascot4/mascot/cgi/peptide_view.pl?file=../data/20091021/F006277.dat&query=1117&hit=1&index=IPI00031411&px=1&section=5&ave_thresh=22))

**2031 - 2044 719.8621 1437.7097 1437.7100 -0 0 R.TSGVLSTTGTPFDR.E**  ([Ions score 28](http://mbp-mascot4/mascot/cgi/peptide_view.pl?file=../data/20091021/F006277.dat&query=1429&hit=1&index=IPI00031411&px=1&section=5&ave_thresh=22))

**2072 - 2094 869.4553 2605.3441 2605.3428 1 0 K.VIVEDQNDNAPVFVNLPYYAVVK.V**  ([Ions score 26](http://mbp-mascot4/mascot/cgi/peptide_view.pl?file=../data/20091021/F006277.dat&query=2504&hit=1&index=IPI00031411&px=1&section=5&ave_thresh=22))

**2125 - 2141 657.6746 1970.0018 1970.0010 0 0 K.EHHEHFQIGPLGEISLK.K**  ([Ions score 21](http://mbp-mascot4/mascot/cgi/peptide_view.pl?file=../data/20091021/F006277.dat&query=2011&hit=1&index=IPI00031411&px=1&section=5&ave_thresh=22))

**2332 - 2349 674.3464 2020.0173 2020.0126 2 0 K.SHDHFHVDSSTGLISLLR.T**  ([Ions score 33](http://mbp-mascot4/mascot/cgi/peptide_view.pl?file=../data/20091021/F006277.dat&query=2067&hit=1&index=IPI00031411&px=1&section=5&ave_thresh=22))

**2440 - 2457 665.3655 1993.0746 1993.0745 0 0 K.HFVIDSATGIITLSNLHR.H**  ([Ions score 73](http://mbp-mascot4/mascot/cgi/peptide_view.pl?file=../data/20091021/F006277.dat&query=2038&hit=1&index=IPI00031411&px=1&section=5&ave_thresh=22))

**2604 - 2614 569.7914 1137.5682 1137.5666 1 0 K.YEVNIGSSAAK.G**  ([Ions score 26](http://mbp-mascot4/mascot/cgi/peptide_view.pl?file=../data/20091021/F006277.dat&query=795&hit=1&index=IPI00031411&px=1&section=5&ave_thresh=22))

**2743 - 2758 612.6221 1834.8444 1834.8446 -0 1 K.GNTPESNRDESFVIDR.Q**  ([Ions score 20](http://mbp-mascot4/mascot/cgi/peptide_view.pl?file=../data/20091021/F006277.dat&query=1871&hit=1&index=IPI00031411&px=1&section=5&ave_thresh=22))

**2776 - 2784 592.3172 1182.6198 1182.6186 1 0 K.WYQFSILAR.C**  ([Ions score 59](http://mbp-mascot4/mascot/cgi/peptide_view.pl?file=../data/20091021/F006277.dat&query=889&hit=1&index=IPI00031411&px=1&section=5&ave_thresh=22))

**3029 - 3043 824.4266 1646.8386 1646.8403 -1 0 K.TLYSDTIPEDVLPGK.L**  ([Ions score 52](http://mbp-mascot4/mascot/cgi/peptide_view.pl?file=../data/20091021/F006277.dat&query=1633&hit=1&index=IPI00031411&px=1&section=5&ave_thresh=22))

**3044 - 3056 731.8820 1461.7494 1461.7497 -0 0 K.LIMQISATDADIR.S**  Oxidation (M) ([Ions score 80](http://mbp-mascot4/mascot/cgi/peptide_view.pl?file=../data/20091021/F006277.dat&query=1452&hit=1&index=IPI00031411&px=1&section=5&ave_thresh=22))

**3057 - 3072 827.4199 1652.8252 1652.8257 -0 0 R.SNAEITYTLLGSGAEK.F**  ([Ions score 52](http://mbp-mascot4/mascot/cgi/peptide_view.pl?file=../data/20091021/F006277.dat&query=1642&hit=1&index=IPI00031411&px=1&section=5&ave_thresh=22))

**3084 - 3101 709.7115 2126.1126 2126.1120 0 1 K.TSTPLDREEQAVYHLLVR.A**  ([Ions score 20](http://mbp-mascot4/mascot/cgi/peptide_view.pl?file=../data/20091021/F006277.dat&query=2137&hit=1&index=IPI00031411&px=1&section=5&ave_thresh=22))

**3491 - 3507 591.9955 1772.9646 1772.9672 -1 0 K.AFEVNPQGVLLTSSAIK.R**  ([Ions score 24](http://mbp-mascot4/mascot/cgi/peptide_view.pl?file=../data/20091021/F006277.dat&query=1784&hit=1&index=IPI00031411&px=1&section=5&ave_thresh=22))

**3491 - 3507 887.4901 1772.9657 1772.9672 -1 0 K.AFEVNPQGVLLTSSAIK.R**  ([Ions score 85](http://mbp-mascot4/mascot/cgi/peptide_view.pl?file=../data/20091021/F006277.dat&query=1785&hit=1&index=IPI00031411&px=1&section=5&ave_thresh=22))

**3520 - 3537 664.0258 1989.0554 1989.0531 1 0 K.VADNGKPQLSSLTYIDIR.V**  ([Ions score 26](http://mbp-mascot4/mascot/cgi/peptide_view.pl?file=../data/20091021/F006277.dat&query=2035&hit=1&index=IPI00031411&px=1&section=5&ave_thresh=22))

**3650 - 3664 922.4359 1842.8573 1842.8577 -0 0 R.FANLTPEEFVGDYWR.N**  ([Ions score 23](http://mbp-mascot4/mascot/cgi/peptide_view.pl?file=../data/20091021/F006277.dat&query=1891&hit=1&index=IPI00031411&px=1&section=5&ave_thresh=22))

**3876 - 3888 499.9202 1496.7387 1496.7372 1 0 R.GTDYSILEIHHGR.L**  ([Ions score 25](http://mbp-mascot4/mascot/cgi/peptide_view.pl?file=../data/20091021/F006277.dat&query=1475&hit=1&index=IPI00031411&px=1&section=5&ave_thresh=22))

**3928 - 3945 603.3359 1806.9858 1806.9840 1 0 R.LVLDQVHTASGTAPGTLK.T**  ([Ions score 28](http://mbp-mascot4/mascot/cgi/peptide_view.pl?file=../data/20091021/F006277.dat&query=1835&hit=1&index=IPI00031411&px=1&section=5&ave_thresh=22))

**MiaPaCa A3:**

Match to: **IPI00031411** Score: **111**

**Gene_Symbol=FAT1 Protocadherin Fat 1 lng=4591 # SP[4593,D,22,D]SNP[4646,g,1064,R]SNP[4677,r,1064,R]SNP[4713,i,1125,I]SNP[4791,l,1125,I]SNP[4844,i,1252,I]SNP[4856,h,1273,H]SNP[4889,r,1273,H]SNP[4931,l,1283,P]SNP[4969,p,1283,P]SNP[5000,l,129,V]SNP[5**

Found in search of C:\mgf\Orbidata\081126_ISW1295_MiaPaca2_A3.mgf

Nominal mass (M_r_): **1068827**; Calculated pI value: **4.55**

NCBI BLAST search of [IPI00031411](http://www.ncbi.nlm.nih.gov/blast/Blast.cgi?ALIGNMENTS=50&ALIGNMENT_VIEW=Pairwise&AUTO_FORMAT=Semiauto&CDD_SEARCH=on&CLIENT=web&COMPOSITION_BASED_STATISTICS=on&DATABASE=nr&DESCRIPTIONS=100&ENTREZ_QUERY=(none)&EXPECT=10&FILTER=L&FORMAT_BLOCK_ON_RESPAGE=None&FORMAT_OBJECT=Alignment&FORMAT_TYPE=HTML&GAPCOSTS=11+1&I_THRESH=0.001&LAYOUT=TwoWindows&MATRIX_NAME=BLOSUM62&NCBI_GI=on&PAGE=Proteins&PROGRAM=blastp&QUERY=IPI00031411&SERVICE=plain&SET_DEFAULTS.x=21&SET_DEFAULTS.y=7&SHOW_OVERVIEW=on&WORD_SIZE=3&END_OF_HTTPGET=Yes) against nr

Unformatted [sequence string](http://mbp-mascot4/mascot/cgi/getseq.pl?IPI_human+IPI00031411+seq) for pasting into other applications

Fixed modifications: Carbamidomethyl (C)

Variable modifications: Oxidation (M)

Cleavage by TrypsinMSIPI, a mixture of enzymes:

cuts C-term side of KR unless next residue is P

cuts C-term side of J

cuts N-term side of J

Sequence Coverage: **1%**

Matched peptides shown in **Bold Red**

**1** MGRHLALLLL LLLLFQHFGD SDGSQRLEQT PLQFTHLEYN VTVQENSAAK

**51** TYVGHPVKMG VYITHPAWEV RYKIVSGDSE NLFKAEEYIL GDFCFLRIRT

**101** KGGNTAILNR EVKDHYTLIV KALEKNTNVE ARTKVRVQVL DTNDLRPLFS

**151** PTSYSVSLPE NTAIRTSIAR VSATDADIGT NGEFYYSFKD RTDMFAIHPT

**201** SGVIVLTGRL DYLETKLYEM EILAADRGMK LYGSSGISSM AKLTVHIEQA

**251** NECAPVITAV TLSPSELDRD PAYAIVTVDD CDQGANGDIA SLSIVAGDLL

**301** QQFRTVRSFP GSKEYKVKAI GGIDWDSHPF GYNLTLQAKD KGTPPQFSSV

**351** KVIHVTSPQF KAGPVKFEKD VYRAEISEFA PPNTPVVMVK AIPAYSHLRY

**401** VFKSTPGKAK FSLNYNTGLI SILEPVKRQQ AAHFELEVTT SDRKASTKVL

**451** VK**VLGANSNP PEFTQTAYK**A AFDENVPIGT TVMSLSAVDP DEGENGYVTY

**501** SIANLNHVPF AIDHFTGAVS TSENLDYELM PRVYTLRIRA SDWGLPYRRE

**551** VEVLATITLN NLNDNTPLFE KINCEGTIPR DLGVGEQITT VSAIDADELQ

**601** LVQYQIEAGN ELDFFSLNPN SGVLSLKRSL MDGLGAKVSF HSLRITATDG

**651** ENFATPLYIN ITVAASHK**LV NLQCEETGVA K**MLAEKLLQA NKLHNQGEVE

**701** DIFFDSHSVN AHIPQFRSTL PTGIQVKENQ PVGSSVIFMN STDLDTGFNG

**751** KLVYAVSGGN EDSCFMIDME TGMLKILSPL DRETTDKYTL NITVYDLGIP

**801** QKAAWRLLHV VVVDANDNPP EFLQESYFVE VSEDKEVHSE IIQVEATDKD

**851** LGPNGHVTYS IVTDTDTFSI DSVTGVVNIA RPLDRELQHE HSLKIEARDQ

**901** AREEPQLFST VVVKVSLEDV NDNPPTFIPP NYRVKVREDL PEGTVIMWLE

**951** AHDPDLGQSG QVRYSLLDHG EGNFDVDKLS GAVRIVQQLD FEKKQVYNLT

**1001** VRAKDKGKPV SLSSTCYVEV EVVDVNENLH PPVFSSFVEK GTVKEDAPVG

**1051** SLVMTVSAHD EDARRDGEIR YSIRDGSGVG VFK**IGEETGV IETSDRLDR**E

**1101** STSHYWLTVF ATDQGVVPLS SFIEIYIEVE DVNDNAPQTS EPVYYPEIME

**1151** NSPKDVSVVQ IEAFDPDSSS NDKLMYKITS GNPQGFFSIH PKTGLITTTS

**1201** RKLDREQQDE HILEVTVTDN GSPPKSTIAR VIVK**ILDEND NKPQFLQK**FY

**1251** KIRLPEREKP DRERNARREP LYHVIATDKD EGPNAEISYS IEDGNEHGKF

**1301** FIEPKTGVVS SKRFSAAGEY DILSIKAVDN GRPQKSSTTR LHIEWISKPK

**1351** PSLEPISFEE SFFTFTVMES DPVAHMIGVI SVEPPGIPLW FDITGGNYDS

**1401** HFDVDKGTGT IIVAKPLDAE QKSNYNLTVE ATDGTTTILT QVFIKVIDTN

**1451** DHRPQFSTSK YEVVIPEDTA PETEILQISA VDQDEKNK**LI YTLQSSR**DPL

**1501** SLKKFRLDPA TGSLYTSEKL DHEAVHQHTL TVMVRDQDVP VKRNFARIVV

**1551** NVSDTNDHAP WFTASSYKGR VYESAAVGSV VLQVTALDKD KGKNAEVLYS

**1601** IESGTFGNIG NSFMIDPVLG SIKTAKELDR SNQAEYDLMV KATDKGSPPM

**1651** SEITSVRIFV TIADNASPKF TSKEYSVELS ETVSIGSFVG MVTAHSQSSV

**1701** VYEIK**DGNTG DAFDINPHSG TIITQK**ALDF ETLPIYTLII QGTNMAGLST

**1751** NTTVLVHLQD ENDNAPVFMQ AEYTGLISES ASINSVVLTD RNVPLVIRAA

**1801** DADKDSNALL VYHIVEPSVH TYFAIDSSTG AIHTVLSLDY EETSIFHFTV

**1851** QVHDMGTPRL FAEYAANVTV HVIDINDCPP VFAKPLYEAS LLLPTYKGVK

**1901** VITVNATDAD SSAFSQLIYS ITEGNIGEKF SMDYKTGALT VQNTTQLRSR

**1951** YELTVRASDG RFAGLTSVKI NVKESKESHL K**FTQDVYSAV VK**ENSTEAET

**2001** LAVITAIGNP INEPLFYHIL NPDRRFKISR TSGVLSTTGT PFDREQQEAF

**2051** DVVVEVTEEH KPSAVAHVVV KVIVEDQNDN APVFVNLPYY AVVKVDTEVG

**2101** HVIRYVTAVD RDSGRNGEVH YYLKEHHEHF QIGPLGEISL KK**QFELDTLN**

**2151 K**EYLVTVVAK DGGNPAFSAE VIVPITVMNK AMPVFEKPFY SAEIAESIQV

**2201** HSPVVHVQAN SPEGLKVFYS ITDGDPFSQF TINFNTGVIN VIAPLDFEAH

**2251** PAYKLSIRAT DSLTGAHAEV FVDIIVDDIN DNPPVFAQQS YAVTLSEASV

**2301** IGTSVVQVRA TDSDSEPNRG ISYQMFGNHS KSHDHFHVDS STGLISLLRT

**2351** LDYEQSRQHT IFVRAVDGGM PTLSSDVIVT VDVTDLNDNP PLFEQQIYEA

**2401** RISEHAPHGH FVTCVKAYDA DSSDIDKLQY SILSGNDHK**H FVIDSATGII**

**2451 TLSNLHR**HAL KPFYSLNLSV SDGVFRSSTQ VHVTVIGGNL HSPAFLQNEY

**2501** EVELAENAPL HTLVMEVKTT DGDSGIYGHV TYHIVNDFAK DRFYINERGQ

**2551** IFTLEKLDRE TPAEKVISVR LMAKDAGGKV AFCTVNVILT DDNDNAPQFR

**2601** ATKYEVNIGS SAAKGTSVVK VLASDADEGS NADITYAIEA DSESVKENLE

**2651** INKLSGVITT KESLIGLENE FFTFFVRAVD NGSPSKESVV LVYVKILPPE

**2701** MQLPKFSEPF YTFTVSEDVP IGTEIDLIRA EHSGTVLYSL VKGNTPESNR

**2751** DESFVIDRQS GRLKLEKSLD HETTKWYQFS ILARCTQDDH EMVASVDVSI

**2801** QVKDANDNSP VFESSPYEAF IVENLPGGSR VIQIRASDAD SGTNGQVMYS

**2851** LDQSQSVEVI ESFAINMETG WITTLKELDH EKRDNYQIKV VASDHGEKIQ

**2901** LSSTAIVDVT VTDVNDSPPR FTAEIYKGTV SEDDPQGGVI AILSTTDADS

**2951** EEINRQVTYF ITGGDPLGQF AVETIQNEWK VYVKKPLDRE KRDNYLLTIT

**3001** ATDGTFSSKA IVEVKVLDAN DNSPVCEKTL YSDTIPEDVL PGKLIMQISA

**3051** TDADIRSNAE ITYTLLGSGA EKFKLNPDTG ELKTSTPLDR EEQAVYHLLV

**3101** RATDGGGRFC QASIVLTLED VNDNAPEFSA DPYAITVFEN TEPGTLLTRV

**3151** QATDADAGLN RKILYSLIDS ADGQFSINEL SGIIQLEKPL DRELQAVYTL

**3201** SLKAVDQGLP RRLTATGTVI VSVLDINDNP PVFEYREYGA TVSEDILVGT

**3251** EVLQVYAASR DIEANAEITY SIISGNEHGK FSIDSKTGAV FIIENLDYES

**3301** SHEYYLTVEA TDGGTPSLSD VATVNVNVTD INDNTPVFSQ DTYTTVISED

**3351** AVLEQSVITV MADDADGPSN SHIHYSIIDG NQGSSFTIDP VRGEVKVTKL

**3401** LDRETISGYT LTVQASDNGS PPRVNTTTVN IDVSDVNDNA PVFSRGNYSV

**3451** IIQENKPVGF SVLQLVVTDE DSSHNGPPFF FTIVTGNDEK AFEVNPQGVL

**3501** LTSSAIKRKE KDHYLLQVKV ADNGKPQLSS LTYIDIRVIE ESIYPPAILP

**3551** LEIFITSSGE EYSGGVIGKI HATDQDVYDT LTYSLDPQMD NLFSVSSTGG

**3601** KLIAHKKLDI GQYLLNVSVT DGKFTTVADI TVHIRQVTQE MLNHTIAIRF

**3651** ANLTPEEFVG DYWRNFQRAL RNILGVRRND IQIVSLQSSE PHPHLDVLLF

**3701** VEKPGSAQIS TKQLLHKINS SVTDIEEIIG VRILNVFQKL CAGLDCPWKF

**3751** CDEKVSVDES VMSTHSTARL SFVTPRHHRA AVCLCKEGRC PPVHHGCEDD

**3801** PCPEGSECVS DPWEEKHTCV CPSGRFGQCP GSSSMTLTGN SYVKYRLTEN

**3851** ENKLEMKLTM RLRTYSTHAV VMYARGTDYS ILEIHHGRLQ YKFDCGSGPG

**3901** IVSVQSIQVN DGQWHAVALE VNGNYARLVL DQVHTASGTA PGTLKTLNLD

**3951** NYVFFGGHIR QQGTRHGRSP QVGNGFRGCM DSIYLNGQEL PLNSKPRSYA

**4001** HIEESVDVSP GCFLTATEDC ASNPCQNGGV CNPSPAGGYY CKCSALYIGT

**4051** HCEISVNPCS SKPCLYGGTC VVDNGGFVCQ CRGLYTGQRC QLSPYCKDEP

**4101** CKNGGTCFDS LDGAVCQCDS GFRGERCQSD IDECSGNPCL HGALCENTHG

**4151** SYHCNCSHEY RGRHCEDAAP NQYVSTPWNI GLAEGIGIVV FVAGIFLLVV

**4201** VFVLCRKMIS RKKKHQAEPK DKHLGPATAF LQRPYFDSKL NKNIYSDIPP

**4251** QVPVRPISYT PSIPSDSRNN LDRNSFEGSA IPEHPEFSTF NPESVHGHRK

**4301** AVAVCSVAPN LPPPPPSNSP SDSDSIQKPS WDFDYDTKVV DLDPCLSKKP

**4351** LEEKPSQPYS ARESLSEVQS LSSFQSESCD DNGYHWDTSD WMPSVPLPDI

**4401** QEFPNYEVID EQTPLYSADP NAIDTDYYPG GYDIESDFPP PPEDFPAADE

**4451** LPPLPPEFSN QFESIHPPRD MPAAGSLGSS SRNRQRFNLN QYLPNFYPLD

**4501** MSEPQTKGTG ENSTCREPHA PYPPGYQRHF EAPAVESMPM SVYASTASCS

**4551** DVSACCEVES EVMMSDYESG DDGHFEEVTI PPLDSQQHTE VJDGSQRLEQ

**4601** TPLQFTHLEY NVTVQENSAA KJGTVKEDAP VGSLVMTVSA HDEDAGRDGE

**4651** IRJGTVKEDA PVGSLVMTVS AHDEDARRDG EIRJLDREST SHYWLTVFAT

**4701** DQGVVPLSSF IEIYIEVEDV NDNAPQTSEP VYYPEIMENS PKDVSVVQIE

**4751** AFDPDSSSND KJLDRESTSH YWLTVFATDQ GVVPLSSFIE LYIEVEDVND

**4801** NAPQTSEPVY YPEIMENSPK DVSVVQIEAF DPDSSSNDKJ FYKIRLPERJ

**4851** REPLYHVIAT DKDEGPNAEI SYSIEDGNEH GKJREPLYRV IATDKDEGPN

**4901** AEISYSIEDG NEHGKJEPLY HVIATDKDEG LNAEISYSIE DGNEHGKFFI

**4951** EPKJEPLYHV IATDKDEGPN AEISYSIEDG NEHGKFFIEP KJALEKNTNL

**5001** EARTKJALEK NTNVEARTKJ ALEKNTNVEA RTKJALEKNT NVEVRTKJFS

**5051** AAGEYDILSI KAVDNGRPQK SSTTRJFSAA GEYDILSIKA VDSGRPQKSS

**5101** TTRJNFARIV VNVSDTNDHA PWFTASSYKG RJNFARIVVN VSDTNDHAPW

**5151** FTTSSYKGRJ GKNAEVLYSI ESGDFGNIGN SFMIDPVLGS IKTAKJGKNA

**5201** EVLYSIESGN FGNIGNSFMI DPVLGSIKTA KJGKNAEVLY SIESGTIGNI

**5251** GNSFMIDPVL GSIKTAKJGS PPMSEITSVR IFVTNADNAS PKFTSKJGSP

**5301** PMSEITSVRI FVTSADNASP KFTSKJGSPP MSEITSVRIF VTIADNASTK

**5351** FTSKJALDFE TLPIYTLIIQ GTNMAGLSTN TTVLVHLQDE NDNAPVFMQA

**5401** EYTGLISESA SINSVVLTDR NVPQVIRAAD ADKJALDFET LPIYTLIIQG

**5451** TNMAGLSTNT TVLVHLQDEN DNAPVFMQAE YTGLISESAS INSVVLTDRN

**5501** VPRVIRAADA DKJAADADKD SNVLLVYHIV EPSVHTYFAI DSSTGAIHTV

**5551** LSLDYEETSI FHFTVQVHDM GTPRLFAEYA ANVTVHVIDI NDCPPVFAKP

**5601** LYEASLLLPT YKJFTQDVYS AVVKTNSTEA ETLAVITAIG NPINEPLFYH

**5651** ILNPDRRJTS GVLSTTGTPF DREAQEAFDV VVEVTEEHKP SAVAHVVVKV

**5701** IVEDQNDNAP VFVNLPYYAV VKJTDMFAIH PTSGVIVLTG RLDFLETKLY

**5751** EMEILAADRJ TDMFAIHPTS GVIVLTGRLD YLETKLYEME ILAADRJNGE

**5801** VHYYLKEHHE HFQIGPLGEK SLKKJDGGNP AFSAEVIVPI TVMNKAMPVF

**5851** EKPFYSAEIA ESIQVHSHVV HVQANSPEGL KVFYSITDGD PFSQFTINFN

**5901** TGVINVIAPL DFEAHPAYKJ AMPVFEKPFY SAEIAESIQV HSPVVHVQAN

**5951** SPEGLKVFYS ITDGDPFSQF TINFNTGVIN VIAPLDFPAH PAYKLSIRJL

**6001** SIRATDSLTG AHAEVFVDDI VDDINDNPPV FAQQSYAVTL SEASVIGTSV

**6051** VQVRATDSDS EPNRJLSIRA TDSLTGAHAE VFVDEIVDDI NDNPPVFAQQ

**6101** SYAVTLSEAS VIGTSVVQVR ATDSDSEPNR JTLDYEQSRQ HTIAVRAVDG

**6151** GMPTLSSDVI VTVDVTDLND NPPLFEQQIY EARJLYGSSG ISSMAKLTVH

**6201** IEQANECAPV ITAVTLSPSE LDRDPAYAIV TVDDCDQGAN GDIASLSIVA

**6251** GDLLQQFRJI LPPEMQLPKF SEPFYTFTVS EIVPIGTEID LIRAEHSGTV

**6301** LYSLVKJILP PEMQLPKFSE PFYTFTVSEV VPIGTEIDLI RAEHSGTVLY

**6351** SLVKJCTQDD HEMVASVDVS IQVKDANDNS PVFESSPYEA FIVENLPGGS

**6401** RVIQIRJCTQ DDHEMVASVD VSIQVKDASD NSPVFESSPY EAFIVENLPG

**6451** GSRVIQIRJV IQIRASDADN GTNGQVMYSL DQSQSVEVIE SFAINMETGW

**6501** ITTLKELDHE KJFTAEIYKG TVSELDPQGG VIAILSTTDA DSEEINRQVT

**6551** YFITGGDPLG QFAVETIQNE WKJFTAEIYK GTVSEPDPQG GVIAILSTTD

**6601** ADSEEINRQV TYFITGGDPL GQFAVETIQN EWKJFTAEIY KGTVSEQDPQ

**6651** GGVIAILSTT DADSEEINRQ VTYFITGGDP LGQFAVETIQ NEWKJGTVSE

**6701** DDPQGGVIAI LSTTDADSEE INRQVTYGIT GGDPLGQFAV ETIQNEWKVY

**6751** VKJGTVSEDD PQGGVIAILS TTDADSEEIN RQVTYSITGG DPLGQFAVET

**6801** IQNEWKVYVK JGTVSEDDPQ GGVIAILSTT DADSEEINRQ VTYFITGGDP

**6851** LAQFAVETIQ NEWKVYVKJA IVEVKVLDAN DNSPVCEYTL YSDTIPEDVL

**6901** PGKLIMQISA TDADIRJVLD ANDNSPVCEK TLYIDTIPED VLPGKLIMQI

**6951** SATDADIRJV LDANDNSPVC EKTLYTDTIP EDVLPGKLIM QISATDADIR

**7001** JATDGGGRFC QASIVLDLED VNDNAPEFSA DPYAITVFEN TEPGTLLTRV

**7051** QATDADAGLN RJKILYSLID SADGQFSINE LIGIIQLEKP LDRELQAVYT

**7101** LSLKJFSIDS KTGAVFIIEN LDYESSHEYY LTVEATDGGT PSLSDVATVN

**7151** VNVTDINDAT PVFSQDTYTT VISEDAVLEQ SVITVMADDA DGPSNSHIHY

**7201** SIIDGNQGSS FTIDPVRGEV KJFSIDSKTG AVFIIENLDY ESSHEYYLTV

**7251** EATDGGTPSL SDVATVNVNV TDINDVTPVF SQDTYTTVIS EDAVLEQSVI

**7301** TVMADDADGP SNSHIHYSII DGNQGSSFTI DPVRGEVKJV NTTTVNIDVS

**7351** DVNDNAPVFS RGNYSVIIQE NKPVGFSVLQ LVVTDEDSSH NGPPFFFTIV

**7401** TENDEKAFEV NPQGVLLTSS AIKJVNTTTV NIDVSDVNDN APVFSRGNYS

**7451** VIIQENKPVG FSVLQLVVTD EDSSHNGPPF FFTIVTGNDE KAFEVNPQGV

**7501** LLTSSAIKJG NYSVIIQENK PVGFSVLQLV VTDEDSSHNG PPFFFTIVTG

**7551** NDEKAFEVNA QGVLLTSSAI KRJGNYSVII QENKPVGFSV LQLVVTDEDS

**7601** SHNGPPFFFT IVTGNDEKAF EVNVQGVLLT SSAIKRJVAD NGKPQLSSLT

**7651** YIDIRVIEES IYPPAILPLE IAITSSGEEY SGGVIGKIHA TDQDVYDTLT

**7701** YSLDPQMDNL FSVSSTGGKJ VADNGKPQLS SLTYIDIRVI EESIYPPAIL

**7751** PLEISITSSG EEYSGGVIGK IHATDQDVYD TLTYSLDPQM DNLFSVSSTG

**7801** GKJVIHVTSP QFKAGPVKFE KJQLLHKIMS SVTDIEEIIG VRILNVFQKJ

**7851** QLLHKIVSSV TDIEEIIGVR ILNVFQKJEG RCPPVHHGCE DHPCPEGSEC

**7901** VSDPWEEKHT CVCPSGRJEG RCPPVHHGCE DPPCPEGSEC VSDPWEEKHT

**7951** CVCPSGRJHT CVCPSGRFGG CPGSSSMTLT GNSYVKYRJH TCVCPSGRFG

**8001** RCPGSSSMTL TGNSYVKYRJ FGQCPGSSSM TLTGNSYVKT RLTENENKJG

**8051** CMDSIYLNGQ ELPLNSKPRS YAHIEESVPV SPGCFLTATE DCASNPCQNG

**8101** GVCNPSPAGG YYCKCSALYI GTHCEISVNP CSSKPCLYGG TCVVDNGGFV

**8151** CQCRJYVFKR TPGKAKJYVF KSTPGKAKJS YAHIEESVDV SPGCFLTATE

**8201** DCASNPCQNG GVCNPSPAGG YYCKCSALYI GTHCEISVNP KSSKPCLYGG

**8251** TCVVDNGGFV CQCRGLYTGQ RJSYAHIEES VDVSPGCFLT ATEDCASNPC

**8301** QNGGVCNPSP AGGYYCKCSA LYIGTHCEIS VNPNSSKPCL YGGTCVVDNG

**8351** GFVCQCRGLY TGQRJDEPCK NGGTCFDSAD GAVCQCDSGF RGERJGRHCA

**8401** DAAPNQYVST PWNIGLAEGI GIVVFVAGIF LLVVVFVLCR KJGRHCEDAA

**8451** PNQYVSTPWN IGLAEGIGIV VFVAGIFLLV VVFVLCRKJN IYSDIPPQVP

**8501** VRYISYTPSI PSDSRNNLDR JKAVAVCSVA PNLPPPPPSN SPSDSDSIQK

**8551** PSWDFDYDTK VVDLDPCLSK JKPLEEKPSQ PYSARESLSS VQSLSSFQSE

**8601** SCDDNGYHWD TSDWMPSVPL PDIQEFPNYE VIDEQTPLYS ADPNAIDTDY

**8651** YPGGYDIESD FPPPPEDFPA ADELPPLPPE FSNQFESIHP PRDMPAAGSL

**8701** GSSSRJKPLE EKPSQPYSAR ESLSEVQSLS SFQSESCDDN GYHWDTSDWM

**8751** PSVPLQDIQE FPNYEVIDEQ TPLYSADPNA IDTDYYPGGY DIESDFPPPP

**8801** EDFPAADELP PLPPEFSNQF ESIHPPRDMP AAGSLGSSSR JKPLEEKPSQ

**8851** PYSARESLSE VQSLSSFQSE SCDDNGYHWD TSDWMPSVPL PDIQEFPNYE

**8901** VIDPQTPLYS ADPNAIDTDY YPGGYDIESD FPPPPEDFPA ADELPPLPPE

**8951** FSNQFESIHP PRDMPAAGSL GSSSRJESLS EVQSLSSFQS ESCDDNGYHW

**9001** DTSDWMPSVP LPDIQEFPNY EVIDEQTPLY SADPNAIDTD YYPGGYDIES

**9051** DFPPPPEDFP AADELPPLPP EFSNQFESIH PPRDMPAAGS LGSSRRNRJE

**9101** SLSEVQSLSS FQSESCDDNG YHWDTSDWMP SVPLPDIQEF PNYEVIDEQT

**9151** PLYSADPNAI DTDYYPGGYD IESDFPPPPE DFPAADELPP LPPEFSNQFE

**9201** SIHPPRDMPA AGSLGSSWRN RJVLGANSNP PEFTQTAYKA AFDENVPIGT

**9251** TIMSLSAVDP DEGENGYVTY SIANLNHVPF AIDHFTGAVS TSENLDYELM

**9301** PRVYTLRJVL GANSNPPEFT QTAYKAAFDE NVPIGTTVMS LSAVDPDEGE

**9351** NGYVTYSIAN LNHVPFAIDH FTGAVSTSEN LDYELMPRVY TLRJVLGANS

**9401** NPPEFTQTAY KAAFDENVPI GTTVMSLSAV DPDEGENGYV TYSIANLNHV

**9451** PFAIDHFTGA VSTSENLDYE LMPRVYTLRJ INCEGTIPRD LGVGEQITTV

**9501** SAIDADELQL VQYQIEAGNE LDFFSLNPNS GVLSLKRJIN CEGTIPRDLG

**9551** VGEQITTVSA IDADELQLVQ YQIEAGNELD LFSLNPNSGV LSLKRJINCE

**9601** GTIPRDLGVG EQITTVSAID ADELQLVQYQ IEAGNELDFF SLNPNSGVLS

**9651** LKRJEVHSEI IQVEATDKDL GPNGHVTYSI LTDTDTFSID SVTGVVNIAR

**9701** PLDRELQHEH SLKJEVHSEI IQVEATDKDL GPNGHVTYSI VTDTDTFSID

**9751** SVTGVVNIAR PLDRELQHEH SLK

**Start - End Observed Mr(expt) Mr(calc) ppm Miss Sequence**

**453 - 469 918.9596 1835.9046 1835.9054 -0 0 K.VLGANSNPPEFTQTAYK.A**  ([Ions score 25](http://mbp-mascot4/mascot/cgi/peptide_view.pl?file=../data/20091021/F006278.dat&query=1978&hit=1&index=IPI00031411&px=1&section=5&ave_thresh=22))

**669 - 681 730.8746 1459.7347 1459.7341 0 0 K.LVNLQCEETGVAK.M**  ([Ions score 47](http://mbp-mascot4/mascot/cgi/peptide_view.pl?file=../data/20091021/F006278.dat&query=1537&hit=1&index=IPI00031411&px=1&section=5&ave_thresh=22))

**1084 - 1099 597.3032 1788.8876 1788.8854 1 1 K.IGEETGVIETSDRLDR.E**  ([Ions score 32](http://mbp-mascot4/mascot/cgi/peptide_view.pl?file=../data/20091021/F006278.dat&query=1921&hit=1&index=IPI00031411&px=1&section=5&ave_thresh=22))

**1235 - 1248 567.9653 1700.8742 1700.8733 0 0 K.ILDENDNKPQFLQK.F**  ([Ions score 30](http://mbp-mascot4/mascot/cgi/peptide_view.pl?file=../data/20091021/F006278.dat&query=1823&hit=1&index=IPI00031411&px=1&section=5&ave_thresh=22))

**1489 - 1497 540.8068 1079.5990 1079.5975 1 0 K.LIYTLQSSR.D**  ([Ions score 20](http://mbp-mascot4/mascot/cgi/peptide_view.pl?file=../data/20091021/F006278.dat&query=681&hit=1&index=IPI00031411&px=1&section=5&ave_thresh=22))

**1706 - 1726 734.3549 2200.0429 2200.0397 1 0 K.DGNTGDAFDINPHSGTIITQK.A**  ([Ions score 34](http://mbp-mascot4/mascot/cgi/peptide_view.pl?file=../data/20091021/F006278.dat&query=2258&hit=1&index=IPI00031411&px=1&section=5&ave_thresh=22))

**1982 - 1992 628.8303 1255.6461 1255.6449 1 0 K.FTQDVYSAVVK.E**  ([Ions score 29](http://mbp-mascot4/mascot/cgi/peptide_view.pl?file=../data/20091021/F006278.dat&query=1181&hit=1&index=IPI00031411&px=1&section=5&ave_thresh=22))

**2143 - 2151 554.2882 1106.5619 1106.5608 1 0 K.QFELDTLNK.E**  ([Ions score 22](http://mbp-mascot4/mascot/cgi/peptide_view.pl?file=../data/20091021/F006278.dat&query=752&hit=1&index=IPI00031411&px=1&section=5&ave_thresh=22))

**2440 - 2457 665.3657 1993.0753 1993.0745 0 0 K.HFVIDSATGIITLSNLHR.H**  ([Ions score 53](http://mbp-mascot4/mascot/cgi/peptide_view.pl?file=../data/20091021/F006278.dat&query=2131&hit=1&index=IPI00031411&px=1&section=5&ave_thresh=22))

**MiaPaCa A4:**

Match to: **IPI00031411** Score: **37**

**Gene_Symbol=FAT1 Protocadherin Fat 1 lng=4591 # SP[4593,D,22,D]SNP[4646,g,1064,R]SNP[4677,r,1064,R]SNP[4713,i,1125,I]SNP[4791,l,1125,I]SNP[4844,i,1252,I]SNP[4856,h,1273,H]SNP[4889,r,1273,H]SNP[4931,l,1283,P]SNP[4969,p,1283,P]SNP[5000,l,129,V]SNP[5**

Found in search of C:\mgf\Orbidata\081126_ISW1295_MiaPaca2_A4.mgf

Nominal mass (M_r_): **1068827**; Calculated pI value: **4.55**
[truncated: 14,449 more chars]
