# Supplementary material for: A Soluble Form of the Giant Cadherin Fat1 Is Released from Pancreatic Cancer Cells by ADAM10 Mediated Ectodomain Shedding
Source: PLoS One. 2014 Mar 13;9(3):e90461. doi: 10.1371/journal.pone.0090461 (PMC3953070; doi:10.1371/journal.pone.0090461)
Supplement: Materials and Methods S1 — (DOCX) [file pone.0090461.s013.docx]

**Supplementary Materials and Methods**

**Enrichment of large proteins from serum**

To enrich the giant Fat1 ectodomain from serum, 2,5 ml serum were diluted to 15 ml with PBS and centrifuged (10 min, 300g, 4°C) to remove debris and floating cells. Afterwards the supernatant was first ultracentrifuged at 16,500g (20 min, 4°C) and afterwards at 117,000g (90 min, 4°C), using a T-890 titanium ﬁxed angle rotor (Sorvall, Langenselbold, Germany). The pellet was resuspended in 100 µl of PBS.

**Protein degylcosylation**

75 µg lysate and 25µg secretome were deglycosylated using the Glycoprotein Deglycosylation Kit (Calbiochem, Cat. No. 262280). A mixture of five deglycosylases was incubated with the samples for 3h at 37°C and afterwards analysed via Western blotting.

**ESI-MS/MS analysis**

Secretome and serum samples were first gel separated as described above, stained with krypton (Pierce, Rockford, USA), cut into 29 slices per protein band and digested with trypsin. Tryptic peptide mixtures were separated using a nano Acquity UPLC system (Waters GmbH, Eschborn, Germany). Peptides were trapped on a nano Acquity C18 column, 180 μm×20 mm, particle size5 μm (Waters GmbH, Eschborn, Germany). The liquid chromatography (LC) separation was performed on a C18 column (BEH) 130 C18100 μm×100 mm, particle size 1.7 μm (Waters GmbH, Eschborn,Germany) with a ﬂow rate of 400 nl/min. For all gel slices samples,the chromatography was carried out using a 1 h gradient of solvent A (98.9% water, 1% acetonitrile, 0.1% formic acid) and solvent B (99.9% acetonitrile and 0.1% μl formic acid) in the following sequence: from 0 to4% B in 1 min, from4 to 40% B in 40min, from 40 to 60% B in 5 min, from 60 to 85% B in 0.1 min, 6 min at 85% B, from 85 to 0% B in 0.1 min, and9 min at 0% B. The nano UPLC system was coupled online to an LTQ Orbitrap XL mass spectrometer (Thermo Scientiﬁc, Bremen, Germany). The instrument was operated in the sensitive mode with the following parameters: capillary voltage 2400 V; temperature 200 °C, pressure 170 bar; normalized collision energy 35 V, activation time 30,000 ms. Data were acquired by scan cycles of one FTMS scan with are solution of 60,000 and a range from 370 to 2000 m/z in parallel with six MS/MS scans in the ion trap of the most abundant precursor ions. The mgf-ﬁles generated by Xcalibur software (Thermo Scientiﬁc, Bremen, Germany) were used for database searches with the MASCOT search engine (Matrix Science, London, UK; version 2.2)against MSIPI database. The peptide mass tolerance for database searches was set to 5 ppm and fragment mass tolerance to 0.5 Da.Carbamidomethylation of C was set as ﬁxed modiﬁcation. Variable modiﬁcations included oxidation of M and deamidation of NQ. One missed cleavage site in case of incomplete trypsin hydrolysis was allowed. Furthermore, proteins were considered as identiﬁed if more than one unique peptide had an individual ion score exceeding the MASCOT identity threshold (ion score cut-off of 22–23). Identiﬁcation under the applied search parameters refers to False Discovery Rate (FDR) b3.5% and a match probability of pb0.05, where p is the probability that the observed match is a random event. Each slice was analyzed separately and MS/MS data were not merged prior to protein database search to maintain the information about molecular weight of each protein, peptide matches and identiﬁcation score. In this way protein catalogs from human pancreatic cancer cell secretomes (A818-4, BxPC3, MiaPaca2, Paca44, Panc1 and HPDE (human pancreatic duct epithelial)) were established. For fat1 detection in serum, a Krypton stained band at 500 kDa corresponding to the Fat1 signal on Western blots was cut out and analyzed together with a secretome control as a positive control.
